# The n-3 PUFA content of the global lipidomes of NIST SRM 2378, SRM 1950, and intralaboratory quality control materials

**DOI:** 10.1016/j.jlr.2025.100970

**Published:** 2025-12-26

**Authors:** Ken D. Stark, Klaudia E. Steckel, Michael Kiebish, Juan J. Aristizabal-Henao

**Affiliations:** 1 Department of Kinesiology and Health Sciences, University of Waterloo, Waterloo, ON, Canada; 2 Lipotype GmbH, Dresden, Germany; 3 BPGbio Inc., Waltham, MA, USA

**Keywords:** omega-3 PUFAs, lipidomics, blood biomarkers, dietary intake, EPA, DHA, MS/MS, SRM 1950, SRM 2378

## Abstract

The regular use of commercially available Standard Reference Materials (SRM) and intralaboratory quality control materials, which are important tools, is essential for standardizing and ensuring high-quality lipidomic analyses. These materials should also be relevant for the research application. To support nutritional lipidomic research, the global lipidome of materials derived from individuals consuming a range of n-3 PUFAs was determined. Nontargeted lipidomics were completed on SRM 2378 (serum), SRM 1950 (plasma), and intralaboratory quality control (plasma) generated from individuals with low omega-3 and high omega-3 status. SRM 2378 includes materials generated from individuals consuming fish oil (SRM 2378-1), flaxseed oil (SRM 2378-2), and no supplements (SRM 2378-3). Specific lipids with differences were identified using fold-based and absolute differences in semiquantitated concentrations. Individual lipids containing 20:5 and 22:6 were highly variable and largely reflected ad hoc intake estimates of EPA and DHA. Fold-based approaches identified low-abundant lipids that differed, whereas absolute differences identified high-abundant lipid species that differed. In addition, differences due to dietary fatty acid intakes were more dramatic than differences between serum and plasma in these nontargeted analyses. The dietary intake of EPA and DHA can impact lipidomic profiles, which should be considered by lipidomic analysts. These results also suggest that comprehensive dietary assessments should be considered during the development of reference and quality control materials.

Lipidomic analyses can provide biomolecular phenotypic information about an organism. The lipidomic phenotype can reflect genetic traits, but it is also influenced by physiological and environmental influences on metabolism. As such, the lipidome of an individual can provide insights into the health status of an individual, and lipidomics is an important approach for biomarker discovery. The field of lipidomics “emerged” over 20 years ago (1, 2, 3), yet efforts to standardize the field and analytical procedures are ongoing (4, 5). The use of reference materials that can be shared across analytical laboratories and tested against different MS platforms and workflows is one strategy recommended to help “harmonize” data across the field (6, 7).

Standard Reference Material (SRM) 1950 Metabolites in Frozen Human Plasma has been used in attempts to establish a referral lipidome for human plasma (6, 7, 8, 9, 10, 11, 12). SRM 1950 was developed by the National Institute of Standards and Technology (NIST) in collaboration with the National Institute of Diabetes and Digestive and Kidney Diseases in 2006 to support interlaboratory metabolomics analysis (6, 13). It was created by pooling human plasma from 100 individuals whose ethnicity reflected that of the US population based on the 2000 Census. While the use of the commercially available SRM 1950 that can be shared across laboratories is highly recommended as a strategy to standardize the lipidomic field (14), it has also been recognized that it may not meet the quality control needs of a specific research project (7). The design of SRM 1950 to represent the plasma of the general US population may limit its application, and it may not represent the plasma of a subpopulation of research interest. NIST recognized this challenge and developed plasma candidate RM 8231 to represent diabetic (RM 8231-1), hypertriglyceridemic (RM 8231-2), and African-American (RM 8231-3) populations (11, 15). However, appropriate reference materials may not exist for all subpopulations of interest or research questions. The cost of commercial reference materials can also limit their routine use, and they cannot be used indefinitely, as there is a limited stock. Laboratories need to consider developing internal reference materials, such as combining small aliquots of the samples to be analyzed for a project into a pool for quality control within their lipidomic workflow plans (7). Venous plasma has been recommended for lipidomic projects over serum as coagulation involves a cascade of reactions involving and changing concentrations of lipids such as lysophospholipids and various oxylipins that are of interest due to their bioactivity (16), but serum is routinely collected in clinical research and may be the only blood type available.

NIST SRM 2378 Fatty Acids in Frozen Human Serum is an SRM formally introduced in 2015 as part of an NIST Fatty Acid Quality Assurance Program in collaboration with the National Institutes of Health Office of Dietary Supplements to help standardize the measurement of individual fatty acids (17). It is well documented that dietary habits can impact circulating blood lipids. Clinically, the focus has traditionally been on examining cholesterol in relation to lipoprotein fractions along with triacylglycerols (TGs). However, blood levels of n-3 PUFAs have been examined extensively, and their use in clinical assessments have increased as the intake of EPA (20:5n-3) and DHA (22:6n-3) is associated with various benefits from infant brain development to cardiovascular health (18, 19, 20, 21). Different dietary intakes of EPA and DHA are associated with unique lipidomic profiles in plasma, serum, erythrocytes, and whole blood (22, 23), but our understanding of how dietary n-3 PUFA impacts the blood lipidome is incomplete. These fatty acid measurements are typically done by gas chromatography coupled with flame ionization detection or MS. SRM 2378 consists of pools of serum from healthy individuals who *1*) were prescribed at least 1 g/d of fish oil for at least 1 month prior to collection, *2*) were prescribed at least 1 g/d of flaxseed oil for at least 1 month prior to collection, and *3*) were eating a “normal” diet without dietary fish or flaxseed oil supplements for 1 month prior to collection (17). While SRM 2378 has been characterized at the fatty acid level (17, 24), lipidomic profiling using liquid chromatography MS/MS-based analyses has not been completed for these SRMs generated through the intake of fatty acid supplements.

Presently, lipidomic profiling of SRM 1950, SRM 2378-1 (fish oil), SRM 2378-2 (flax oil), and SRM 2378-3 was completed with an untargeted method with an ultra-HPLC-MS/MS (UHPLC-MS/MS) platform. In addition to the SRMs, we also examined quality control materials that were generated internally for routine fatty acid analyses. These intralaboratory quality control (ILQC) materials were generated from the plasma of individuals with low omega-3 PUFA status (ILQC LO3) and from those with high omega-3 PUFA status (ILQC HO3). While a focus was examining omega-3 PUFA differences in the lipidomes, we also made comparisons to check if lipidomic differences due to previously documented preanalytical procedures (16, 25) were also present in the reference materials examined herein.

## Materials and methods

### Reference materials

All NIST plasma and serum samples were collected after informed consent under approved Institutional Review Board protocols reviewed by the NIST Human Subjects Protection Office. Details about the preparation of SRM 1950 (13) and SRM 2378 (24) have been published previously, and these materials are available commercially from NIST. In brief, SRM 1950 is a plasma material produced from 100 fasted individuals representing the US population, as defined by race, sex, and health in the age range of 40–50 years. For SRM 1950, the plasma was isolated using lithium heparin as an anticoagulant. SRM 2378 is a set of three serum materials. SRM 2378-1 was produced from three healthy donors who took 1,000 mg/day of fish oil supplements for a minimum of 1 month. SRM 2378-2 was produced from three healthy donors who took 1,000 mg/day of flaxseed oil supplements for a minimum of 1 month prior to collection. SRM 2378-3 was produced from three healthy donors who did not take either fish or flaxseed oil supplements for 1 month prior to collection. The fatty acid content of the fish oil and flaxseed oil used in the intervention was not reported.

The ILQC LO3 and ILQC HO3 plasma samples were collected in September 2015 after informed written consent under approved protocols as reviewed by the University of Waterloo Human Ethics Committee and abided by the Declaration of Helsinki principles. All participants were either undergraduate or graduate students completing research in the Department of Kinesiology and Health Sciences at the University of Waterloo. A trained technician collected 50–60 ml of venous blood from the antecubital vein of four participants who had fasted overnight into evacuated tubes (Vacutainer; Becton Dickinson, Franklin Lakes, NJ) with EDTA as an anticoagulant. After centrifugation, plasma samples from two individuals were pooled together for the ILQC LO3 and ILQC HO3. ILQC LO3 was produced from one male (21 years of age) and one female (25 years of age) who did not consume seafood or take n-3 PUFA supplements as part of their regular diet. ILQC HO3 was produced from one male (24 years of age) and one female (28 years of age) known to consume seafood and/or fish oil supplements as part of their regular diet. The ILQC LO3 and ILQC HO3 pooled plasma were then aliquoted to cryovials (500 μl each) and frozen and stored at −80°. The fatty acid compositions of ILQC LO3 and ILQC HO3 were determined (and continued to be determined for ILQC checks) using gas chromatography with flame ionization methods (26), but the composition values have not been published.

### Analyses

The reference materials were analyzed using a nontargeted, semiquantitative lipidomic approach using UHPLC-MS/MS with iterative exclusion data-dependent acquisition and lipid identifications (IE-Omics), as described previously (11). Lipidomic analytical procedures and resultant use of data were approved by the University of Waterloo Human Ethics Committee.

### Lipid extraction

Lipids were extracted from the plasma and serum reference materials in quadruplicate using the Folch-based extraction (27), which is known to cover a wide range of lipids (28). Briefly, 50 μl of the sample was added to 3 ml 2:1 chloroform/methanol (v/v), which contained deuterium-labeled internal standards for the major lipid classes (EquiSPLASH Lipidomix; Avanti Polar Lipids, Alabaster, AL). For each sample, 0.5 μg of each internal standard were added by adding 50 μl of an EquiSPLASH diluted 10-fold solution (providing 10 μg of each standard/ml). Samples were then vortexed, and then 500 μl of saline buffer were added. The samples were then inverted three times to mix and then centrifuged at 1,800 *g*_n_ for 5 min to separate the aqueous and organic layers. The organic layer that contained the lipids was collected, dried under nitrogen gas, and then reconstituted in 100 μl of isopropanol to ensure lipid solubility while preventing evaporation (29). Samples in isopropanol were stored at 4°C until analysis by UHPLC-MS/MS.

### Ultra-HPLC/MS/MS

The samples in isopropanol were introduced by an autosampler (10 μl) into an Agilent 1290 UHPLC system with an Acquity Premier CSH C18 column (2.1 mm × 150 mm, 1.7 μm; Waters) at the column compartment temperature of 50°C. Lipids were separated using a mobile phase program consisting of A: 60:40 acetonitrile:water (v/v) and B: 90:10 isopropanol:acetonitrile (v/v), with A and B both containing 10 mM ammonium formate, 0.1% formic acid, and 0.1 μM reserpine (lock mass). The gradient elution used a flow rate of 250 μl/min with the program starting at 68% A and 32% B, with a linear increase to 55% B from 0 to 5 min, a linear increase to 65% from 5 to 8 min, a 2 min hold at 65% B followed by a linear increase to 80% B from 10 to 14 min, a linear increase to 90% B from 14 to 25 min, a linear increase to 100% B from 25 to 25.5 min, a 90 s hold at 100% B, a drop to 32% B at 27.1 min, and equilibration until the 30 min mark. Analytes were introduced into a Thermo Q-Exactive Plus mass spectrometer from the UHPLC using electrospray ionization. MS was completed using a polarity-switching full-scan method at 17,500 resolution with spray voltages +3.5 kV and −2.5 kV in the positive and negative ion modes, respectively, and a scan range *m/z* 120–1,600. Centroided spectra were lock-mass corrected automatically during acquisition using reserpine [M + H]^+^ *m/z* 609.28121 and [M + formate]^-^ *m/z* 653.27104. Representative plasma and serum pools were used as targets in pseudo top-40 data-dependent MS/MS experiments with iterative exclusion lists using IE-Omics (30). For lipid identifications, three to seven points per peak were generated using a 6-s dynamic exclusion for MS/MS runs in pseudo top-40 data-dependent acquisition. These were not integrated as LipidMatch uses MS/MS runs only for lipid identifications. Peak area integration for semiquantitation was done using full-scan runs only (with polarity switching). At a resolution of 17,500 on the Q-Exactive Plus (full scan with polarity switching), 20–30 points per peak were obtained on average from chromatographic peaks that were 15–25 s in duration.

### Data processing and statistical analyses

Lipids were putatively identified, and peaks were integrated using LipidMatch software (31). Semiquantitative values were generated from the integrated peaks by normalizing for sample volume and the most appropriate deuterated internal standard subclass amount as described earlier (11). For lipid classes without representative standards, EquiSPLASH internal standards were chosen based on structure, polarity, adduct, and/or proximity by retention time. The mass spectra of lipid species containing rare fatty acyls and/or rare combinations of fatty acids were inspected manually but not confirmed by authentic standards. For quality control, a plasma sample purchased from BioIVT (Woodbury, NY) was run with the analytical batch as technical quadruplicates. The median %CV across these plasma technical quadruplicates for all lipids was ∼12%. Lipid class and subclass summations, and identification of top 10 individual lipids with the largest semiquantitative differences between SRM 2378-1 with SRM 2378-3, and ILQC HO3 with ILQC LO3 to contrast reference materials between individuals with low and high EPA + DHA status were completed using IBM SPSS Statistics, version 30.0.0.0 (172). This was also completed for differences between SRM 1950, SRM 2378-3, and ILQC LO3 to determine if lipidomic differences due to the procedure might also be present. These were compared statistically using one-way ANOVA with post hoc comparisons completed using Tukey's procedure when the six reference materials were compared and independent *t*-tests for the top 10 individual lipid pairings. Lipid identifications of the reference materials were compared by preparing a Venn diagram using InteractiVenn (32). Prior to analyses using MetaboAnalyst 6.0 (33), individual lipids with values of 0 were manually assigned a consistent value of 1.00E-11 rather than using the automated missing value estimation options. The MetaboAnalyst automated approach replaces zero values with a value based on the other values reported within a feature, resulting in different values being used to replace zero values across features. Within MetaboAnalyst, the data were log-transformed and Pareto scaled (34) before principal component analyses (PCA) and volcano plots for fold differences were performed. For the volcano plots, a fold change threshold of 2.0 and a false discovery rate *P* value threshold of 0.01 were set.

## Results

### Lipid classes and subclasses

**Figure 1:**
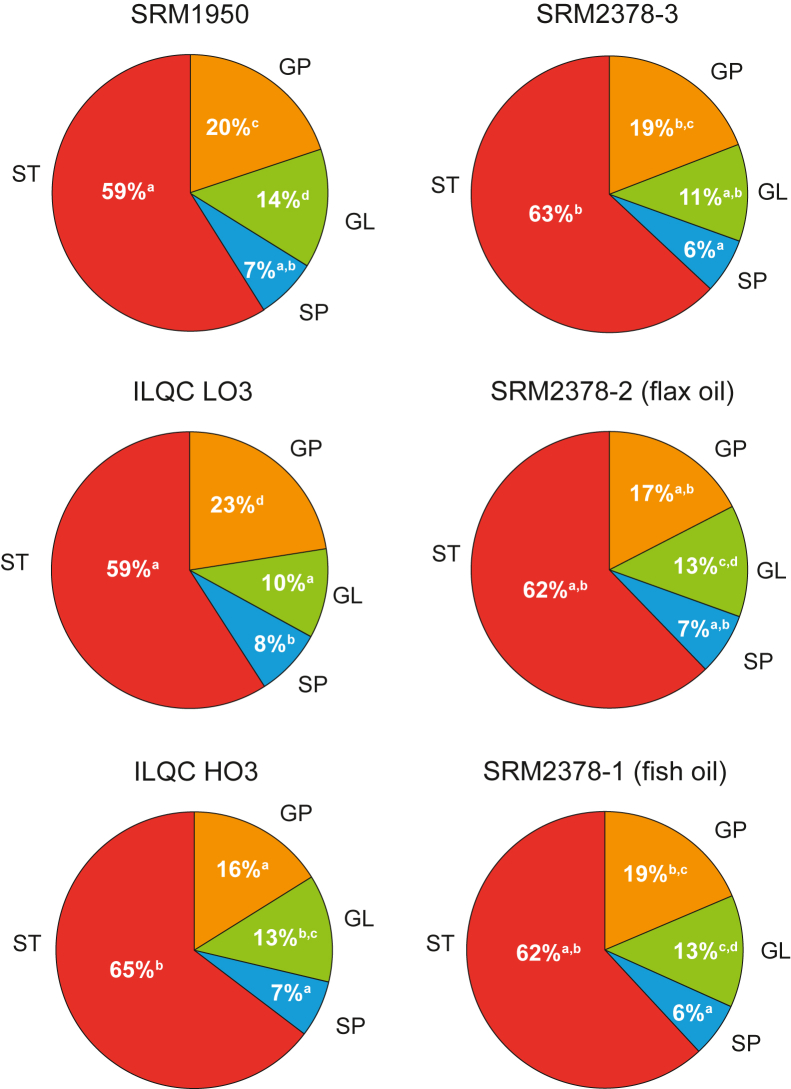
Relative distribution of the main lipid classes in between reference materials from humans. Lipid classes with different superscripts across pie charts are significantly different by Tukey’s post hoc test (*P* < 0.05) after a significant one-way ANOVA *F* value (*P* < 0.05). GL, glycerolipid; GP, glycerophospholipid; HO3, high omega-3; ILQC, intra-laboratory quality control; LO3, low omega-3; SP, sphingolipid; SRM, National Institutes of Standards and Technology Standard Reference Material; ST, sterols.

The percentage of lipid classes by abundance differed slightly but significantly across the reference materials (Fig. 1). Sterols (STs) were the most abundant lipids in all the samples, ranging from 59% of the lipid abundance in SRM 1950 and ILQC LO3 to 65% in ILQC HO3, followed by glycerophospholipids, ranging from 16% in ILQC HO3 to 23% in ILQC LO3. Glycerolipids ranged from 10% in ILQC LO3 to 14% in SRM 1950, whereas sphingolipids ranged from 6% in SRM 2378-1 (fish oil) and 3–8% in SRM 2378 ILQC LO3. However, there were significant differences in the total concentrations of lipids across the materials (Table 1) as ILQC LO3 (3,584 ± 243 nmol/ml), SRM 1950 (3,816 ± 43 nmol/ml), and SRM 2378-3 (4,039 ± 141 nmol/ml) were significantly lower than SRM 2378-1 (4,690 ± 378 nmol/ml), ILQC HO3 (4,847 ± 69 nmol/ml), and SRM 2379-2 (flax oil) (5,263 ± 255 nmol/ml). These total lipid differences were mainly a result of higher concentrations of two of the most abundant subclasses of lipids, cholesteryl esters (CEs) and TGs, in SRM 2378-1, ILQC HO3, and SRM 2379-2. There were no significant differences in the concentrations of the other highly abundant phosphatidylcholine (PC) subclasses across any of the reference materials. Interestingly, however, the concentrations of the lysophosphatidylcholine (LPC) subclass were the highest in the serum-based SRM 2378-1 (133.0 ± 0.7 nmol/ml), SRM 2378-2 (135.9 ± 2.8 nmol/ml), and SRM 2378-3 (151.9 ± 0.7 nmol/ml), followed by SRM 1950 (108.9 ± 4.2 nmol/ml) and then ILQC LO3 (57.8 ± 1.1 nmol/ml) and ILQC HO3 (59.8 ± 1.8 nmol/ml).

**Table 1:** Semiquantitative concentrations of lipid subclasses in reference materials from humans

Lipid	SRM 1950	SRM 2378-3	SRM 2378-2 (flax oil)	SRM 2378-1 (fish oil)	ILQC LO3	ILQC HO3
	nmol/mL					
Glycerolipids						
TG	518 ± 4^c^	446 ± 32^b^	667 ± 8^d^	599 ± 32^d^	365 ± 35^a^	598 ± 30^d^
DG	12.4 ± 0.3^b^	12.3 ± 0.9^b^	18.0 ± 0.6^d^	14.5 ± 0.1^c^	9.1 ± 0.7^a^	10.6 ± 1.2^a,b^
OxTG	0.39 ± 0.03^d^	0.17 ± 0.01^a^	0.25 ± 0.01^b,c^	0.45 ± 0.04^d^	0.20 ± 0.01^a,b^	0.27 ± 0.01^c^
GlycoGL	0.25 ± 0.05^a,b^	0.22 ± 0.02^a,b^	0.26 ± 0.02^a,b^	0.18 ± 0.01^a^	0.30 ± 0.03^b^	0.22 ± 0.06^a,b^
Glycerophospholipids						
PC	504 ± 7	497 ± 49	618 ± 4	561 ± 78	566 ± 48	563 ± 56
LPC	109 ± 4^b^	133 ± 1^c^	136 ± 3^c^	152 ± 1^day^	58 ± 1^a^	60 ± 2^a^
Ether PC	72.0 ± 1.1^a^	66.7 ± 6.0^a^	75.0 ± 0.4^a^	71.0 ± 7.2^a^	93.2 ± 5.7^b^	68.5 ± 4.9^a^
Ether PE	25.8 ± 0.7^a,b^	29.2 ± 2.7^a,b,c^	32.1 ± 0.3^c^	30.0 ± 1.7^b,c^	33.4 ± 2.3^c^	24.9 ± 1.6^a^
PI	20.0 ± 0.2^a,b^	15.4 ± 1.7^a^	19.1 ± 1.5^a,b^	23.3 ± 3.1^b^	16.2 ± 1.7^a^	29.2 ± 2.2^c^
PE	13.6 ± 0.3^a^	13.9 ± 1.3^a,b^	17.4 ± 0.2^a,b^	17.5 ± 1.5^b^	23.5 ± 1.5^c^	21.4 ± 2.2^c^
DMPE	5.90 ± 0.76^a^	7.23 ± 1.01^a,b^	7.01 ± 0.53^a,b^	6.68 ± 0.23^a,b^	8.47 ± 0.89^b^	6.61 ± 0.05^a^
LPE	4.21 ± 0.23^c^	4.56 ± 0.15^d^	4.00 ± 0.09^c^	5.86 ± 0.07^e^	2.55 ± 0.04^a^	3.36 ± 0.06^b^
Ether PS	2.93 ± 0.24	3.99 ± 1.93	5.87 ± 0.78	3.78 ± 1.39	3.99 ± 1.95	3.53 ± 2.06
OxPL	1.42 ± 0.03^d^	0.99 ± 0.08^a^	1.31 ± 0.06^c,d^	1.11 ± 0.04^a,b^	1.20 ± 0.04^b,c^	1.15 ± 0.02^b^
PS	0.25 ± 0.03^a^	0.69 ± 0.10^b,c^	0.65 ± 0.08^b,c^	0.79 ± 0.06^c^	1.11 ± 0.10^d^	0.55 ± 0.05^b^
Prenols						
CoQ	0.51 ± 0.02^b^	0.44 ± 0.02^a^	0.74 ± 0.02^d^	0.60 ± 0.02^c^	0.49 ± 0.03^a,b^	0.58 ± 0.02^c^
Sphingolipids						
SM	264 ± 12^a,b^	249 ± 1^a^	365 ± 3^d^	283 ± 9^b^	273 ± 4^b^	311 ± 9^c^
Cer	8.97 ± 0.24^b^	9.66 ± 0.62^b^	13.88 ± 0.37^d^	10.90 ± 0.49^c^	6.39 ± 0.44^a^	7.32 ± 0.17^a^
HexCer	2.11 ± 0.03^a,b^	1.88 ± 0.13^a^	2.66 ± 0.11^d,e^	2.40 ± 0.08^c,d^	2.23 ± 0.05^b,c^	2.76 ± 0.14^e^
STs						
CE	2,249 ± 49^a^	2,546 ± 107^a,b^	3,279 ± 258^c^	2,905 ± 263^b,c^	2,119 ± 162^a^	3,134 ± 26^c^
Total	3,816 ± 43^a^	4,039 ± 141^a^	5,263 ± 255^b^	4,690 ± 378^b^	3,584 ± 243^a^	4,847 ± 69^b^

Values are mean ± SD.

Different alphabetical superscripts across lipid subclasses indicate significant differences by Tukey’s post hoc test (*P* < 0.05) after a significant *F* value by one-way ANOVA (*P* < 0.05).

CE, cholesteryl ester; Cer, ceramide; CoQ, coenzyme quinone; DG, diacylglycerol; DM, dimethyl; DMPE, dimethyl phosphatidylethanolamine; GlycoGL, glycosyldiradylglycerol; HexCer, hexosylglycoceramide; HO3, high omega-3; ILQC, intra-laboratory quality control; L, lyso; LO3, low omega-3; Ox, oxidized; PC, phosphatidylcholine; PE, phosphatidylethanolamine; PI, phosphatidylinositol; PL, phospholipid; PS, phosphatidylserine; SM, sphingomyelin; SRM, National Institutes of Standards and Technology Standard Reference Material; TG, triacylglycerol.

### Individual lipid identifications

**Figure 2:**
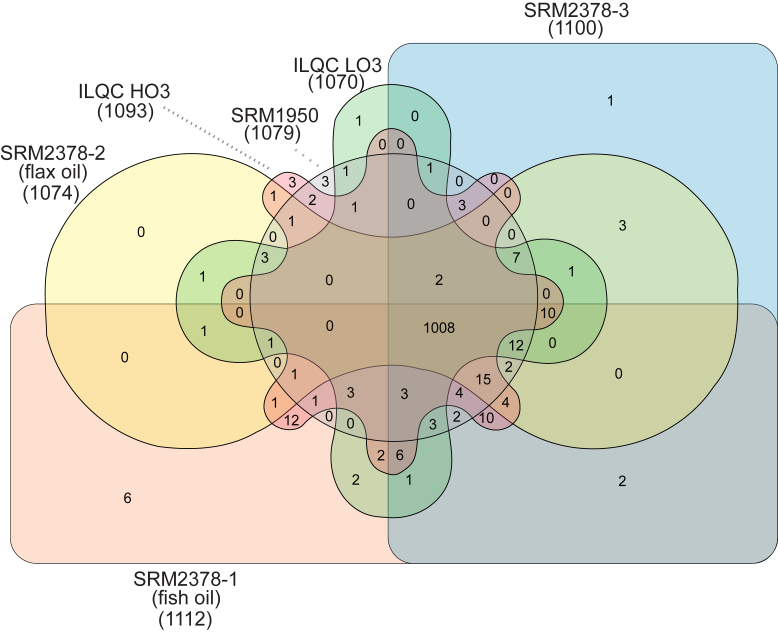
Common and unique number of lipid identifications between reference materials from humans. HO3, high omega-3; ILQC, intra-laboratory quality control; LO3, low omega-3; SRM, National Institutes of Standards and Technology Standard Reference Material.

A total of 1,147 unique lipids were identified in the plasma/serum materials, with 1,008 lipids being common across the six materials. The highest number of individual lipids identified was 1,112 in SRM 2378-1 (fish oil), and the lowest was 1,070 in ILQC LO3 (Fig. 2). SRM 2378-1 (fish oil) also had the highest number of unique lipids (n = 6), which included an oxidized TG (OxTG 18:1_18:1_20:4(OOH)), PC 18:3_20:5, TG 10:0_18:2_18:3, TG 14:0_16:0_16:1, TG 18:2_18:2_8:0, and a phosphatidylethanolamine (PE 17:0_20:5).

Monomethyl phosphatidylethanolamine 18:1_20:5, OxPC 18:0_18:2(1O), and OxPC 18:0_20:4(1O) were unique to SRM 1950, and monomethyl phosphatidylethanolamine 16:1_22:6, OxTG 18:1_18:1_18:3(OOH), and a phosphatidylserine (PS; phosphatidylinositol [PI] 16:0_18:0) were unique to ILQC HO3. Diacylglycerol 18:2_18:2 was observed only in ILQC LO3, and a plasmanyl phosphatidyl choline (PC O-16:0/17:0) was unique to SRM 2378-3. There was no individual lipid unique to SRM 2378-2 (flax oil).

### Lipidome comparisons

**Figure 3:**
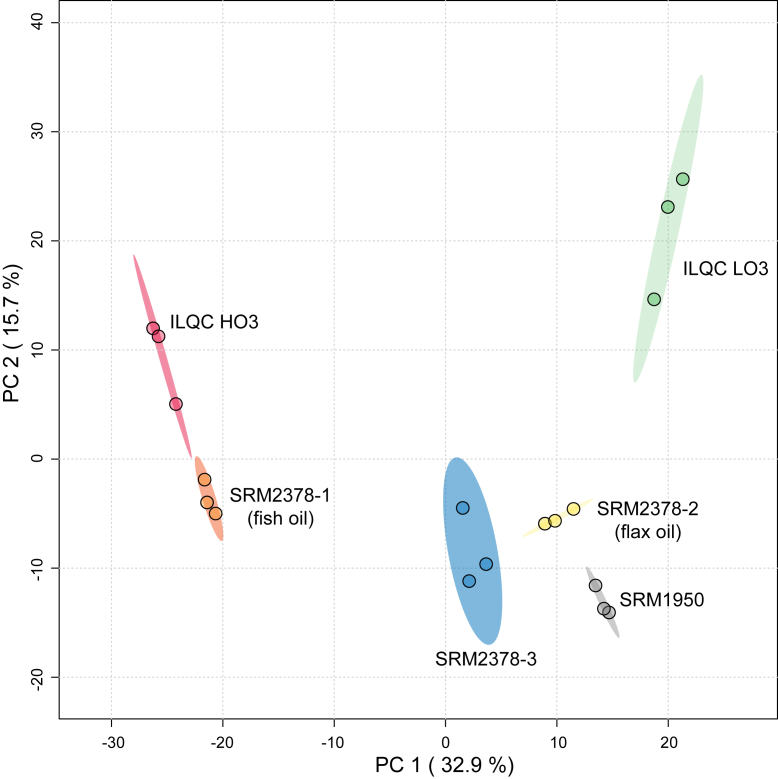
Principal component analysis score plot for Pareto-scaled semiquantitative lipidomics profiles (1,147 lipids) of reference materials from humans consuming different amounts of omega-3 PUFAs. ILQC HO3 (high omega-3), ILQC LO3 low omega-3), SRM 1950, SRM 2378-1 (fish oil), SRM 2378-2 (flax oil), and SRM 2378-3 (no supplement).

Clustering of the reference material/quality control material replicates was observed by PCA (scores plot in Fig. 3). Principal component 1 accounted for 32.9% of the variance, whereas principal component 2 accounted for 15.7% of the variance. SRM 1950, SRM 2378-2 (flax oil), and SRM 2378-3 (no supplements) were all located in the same quadrant, but all the materials were significantly different from each other by PEMANOVA (*P* < 0.001). SRM 1950 versus SRM 2378-1 (fish oil) were the most distinct from each other, having the largest pseudo-*F* value (812.33), whereas SRM 2378-2 (flax oil) versus SRM 2378-3 (no supplements) had the smallest pseudo-*F* value (12.94). When principal component 3, which accounted for an additional 10.3% of the variance, was examined, the 3-dimensional plot (not shown) highlighted an additional third-dimensional separation of SRM 1950 from SRM 2378-2 (flax oil) and SRM 2378-3. A relatively large third-dimensional separation between SRM 2378-1 (fish oil) and ILQC HO3 was also evident.

### Omega-3 PUFAs and the lipidome

**Figure 4:**
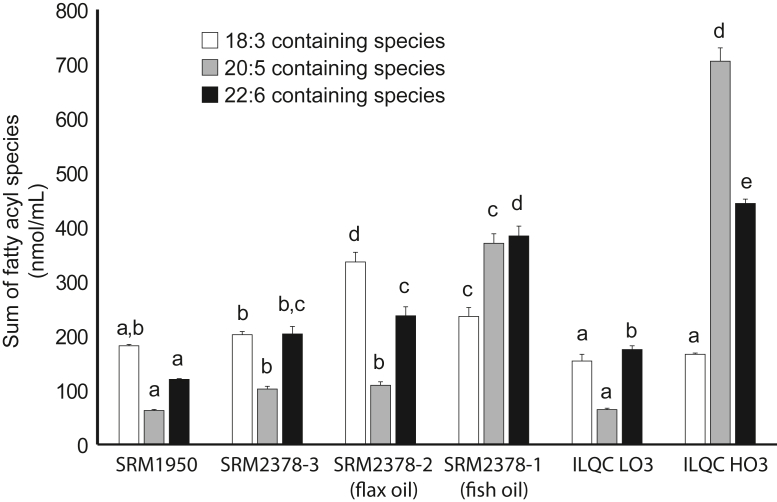
Semiquantitative concentrations of lipid species containing omega-3 polyunsaturated fatty acyls (18:3, 20:5, and 22:6) in plasma/serum reference materials from humans. Columns within a fatty acyl species with different alphabetical labels are significantly different by Tukey’s post hoc test (*P* < 0.05) after a significant *F* value by one-way ANOVA (*P* < 0.05). ILQC HO3 (high omega-3), ILQC LO3 low omega-3), SRM 1950, SRM 2378-1 (fish oil), SRM 2378-2 (flax oil), and SRM 2378-3 (no supplement).

Visual inspection of the PCA score plot indicated that the reference materials associated with the intakes of the long-chain PUFAs from fish oil, specifically EPA and DHA, had considerably different lipidomes as compared with other reference materials derived from individuals with lower EPA and DHA intakes, including the individuals who consumed flaxseed oil that provided alpha linolenic acid (18:3n-3). Lipid species containing 18:3, 20:5, and 22:6 were identified, and semiquantitative concentrations of the reference material/quality control material were summed (Fig. 4). Since our UHPLC-MS/MS analyses did not include orthogonal approaches, such as ion mobility or ozone-induced dissociation, the 18:3-containing lipid species included lipid species with both 18:3n-3 and 18:2n-6 fatty acyls. EPA (20:5)-containing lipid species had the greatest dynamic range across the reference materials. The sums of the EPA species were low (62–108 nmol/ml), unless the reference material came from individuals consuming fish and/or fish oil. SRM 2378-1 (fish oil) had 370 ± 18 nmol/ml, and ILQC HO3 had 705 ± 24 nmol/ml of 20:5-containing lipids. Lipids containing DHA followed a similar pattern, but on a more limited range, as the lipids in materials with low DHA content ranged from 120 to 237 nmol/ml, whereas SRM 2378-1 (fish oil) had 384 ± 18 nmol/ml and ILQC HO3 had 443 ± 8 nmol/ml of DHA-containing lipids. The amount of lipids containing 18:3 was uniquely high in SRM 2378-2 (flax oil) at 336 ± 18 nmol/ml, whereas the remaining materials ranged from 153 to 235 nmol/ml. Gas chromatography analyses of SRM 2378 are available and indicate that flaxseed oil supplementation can increase 18:3n-3 by approximately 90% and 18:3n-6 by approximately 50%, whereas fish oil supplementation increases 18:3n-3 by approximately 80% and reduces 18:2n-6 by approximately 20% as compared with SRM 2378-3 (no supplements) (24). It is important to note that SRM 2378-3 (no supplements) had over 60% higher amounts of 20:5- and 22-6-containing lipids, as compared with SRM 1950 but no difference in 18:3-containing lipids.

The 20:5- and 22:6-containing lipids of the ILQC and SRM 2378 materials were sorted by lipid class for additional insights (Table 2). The ILQC HO3 and SRM 2378-1 materials that were created from individuals who consumed fish and/or fish oil supplements had higher amounts of 20:5 in all lipid classes as compared with the other materials. The 22:6 content was also higher in the lipid classes except for PE, ether PE, and ether PS in ILQC HO3 as compared with ILQC LO3 and ether PE in SRM 2378-1, as compared with the other SRM 2378 levels. Interestingly, the material derived from individuals consuming flaxseed oil supplements (SRM 2378-2) had the same amount of 20:5- and 22:6-containing lipids in their lipid classes as those who took no supplements (SRM 2378-3), except for higher 20:5 and 22:6 in TG and slightly lower 22:6 in PI.

**Table 2:** Semiquantitative comparison of lipids containing 20:5 and 20:6 in ILQC and SRM 2378 by lipid class

Lipid	ILQC LO3	ILQC HO3	SRM 2378-3 (no supplements)	SRM 2378-2 (flaxseed oil)	SRM 2378-1 (fish oil)
Containing EPA		nmol/mL			
PC	14.65 ± 0.34	78.51 ± 3.90^a^	16.11 ± 0.92^1^	18.17 ± 0.13^1^	45.86 ± 2.22^2^
Ether PC	1.67 ± 0.06	5.78 ± 0.27^a^	1.23 ± 0.05^1^	1.17 ± 0.03^1^	3.89 ± 0.26^2^
PE	0.67 ± 0.02	2.35 ± 0.07^a^	0.42 ± 0.05^1^	0.41 ± 0.01^1^	1.12 ± 0.01^2^
Ether PE	1.00 ± 0.09	4.60 ± 0.61^a^	1.00 ± 0.10^1^	1.08 ± 0.02^1^	3.78 ± 0.38^2^
PI	0.006 ± 0.001	0.316 ± 0.017^a^	0.010 ± 0.001^1^	0.013 ± 0.001^1^	0.077 ± 0.004^2^
CE	42.67 ± 2.90	571.16 ± 27.38^a^	78.47 ± 6.29^1^	79.43 ± 6.69^1^	295.63 ± 15.00^2^
TG	3.06 ± 0.09	41.71 ± 0.93^a^	4.55 ± 0.20^1^	8.27 ± 0.31^2^	19.28 ± 0.41^3^
Total	49.3 ± 2.9	628.5 ± 27.2^a^	86.1 ± 5.8^1^	91.0 ± 6.6^1^	325.6 ± 15.4^2^
Containing DHA		nmol/mL			
PC	46.73 ± 2.92	72.36 ± 6.08^a^	45.15 ± 2.90^1^	48.40 ± 0.55^1^	71.59 ± 7.75^2^
Ether PC	4.57 ± 0.12	5.60 ± 0.28^a^	3.30 ± 0.18^1^	3.47 ± 0.04^1^	5.65 ± 0.34^2^
PE	5.28 ± 0.39	6.66 ± 0.83	3.06 ± 0.33^1^	3.24 ± 0.15^1^	4.70 ± 0.63^2^
Ether PE	4.91 ± 0.56	5.36 ± 0.75	4.72 ± 0.80	5.59 ± 0.03	6.58 ± 1.07
PI	0.469 ± 0.011	2.096 ± 0.078^a^	0.498 ± 0.022^1^	0.374 ± 0.020^2^	0.928 ± 0.049^3^
Ether PS	0.121 ± 0.036	0.192 ± 0.105	0.076 ± 0.021^1,2^	0.039 ± 0.015^1^	0.131 ± 0.034^2^
CE	102.0 ± 6.2	270.2 ± 20.0^a^	134.6 ± 16.6^1^	157.0 ± 16.8^1^	263.3 ± 6.7^2^
TG	10.30 ± 1.06	79.88 ± 4.15^a^	11.71 ± 0.92^1^	18.23 ± 0.42^2^	30.31 ± 1.33^3^
Total	113.6 ± 6.3	356.2 ± 15.9^a^	147.6 ± 15.9^1^	176.6 ± 16.7^1^	296.3 ± 8.2^2^

Values are mean ± SD.

Different numerical superscripts across lipid subclasses indicate significant differences by Tukey’s post hoc test (*P* < 0.05) after a significant *F* value by one-way ANOVA (*P* < 0.05).

CE, cholesteryl ester; HO3, high omega-3; ILQC, intra-laboratory quality control; LO3, low omega-3; PC, phosphatidylcholine; PE, phosphatidylethanolamine; PI, phosphatidylinositol; PS, phosphatidylserine; SRM, National Institutes of Standards and Technology Standard Reference Material; TG, triacylglycerol.

a Significantly different than ILQC LO3 by independent *t*-test (*P* < 0.05).

Differences in lipidomes based on fish/fish oil consumption were examined further by identifying the top 10 individual lipids that were higher or lower in serum-based SRM 2378-1 (fish oil) versus SRM 2378-3 (no supplements) (Table 3, Supplemental Table S1) and in plasma-based ILQC HO3 versus ILQC LO3 (Table 4, Supplemental Table S2). The individual lipid species were ranked by fold and concentration differences.

**Table 3:** Lipids higher in SRM 2378-1 relative to SRM 2378-3

Lipid	Difference (nmol/ml)	Fold change	*P*^a^
Largest fold changes—top 10			
TG 18:1_20:4_8:0	0.255	2.55E+10	<0.001
TG 18:2_18:2_8:0	0.138	1.38E+10	<0.001
TG 10:0_16:0_20:5	0.112	1.12E+10	<0.001
TG 10:0_18:2_18:3	0.098	9.79E+09	<0.001
TG 10:0_10:0_14:0	0.058	5.85E+09	<0.001
TG 17:1_20:4_20:5	0.017	1.71E+09	<0.001
TG 17:0_20:5_22:6	0.011	1.13E+09	<0.001
TG 17:2_17:2_17:2	0.007	7.17E+08	<0.001
OxTG 16:0_22:6_18:1 (Ke_or_Epoxy)	0.004	4.39E+08	<0.001
PG 16:0_18:2	0.004	4.36E+08	<0.001
Largest differences—top 10			
CE 20:5	217	3.77	<0.001
CE 22:6	129	1.96	<0.001
PC 16:0_20:5	19.2	3.15	<0.001
PC 16:0_22:6	16.0	1.55	<0.01
SM d18:1/24:1	11.7	1.46	<0.05
TG 16:0_16:1_18:1	9.69	1.39	<0.001
TG 16:0_16:0_18:1	8.44	1.46	<0.05
PC 18:0_22:6	7.84	1.76	<0.05
PC 18:0_20:5	7.52	2.27	<0.01
LPC 16:0	7.45	1.13	<0.01

CE, cholesteryl ester; L, lyso; Ox, oxidized; PC, phosphatidylcholine; PG, phosphatidylglycerol; SM, sphingomyelin; SRM, National Institutes of Standards and Technology Standard Reference Material; TG, triacylglycerol.

a As determined by false discovery rate for largest fold changes using MetaboAnalyst 6.0 and by independent *t*-test for largest differences using IBM SPSS Statistics (version 30.0.0.0).

**Table 4:** Lipids higher in ILQC HO3 relative to ILQC LO3

Lipid	Difference (nmol/ml)	Fold change	*P*^a^
Largest fold changes—top 10			
TG 16:0_20:5_20:5	1.005	1.00E+11	<0.001
TG 16:1_20:5_20:5	0.246	2.46E+10	<0.001
TG 20:5_20:5_22:6	0.159	1.59E+10	<0.001
TG 20:5_20:5_20:5	0.131	1.31E+10	<0.001
TG 14:0_20:5_22:6	0.120	1.20E+10	<0.001
TG 20:2_22:5_22:6	0.119	1.19E+10	<0.001
TG 17:0_20:5_22:6	0.109	1.09E+10	<0.001
TG 17:1_20:4_20:5	0.108	1.08E+10	<0.001
TG 14:0_22:6_22:6	0.100	1.00E+10	<0.001
TG 20:5_22:6_22:6	0.098	9.78E+09	<0.001
Largest differences—top 10			
CE 20:5	528	13.38	<0.001
CE 22:6	168	2.65	<0.001
CE 20:4	116	1.47	<0.001
CE 18:1	96.9	1.42	<0.05
PC 16:0_20:5	42.0	6.52	<0.001
CE 16:1	35.9	3.92	<0.001
PC 18:0_20:5	17.2	3.85	<0.01
TG 16:0_16:0_18:1	16.9	2.47	<0.001
TG 16:0_18:1_22:6	16.0	5.90	<0.001
PC 16:0_22:6	15.6	1.50	<0.01

CE, cholesteryl ester; HO3, high omega-3; ILQC, intra-laboratory quality control; LO3, low omega-3; PC, phosphatidylcholine; TG, triacylglycerol.

a As determined by false discovery rate for largest fold changes using MetaboAnalyst 6.0 and by independent *t*-test for largest differences using IBM SPSS Statistics (version 30.0.0.0).

In the serum-based SRM 2378-1 (fish oil) to SRM 2378-3 (no supplements) fold comparisons, the top 10 higher in SRM 2378-1 consisted of 8 TG species, 1 OxTG, and 1 phosphatidylglycerol. The highest fold difference in SRM 2378-1 was TG 18:1_20:4_8:0, which was 25-billion-fold higher than in SRM 2378-3, but the absolute difference was only 0.255 nmol/ml (Table 3). All eight of the TG species with high fold amounts in SRM 2378-1 contained rare fatty acids of low abundance (8:0, 10:0, 17:0, 17:1, and 17:2), whereas 20:5 and/or 22:6 were observed in 4 of the top 10 higher fold lipids in SRM 2378-1. In ILQC HO3, TG 16:0_20:5_20:5 was 100-billion-fold higher than in ILQC LO3, but the absolute difference was only 1.005 nmol/ml (Table 4). The top 10 lipids with high fold differences in ILQC HO3 relative to ILQC LO3 were all TG species, with eight containing at least two 20:5 and/or 22:6 fatty acyls. The other two TG species were TG 20:2_22:5_22:6 and TG 17:1_20:4_20:5. Interestingly, TG 17:1_20:4_20:5 along with TG 17:0_20:5_22:6 were the only lipid species within the top 10 higher-fold differences for both the SRM and the ILQC comparisons.

For higher concentrations, CE 20:5 and CE 22:6 were the first and second largest differences, respectively, in both the serum SRM 2378-1 and the plasma ILQC HO3 as compared with SRM 2378-3 and ILQC LO3, respectively. The concentration of CE 20:5 in ILQC HO3 was 528 nmol/ml higher than the amount in ILQC LO3, whereas it was 217 nmol/ml higher in SRM 2378-1 as compared with SRM 2378-3. PC 16:0_20:5, PC 16:0_22:6, PC 18:0_20:5, and TG 16:0_16:0_18:1 were in the top 10 highest absolute differences in both the SRM and ILQC comparisons. PC 18:0_22:6 was in the top 10 highest absolute differences for SRM 2378-1 as compared with SRM 2378-3 but was ranked 16th in absolute differences in ILQC HO3 as compared with ILQC LO3. ILQC HO3 also had considerably higher differences in CE 20:4 (+116 nmol/ml), CE 18:1 (+97 nmol/ml), and CE 16:1 (+36 nmol/ml), reflecting the large difference in total STs between ILQC HO3 and ILQC LO3 (Table 1).

For lipids that were low in the reference materials derived from individuals consuming fish and/or fish oil, the only consistent individual lipids that were lower in SRM 2378-1 and ILQC HO3 relative to SRM 2378-3 and ILQC LO3, respectively, were lower fold levels of a plasmanyl PE (PE O-20:0/20:4) and a glucuronosyl diacylglycerol (GlcADG 18:1_18:1) (Supplemental Tables S1 and S2). The top 10 lower fold lipids in SRM 2378-1 included 3 plasmanyl and 2 plasmenyl phospholipids, a GlcADG, an OxTG, an SM, and 2 ceramides, whereas in ILQC HO3, there was a PE O, a GlcADG, 4 OxTG, a coenzyme Q, 2 dimethyl PE, and a PC. SRM 2378-1 had a lower concentration (−71.8 nmol/ml) of CE 20:4 as compared with SRM 2378-3, whereas the rest of the top 10 lower lipids had differences of >−2.5 nmol/ml. In ILQC HO3, there were five PC species with differences <−10 nmol/ml. Interestingly, eight of the top 10 lipids with lower concentrations in ILQC HO3 as compared with ILQC LO3 contained 18:2 (18:2n-6) fatty acyls, whereas the remaining two contained 20:3 (20:3n-6) and 20:4 (20:4n-6).

### Comparing materials with low omega-3 levels

Volcano plots were used to examine the differences between SRM 1950, plasma prepared using lithium heparin, SRM 2378-3 (no supplements), serum, and ILQC LO3, and plasma prepared using EDTA (Supplemental Figs. S1–3). Thirty-one lipids were significantly higher fold in SRM 1950 (plasma by heparin) as compared with SRM 2378-3 (serum) (Supplemental Figure S1), which included seven OxPC and seven OxTG, and 83 lipids were significantly lower in SRM 1950 as compared with SRM 2378-3, with 58 being TG and 14 being PC species (data not shown). Twenty-eight of the lipids that were lower in SRM 1950 and higher in SRM 2378-3 contained 20:5 and/or 22:6 fatty acyls (7 PCs, 1 PE, and 20 TGs). When SRM 1950 (plasma by heparin) was compared with ILQC LO3 (plasma by EDTA) (Supplemental Figure S2), SRM 1950 had 174 lipids that were significantly higher and 74 that were significantly lower than in ILQC LO3. Once again, SRM 1950 had higher levels of oxidized lipids (7 OxLPCs, 7 OxPCs, and 9 OxTGs) and lower levels of lipids containing 20:5 and/or 22:6 fatty acyls (6 PCs, 13 ether PCs, 5 PEs, 2 ether PSs, and 6 TGs). When SRM 2378-3 (serum) was compared with ILQC LO3 (plasma by EDTA) (Supplemental Figure S3), SRM 2378-3 had 183 lipids that were significantly higher and 36 that were significantly lower than in ILQC LO3. Most of the higher lipids in SRM 2378-3 were TG (122), and of the higher lipids, 27 contained 20:5 and/or 22:6 fatty acyls (21 TGs, 1 diacylglycerol, 2 LPCs, 1 LPI, and 1 PI).

When lipids with the largest concentration differences were examined (Supplemental Tables 3–5), SRM 1950 tended to have higher levels of TG species containing 16- and 18-carbon fatty acyls than both SRM 2378-3 (serum) and ILQC LO3 (EDTA plasma). In contrast, SRM 2378-3 tended to have higher levels of CE species than both SRM 1950 and ILQC LO3, including CE 18:3, CE 20:4, CE 20:5, and CE 22:6. SRM 2378-3 had higher LPC 18:2 than ILQC LO3 and higher LPC 16:0 and LPC 18:0 than both SRM 1950 and ILQC LO3.

## Discussion

Untargeted lipidomic profiles of commercially available plasma and serum SRMs (SRM 1950, SRM 2378-1, SRM 2378-2, and SRM 2378-3) and ILQC plasma (ILQC LO3 and ILQC HO3) were compared. SRM 2378 was included as part of the NIST interlaboratory comparison exercise for lipidomics (35), but only the SRM 1950 results have been reported (6). The SRM 2378 standard series (serum) and ILQC materials (plasma) were primarily developed to support routine fatty acid determinations across a range of n-3 PUFA intakes. The lipidomic analyses of these materials revealed that individual lipids containing EPA (20:5) and DHA (22:6) were highly variable and likely influenced by the dietary intake of EPA and DHA. The intakes of EPA and DHA of the samples used to generate each reference material were not estimated, but they can be predicted using available gas chromatography-based fatty acid compositions a posteriori (see below). While differences between plasma versus serum materials were also observed, the extent of the differences was relatively limited. In addition, we demonstrate the importance of considering differences in concentrations or abundance when comparing lipidomes. Most metabolomic approaches rely on fold-based changes, which are inherently biased toward identifying low-abundant compounds at the limits of detection, whereas compounds of high abundance are overlooked despite large absolute concentration changes.

Total lipid concentrations differed across the reference materials with SRM 2378-1 (fish oil), SRM 2378-2 (flax oil), and ILQC HO3 having approximately 30% more total lipids as compared with SRM 1950, SRM 2378-3 (no supplements), and ILQC LO3. This was largely reflected in the CE and TG fractions, which then influenced which individual lipids were identified in the top 10 different by concentration. There was, however, relative consistency across the proportions of lipid classes across the reference materials, with sterols consisting of 59–65% of the lipidome-estimated concentration, followed by glycerophospholipids (16–23%), glycerolipids (10–14%), and sphingolipids (6–8%). The consistency was also evident when examining the number of lipids identified, as 1,008 lipids (over 90% of the total lipids) were commonly identified across all six materials.

The lipidomic profile of SRM 1950 has been and continues to be determined by researchers across the globe. In 2010, the LIPID MAPS Consortium used several analytical platforms and workflows to characterize SRM 1950 based on defined lipid categories and classes and identified 588 lipid species (8). In 2017, the NIST interlaboratory harmonizing exercise involving 31 different laboratories using their own laboratory procedures identified 1,527 unique lipids (6). In the present study, 1,079 lipid species were identified in SRM 1950, which is high for a single analytical workflow as compared with single workflows in the past (6, 8). The number of identifications is also high relative to more recent reports (36, 37, 38), including a 2020 report where 451 lipids were identified by the same analyst using a similar workflow at a different location (11). The increased number of identifications that were observed across all lipid classes can largely be attributed to fast polarity switching for every sample injection, which was done in this study. This circumvented the computational challenges associated with retention-time alignment of lipids in separate positive and negative runs if there is chromatographic peak drift outside the ±0.07 min LipidMatch default search window, which would result in spectra (MS or MS/MS) being missed. Additional improvements were also due to pseudo top-40 data-dependent MS/MS as compared with pseudo top 30 in the 2020 report (11), and recent updates and continued growth of the LipidMatch database that expanded the searchable lipid library. However, the semiquantitated lipid concentrations in this report are in the lower range of those previously estimated for SRM 1950 (6), highlighting the ongoing challenge with quantitation with nontargeted lipidomic analyses.

Interlaboratory comparisons of nontargeted data will always be challenging. Differences can occur not only due to extraction protocols, implementation, and number of internal standards, type of instrumentation, and data acquisition methods but also with data analysis approaches. Different programs (i.e., LipidMatch, Compound Discoverer, ProgenesisQI, etc.) use different peak picking algorithms, which can have a considerable impact on the final peak areas. Visual inspection of each peak (manual peak picking) for all analytes and internal standards can largely account for this, but this is only feasible in targeted analyses in which only a few features (typically <100) are monitored. Batch correction-like strategies may be implemented to calibrate nontargeted data and compare across published studies, if the raw data are available. In this study, the internal standard peaks were visually inspected and integrated for every sample using a custom Thermo QuanBrowser processing method to ensure their accuracy. Peak picking and integration of all endogenous lipids were performed automatically using LipidMatch.

The consumption of EPA and DHA from seafood and fish oil supplements was reflected in the lipidomic profiles of SRM 2378-1 (fish oil) and ILQC HO3. SRM 2378-2 (flax oil) and SRM 2378-3 (no supplements) also contained higher concentrations of 20:5 and 22:6 containing lipid species than SRM 1950 and more 20:5 than ILQC LO3. The dietary intake amount of EPA and DHA appears to be important for determining lipidomic biomarkers of n-3 PUFA. Previously, PC 16:0_20:5 and PC 16:0_22:6 have been identified as possible biomarkers of n-3 PUFA intake using an interventional study design using EPA, DHA, and EPA + DHA supplements (23). Both these lipids were in the top 10 lipids with the largest concentration differences when we compared SRM 2378-1 and SRM 2378-3 and ILQC HO3 and ILQC LO3, but differences in CE 20:5 and CE 22:6 were on a larger scale (Tables 2 and 3). PC 18:0_20:5 and PC 18:0_22:6 were the two other 20:5- and/or 22:6-containing phospholipids that were consistent in demonstrating large absolute differences between materials generated from individuals with different EPA and DHA intakes. We also observed a wide dynamic range for lipids containing EPA, but we could not examine if this was proportional, as the reference standards were derived from individuals who consumed or did not consume fish oil/eat fish. A wide dynamic range for EPA in plasma as measured by gas chromatography has been documented previously as EPA fatty acid levels can get quite low in populations that do not consume seafood (<0.6% of total fatty acids) as compared with DHA, which seems to be conserved when it is not consumed (>1.5% of total fatty acids) (39). Correlations between dietary intakes of EPA and DHA and lipidomics species in whole blood have been examined in a Danish population (22). Several EPA-containing plasmenyl PE species that would be found in erythrocytes had the strongest associations with EPA + DHA intakes, but FFA 20:5, CE 20:5, and CE 22:6 that would be found in plasma also had strong associations with intake (22). There were also several PC species containing either EPA and DHA with strong associations with intake, but these could be present in both plasma and erythrocytes (22).

When fold differences between the reference materials from those consuming fish/fish oil and from those that did not were examined, unique and rare TG species were also identified. This highlights how fold difference comparisons are biased toward identifying compounds of relatively low abundance that are atypical in the food supply and our metabolism. In the SRM 2378 comparisons, several of the TG species identified contained 8:0-, 10:0-, or 17-carbon fatty acids, which were unexpected. While 8:0 and 10:0 can be found in coconut oil and odd-chain fatty acids can be found in ruminant-derived food products, particularly dairy, the present observations are likely related to the intake of fish oil supplements. Measurable amounts of 10:0 and 17:0 have been reported in various fish oils (40), and 17:1 and 17:2 have been reported in the marine algae and plankton that fish consume (41, 42, 43) when a method capable of their detection was used. The appearance of 8:0- and 10:0-containing lipids may also be associated with fish oil supplements. Short- and medium-chain fatty acid monoacylglycerols are used in aquaculture feed systems due to their antiparasitic and antimicrobial properties (44, 45), and these short-chain fatty acids can then accumulate in tissue body oils (46). Unfortunately, dietary information about these reference materials is limited to descriptions of the supplementation of 1,000 mg/d of either fish oil or flaxseed oil for 1 month for SRM 2378-1 and SRM 2378-2, respectively, without any details about the manufacturer and fatty acid composition of these oil supplements (24). In the ILQC materials, TG species containing multiple 20:5, 22:5, and/or 22:6 acyls were identified by fold-based comparisons and may reflect a much higher intake of n-3 PUFA by the individuals who provided plasma for ILQC HO3 along with less reliance on fish oil supplements as their main source of EPA and DHA. The individuals used to generate ILQC HO3 materials were recruited as they were known to consume seafood relatively regularly and use fish oil supplementation as an alternative when seafood consumption was not convenient, although their dietary habits were not formally assessed. The lack of dietary information about the population samples from which the reference materials and quality control materials were produced limits the ability to interpret these lipidomic profiles. In the future, the use of dietary assessments should be included when reference materials and quality control materials are generated. However, for n-3 PUFA status, there are publications defining the relationship between blood levels of fatty acids and dietary intakes of EPA + DHA (26, 47).

The fatty acid compositions of SRM 1950 (13) and SRM 2378 (24) have been published previously, and we routinely determine the fatty acid composition of ILQC LO3 (2.29 ± 0.05 wt%) and ILQC HO3 (8.81 ± 0.09 wt%) within our laboratory for quality control checks. From the fatty acid levels, we can estimate that EPA + DHA intake of the SRM 1950 sample was 107 mg/d. Given SRM 1950 was designed to reflect the general US population, the What We Eat in America component of the National Health and Nutrition Examination Survey can be used as a cross reference, which indicates EPA + DHA intakes in the general US population have ranged from 90 to 140 mg/d over the years (48). EPA + DHA intakes for ILQC LO3 were estimated to be 122 mg/d, 133 mg/d for SRM 2378-3 (no supplements), and 116 mg/d for SRM 2378-2 (flax oil), based on the available fatty acid composition data. The intake estimate for SRM 2378-1 (fish oil) was 337 mg/d, which is somewhat low after supplementation with 1,000 mg/d of fish oil combined with the estimated background diet intake of EPA + DHA of 116–133 mg/d from SRM 2378-2 and SRM 2378-3. While the fish oil supplement used likely had a relatively low concentration of EPA + DHA (49), it is also possible that 1 month was not long enough for blood levels of EPA and DHA in particular to plateau (47, 50), and/or the adherence to the supplementation protocol was not complete (51), which are all common challenges with fish oil intervention studies. In contrast, the EPA + DHA intake estimate for ILQC HO3 was 943 mg/d. In the future, the development of reference materials should include some form of dietary assessment of the population sample in addition to demographic information.

The comparison of SRM 1950, SRM 2378-3, and ILQC LO3 as representatives of plasma collected using heparin, serum, and plasma collected using EDTA was intended to determine lipidomic differences due to preanalytical conditions in tightly controlled comparisons (16, 25) could be observed in reference materials derived from different donors. Despite all three of these materials being taken from individuals with relatively low n-3 PUFA status, there was evidence of higher n-3 PUFA status of the SRM 2378-3 material, followed by ILQC LO3 and then SRM 1950. SRM 1950 had higher amounts of oxidized lipids (OxPC and OxTG) than SRM 2378-3 and ILQC LO3. While EDTA tends to result in higher detectable amounts of PC, SM, and TG in plasma as compared with heparin due to enhanced extraction recovery and matrix effects (52), the differences in these lipids and CE species in the reference materials we examined are likely more indicative of differences in the amounts of circulating lipoproteins between the donors of SRM 1950, SRM 2378-3, and ILQC LO3. While the lipoprotein profiles of the donors are not known, age is strongly associated with increased lipoprotein TG and CE (53). The SRM 1950 donors ranged between 40 and 50 years of age, and the ILQC LO3 donors were in their low 20s. The age of the donors for SRM 2378-3 was not reported (24). The increased oxidized lipids in SRM 1950 could be a storage effect (16), as it was created in 2006 (13), as compared with SRM 2378-3 in 2012 (24) and ILQC LO3 in 2015. However, the oxidized lipids in SRM 1950 may have also been generated during sample collection and preparation (16) as SRM 1950 required the processing of blood samples from 100 individuals, as compared with only three for SRM 2378-3 and two for ILQC LO3. Elevated lysophospholipids can not only be an indicator of extended sample processing in general but also of coagulation during serum collection (16). SRM 1950 (heparin plasma from 2006) had higher amounts of LPC 18:0 as compared with ILQC LO3 (EDTA plasma from 2015) but lower amounts of LPC 16:0 and LPC 18:2 of the SRM 2378-3 (serum from 2012). The present results not only confirm previous observations about the effects of preanalytics on lipidomic profiles but also suggest that different sample preparations can be used for untargeted global analyses and generate useful and comparable lipidomic profiles of highly abundant and less metabolically active lipids.

In conclusion, this is the first publication reporting the global lipidomic profiles of the SRM 2378 sample series to our knowledge, and we have included the lipidomic profiling of SRM 1950 and IQLC materials used for fatty acid profiling of plasma. EPA- and DHA-containing lipids were pronounced in the reference materials generated from donors after fish oil supplementation (SRM 2378-1) and donors who ate seafood/used supplements regularly (ILQC HO3), but differences were also observed in the reference materials created from donors not using supplements and consuming typical North American diets low in seafood. Fatty acid isomers can be a challenge in interpreting untargeted lipidomic analyses, but herein, we demonstrated the value of using highly quantitative fatty acid composition analyses by gas chromatography to support the interpretation of lipidomic data. Given the value of using reference materials with lipidomic profiles relevant to subpopulations of a research interest (11, 15), demographic information about the donors needs to be collected. The impact of n-3 PUFA status on the lipidome indicates that collecting dietary intake information at the time of donation for reference materials and research subpopulations should be considered, as this information cannot be gathered ad hoc. Dietary intakes of EPA and DHA can correlate with measures of 20:5n-3 and 22:6n-3 in blood by gas chromatography (47, 54) and with some lipidomic species (22). However, the strength of the relationship between blood biomarkers and nutrient intakes can be dependent on the type of dietary assessment tool due to the sporadic nature of seafood intake, particularly in Westernized societies (22, 51, 55). While NIST has recently developed the RM 8231 series of reference materials to consider ethnicity, disease, and/or metabolic status, the dietary intakes of the donors that provide the plasma used to create these materials are not documented (15). For SRM 2378, the dosage of fish oil and flaxseed oil that the donors were provided is indicated, but the fatty acid concentrations of these supplements, and adherence to the supplementation, a common problem (56), are not documented, and there is no information on the background diet of the participants. Our own laboratory failed to complete dietary assessments on the donors who provided the plasma that we used to create our ILQC LO3 and ILQC HO3 materials in 2015. Moving forward, dietary assessments should be completed on donors when reference materials are developed, and lipidomic analysts, particularly those engaging in nontargeted approaches, need to consider using gas chromatographic analyses to better establish the acyl composition of the lipidome. These efforts are needed to understand the impact of diet on the lipidome and to continue to improve the ability to interpret lipidomic profiles.

## Data availability

The datasets collected and analyzed in the present study are available from the corresponding author on reasonable request. A table of the normalized semiquantitative concentrations for all lipids identified in this study will be provided in the supplemental tables.

## Supplemental data

This article contains supplemental data.

## Conflict of interests

K. D. S. is currently on the Board of Directors of the International Society for the Study of Fatty Acids and Lipids. J. J. A.-H. generated the intralaboratory materials while completing his PhD at the University of Waterloo and completed the lipidomic analyses while working for BPGbio, Inc, under the supervision of M. K. K. E. S. processed data while completing MSc at the University of Waterloo and now works for Lipotype GmbH. Both BPGbio and Lipotype are companies that provide lipidomic analytical services. All other authors declare that they have no conflicts of interest with the contents of this article.

## Supplementary Data

Supplementary Table 6

Supplementary Figures s
