# Supplementary material for: The n-3 PUFA content of the global lipidomes of NIST SRM 2378, SRM 1950, and intralaboratory quality control materials
Source: J Lipid Res. 2025 Dec 26;67(2):100970. doi: 10.1016/j.jlr.2025.100970 (PMC12861023; doi:10.1016/j.jlr.2025.100970)
Supplement: Supplementary Tables 1-5 [file mmc1.docx]

**Supplementary Table 1.** Lipids Lower in SRM 2378-1 relative to SRM 2378-3

| Largest fold changes – Top 10 | Difference (nmol/mL) | Fold diff | P-value* |
| --- | --- | --- | --- |
| PC O-16:1/14:0 | -0.0474 | 2.11E-10 | <0.001 |
| PE O-20:0/20:4 | -0.0087 | 1.15E-09 | <0.001 |
| PE P-18:1/24:1 | -0.0078 | 1.29E-09 | <0.001 |
| GlcADG 18:1_18:1 | -0.0019 | 5.15E-09 | <0.001 |
| OxTG 18:0_18:2_18:2(OH) | -0.0007 | 1.5E-08 | <0.001 |
| PE P-20:0/20:2 | -0.0311 | 0.27322 | <0.01 |
| SM d17:1/18:0 | -0.9753 | 0.38396 | <0.01 |
| PE O-16:0/22:4 | -0.0229 | 0.39264 | <0.01 |
| Cer-NP t18:1/23:1 | -0.0026 | 0.43956 | <0.01 |
| Cer-NS d18:1/24:2 | -0.0163 | 0.49328 | <0.01 |
| Largest differences – Top 10 |  |  |  |
| CE 20:4 | -71.8 | 0.79 | <0.05 |
| CE 22:5 | -2.49 | 0.62 | <0.01 |
| PE P-16:0/20:4 | -1.05 | 0.67 | <0.05 |
| SM d17:1/18:0 | -0.98 | 0.38 | <0.001 |
| PC 18:1_20:3 | -0.75 | 0.75 | <0.01 |
| LPC 20:4 | -0.74 | 0.83 | <0.01 |
| PC O-18:0/20:4 | -0.69 | 0.76 | <0.05 |
| PC 18:0_22:4 | -0.57 | 0.71 | <0.001 |
| PC P-16:0/20:4 | -0.53 | 0.88 | <0.001 |
| TG 16:0_18:1_22:4 | -0.37 | 0.80 | <0.05 |

*As determined by FDR for largest fold changes using MetaboAnalyst 6.0 and by
independent t-test for largest differences using IBM SPSS Statistics (Version 30.0.0.0). SRM, National Institutes of Standards and Technology Standard Reference Material; PC O, plasmanyl phosphatidylcholine; PE O, plasmanyl phosphatidylethanolamine; PE P, plasmenyl phosphatidylethanolamine; GlcADG, glucuronosyl diacylglycerol; OxTG, oxidized triacylglycerol; SM, sphingomyelin; Cer-NP. non-hydroxy phytosphingosine ceramide; Cer-NS, non-hydroxy sphingosine ceramide; CE, cholesteryl ester; LPC, lysophosphatidylcholine; TG, triacylglycerol.

**Supplementary Table 2.** Lipids Lower in ILQC HO3 relative to ILQC LO3

| Largest fold changes – Top 10 | Difference (nmol/mL) | Fold diff | P-value* |
| --- | --- | --- | --- |
| PE O-20:0/20:4 | -0.0506 | 1.98E-10 | <0.001 |
| OxTG 16:0_18:2_18:1(Ke_or_Epoxy) | -0.0328 | 3.05E-10 | <0.001 |
| Co Q9 | -0.0238 | 4.19E-10 | <0.001 |
| OxTG 18:1_18:1_18:1(Ke_or_Epoxy) | -0.0169 | 5.93E-10 | <0.001 |
| OxTG 18:1_18:2_18:1(Ke_or_Epoxy) | -0.0122 | 8.17E-10 | <0.001 |
| DMPE 18:0_22:5 | -0.0118 | 8.49E-10 | <0.001 |
| OxTG 18:1_18:1_18:2(OH) | -0.0108 | 9.27E-10 | <0.001 |
| GlcADG 18:1_18:1 | -0.0042 | 2.39E-09 | <0.001 |
| DMPE 16:0_22:6 | -0.0041 | 2.45E-09 | <0.001 |
| PC 15:1_16:0 | -0.0019 | 5.15E-09 | <0.001 |
| Largest differences – Top 10 |  |  |  |
| PC 16:0_18:2 | -42.0 | 0.68 | <0.05 |
| PC 18:0_18:2 | -32.7 | 0.67 | <0.05 |
| PC 16:0_20:3 | -20.7 | 0.59 | <0.001 |
| PC 18:1_18:2 | -11.5 | 0.47 | <0.001 |
| PC 18:2_18:2 | -11.0 | 0.37 | <0.001 |
| TG 18:1_18:1_18:2 | -7.97 | 0.68 | <0.001 |
| TG 16:0_18:2_18:2 | -6.42 | 0.82 | <0.05 |
| TG 18:1_18:2_18:2 | -4.48 | 0.77 | <0.01 |
| PE P-18:0/20:4 | -3.50 | 0.49 | <0.01 |
| LPC 18:2 | -3.31 | 0.71 | <0.01 |

*As determined by FDR for largest fold changes using MetaboAnalyst 6.0 and by
independent t-test for largest differences using IBM SPSS Statistics (Version 30.0.0.0). ILQC, Intra-laboratory quality control; HO3, high omega-3; LO3, low omega-3; PE O, plasmanyl phosphatidylethanolamine; OxTG, oxidized triacylglycerol; CoQ, coenzyme quinone; DMPE, dimethyl phosphatidylethanolamine; GlcADG, glucuronosyl diacylglycerol; PC, phosphatidylcholine; PE P, plasmenyl phosphatidylethanolamine; LPC, lysophosphatidylcholine.

**Supplementary Table 3.** Lipids with the concentration differences between SRM 1950 (heparin plasma) and SRM 2378-3 (serum)

| Top 10 lipids higher in SRM 1950 | Difference (nmol/mL) | Fold diff | P-value* |
| --- | --- | --- | --- |
| TG 16:0_18:1_18:2 | 14.27 | 1.26 | <0.01 |
| TG 16:0_18:2_18:2 | 13.41 | 1.39 | <0.001 |
| TG 16:0_18:1_18:1 | 11.83 | 1.22 | <0.05 |
| CE 16:1 | 8.21 | 1.44 | <0.01 |
| TG 16:0_16:1_18:1 | 8.03 | 1.33 | <0.001 |
| PC 16:0_20:3 | 7.20 | 1.20 | <0.05 |
| TG 14:0_18:1_18:2 | 6.36 | 1.40 | <0.001 |
| SM d18:1/16:0 | 5.07 | 1.10 | <0.01 |
| SM d18:2/24:1 | 4.79 | 1.26 | <0.01 |
| TG 16:0_18:2_18:3 | 4.18 | 1.56 | <0.001 |
| Top 10 lipids higher in SRM 2378-3 |  |  |  |
| CE 20:4 | -76.28 | 0.78 | <0.01 |
| CE 22:6 | -65.31 | 0.51 | <0.01 |
| CE 20:5 | -36.49 | 0.54 | <0.001 |
| CE 18:3 | -27.65 | 0.85 | <0.01 |
| CE 16:0 | -14.57 | 0.46 | <0.001 |
| LPC 16:0 | -9.85 | 0.83 | <0.01 |
| PC 16:0_22:6 | -9.05 | 0.69 | <0.001 |
| PC 18:2_18:2 | -5.63 | 0.62 | <0.001 |
| LPC 18:2 | -5.43 | 0.73 | <0.001 |
| CE 20:2 | -4.36 | 0.52 | <0.001 |

*As determined independent t-test for largest differences using IBM SPSS Statistics Version 30.0.0.0). SRM, National Institutes of Standards and Technology Standard Reference Material; TG, triacylglycerol; CE, cholesteryl ester; PC, phosphatidylcholine; SM, sphingomyelin; LPC, lysophosphatidylcholine.

**Supplementary Table 4.** Lipids with the concentration differences between SRM 1950 (heparin plasma) and ILQC LO3 (EDTA plasma)

| Top 10 lipids higher in SRM 1950 | Difference (nmol/mL) | Fold diff | P-value* |
| --- | --- | --- | --- |
| CE 18:1 | 52.58 | 1.23 | <0.05 |
| LPC 16:0 | 23.79 | 1.96 | <0.001 |
| CE 18:3 | 23.71 | 1.19 | <0.05 |
| CE 16:1 | 14.49 | 2.18 | <0.001 |
| LPC 18:0 | 13.74 | 2.61 | <0.001 |
| TG 16:0_18:1_18:1 | 13.19 | 1.25 | <0.05 |
| TG 16:0_16:1_18:1 | 12.83 | 1.65 | <0.001 |
| TG 16:0_18:1_18:2 | 12.82 | 1.23 | <0.05 |
| TG 16:0_18:2_18:2 | 12.71 | 1.36 | <0.01 |
| TG 16:0_16:0_18:1 | 10.42 | 1.91 | <0.001 |
| Top 10 lipids higher in ILQC LO3 |  |  |  |
| CE 22:6 | -32.72 | 0.68 | <0.001 |
| CE 16:0 | -11.80 | 0.51 | <0.001 |
| PC 16:0_22:6 | -11.18 | 0.64 | <0.001 |
| SM d18:1/24:1 | -9.53 | 0.74 | <0.05 |
| PC 18:2_18:2 | -8.28 | 0.53 | <0.001 |
| PC 18:1_18:2 | -8.18 | 0.62 | <0.001 |
| SM d17:1/24:1 | -3.57 | 0.46 | <0.01 |
| PE 16:0_22:6 | -2.31 | 0.27 | <0.001 |
| PC 16:0_22:5 | -2.15 | 0.84 | <0.05 |
| PC P-16:0/18:2 | -2.12 | 0.63 | <0.001 |

*As determined independent t-test for largest differences using IBM SPSS Statistics Version 30.0.0.0). SRM, National Institutes of Standards and Technology Standard Reference Material; ILQC, Intra-laboratory quality control; LO3, low omega-3; CE, cholesteryl ester; LPC, lysophosphatidylcholine; TG, triacylglycerol; PC, phosphatidylcholine; SM, sphingomyelin; PE, phosphatidylethanolamine; PC P, plasmenyl phosphatidylcholine.

**Supplementary Table 5.** Lipids with the concentration differences between SRM 2378-3 (serum) and ILQC LO3 (EDTA plasma)

| Top 10 lipids higher in SRM 2378-3 | Difference (nmol/mL) | Fold diff | P-value* |
| --- | --- | --- | --- |
| CE 20:4 | 93.69 | 1.38 | <0.01 |
| CE 18:3 | 51.36 | 1.40 | <0.01 |
| CE 20:5 | 35.80 | 1.84 | <0.001 |
| LPC 16:0 | 33.64 | 2.35 | <0.001 |
| CE 22:6 | 32.59 | 1.32 | <0.05 |
| LPC 18:0 | 17.92 | 3.11 | <0.001 |
| LPC 18:2 | 8.58 | 1.75 | <0.001 |
| TG 16:0_18:0_18:1 | 8.06 | 2.27 | <0.001 |
| LPC 18:1 | 7.26 | 1.99 | <0.001 |
| TG 16:0_16:0_18:1 | 6.84 | 1.59 | <0.01 |
| Top 10 lipids higher in ILQC LO3 |  |  |  |
| PC 16:0_20:3 | -13.71 | 0.73 | <0.01 |
| SM d18:1/24:1 | -11.45 | 0.69 | <0.05 |
| PC 18:1_18:2 | -6.19 | 0.71 | <0.001 |
| SM d18:2/24:1 | -6.08 | 0.75 | <0.001 |
| SM d18:1/16:0 | -3.95 | 0.93 | <0.05 |
| TG 18:1_18:2_18:2 | -3.89 | 0.80 | <0.001 |
| TG 18:1_18:1_18:2 | -3.30 | 0.87 | <0.05 |
| SM d17:1/24:1 | -3.22 | 0.51 | <0.05 |
| PC 18:2_18:2 | -2.65 | 0.85 | <0.01 |
| PC 16:0_22:5 | -2.32 | 0.83 | <0.05 |

*As determined independent t-test for largest differences using IBM SPSS Statistics Version 30.0.0.0). SRM, National Institutes of Standards and Technology Standard Reference Material; ILQC, Intra-laboratory quality control; LO3, low omega-3; CE, cholesteryl ester; LPC, lysophosphatidylcholine; TG, triacylglycerol; PC, phosphatidylcholine.
