# Supplementary material for: The n-3 PUFA content of the global lipidomes of NIST SRM 2378, SRM 1950, and intralaboratory quality control materials
Source: J Lipid Res. 2025 Dec 26;67(2):100970. doi: 10.1016/j.jlr.2025.100970 (PMC12861023; doi:10.1016/j.jlr.2025.100970)
Supplement: Supplementary Table 6 [file mmc2.docx]

**Supplementary Table 6**. Semi-quantitative concentrations of all identified lipid species

| **Lipid** | **m/z**  **max intensity** | **Adducts**  **Confirmed by MS/MS** | **RT min** | **SRM 1950** | **SRM2378-3**  **No supplement** | **SRM2378-2**  **Flax oil** | **SRM2378-1**  **Fish oil** | **ILQC LO3** | **ILQC HO3** |
| --- | --- | --- | --- | --- | --- | --- | --- | --- | --- |
|  |  |  |  | ***Concentration (nmol/mL)*** | | | | | |
| ***Glycerolipids*** |  |  |  |  |  |  |  |  |  |
| DG 12:0_18:1 | 556.4943 | [M+NH4]+ | 13.89 | 0.022 ± 0.001 | 0.040 ± 0.011 | 0.021 ± 0.002 | 0.057 ± 0.017 | <0.001 | 0.014 ± 0.002 |
| DG 14:0_16:0 | 558.5100 | [M+NH4]+ | 15.20 | 0.079 ± 0.005 | 0.10 ± 0.02 | 0.076 ± 0.003 | 0.23 ± 0.03 | 0.011 ± <0.001 | 0.063 ± 0.007 |
| DG 14:0_18:2 | 582.5098 | [M+NH4]+ | 14.23 | 0.087 ± 0.008 | 0.14 ± 0.05 | 0.15 ± 0.02 | 0.21 ± 0.04 | 0.11 ± 0.06 | 0.12 ± 0.06 |
| DG 15:0_18:1 | 598.5417 | [M+NH4]+ | 15.82 | 0.043 ± 0.002 | 0.046 ± 0.008 | 0.065 ± 0.003 | 0.057 ± 0.002 | 0.016 ± 0.002 | 0.032 ± 0.002 |
| DG 16:0_16:0 | 586.5416 | [M+NH4]+ | 16.30 | 0.37 ± 0.004 | 0.34 ± 0.01 | 0.37 ± 0.003 | 0.73 ± 0.02 | 0.13 ± 0.002 | 0.33 ± 0.002 |
| DG 16:0_16:1 | 584.5256 | [M+NH4]+ | 15.26 | 0.41 ± 0.02 | 0.36 ± 0.03 | 0.44 ± 0.01 | 0.53 ± 0.06 | 0.11 ± 0.003 | 0.29 ± 0.01 |
| DG 16:0_18:0 | 614.5723 | [M+NH4]+ | 17.17 | 0.54 ± 0.01 | 0.41 ± 0.02 | 0.58 ± 0.01 | 0.49 ± 0.01 | 0.34 ± 0.006 | 0.38 ± 0.001 |
| DG 16:0_18:1 | 612.5562 | [M+NH4]+ | 16.34 | 2.09 ± 0.02 | 1.93 ± 0.06 | 2.81 ± 0.07 | 2.29 ± 0.18 | 0.99 ± 0.03 | 1.19 ± 0.01 |
| DG 16:0_18:2 | 610.5407 | [M+NH4]+ | 15.47 | 1.74 ± 0.07 | 1.43 ± 0.11 | 2.43 ± 0.09 | 1.86 ± 0.08 | 0.94 ± 0.08 | 1.06 ± 0.11 |
| DG 16:0_18:3 | 608.5256 | [M+NH4]+ | 14.52 | 0.21 ± 0.01 | 0.15 ± 0.003 | 0.31 ± 0.01 | 0.27 ± 0.01 | 0.074 ± 0.001 | 0.086 ± 0.006 |
| DG 16:0_19:0 | 628.5890 | [M+NH4]+ | 16.92 | 0.001 ± <0.001 | 0.005 ± 0.001 | <0.001 | 0.002 ± 0.001 | <0.001 | 0.002 ± <0.001 |
| DG 16:0_20:4 | 634.5411 | [M+NH4]+ | 15.14 | 0.14 ± 0.01 | 0.11 ± 0.006 | 0.14 ± <0.001 | 0.20 ± 0.01 | 0.045 ± 0.004 | 0.14 ± 0.005 |
| DG 16:0_22:6 | 658.5411 | [M+NH4]+ | 14.71 | 0.016 ± 0.002 | 0.026 ± 0.001 | 0.042 ± 0.002 | 0.11 ± 0.003 | 0.010 ± 0.001 | 0.19 ± 0.006 |
| DG 16:1_18:1 | 610.5411 | [M+NH4]+ | 15.18 | 0.003 ± 0.001 | 0.005 ± 0.008 | 0.003 ± 0.001 | 0.008 ± 0.006 | 0.006 ± 0.005 | 0.009 ± 0.007 |
| DG 16:1_18:2 | 608.5258 | [M+NH4]+ | 14.27 | 0.11 ± 0.01 | 0.044 ± 0.024 | 0.15 ± 0.004 | 0.076 ± 0.022 | 0.034 ± 0.016 | 0.069 ± 0.014 |
| DG 16:1_20:4 | 632.5249 | [M+NH4]+ | 13.82 | 0.057 ± 0.011 | 0.027 ± 0.008 | 0.070 ± 0.003 | 0.056 ± 0.013 | 0.006 ± 0.002 | 0.049 ± 0.007 |
| DG 17:0_18:0 | 628.5885 | [M+NH4]+ | 17.59 | 0.010 ± 0.001 | 0.004 ± 0.003 | 0.010 ± 0.004 | 0.007 ± 0.003 | 0.002 ± 0.002 | 0.007 ± 0.004 |
| DG 17:0_18:1 | 626.5731 | [M+NH4]+ | 16.77 | 0.046 ± 0.006 | 0.069 ± 0.003 | 0.079 ± 0.002 | 0.065 ± 0.004 | 0.028 ± 0.003 | 0.034 ± 0.001 |
| DG 17:1_18:1 | 624.5562 | [M+NH4]+ | 15.86 | 0.055 ± 0.006 | 0.068 ± 0.003 | 0.11 ± 0.003 | 0.069 ± 0.001 | 0.049 ± 0.001 | 0.047 ± 0.002 |
| DG 18:0_18:0 | 642.6036 | [M+NH4]+ | 18.00 | 1.25 ± 0.02 | 0.77 ± 0.04 | 1.48 ± 0.05 | 0.57 ± 0.01 | 1.10 ± 0.03 | 0.90 ± 0.02 |
| DG 18:0_18:1 | 640.5887 | [M+NH4]+ | 17.22 | 0.42 ± 0.003 | 0.47 ± 0.01 | 0.49 ± 0.01 | 0.52 ± 0.01 | 0.19 ± 0.005 | 0.28 ± 0.005 |
| DG 18:0_18:2 | 638.5720 | [M+NH4]+ | 30.20 | 0.001 ± <0.001 | 0.002 ± 0.002 | 0.003 ± 0.002 | 0.003 ± 0.001 | 0.003 ± 0.002 | 0.002 ± 0.001 |
| DG 18:0_20:1 | 668.6205 | [M+NH4]+ | 18.02 | 0.023 ± 0.001 | 0.037 ± 0.003 | 0.041 ± 0.004 | 0.035 ± 0.004 | 0.016 ± 0.002 | 0.026 ± 0.002 |
| DG 18:0_20:4 | 662.5726 | [M+NH4]+ | 16.23 | 0.032 ± 0.005 | 0.049 ± 0.004 | 0.051 ± 0.002 | 0.038 ± 0.002 | 0.017 ± 0.004 | 0.024 ± 0.003 |
| DG 18:1_18:1 | 638.5724 | [M+NH4]+ | 16.38 | 2.37 ± 0.20 | 2.44 ± 0.03 | 4.04 ± 0.09 | 2.30 ± 0.07 | 2.49 ± 0.07 | 1.61 ± 0.03 |
| DG 18:1_19:0 | 654.6024 | [M+NH4]+ | 17.49 | <0.001 | 0.001 ± <0.001 | 0.001 ± 0.001 | <0.001 | <0.001 | <0.001 |
| DG 18:1_20:1 | 666.6037 | [M+NH4]+ | 17.42 | 0.68 ± 0.02 | 0.87 ± 0.15 | 1.16 ± 0.11 | 0.88 ± 0.06 | 0.64 ± 0.13 | 0.50 ± 0.42 |
| DG 18:1_20:2 | 664.5879 | [M+NH4]+ | 16.65 | 0.24 ± 0.001 | 0.28 ± 0.04 | 0.41 ± 0.03 | 0.20 ± 0.13 | 0.12 ± 0.11 | 0.16 ± 0.11 |
| DG 18:1_20:3 | 662.5724 | [M+NH4]+ | 16.06 | 0.056 ± 0.006 | 0.051 ± 0.007 | 0.067 ± 0.003 | 0.039 ± 0.004 | 0.044 ± 0.001 | 0.045 ± 0.003 |
| DG 18:1_20:4 | 660.5568 | [M+NH4]+ | 15.20 | 0.29 ± 0.03 | 0.27 ± 0.01 | 0.43 ± 0.005 | 0.37 ± 0.01 | 0.19 ± 0.003 | 0.35 ± 0.01 |
| DG 18:1_22:1 | 694.6350 | [M+NH4]+ | 18.31 | 0.66 ± 0.06 | 1.40 ± 0.69 | 1.29 ± 0.26 | 1.47 ± 0.46 | 1.10 ± 0.43 | 1.19 ± 0.50 |
| DG 18:1_22:4 | 688.5881 | [M+NH4]+ | 16.06 | 0.070 ± 0.006 | 0.038 ± 0.001 | 0.056 ± 0.003 | 0.032 ± 0.004 | 0.028 ± <0.001 | 0.047 ± 0.001 |
| DG 18:1_22:5 | 686.5726 | [M+NH4]+ | 15.24 | 0.017 ± 0.003 | 0.016 ± 0.005 | 0.054 ± 0.001 | 0.031 ± 0.004 | 0.017 ± 0.004 | 0.093 ± 0.011 |
| DG 18:1_22:6 | 684.5569 | [M+NH4]+ | 14.79 | 0.042 ± 0.003 | 0.058 ± 0.003 | 0.12 ± 0.003 | 0.16 ± 0.01 | 0.075 ± 0.003 | 0.45 ± 0.01 |
| DG 18:1_24:1 | 722.6667 | [M+NH4]+ | 18.85 | 0.003 ± <0.001 | 0.003 ± 0.001 | 0.004 ± <0.001 | 0.002 ± <0.001 | 0.003 ± <0.001 | 0.002 ± <0.001 |
| DG 18:2_18:2 | 634.5410 | [M+NH4]+ | 17.33 | <0.001 | <0.001 | <0.001 | <0.001 | <0.001 | <0.001 |
| DG 18:2_18:3 | 632.5256 | [M+NH4]+ | 13.64 | 0.069 ± 0.011 | 0.038 ± 0.002 | 0.11 ± 0.005 | 0.054 ± 0.001 | 0.015 ± 0.004 | 0.029 ± 0.012 |
| DG 18:2_20:4 | 658.5417 | [M+NH4]+ | 14.15 | 0.12 ± 0.03 | 0.11 ± 0.01 | 0.20 ± 0.004 | 0.23 ± 0.03 | 0.065 ± 0.008 | 0.28 ± 0.10 |
| DG 18:2_20:5 | 656.5265 | [M+NH4]+ | 13.00 | 0.011 ± 0.004 | 0.016 ± 0.002 | 0.050 ± 0.006 | 0.17 ± 0.04 | 0.002 ± 0.001 | 0.22 ± 0.01 |
| DG 18:2_22:6 | 682.5408 | [M+NH4]+ | 13.64 | 0.038 ± 0.005 | 0.036 ± 0.002 | 0.11 ± 0.002 | 0.099 ± 0.008 | 0.049 ± 0.002 | 0.26 ± 0.006 |
| GlcADG 16:0_18:1 | 788.5912 | [M+NH4]+ | 11.33 | 0.022 ± 0.008 | 0.023 ± 0.007 | 0.051 ± 0.007 | 0.012 ± 0.009 | 0.013 ± 0.005 | 0.004 ± 0.003 |
| GlcADG 18:1_18:1 | 814.6069 | [M+NH4]+ | 11.44 | 0.005 ± 0.007 | 0.002 ± 0.001 | 0.002 ± 0.001 | <0.001 | 0.004 ± 0.003 | <0.001 |
| GlcADG 20:4_22:6 | 863.5307 | [M-H]- | 13.83 | <0.001 | <0.001 | <0.001 | <0.001 | <0.001 | <0.001 |
| MGDG 17:0_18:1 | 815.5903 | [M+HCO2]- | 13.42 | 0.18 ± 0.02 | 0.17 ± 0.01 | 0.20 ± 0.01 | 0.14 ± 0.02 | 0.25 ± 0.05 | 0.19 ± 0.04 |
| MGDG 18:0_18:1 | 829.6027 | [M+HCO2]- | 15.28 | <0.001 | <0.001 | <0.001 | <0.001 | <0.001 | <0.001 |
| MGDG 18:0_20:0 | 859.6520 | [M+HCO2]- | 15.72 | 0.001 ± 0.001 | 0.001 ± 0.002 | 0.003 ± 0.003 | 0.003 ± 0.001 | 0.003 ± 0.003 | 0.003 ± 0.002 |
| MGDG 18:1_19:0 | 843.6231 | [M+HCO2]- | 14.86 | 0.040 ± 0.027 | 0.025 ± 0.023 | 0.001 ± 0.001 | 0.003 ± 0.001 | 0.024 ± 0.022 | 0.008 ± 0.007 |
| SQDG 16:0_16:0 | 812.5568 | [M+NH4]+ | 12.18 | 0.002 ± 0.001 | 0.002 ± 0.001 | 0.002 ± 0.002 | 0.021 ± <0.001 | 0.004 ± 0.003 | 0.017 ± 0.008 |
| TG O-16:0_16:0_16:1 | 808.7754 | [M+NH4]+ | 23.35 | 0.012 ± <0.001 | 0.019 ± 0.007 | 0.015 ± 0.001 | 0.015 ± 0.005 | 0.005 ± 0.002 | 0.008 ± 0.003 |
| TG O-16:0_16:0_18:0 | 838.8231 | [M+NH4]+ | 25.51 | 0.011 ± 0.001 | 0.023 ± 0.004 | 0.014 ± 0.003 | 0.012 ± 0.001 | 0.004 ± <0.001 | 0.006 ± 0.001 |
| TG O-16:0_16:0_18:1 | 836.8068 | [M+NH4]+ | 24.39 | 0.11 ± 0.01 | 0.16 ± 0.02 | 0.14 ± 0.01 | 0.12 ± 0.01 | 0.097 ± 0.015 | 0.092 ± 0.012 |
| TG O-16:0_16:0_18:2 | 834.7915 | [M+NH4]+ | 23.45 | 0.041 ± 0.002 | 0.046 ± 0.015 | 0.046 ± 0.006 | 0.033 ± 0.006 | 0.023 ± 0.008 | 0.019 ± 0.005 |
| TG O-16:0_16:0_20:4 | 858.7913 | [M+NH4]+ | 23.10 | 0.003 ± 0.001 | 0.010 ± 0.007 | 0.006 ± 0.001 | 0.007 ± 0.002 | 0.007 ± 0.001 | 0.001 ± <0.001 |
| TG O-16:0_18:1_20:4 | 884.8081 | [M+NH4]+ | 23.10 | <0.001 | 0.002 ± 0.003 | 0.001 ± <0.001 | <0.001 | 0.007 ± 0.001 | 0.001 ± 0.001 |
| TG O-18:0_14:0_16:0 | 810.7913 | [M+NH4]+ | 24.45 | 0.015 ± 0.002 | 0.027 ± 0.011 | 0.015 ± 0.002 | 0.015 ± 0.005 | 0.006 ± 0.004 | 0.008 ± 0.005 |
| TG O-18:0_16:0_18:0 | 866.8532 | [M+NH4]+ | 26.55 | 0.001 ± <0.001 | 0.003 ± 0.001 | 0.001 ± <0.001 | <0.001 | <0.001 | <0.001 |
| TG O-18:0_16:0_18:1 | 864.8377 | [M+NH4]+ | 25.44 | 0.039 ± 0.002 | 0.063 ± 0.012 | 0.053 ± 0.005 | 0.044 ± 0.008 | 0.023 ± 0.003 | 0.022 ± 0.003 |
| TG O-18:0_18:1_18:2 | 888.8390 | [M+NH4]+ | 24.33 | 0.030 ± 0.001 | 0.032 ± 0.002 | 0.045 ± 0.001 | 0.020 ± 0.004 | 0.024 ± 0.006 | 0.012 ± 0.003 |
| TG O-18:0_18:1_22:6 | 936.8390 | [M+NH4]+ | 23.60 | <0.001 | 0.001 ± 0.001 | <0.001 | 0.002 ± 0.001 | 0.001 ± 0.001 | <0.001 |
| TG O-18:1_16:0_18:1 | 862.8230 | [M+NH4]+ | 24.33 | 0.12 ± 0.01 | 0.17 ± 0.01 | 0.18 ± 0.004 | 0.14 ± 0.01 | 0.11 ± 0.01 | 0.092 ± 0.008 |
| TG O-18:1_16:0_18:2 | 860.8073 | [M+NH4]+ | 23.39 | 0.062 ± 0.004 | 0.075 ± 0.010 | 0.082 ± 0.005 | 0.039 ± 0.009 | 0.051 ± 0.004 | 0.007 ± 0.005 |
| TG O-18:1_16:0_24:0 | 948.9327 | [M+NH4]+ | 28.41 | 0.005 ± 0.002 | 0.006 ± 0.002 | 0.007 ± 0.001 | 0.006 ± 0.002 | 0.002 ± 0.001 | 0.007 ± 0.002 |
| TG O-18:1_16:0_24:1 | 946.9161 | [M+NH4]+ | 27.38 | 0.008 ± 0.002 | 0.007 ± 0.002 | 0.016 ± 0.002 | 0.009 ± 0.003 | 0.004 ± 0.001 | 0.010 ± 0.003 |
| TG O-20:0_16:0_18:1 | 892.8684 | [M+NH4]+ | 26.48 | 0.010 ± <0.001 | 0.014 ± 0.003 | 0.012 ± 0.001 | 0.010 ± 0.001 | 0.003 ± 0.001 | 0.007 ± 0.002 |
| TG O-20:0_18:0_18:1 | 920.9013 | [M+NH4]+ | 27.50 | 0.002 ± <0.001 | 0.004 ± 0.001 | 0.003 ± <0.001 | 0.001 ± 0.001 | 0.001 ± 0.001 | 0.004 ± 0.001 |
| TG O-20:0_18:1_18:2 | 916.8693 | [M+NH4]+ | 25.41 | <0.001 | <0.001 | <0.001 | <0.001 | <0.001 | <0.001 |
| TG O-20:1_16:0_18:1 | 890.8539 | [M+NH4]+ | 25.39 | 0.018 ± 0.003 | 0.027 ± 0.003 | 0.025 ± 0.002 | 0.017 ± 0.004 | 0.010 ± 0.001 | 0.010 ± 0.002 |
| TG O-20:1_18:0_18:1 | 918.8844 | [M+NH4]+ | 26.37 | <0.001 | <0.001 | <0.001 | <0.001 | <0.001 | 0.001 ± 0.002 |
| OxTG 14:0_16:0_18:1 (Ke_or_Epoxy) | 836.7343 | [M+NH4]+ | 18.14 | 0.006 ± 0.001 | 0.004 ± 0.001 | 0.002 ± <0.001 | 0.021 ± 0.002 | 0.001 ± <0.001 | 0.024 ± 0.003 |
| OxTG 14:0_16:0_20:1(Ke_or_Epoxy) | 864.7659 | [M+NH4]+ | 18.74 | 0.003 ± 0.002 | 0.001 ± 0.001 | <0.001 | 0.001 ± 0.002 | <0.001 | 0.002 ± 0.002 |
| OxTG 16:0_16:0_18:1(Ke_or_Epoxy) | 864.7657 | [M+NH4]+ | 18.98 | 0.034 ± 0.005 | 0.013 ± 0.003 | 0.008 ± 0.002 | 0.081 ± 0.010 | 0.006 ± 0.001 | 0.10 ± 0.003 |
| OxTG 16:0_16:0_18:2(OOH) | 880.7609 | [M+NH4]+ | 18.21 | 0.001 ± <0.001 | <0.001 | <0.001 | <0.001 | <0.001 | <0.001 |
| OxTG 16:0_16:0_18:3(OOH) | 878.7448 | [M+NH4]+ | 17.51 | <0.001 | <0.001 | <0.001 | <0.001 | <0.001 | <0.001 |
| OxTG 16:0_16:0_20:1(Ke_or_Epoxy) | 892.7969 | [M+NH4]+ | 21.67 | 0.001 ± 0.001 | <0.001 | <0.001 | <0.001 | <0.001 | <0.001 |
| OxTG 16:0_16:1_18:1(Ke_or_Epoxy) | 862.7505 | [M+NH4]+ | 18.17 | 0.026 ± 0.004 | 0.012 ± 0.002 | 0.008 ± 0.001 | 0.038 ± 0.002 | 0.006 ± 0.001 | 0.035 ± 0.002 |
| OxTG 16:0_18:0_18:1(Ke_or_Epoxy) | 892.7974 | [M+NH4]+ | 19.72 | 0.008 ± 0.003 | 0.004 ± 0.001 | <0.001 | 0.012 ± 0.003 | <0.001 | <0.001 |
| OxTG 16:0_18:1_16:1(Ke_or_Epoxy) | 862.7504 | [M+NH4]+ | 18.44 | 0.026 ± 0.002 | 0.008 ± 0.001 | 0.007 ± 0.003 | 0.026 ± 0.003 | 0.005 ± 0.002 | 0.020 ± 0.002 |
| OxTG 16:0_18:1_18:1(Ke_or_Epoxy) | 890.7816 | [M+NH4]+ | 18.97 | 0.068 ± 0.006 | 0.046 ± 0.002 | 0.046 ± 0.001 | 0.11 ± 0.01 | 0.053 ± 0.001 | 0.050 ± 0.005 |
| OxTG 16:0_18:1_18:1(OOH) | 908.7919 | [M+NH4]+ | 18.69 | <0.001 | <0.001 | <0.001 | <0.001 | <0.001 | <0.001 |
| OxTG 16:0_18:1_18:2(Ke_or_Epoxy) | 888.7659 | [M+NH4]+ | 18.50 | 0.039 ± 0.002 | 0.018 ± 0.002 | 0.032 ± 0.002 | 0.042 ± 0.001 | 0.023 ± 0.004 | 0.005 ± 0.001 |
| OxTG 16:0_18:1_18:2(OOH) | 906.7763 | [M+NH4]+ | 18.24 | 0.019 ± 0.003 | 0.005 ± 0.001 | 0.038 ± 0.002 | 0.027 ± 0.006 | 0.004 ± 0.001 | 0.007 ± 0.003 |
| OxTG 16:0_18:1_20:3(Ke_or_Epoxy) | 914.7823 | [M+NH4]+ | 18.89 | 0.001 ± <0.001 | <0.001 | <0.001 | 0.002 ± 0.001 | <0.001 | <0.001 |
| OxTG 16:0_18:2_18:1(Ke_or_Epoxy) | 888.7664 | [M+NH4]+ | 18.27 | 0.058 ± 0.004 | 0.031 ± 0.002 | 0.040 ± 0.004 | 0.005 ± 0.007 | 0.033 ± 0.002 | <0.001 |
| OxTG 16:0_18:2_18:2(OH) | 888.7663 | [M+NH4]+ | 17.78 | 0.004 ± 0.003 | <0.001 | 0.001 ± 0.001 | 0.003 ± <0.001 | 0.001 ± 0.001 | <0.001 |
| OxTG 16:0_18:2_18:2(OOH) | 904.7608 | [M+NH4]+ | 17.65 | 0.017 ± 0.003 | 0.001 ± 0.001 | 0.007 ± <0.001 | 0.008 ± 0.005 | <0.001 | <0.001 |
| OxTG 16:0_20:4_18:1(Ke_or_Epoxy) | 912.7660 | [M+NH4]+ | 18.03 | 0.001 ± 0.001 | <0.001 | <0.001 | 0.004 ± 0.001 | 0.002 ± 0.001 | <0.001 |
| OxTG 16:0_22:6_18:1(Ke_or_Epoxy) | 936.7650 | [M+NH4]+ | 17.74 | <0.001 | <0.001 | <0.001 | 0.004 ± 0.001 | <0.001 | 0.008 ± 0.001 |
| OxTG 16:1_18:0_18:2(OH) | 890.7817 | [M+NH4]+ | 18.68 | <0.001 | <0.001 | <0.001 | <0.001 | <0.001 | <0.001 |
| OxTG 18:0_18:1_18:1(Ke_or_Epoxy) | 918.8121 | [M+NH4]+ | 20.03 | 0.001 ± <0.001 | 0.001 ± 0.001 | <0.001 | 0.001 ± 0.001 | 0.001 ± 0.001 | <0.001 |
| OxTG 18:0_18:2_18:2(OH) | 916.7975 | [M+NH4]+ | 18.53 | 0.003 ± 0.001 | 0.001 ± <0.001 | 0.001 ± <0.001 | <0.001 | 0.001 ± <0.001 | <0.001 |
| OxTG 18:1_18:1_16:1(Ke_or_Epoxy) | 888.7650 | [M+NH4]+ | 20.07 | <0.001 | <0.001 | <0.001 | <0.001 | <0.001 | <0.001 |
| OxTG 18:1_18:1_17:1(OH) | 904.7979 | [M+NH4]+ | 18.63 | 0.001 ± <0.001 | <0.001 | <0.001 | <0.001 | <0.001 | <0.001 |
| OxTG 18:1_18:1_18:1(Ke_or_Epoxy) | 916.7971 | [M+NH4]+ | 19.09 | 0.012 ± 0.002 | 0.005 ± 0.001 | 0.002 ± 0.002 | 0.001 ± 0.001 | 0.017 ± 0.006 | <0.001 |
| OxTG 18:1_18:1_18:2(OH) | 916.7974 | [M+NH4]+ | 18.31 | 0.014 ± 0.003 | 0.006 ± 0.002 | 0.007 ± 0.001 | 0.008 ± 0.004 | 0.011 ± 0.002 | <0.001 |
| OxTG 18:1_18:1_18:2(OOH) | 932.7931 | [M+NH4]+ | 18.29 | <0.001 | <0.001 | 0.023 ± 0.003 | 0.003 ± 0.004 | <0.001 | 0.007 ± 0.001 |
| OxTG 18:1_18:1_18:3(OOH) | 930.7762 | [M+NH4]+ | 18.62 | <0.001 | <0.001 | <0.001 | <0.001 | <0.001 | 0.003 ± 0.001 |
| OxTG 18:1_18:1_20:3(Ke_or_Epoxy) | 940.7959 | [M+NH4]+ | 18.86 | 0.001 ± 0.001 | <0.001 | <0.001 | <0.001 | 0.001 ± <0.001 | <0.001 |
| OxTG 18:1_18:1_20:4(OOH) | 956.7918 | [M+NH4]+ | 18.39 | <0.001 | <0.001 | <0.001 | 0.002 ± 0.001 | <0.001 | <0.001 |
| OxTG 18:1_18:2_16:1(Ke_or_Epoxy) | 886.7501 | [M+NH4]+ | 17.81 | 0.016 ± 0.001 | 0.003 ± 0.001 | 0.011 ± 0.004 | 0.032 ± 0.001 | 0.009 ± 0.001 | 0.002 ± 0.001 |
| OxTG 18:1_18:2_18:1(Ke_or_Epoxy) | 914.7823 | [M+NH4]+ | 18.64 | 0.018 ± 0.001 | 0.002 ± 0.001 | 0.004 ± 0.001 | 0.003 ± <0.001 | 0.012 ± 0.001 | <0.001 |
| OxTG 18:1_18:2_18:2(OH) | 914.7817 | [M+NH4]+ | 17.87 | 0.013 ± 0.002 | 0.003 ± <0.001 | 0.008 ± 0.002 | 0.016 ± 0.002 | 0.009 ± 0.001 | <0.001 |
| OxTG 18:1_18:2_18:2(OOH) | 930.7775 | [M+NH4]+ | 17.65 | 0.002 ± 0.001 | 0.002 ± 0.001 | 0.004 ± 0.001 | <0.001 | 0.001 ± 0.001 | 0.003 ± 0.001 |
| TG 10:0_10:0_14:0 | 628.5511 | [M+NH4]+ | 16.73 | 0.001 ± <0.001 | <0.001 | <0.001 | 0.058 ± 0.007 | <0.001 | 0.055 ± 0.007 |
| TG 10:0_10:0_16:0 | 656.5835 | [M+NH4]+ | 17.60 | 0.034 ± 0.002 | 0.005 ± 0.001 | 0.006 ± 0.001 | 0.22 ± 0.02 | <0.001 | 0.31 ± 0.005 |
| TG 10:0_12:0_14:0 | 656.5835 | [M+NH4]+ | 17.34 | 0.019 ± 0.001 | 0.023 ± 0.001 | 0.021 ± 0.002 | 0.17 ± 0.02 | 0.001 ± <0.001 | 0.045 ± 0.016 |
| TG 10:0_12:0_16:0 | 684.6141 | [M+NH4]+ | 18.19 | 0.063 ± 0.004 | 0.088 ± 0.003 | 0.023 ± 0.002 | 0.64 ± 0.01 | 0.006 ± <0.001 | 0.15 ± <0.001 |
| TG 10:0_14:0_16:0 | 712.6461 | [M+NH4]+\|[M+Na]+ | 19.01 | 0.097 ± 0.005 | 0.30 ± 0.03 | 0.083 ± 0.002 | 1.54 ± 0.01 | 0.008 ± 0.001 | 0.13 ± 0.002 |
| TG 10:0_15:0_16:0 | 726.6611 | [M+NH4]+ | 19.45 | 0.023 ± 0.001 | 0.058 ± 0.005 | 0.006 ± 0.001 | 0.19 ± 0.01 | <0.001 | 0.029 ± 0.002 |
| TG 10:0_15:0_18:1 | 752.6770 | [M+NH4]+ | 19.46 | 0.034 ± 0.002 | 0.060 ± 0.011 | 0.014 ± 0.002 | 0.23 ± 0.02 | 0.003 ± 0.001 | 0.061 ± 0.002 |
| TG 10:0_15:0_18:2 | 750.6611 | [M+NH4]+ | 18.72 | 0.010 ± 0.002 | 0.015 ± 0.003 | 0.003 ± <0.001 | 0.15 ± 0.01 | <0.001 | 0.022 ± <0.001 |
| TG 10:0_15:1_16:0 | 724.6455 | [M+NH4]+ | 18.79 | 0.021 ± 0.003 | 0.020 ± 0.001 | 0.023 ± 0.004 | 0.32 ± 0.02 | 0.008 ± 0.001 | 0.016 ± 0.003 |
| TG 10:0_16:0_18:1 | 766.6922 | [M+NH4]+\|[M+Na]+ | 19.92 | 0.84 ± 0.05 | 1.66 ± 0.09 | 0.62 ± 0.01 | 5.12 ± 0.15 | 0.10 ± 0.01 | 1.27 ± 0.05 |
| TG 10:0_16:0_18:2 | 764.6770 | [M+NH4]+\|[M+Na]+ | 19.11 | 0.37 ± 0.02 | 0.59 ± 0.08 | 0.24 ± 0.02 | 3.32 ± 0.14 | 0.039 ± 0.006 | 0.40 ± 0.03 |
| TG 10:0_16:0_18:3 | 762.6612 | [M+NH4]+\|[M+Na]+ | 18.46 | 0.072 ± 0.008 | 0.11 ± 0.01 | 0.044 ± 0.001 | 0.83 ± 0.06 | 0.002 ± 0.001 | 0.075 ± 0.002 |
| TG 10:0_16:0_20:4 | 788.6765 | [M+NH4]+ | 18.78 | 0.022 ± 0.009 | 0.053 ± 0.014 | 0.008 ± 0.003 | 0.26 ± 0.004 | <0.001 | 0.023 ± 0.002 |
| TG 10:0_16:0_20:5 | 786.6609 | [M+NH4]+ | 18.18 | <0.001 | <0.001 | <0.001 | 0.11 ± 0.02 | <0.001 | 0.011 ± 0.002 |
| TG 10:0_16:0_22:6 | 812.6756 | [M+NH4]+ | 18.48 | 0.003 ± 0.002 | 0.043 ± 0.002 | 0.001 ± 0.001 | 0.31 ± 0.01 | <0.001 | 0.064 ± 0.003 |
| TG 10:0_18:1_18:2 | 790.6928 | [M+NH4]+\|[M+Na]+ | 19.13 | 0.42 ± 0.02 | 0.62 ± 0.04 | 0.39 ± 0.01 | 1.85 ± 0.02 | 0.10 ± 0.01 | 0.38 ± 0.01 |
| TG 10:0_18:1_18:3 | 788.6764 | [M+NH4]+ | 18.63 | 0.036 ± 0.012 | 0.066 ± 0.003 | 0.023 ± 0.002 | 0.23 ± 0.02 | <0.001 | 0.017 ± 0.005 |
| TG 10:0_18:1_20:4 | 814.6919 | [M+NH4]+ | 18.99 | 0.052 ± 0.002 | 0.064 ± 0.008 | 0.056 ± 0.003 | 0.30 ± 0.02 | 0.016 ± 0.003 | 0.14 ± 0.01 |
| TG 10:0_18:2_18:2 | 788.6764 | [M+NH4]+ | 18.47 | 0.063 ± 0.020 | 0.10 ± 0.00 | 0.048 ± 0.002 | 0.50 ± 0.01 | <0.001 | 0.026 ± 0.003 |
| TG 10:0_18:2_18:3 | 786.6605 | [M+NH4]+ | 17.73 | <0.001 | <0.001 | <0.001 | 0.098 ± 0.007 | <0.001 | <0.001 |
| TG 10:0_18:2_20:4 | 812.6751 | [M+NH4]+ | 18.11 | 0.015 ± 0.001 | 0.027 ± <0.001 | 0.017 ± 0.003 | 0.21 ± 0.01 | 0.002 ± <0.001 | 0.042 ± 0.002 |
| TG 10:0_18:2_8:0 | 652.5515 | [M+NH4]+ | 15.76 | <0.001 | <0.001 | <0.001 | 0.009 ± <0.001 | <0.001 | 0.002 ± 0.001 |
| TG 10:0_20:4_20:4 | 836.6756 | [M+NH4]+ | 17.81 | <0.001 | <0.001 | <0.001 | 0.027 ± 0.006 | <0.001 | 0.002 ± 0.002 |
| TG 12:0_14:0_16:0 | 740.6773 | [M+NH4]+\|[M+Na]+ | 19.92 | 0.28 ± 0.02 | 0.79 ± 0.05 | 0.19 ± 0.005 | 2.66 ± 0.05 | 0.016 ± <0.001 | 0.37 ± 0.01 |
| TG 12:0_15:0_16:0 | 754.6924 | [M+NH4]+ | 20.38 | 0.034 ± 0.002 | 0.10 ± 0.02 | 0.011 ± 0.001 | 0.18 ± 0.01 | <0.001 | 0.036 ± 0.002 |
| TG 12:0_15:0_18:1 | 780.7069 | [M+NH4]+ | 20.38 | 0.10 ± 0.002 | 0.25 ± 0.02 | 0.09 ± 0.002 | 0.42 ± 0.01 | 0.018 ± 0.001 | 0.17 ± 0.004 |
| TG 12:0_15:0_18:2 | 778.6924 | [M+NH4]+ | 19.55 | 0.037 ± 0.002 | 0.071 ± 0.005 | 0.029 ± 0.001 | 0.21 ± 0.01 | 0.008 ± <0.001 | 0.051 ± 0.003 |
| TG 12:0_15:0_18:3 | 776.6763 | [M+NH4]+ | 18.70 | 0.005 ± 0.001 | 0.012 ± 0.001 | 0.003 ± 0.001 | 0.063 ± 0.007 | <0.001 | 0.006 ± 0.001 |
| TG 12:0_16:0_18:1 | 794.7238 | [M+NH4]+ | 20.85 | 3.09 ± 0.03 | 5.10 ± 0.05 | 2.65 ± 0.04 | 7.63 ± 0.15 | 0.65 ± 0.03 | 5.25 ± 0.16 |
| TG 12:0_16:0_18:2 | 792.7082 | [M+NH4]+\|[M+Na]+ | 20.00 | 1.70 ± 0.08 | 2.61 ± 0.29 | 1.52 ± 0.02 | 4.67 ± 0.43 | 0.32 ± 0.07 | 1.71 ± 0.25 |
| TG 12:0_16:0_18:3 | 790.6925 | [M+NH4]+ | 19.66 | 0.031 ± <0.001 | 0.022 ± 0.006 | 0.020 ± 0.003 | 0.054 ± 0.007 | 0.001 ± <0.001 | 0.035 ± 0.006 |
| TG 12:0_16:0_20:4 | 816.7082 | [M+NH4]+ | 19.70 | 0.16 ± 0.01 | 0.19 ± 0.03 | 0.15 ± 0.01 | 0.54 ± 0.05 | 0.029 ± 0.006 | 0.21 ± 0.01 |
| TG 12:0_16:0_20:5 | 814.6918 | [M+NH4]+ | 19.20 | 0.018 ± 0.001 | 0.022 ± 0.003 | 0.022 ± 0.002 | 0.056 ± 0.007 | 0.005 ± 0.002 | 0.031 ± 0.007 |
| TG 12:0_16:0_22:6 | 840.7082 | [M+NH4]+\|[M+Na]+ | 19.30 | 0.051 ± 0.001 | 0.12 ± 0.01 | 0.078 ± 0.004 | 0.36 ± 0.02 | 0.021 ± 0.004 | 0.27 ± 0.03 |
| TG 12:0_16:1_22:6 | 838.6923 | [M+NH4]+ | 18.55 | 0.004 ± <0.001 | 0.016 ± 0.002 | 0.015 ± 0.003 | 0.10 ± 0.01 | 0.005 ± 0.001 | 0.053 ± 0.009 |
| TG 12:0_17:1_18:2 | 804.7082 | [M+NH4]+ | 19.57 | 0.039 ± 0.002 | 0.070 ± 0.003 | 0.050 ± 0.001 | 0.15 ± 0.01 | 0.016 ± 0.002 | 0.051 ± 0.002 |
| TG 12:0_18:1_18:2 | 818.7239 | [M+NH4]+\|[M+Na]+ | 20.02 | 3.47 ± 0.15 | 3.97 ± 0.16 | 4.28 ± 0.08 | 6.34 ± 0.08 | 1.30 ± 0.11 | 2.66 ± 0.08 |
| TG 12:0_18:2_18:2 | 816.7081 | [M+NH4]+ | 19.25 | 0.56 ± 0.02 | 0.72 ± 0.05 | 0.71 ± 0.03 | 1.50 ± 0.04 | 0.20 ± 0.02 | 0.47 ± 0.02 |
| TG 12:0_18:2_18:3 | 814.6910 | [M+NH4]+\|[M+Na]+ | 18.59 | 0.084 ± 0.004 | 0.12 ± 0.004 | 0.10 ± 0.001 | 0.37 ± 0.03 | 0.037 ± 0.001 | 0.085 ± 0.006 |
| TG 12:0_18:2_20:4 | 840.7089 | [M+NH4]+\|[M+Na]+ | 18.89 | 0.15 ± 0.005 | 0.17 ± 0.01 | 0.22 ± 0.01 | 0.80 ± 0.04 | 0.065 ± 0.004 | 0.39 ± 0.02 |
| TG 12:0_18:2_20:5 | 838.6922 | [M+NH4]+ | 18.31 | 0.004 ± 0.002 | 0.006 ± 0.001 | 0.003 ± 0.001 | 0.12 ± 0.004 | 0.003 ± <0.001 | 0.049 ± 0.005 |
| TG 12:0_18:2_22:6 | 864.7091 | [M+NH4]+ | 18.58 | 0.011 ± <0.001 | 0.035 ± 0.002 | 0.041 ± 0.004 | 0.16 ± 0.01 | 0.006 ± 0.001 | 0.11 ± 0.01 |
| TG 12:0_20:4_20:5 | 862.6925 | [M+NH4]+ | 17.93 | <0.001 | <0.001 | 0.001 ± <0.001 | 0.026 ± 0.003 | <0.001 | 0.016 ± 0.001 |
| TG 13:0_15:0_15:0 | 754.6929 | [M+NH4]+ | 18.17 | 0.009 ± 0.002 | 0.014 ± 0.005 | 0.004 ± 0.002 | 0.13 ± 0.004 | <0.001 | 0.007 ± 0.006 |
| TG 13:0_18:2_18:3 | 828.7086 | [M+NH4]+ | 19.19 | 0.014 ± 0.001 | 0.024 ± 0.002 | 0.018 ± 0.001 | 0.077 ± 0.004 | 0.004 ± 0.001 | 0.040 ± 0.002 |
| TG 14:0_14:0_16:0 | 768.7086 | [M+NH4]+ | 20.87 | 0.65 ± 0.01 | 1.56 ± 0.03 | 0.45 ± 0.004 | 2.92 ± 0.05 | 0.058 ± 0.002 | 1.18 ± 0.02 |
| TG 14:0_14:0_22:5 | 842.7239 | [M+NH4]+ | 18.61 | 0.011 ± 0.002 | 0.013 ± 0.001 | 0.014 ± 0.002 | 0.040 ± 0.006 | 0.005 ± 0.001 | 0.008 ± 0.001 |
| TG 14:0_14:1_16:0 | 766.6923 | [M+NH4]+ | 20.33 | 0.020 ± 0.002 | 0.015 ± 0.006 | 0.002 ± <0.001 | 0.028 ± 0.010 | <0.001 | 0.007 ± 0.003 |
| TG 14:0_15:0_16:0 | 782.7242 | [M+NH4]+ | 21.36 | 0.098 ± 0.007 | 0.24 ± 0.04 | 0.073 ± 0.006 | 0.38 ± 0.03 | 0.014 ± 0.001 | 0.23 ± 0.01 |
| TG 14:0_15:0_18:1 | 808.7391 | [M+NH4]+\|[M+Na]+ | 21.34 | 0.47 ± 0.003 | 0.74 ± 0.07 | 0.51 ± 0.01 | 1.09 ± 0.02 | 0.18 ± 0.02 | 1.01 ± 0.01 |
| TG 14:0_15:0_18:2 | 806.7243 | [M+NH4]+ | 20.46 | 0.22 ± 0.01 | 0.32 ± 0.05 | 0.26 ± 0.01 | 0.50 ± 0.03 | 0.078 ± 0.017 | 0.27 ± 0.03 |
| TG 14:0_15:0_18:3 | 804.7085 | [M+NH4]+ | 19.73 | 0.042 ± 0.002 | 0.061 ± 0.011 | 0.051 ± 0.002 | 0.12 ± 0.01 | 0.014 ± 0.002 | 0.039 ± 0.004 |
| TG 14:0_15:0_22:6 | 854.7242 | [M+NH4]+ | 19.71 | 0.003 ± <0.001 | 0.011 ± 0.003 | 0.011 ± 0.001 | 0.043 ± 0.003 | 0.001 ± <0.001 | 0.050 ± 0.005 |
| TG 14:0_16:0_16:0 | 796.7402 | [M+NH4]+ | 21.85 | 1.00 ± 0.02 | 1.68 ± 0.08 | 0.68 ± 0.01 | 3.84 ± 0.04 | 0.14 ± 0.002 | 2.73 ± 0.07 |
| TG 14:0_16:0_16:1 | 794.7239 | [M+NH4]+ | 30.34 | <0.001 | <0.001 | <0.001 | <0.001 | <0.001 | <0.001 |
| TG 14:0_16:0_17:0 | 810.7556 | [M+NH4]+ | 22.22 | 0.11 ± 0.003 | 0.30 ± 0.06 | 0.11 ± 0.01 | 0.51 ± 0.12 | 0.038 ± 0.013 | 0.27 ± 0.11 |
| TG 14:0_16:0_18:0 | 824.7712 | [M+NH4]+\|[M+Na]+ | 22.91 | 2.03 ± 0.07 | 2.85 ± 0.24 | 1.97 ± 0.03 | 6.69 ± 0.45 | 0.64 ± 0.02 | 6.04 ± 0.60 |
| TG 14:0_16:0_18:1 | 822.7554 | [M+NH4]+\|[M+Na]+ | 21.83 | 8.31 ± 0.05 | 9.03 ± 0.34 | 7.39 ± 0.03 | 14.1 ± 0.8 | 3.20 ± 0.10 | 14.2 ± 0.6 |
| TG 14:0_16:0_18:2 | 820.7394 | [M+NH4]+ | 20.93 | 9.44 ± 0.06 | 9.12 ± 1.27 | 9.53 ± 0.05 | 13.2 ± 1.6 | 3.19 ± 0.63 | 8.77 ± 1.74 |
| TG 14:0_16:0_18:4 | 816.7079 | [M+NH4]+ | 20.03 | 0.028 ± 0.003 | 0.030 ± 0.004 | 0.027 ± 0.002 | 0.072 ± 0.006 | 0.008 ± 0.003 | 0.039 ± 0.010 |
| TG 14:0_16:0_20:4 | 844.7390 | [M+NH4]+\|[M+Na]+ | 20.65 | 0.84 ± 0.03 | 0.66 ± 0.08 | 0.76 ± 0.02 | 1.54 ± 0.14 | 0.16 ± 0.03 | 1.01 ± 0.08 |
| TG 14:0_16:0_22:6 | 868.7396 | [M+NH4]+ | 20.22 | 0.19 ± 0.004 | 0.31 ± 0.03 | 0.31 ± 0.01 | 1.06 ± 0.04 | 0.084 ± 0.014 | 1.87 ± 0.08 |
| TG 14:0_16:1_18:1 | 820.7393 | [M+NH4]+ | 18.28 | 0.016 ± 0.001 | 0.018 ± 0.003 | 0.006 ± 0.001 | 0.17 ± 0.01 | <0.001 | 0.016 ± 0.001 |
| TG 14:0_16:1_20:5 | 840.7082 | [M+NH4]+ | 19.08 | 0.051 ± 0.002 | 0.069 ± 0.005 | 0.067 ± 0.005 | 0.35 ± 0.03 | 0.020 ± <0.001 | 0.29 ± 0.02 |
| TG 14:0_16:1_22:6 | 866.7237 | [M+NH4]+ | 19.28 | 0.10 ± 0.002 | 0.13 ± 0.02 | 0.17 ± 0.01 | 0.45 ± 0.05 | 0.044 ± 0.008 | 0.72 ± 0.07 |
| TG 14:0_17:1_18:1 | 834.7545 | [M+NH4]+ | 22.20 | 0.013 ± 0.002 | 0.044 ± 0.002 | 0.029 ± 0.002 | 0.030 ± 0.002 | 0.012 ± 0.002 | 0.010 ± 0.002 |
| TG 14:0_18:1_18:2 | 846.7553 | [M+NH4]+ | 20.95 | 22.1 ± 0.3 | 15.7 ± 0.8 | 26.9 ± 0.2 | 21.5 ± 1.4 | 12.9 ± 1.2 | 16.0 ± 1.1 |
| TG 14:0_18:2_18:2 | 844.7395 | [M+NH4]+\|[M+Na]+ | 20.13 | 5.83 ± 0.17 | 4.13 ± 0.48 | 8.56 ± 0.25 | 6.98 ± 0.30 | 2.95 ± 0.50 | 3.30 ± 0.17 |
| TG 14:0_18:2_18:3 | 842.7243 | [M+NH4]+\|[M+Na]+ | 19.45 | 0.90 ± 0.03 | 0.80 ± 0.03 | 1.37 ± 0.04 | 2.22 ± 0.09 | 0.43 ± 0.04 | 0.93 ± 0.02 |
| TG 14:0_18:2_20:5 | 866.7239 | [M+NH4]+\|[M+Na]+ | 19.10 | 0.15 ± 0.003 | 0.14 ± 0.006 | 0.29 ± 0.01 | 0.84 ± 0.06 | 0.094 ± 0.002 | 0.56 ± 0.05 |
| TG 14:0_18:2_22:6 | 892.7398 | [M+NH4]+ | 19.42 | 0.18 ± 0.01 | 0.20 ± 0.02 | 0.39 ± 0.02 | 0.83 ± 0.08 | 0.11 ± 0.02 | 1.24 ± 0.16 |
| TG 14:0_18:2_8:0 | 708.6133 | [M+NH4]+ | 17.55 | 0.019 ± 0.003 | 0.012 ± 0.002 | 0.010 ± 0.002 | 0.45 ± 0.03 | 0.001 ± <0.001 | 0.16 ± 0.006 |
| TG 14:0_18:3_22:6 | 890.7243 | [M+NH4]+ | 18.68 | <0.001 | 0.002 ± 0.003 | 0.053 ± 0.001 | 0.27 ± 0.01 | 0.003 ± 0.002 | 0.29 ± 0.02 |
| TG 14:0_20:5_22:6 | 914.7252 | [M+NH4]+ | 18.52 | <0.001 | <0.001 | <0.001 | 0.048 ± 0.004 | <0.001 | 0.12 ± 0.01 |
| TG 14:0_22:6_22:6 | 940.7409 | [M+NH4]+ | 18.76 | <0.001 | <0.001 | <0.001 | 0.026 ± 0.003 | <0.001 | 0.10 ± 0.01 |
| TG 14:1_15:0_16:0 | 780.7076 | [M+NH4]+ | 20.61 | 0.007 ± <0.001 | 0.016 ± 0.002 | 0.002 ± <0.001 | 0.034 ± 0.001 | <0.001 | 0.011 ± 0.002 |
| TG 14:1_15:1_16:0 | 778.6923 | [M+NH4]+ | 19.85 | 0.018 ± 0.001 | 0.024 ± 0.004 | 0.019 ± 0.001 | 0.064 ± 0.007 | 0.004 ± 0.001 | 0.037 ± 0.004 |
| TG 14:1_16:0_18:1 | 820.7394 | [M+NH4]+ | 23.28 | 0.001 ± 0.001 | 0.001 ± 0.001 | 0.001 ± 0.001 | 0.003 ± 0.001 | <0.001 | 0.001 ± <0.001 |
| TG 14:1_16:0_18:2 | 818.7242 | [M+NH4]+ | 20.66 | 0.057 ± 0.003 | 0.033 ± 0.010 | 0.045 ± 0.004 | 0.072 ± 0.012 | 0.009 ± 0.004 | 0.066 ± 0.017 |
| TG 14:1_16:0_20:4 | 842.7253 | [M+NH4]+ | 19.86 | 0.25 ± 0.01 | 0.23 ± 0.04 | 0.28 ± 0.01 | 1.07 ± 0.08 | 0.067 ± 0.017 | 1.02 ± 0.06 |
| TG 14:1_16:0_8:0 | 682.5986 | [M+NH4]+ | 17.57 | 0.085 ± 0.009 | 0.014 ± 0.003 | 0.006 ± 0.001 | 0.63 ± 0.03 | <0.001 | 0.93 ± 0.002 |
| TG 14:1_18:1_8:0 | 708.6141 | [M+NH4]+ | 17.77 | <0.001 | 0.002 ± 0.001 | 0.002 ± 0.001 | 0.041 ± 0.008 | <0.001 | 0.018 ± 0.004 |
| TG 15:0_16:0_16:0 | 810.7550 | [M+NH4]+\|[M+Na]+ | 22.39 | 0.25 ± 0.01 | 0.41 ± 0.08 | 0.25 ± 0.004 | 0.70 ± 0.06 | 0.060 ± 0.004 | 0.69 ± 0.04 |
| TG 15:0_16:0_16:1 | 808.7393 | [M+NH4]+ | 21.53 | 0.049 ± 0.001 | 0.058 ± 0.033 | 0.035 ± 0.003 | 0.12 ± 0.01 | 0.004 ± 0.004 | 0.047 ± 0.019 |
| TG 15:0_16:0_18:1 | 836.7705 | [M+NH4]+\|[M+Na]+ | 22.34 | 2.02 ± 0.06 | 2.58 ± 0.16 | 2.74 ± 0.04 | 3.34 ± 0.17 | 1.26 ± 0.11 | 3.38 ± 0.02 |
| TG 15:0_16:0_18:2 | 834.7548 | [M+NH4]+ | 21.65 | 0.28 ± 0.01 | 0.30 ± 0.08 | 0.32 ± 0.01 | 0.32 ± 0.06 | 0.18 ± 0.04 | 0.21 ± 0.08 |
| TG 15:0_16:0_20:4 | 858.7556 | [M+NH4]+ | 21.03 | 0.070 ± 0.004 | 0.069 ± 0.016 | 0.069 ± 0.007 | 0.088 ± 0.012 | 0.032 ± 0.013 | 0.074 ± 0.011 |
| TG 15:0_16:0_20:5 | 856.7402 | [M+NH4]+ | 20.40 | 0.013 ± 0.001 | 0.014 ± 0.006 | 0.017 ± 0.002 | 0.068 ± 0.015 | 0.004 ± 0.001 | 0.094 ± 0.022 |
| TG 15:0_16:0_22:6 | 882.7558 | [M+NH4]+ | 20.61 | 0.020 ± 0.003 | 0.039 ± 0.011 | 0.048 ± 0.004 | 0.11 ± 0.02 | 0.012 ± 0.003 | 0.24 ± 0.02 |
| TG 15:0_16:0_8:0 | 698.6299 | [M+NH4]+ | 18.61 | 0.026 ± 0.004 | 0.038 ± 0.005 | 0.026 ± 0.002 | 0.21 ± 0.01 | 0.015 ± 0.001 | 0.015 ± 0.006 |
| TG 15:0_16:1_18:1 | 834.7551 | [M+NH4]+\|[M+Na]+ | 21.35 | 1.65 ± 0.06 | 1.78 ± 0.22 | 2.38 ± 0.02 | 2.33 ± 0.20 | 1.05 ± 0.13 | 2.05 ± 0.15 |
| TG 15:0_16:1_18:2 | 832.7400 | [M+NH4]+\|[M+Na]+ | 20.47 | 0.47 ± 0.01 | 0.49 ± 0.02 | 0.72 ± 0.002 | 0.75 ± 0.02 | 0.28 ± 0.03 | 0.46 ± 0.02 |
| TG 15:0_17:1_17:2 | 832.7390 | [M+NH4]+ | 18.61 | <0.001 | <0.001 | <0.001 | <0.001 | <0.001 | <0.001 |
| TG 15:0_18:1_18:2 | 860.7709 | [M+NH4]+\|[M+Na]+ | 21.43 | 3.13 ± 0.11 | 2.77 ± 0.20 | 5.61 ± 0.07 | 3.16 ± 0.16 | 2.70 ± 0.24 | 2.59 ± 0.12 |
| TG 15:0_18:1_22:6 | 908.7712 | [M+NH4]+ | 20.68 | 0.051 ± 0.003 | 0.069 ± 0.007 | 0.13 ± 0.01 | 0.17 ± 0.01 | 0.062 ± 0.010 | 0.55 ± 0.04 |
| TG 15:0_18:2_18:2 | 858.7562 | [M+NH4]+\|[M+Na]+ | 20.59 | 0.98 ± 0.03 | 0.88 ± 0.02 | 1.80 ± 0.01 | 1.22 ± 0.02 | 0.83 ± 0.06 | 0.81 ± 0.004 |
| TG 15:0_18:2_18:3 | 856.7399 | [M+NH4]+ | 19.95 | 0.14 ± 0.01 | 0.14 ± 0.01 | 0.28 ± 0.004 | 0.29 ± 0.01 | 0.13 ± 0.003 | 0.16 ± 0.02 |
| TG 15:0_18:2_20:4 | 882.7554 | [M+NH4]+ | 20.28 | 0.11 ± 0.002 | 0.13 ± 0.005 | 0.18 ± 0.003 | 0.41 ± 0.005 | 0.059 ± 0.009 | 0.72 ± 0.01 |
| TG 15:0_18:2_20:5 | 880.7392 | [M+NH4]+ | 19.59 | 0.014 ± 0.002 | 0.019 ± 0.003 | 0.037 ± <0.001 | 0.10 ± 0.01 | 0.005 ± 0.001 | 0.13 ± 0.01 |
| TG 15:0_18:2_22:6 | 906.7552 | [M+NH4]+ | 19.87 | 0.013 ± <0.001 | 0.022 ± 0.005 | 0.057 ± 0.003 | 0.082 ± 0.010 | 0.021 ± 0.006 | 0.19 ± 0.02 |
| TG 15:1_15:1_15:1 | 776.6775 | [M+NH4]+ | 19.05 | 0.013 ± 0.001 | 0.020 ± 0.003 | 0.015 ± 0.001 | 0.072 ± 0.007 | 0.002 ± <0.001 | 0.028 ± 0.002 |
| TG 15:1_16:0_18:3 | 830.7241 | [M+NH4]+ | 20.09 | 0.025 ± 0.002 | 0.031 ± 0.009 | 0.031 ± 0.004 | 0.066 ± 0.010 | 0.011 ± 0.003 | 0.031 ± 0.003 |
| TG 15:1_16:0_20:5 | 854.7240 | [M+NH4]+ | 19.48 | 0.008 ± <0.001 | 0.009 ± 0.001 | 0.013 ± 0.002 | 0.044 ± 0.004 | 0.002 ± 0.001 | 0.047 ± 0.009 |
| TG 15:1_16:0_22:6 | 880.7395 | [M+NH4]+ | 19.78 | <0.001 | <0.001 | <0.001 | <0.001 | <0.001 | <0.001 |
| TG 15:1_16:0_8:0 | 696.6130 | [M+NH4]+ | 17.80 | 0.006 ± <0.001 | 0.005 ± <0.001 | 0.006 ± <0.001 | 0.080 ± 0.001 | 0.003 ± <0.001 | 0.024 ± 0.004 |
| TG 15:1_16:1_18:2 | 830.7239 | [M+NH4]+ | 19.79 | 0.068 ± <0.001 | 0.086 ± 0.003 | 0.10 ± 0.01 | 0.18 ± 0.005 | 0.037 ± 0.004 | 0.077 ± 0.001 |
| TG 15:1_17:1_17:1 | 832.7389 | [M+NH4]+ | 18.40 | <0.001 | <0.001 | <0.001 | 0.12 ± 0.01 | <0.001 | <0.001 |
| TG 15:1_18:2_18:3 | 854.7247 | [M+NH4]+ | 19.33 | 0.009 ± 0.001 | 0.011 ± 0.001 | 0.025 ± 0.001 | 0.061 ± 0.008 | 0.012 ± 0.001 | 0.043 ± 0.008 |
| TG 16:0_16:0_16:0 | 824.7709 | [M+NH4]+ | 21.36 | 0.015 ± 0.002 | 0.043 ± 0.004 | 0.008 ± 0.004 | 0.066 ± 0.012 | 0.002 ± 0.001 | 0.032 ± 0.005 |
| TG 16:0_16:0_16:1 | 822.7555 | [M+NH4]+ | 23.51 | 0.003 ± 0.002 | 0.005 ± 0.002 | 0.004 ± 0.003 | 0.006 ± 0.004 | <0.001 | 0.007 ± 0.005 |
| TG 16:0_16:0_17:0 | 838.7865 | [M+NH4]+\|[M+Na]+ | 23.39 | 0.29 ± 0.004 | 0.50 ± 0.14 | 0.36 ± 0.02 | 0.68 ± 0.09 | 0.089 ± 0.017 | 0.73 ± 0.13 |
| TG 16:0_16:0_17:1 | 836.7712 | [M+NH4]+ | 23.27 | 0.020 ± 0.002 | 0.045 ± 0.013 | 0.026 ± 0.001 | 0.050 ± 0.012 | 0.005 ± 0.003 | 0.017 ± 0.003 |
| TG 16:0_16:0_18:0 | 852.8020 | [M+NH4]+\|[M+Na]+ | 23.93 | 2.27 ± 0.16 | 3.94 ± 0.19 | 3.21 ± 0.05 | 7.41 ± 0.51 | 0.91 ± 0.03 | 5.56 ± 0.74 |
| TG 16:0_16:0_18:1 | 850.7867 | [M+NH4]+\|[M+Na]+ | 22.87 | 21.9 ± 0.4 | 18.3 ± 2.1 | 20.9 ± 0.2 | 26.8 ± 3.7 | 11.5 ± 1.0 | 28.4 ± 2.9 |
| TG 16:0_16:0_19:0 | 866.8184 | [M+NH4]+ | 22.93 | 0.068 ± 0.006 | 0.15 ± 0.06 | 0.057 ± 0.010 | 0.40 ± 0.02 | 0.049 ± 0.009 | 0.37 ± 0.05 |
| TG 16:0_16:0_20:4 | 872.7716 | [M+NH4]+\|[M+Na]+ | 21.62 | 2.13 ± 0.13 | 1.35 ± 0.20 | 1.86 ± 0.05 | 2.88 ± 0.21 | 0.52 ± 0.12 | 2.96 ± 0.26 |
| TG 16:0_16:0_20:5 | 870.7556 | [M+NH4]+\|[M+Na]+ | 20.78 | 0.81 ± 0.04 | 0.52 ± 0.16 | 0.75 ± 0.06 | 2.09 ± 0.16 | 0.19 ± 0.07 | 3.85 ± 0.31 |
| TG 16:0_16:0_22:6 | 896.7710 | [M+NH4]+\|[M+Na]+ | 21.18 | 0.42 ± 0.01 | 0.56 ± 0.05 | 0.68 ± 0.03 | 2.17 ± 0.11 | 0.30 ± 0.02 | 6.07 ± 0.28 |
| TG 16:0_16:0_23:0 | 922.8816 | [M+NH4]+ | 24.94 | 0.13 ± 0.01 | 0.30 ± 0.01 | 0.20 ± 0.01 | 0.38 ± 0.02 | 0.059 ± 0.002 | 0.21 ± 0.03 |
| TG 16:0_16:1_18:1 | 848.7709 | [M+NH4]+\|[M+Na]+ | 21.81 | 32.6 ± 0.2 | 24.5 ± 0.9 | 34.6 ± 0.2 | 34.2 ± 1.5 | 19.7 ± 0.9 | 34.7 ± 1.4 |
| TG 16:0_16:1_8:0 | 710.6297 | [M+NH4]+\|[M+Na]+ | 18.25 | 0.079 ± 0.006 | 0.052 ± 0.025 | 0.007 ± 0.001 | 0.80 ± 0.10 | 0.002 ± 0.001 | 0.27 ± 0.02 |
| TG 16:0_17:0_18:0 | 866.8179 | [M+NH4]+\|[M+Na]+ | 24.36 | 0.14 ± 0.01 | 0.32 ± 0.02 | 0.20 ± 0.01 | 0.38 ± 0.01 | 0.050 ± 0.005 | 0.25 ± 0.03 |
| TG 16:0_17:0_18:1 | 864.8022 | [M+NH4]+\|[M+Na]+ | 23.37 | 1.65 ± 0.04 | 1.87 ± 0.40 | 2.15 ± 0.09 | 2.03 ± 0.21 | 0.77 ± 0.16 | 2.27 ± 0.22 |
| TG 16:0_17:0_20:4 | 886.7866 | [M+NH4]+ | 22.18 | 0.058 ± 0.006 | 0.069 ± 0.003 | 0.068 ± 0.005 | 0.084 ± 0.003 | 0.048 ± 0.006 | 0.095 ± 0.006 |
| TG 16:0_17:0_22:6 | 910.7865 | [M+NH4]+ | 21.54 | 0.004 ± 0.003 | 0.009 ± 0.004 | 0.004 ± 0.001 | 0.012 ± 0.004 | 0.002 ± 0.001 | 0.11 ± 0.01 |
| TG 16:0_17:1_18:1 | 862.7864 | [M+NH4]+\|[M+Na]+ | 22.32 | 4.41 ± 0.16 | 3.91 ± 0.29 | 6.87 ± 0.15 | 4.27 ± 0.28 | 3.42 ± 0.34 | 4.52 ± 0.15 |
| TG 16:0_17:1_18:2 | 860.7711 | [M+NH4]+ | 22.31 | 0.056 ± 0.004 | 0.11 ± 0.01 | 0.13 ± 0.01 | 0.072 ± 0.002 | 0.057 ± 0.010 | 0.031 ± 0.002 |
| TG 16:0_17:1_20:4 | 884.7700 | [M+NH4]+ | 21.09 | 0.15 ± 0.01 | 0.15 ± 0.02 | 0.18 ± 0.01 | 0.31 ± 0.01 | 0.071 ± 0.008 | 0.61 ± 0.005 |
| TG 16:0_18:0_18:0 | 880.8333 | [M+NH4]+\|[M+Na]+ | 24.95 | 0.58 ± 0.08 | 1.44 ± 0.08 | 0.97 ± 0.01 | 1.72 ± 0.05 | 0.20 ± 0.01 | 0.96 ± 0.17 |
| TG 16:0_18:0_18:1 | 878.8170 | [M+NH4]+\|[M+Na]+ | 23.89 | 15.4 ± 0.3 | 14.4 ± 0.9 | 15.7 ± 0.2 | 18.7 ± 1.3 | 6.36 ± 0.47 | 15.1 ± 0.9 |
| TG 16:0_18:0_19:0 | 894.8493 | [M+NH4]+ | 25.45 | 0.017 ± 0.004 | 0.058 ± 0.004 | 0.021 ± 0.003 | 0.058 ± 0.005 | 0.005 ± 0.001 | 0.027 ± 0.003 |
| TG 16:0_18:0_20:0 | 908.8648 | [M+NH4]+\|[M+Na]+ | 25.97 | 0.067 ± 0.013 | 0.25 ± 0.02 | 0.11 ± 0.01 | 0.23 ± 0.01 | 0.029 ± 0.004 | 0.16 ± 0.02 |
| TG 16:0_18:0_22:0 | 936.8961 | [M+NH4]+ | 26.97 | 0.017 ± 0.003 | 0.098 ± 0.013 | 0.023 ± 0.001 | 0.094 ± 0.007 | 0.006 ± 0.001 | 0.084 ± 0.014 |
| TG 16:0_18:0_22:6 | 924.8021 | [M+NH4]+ | 22.16 | 0.20 ± 0.01 | 0.26 ± 0.03 | 0.30 ± 0.01 | 0.75 ± 0.03 | 0.11 ± 0.03 | 1.34 ± 0.07 |
| TG 16:0_18:0_23:0 | 950.9113 | [M+NH4]+ | 27.43 | <0.001 | 0.004 ± 0.001 | 0.001 ± <0.001 | 0.004 ± 0.002 | <0.001 | 0.002 ± 0.001 |
| TG 16:0_18:0_24:0 | 964.9281 | [M+NH4]+ | 27.96 | 0.001 ± <0.001 | 0.034 ± 0.011 | 0.001 ± 0.001 | 0.024 ± 0.010 | <0.001 | 0.012 ± 0.008 |
| TG 16:0_18:0_26:0 | 992.9584 | [M+NH4]+ | 28.63 | <0.001 | 0.016 ± 0.001 | 0.001 ± <0.001 | 0.011 ± 0.001 | <0.001 | 0.005 ± 0.001 |
| TG 16:0_18:1_18:1 | 876.8025 | [M+NH4]+\|[M+Na]+ | 22.81 | 66.6 ± 0.5 | 54.8 ± 5.4 | 86.3 ± 0.5 | 53.9 ± 6.8 | 53.4 ± 6.0 | 51.5 ± 5.3 |
| TG 16:0_18:1_18:2 | 874.7870 | [M+NH4]+\|[M+Na]+ | 21.93 | 68.7 ± 0.5 | 54.5 ± 4.7 | 92.2 ± 1.4 | 52.4 ± 4.4 | 55.9 ± 6.2 | 47.4 ± 3.9 |
| TG 16:0_18:1_18:3 | 872.7691 | [M+NH4]+ | 20.03 | 0.019 ± 0.004 | 0.033 ± 0.035 | 0.024 ± 0.006 | 0.040 ± 0.041 | 0.054 ± 0.035 | 0.024 ± 0.023 |
| TG 16:0_18:1_20:3 | 900.8022 | [M+NH4]+\|[M+Na]+ | 22.35 | 2.10 ± 0.09 | 1.27 ± 0.37 | 1.94 ± 0.17 | 0.95 ± 0.16 | 0.78 ± 0.17 | 1.21 ± 0.25 |
| TG 16:0_18:1_20:4 | 898.7873 | [M+NH4]+\|[M+Na]+ | 21.60 | 9.36 ± 0.24 | 6.33 ± 0.41 | 10.5 ± 0.1 | 8.83 ± 0.17 | 4.93 ± 0.47 | 12.4 ± 0.7 |
| TG 16:0_18:1_21:0 | 920.8652 | [M+NH4]+ | 25.42 | 0.030 ± 0.003 | 0.078 ± 0.014 | 0.033 ± 0.003 | 0.088 ± 0.018 | 0.009 ± 0.005 | 0.060 ± 0.008 |
| TG 16:0_18:1_22:0 | 934.8810 | [M+NH4]+\|[M+Na]+ | 25.93 | 0.21 ± 0.01 | 0.66 ± 0.04 | 0.19 ± 0.01 | 0.60 ± 0.03 | 0.067 ± 0.007 | 0.55 ± 0.03 |
| TG 16:0_18:1_22:1 | 932.8655 | [M+NH4]+ | 25.51 | 0.003 ± 0.001 | 0.002 ± 0.002 | 0.003 ± 0.003 | 0.004 ± 0.001 | 0.002 ± 0.001 | 0.006 ± 0.001 |
| TG 16:0_18:1_22:4 | 926.8170 | [M+NH4]+ | 22.31 | 2.74 ± 0.08 | 1.82 ± 0.11 | 3.06 ± 0.10 | 1.46 ± 0.08 | 1.85 ± 0.11 | 2.88 ± 0.07 |
| TG 16:0_18:1_22:5 | 924.8022 | [M+NH4]+\|[M+Na]+ | 21.55 | 8.67 ± 0.15 | 6.42 ± 0.61 | 11.5 ± 0.2 | 7.32 ± 0.24 | 6.93 ± 0.69 | 14.1 ± 1.0 |
| TG 16:0_18:1_22:6 | 922.7873 | [M+NH4]+ | 21.17 | 3.19 ± 0.08 | 3.37 ± 0.24 | 5.02 ± 0.08 | 6.58 ± 0.44 | 3.26 ± 0.27 | 19.2 ± 1.2 |
| TG 16:0_18:1_23:0 | 948.8964 | [M+NH4]+ | 26.44 | 0.025 ± 0.004 | 0.072 ± 0.010 | 0.025 ± 0.004 | 0.076 ± 0.002 | 0.014 ± 0.001 | 0.055 ± 0.005 |
| TG 16:0_18:1_24:0 | 962.9121 | [M+NH4]+\|[M+Na]+ | 26.93 | 0.11 ± 0.004 | 0.43 ± 0.04 | 0.09 ± 0.003 | 0.32 ± 0.03 | 0.037 ± 0.004 | 0.30 ± 0.02 |
| TG 16:0_18:1_25:0 | 976.9272 | [M+NH4]+ | 27.42 | 0.009 ± 0.001 | 0.028 ± 0.007 | 0.009 ± 0.001 | 0.028 ± 0.003 | 0.005 ± 0.002 | 0.021 ± 0.005 |
| TG 16:0_18:1_8:0 | 738.6614 | [M+NH4]+ | 19.02 | 0.24 ± 0.01 | 0.45 ± 0.05 | 0.17 ± 0.01 | 2.62 ± 0.08 | 0.015 ± 0.002 | 0.37 ± 0.02 |
| TG 16:0_18:2_18:2 | 872.7708 | [M+NH4]+\|[M+Na]+ | 21.06 | 48.2 ± 0.7 | 34.8 ± 1.7 | 68.4 ± 0.9 | 38.2 ± 1.2 | 35.5 ± 2.8 | 29.1 ± 1.7 |
| TG 16:0_18:2_18:3 | 870.7550 | [M+NH4]+\|[M+Na]+ | 20.31 | 11.6 ± 0.3 | 7.40 ± 0.75 | 18.9 ± 0.6 | 12.9 ± 0.1 | 7.28 ± 1.13 | 10.2 ± 0.1 |
| TG 16:0_18:2_18:4 | 868.7399 | [M+NH4]+\|[M+Na]+ | 19.74 | 1.77 ± 0.04 | 1.27 ± 0.04 | 3.15 ± 0.09 | 4.14 ± 0.09 | 1.03 ± 0.05 | 4.49 ± 0.04 |
| TG 16:0_18:2_20:4 | 896.7709 | [M+NH4]+\|[M+Na]+ | 20.77 | 7.73 ± 0.14 | 5.09 ± 0.18 | 9.96 ± 0.21 | 11.3 ± 0.1 | 3.56 ± 0.36 | 18.7 ± 0.4 |
| TG 16:0_18:2_20:5 | 894.7554 | [M+NH4]+\|[M+Na]+ | 20.04 | 1.92 ± 0.04 | 1.63 ± 0.12 | 3.25 ± 0.09 | 5.99 ± 0.10 | 1.01 ± 0.11 | 13.3 ± 0.2 |
| TG 16:0_18:2_22:6 | 920.7711 | [M+NH4]+\|[M+Na]+ | 20.33 | 1.77 ± 0.03 | 1.90 ± 0.15 | 3.74 ± 0.08 | 4.28 ± 0.12 | 1.74 ± 0.20 | 11.5 ± 0.6 |
| TG 16:0_18:2_25:0 | 974.9118 | [M+NH4]+ | 26.70 | <0.001 | 0.001 ± <0.001 | <0.001 | <0.001 | <0.001 | <0.001 |
| TG 16:0_18:2_8:0 | 736.6452 | [M+NH4]+\|[M+Na]+ | 18.29 | 0.099 ± 0.004 | 0.12 ± 0.01 | 0.053 ± 0.002 | 1.20 ± 0.06 | 0.006 ± 0.001 | 0.12 ± 0.01 |
| TG 16:0_18:3_22:6 | 918.7559 | [M+NH4]+ | 19.71 | 0.13 ± 0.001 | 0.12 ± 0.03 | 0.24 ± 0.02 | 0.46 ± 0.07 | 0.060 ± 0.024 | 1.26 ± 0.22 |
| TG 16:0_18:3_23:0 | 944.8654 | [M+NH4]+ | 24.80 | 0.003 ± 0.003 | 0.009 ± 0.006 | 0.007 ± 0.005 | 0.007 ± 0.001 | 0.003 ± 0.002 | 0.006 ± 0.003 |
| TG 16:0_18:3_8:0 | 734.6296 | [M+NH4]+ | 17.87 | 0.010 ± 0.001 | 0.009 ± 0.002 | 0.011 ± 0.001 | 0.12 ± 0.01 | <0.001 | 0.023 ± 0.003 |
| TG 16:0_20:3_22:6 | 946.7864 | [M+NH4]+ | 20.69 | 0.082 ± 0.007 | 0.063 ± 0.019 | 0.10 ± 0.01 | 0.098 ± 0.031 | 0.045 ± 0.011 | 0.40 ± 0.11 |
| TG 16:0_20:4_22:6 | 944.7711 | [M+NH4]+ | 19.64 | 0.39 ± 0.01 | 0.46 ± 0.02 | 0.80 ± 0.02 | 1.51 ± 0.05 | 0.38 ± 0.02 | 3.85 ± 0.03 |
| TG 16:0_20:4_8:0 | 760.6451 | [M+NH4]+ | 17.99 | <0.001 | <0.001 | <0.001 | 0.12 ± 0.004 | <0.001 | <0.001 |
| TG 16:0_20:5_20:5 | 916.7398 | [M+NH4]+\|[M+Na]+ | 19.09 | <0.001 | 0.018 ± 0.002 | 0.024 ± 0.002 | 0.31 ± 0.01 | <0.001 | 1.00 ± 0.05 |
| TG 16:0_20:5_22:6 | 942.7557 | [M+NH4]+\|[M+Na]+ | 19.36 | 0.009 ± 0.002 | 0.023 ± 0.004 | 0.035 ± 0.005 | 0.30 ± 0.01 | 0.006 ± 0.001 | 1.49 ± 0.09 |
| TG 16:0_20:5_8:0 | 758.6298 | [M+NH4]+ | 17.24 | <0.001 | <0.001 | <0.001 | 0.001 ± 0.001 | <0.001 | <0.001 |
| TG 16:0_22:0_22:6 | 980.8649 | [M+NH4]+ | 24.21 | <0.001 | 0.001 ± 0.001 | 0.001 ± <0.001 | 0.005 ± 0.002 | <0.001 | 0.022 ± 0.007 |
| TG 16:0_22:1_22:6 | 978.8490 | [M+NH4]+ | 23.13 | 0.002 ± 0.001 | 0.015 ± 0.002 | 0.006 ± 0.001 | 0.049 ± 0.002 | 0.006 ± 0.001 | 0.080 ± 0.007 |
| TG 16:0_22:6_22:6 | 968.7708 | [M+NH4]+\|[M+Na]+ | 19.64 | 0.013 ± 0.003 | 0.032 ± 0.004 | 0.064 ± 0.005 | 0.20 ± 0.01 | 0.022 ± 0.002 | 1.38 ± 0.08 |
| TG 16:0_22:6_8:0 | 784.6457 | [M+NH4]+ | 17.66 | <0.001 | 0.001 ± <0.001 | <0.001 | 0.11 ± 0.003 | <0.001 | 0.001 ± 0.001 |
| TG 16:1_16:1_8:0 | 708.6136 | [M+NH4]+ | 17.88 | <0.001 | <0.001 | <0.001 | 0.002 ± 0.001 | <0.001 | 0.001 ± <0.001 |
| TG 16:1_17:1_18:2 | 858.7549 | [M+NH4]+ | 21.38 | 0.002 ± <0.001 | 0.001 ± <0.001 | 0.003 ± 0.001 | 0.002 ± 0.001 | 0.001 ± <0.001 | 0.001 ± 0.001 |
| TG 16:1_18:1_18:1 | 874.7868 | [M+NH4]+ | 20.49 | 0.079 ± 0.016 | 0.15 ± 0.01 | 0.13 ± 0.03 | 0.15 ± 0.02 | 0.11 ± 0.01 | 0.060 ± 0.001 |
| TG 16:1_18:1_18:2 | 872.7712 | [M+NH4]+ | 22.27 | 0.018 ± 0.005 | 0.020 ± 0.005 | 0.020 ± 0.008 | 0.023 ± 0.008 | 0.016 ± 0.003 | 0.017 ± 0.006 |
| TG 16:1_18:1_21:0 | 918.8497 | [M+NH4]+ | 24.75 | 0.014 ± 0.008 | 0.026 ± 0.006 | 0.015 ± 0.009 | 0.020 ± 0.003 | 0.005 ± 0.001 | 0.015 ± 0.004 |
| TG 16:1_18:2_18:2 | 870.7547 | [M+NH4]+ | 19.12 | <0.001 | 0.003 ± 0.003 | <0.001 | 0.002 ± 0.001 | <0.001 | 0.002 ± 0.002 |
| TG 16:1_18:2_18:4 | 866.7242 | [M+NH4]+ | 18.89 | 0.059 ± 0.002 | 0.051 ± 0.005 | 0.13 ± 0.003 | 0.23 ± 0.04 | 0.056 ± 0.001 | 0.096 ± 0.012 |
| TG 16:1_18:2_20:5 | 892.7397 | [M+NH4]+ | 19.11 | 0.24 ± 0.003 | 0.19 ± 0.004 | 0.54 ± 0.02 | 0.95 ± 0.08 | 0.18 ± 0.01 | 1.03 ± 0.15 |
| TG 16:1_18:2_22:6 | 918.7557 | [M+NH4]+ | 19.43 | 0.53 ± 0.01 | 0.53 ± 0.04 | 1.14 ± 0.05 | 2.19 ± 0.08 | 0.40 ± 0.04 | 4.06 ± 0.24 |
| TG 16:1_18:2_8:0 | 734.6303 | [M+NH4]+ | 17.61 | 0.016 ± 0.001 | 0.021 ± 0.002 | 0.006 ± <0.001 | 0.25 ± 0.01 | <0.001 | 0.020 ± 0.001 |
| TG 16:1_20:4_20:5 | 916.7396 | [M+NH4]+\|[M+Na]+ | 18.73 | 0.053 ± 0.004 | 0.058 ± 0.012 | 0.13 ± 0.01 | 0.39 ± 0.02 | 0.015 ± 0.015 | 0.82 ± 0.09 |
| TG 16:1_20:5_20:5 | 914.7243 | [M+NH4]+ | 18.16 | <0.001 | <0.001 | 0.002 ± <0.001 | 0.084 ± 0.009 | <0.001 | 0.25 ± 0.002 |
| TG 16:1_20:5_22:6 | 940.7399 | [M+NH4]+ | 18.49 | <0.001 | 0.004 ± 0.002 | 0.005 ± 0.002 | 0.053 ± 0.004 | <0.001 | 0.26 ± 0.02 |
| TG 17:0_17:0_17:2 | 862.7874 | [M+NH4]+ | 20.85 | 1.19 ± 0.05 | 1.21 ± 0.06 | 1.19 ± 0.04 | 1.36 ± 0.10 | 0.62 ± 0.03 | 0.96 ± 0.08 |
| TG 17:0_17:1_17:1 | 862.7848 | [M+NH4]+ | 20.00 | 0.27 ± 0.01 | 0.40 ± 0.07 | 0.30 ± 0.01 | 0.58 ± 0.03 | 0.11 ± 0.004 | 0.29 ± 0.03 |
| TG 17:0_17:1_19:0 | 892.8339 | [M+NH4]+ | 26.30 | <0.001 | 0.001 ± <0.001 | 0.001 ± 0.001 | 0.001 ± 0.001 | <0.001 | <0.001 |
| TG 17:0_18:0_18:0 | 894.8489 | [M+NH4]+ | 25.24 | 0.005 ± 0.001 | 0.018 ± 0.008 | 0.004 ± 0.001 | 0.018 ± 0.005 | 0.001 ± <0.001 | 0.007 ± 0.001 |
| TG 17:0_18:0_18:1 | 892.8340 | [M+NH4]+\|[M+Na]+ | 24.37 | 0.46 ± 0.03 | 0.74 ± 0.04 | 0.57 ± 0.01 | 0.78 ± 0.02 | 0.22 ± 0.02 | 0.51 ± 0.02 |
| TG 17:0_18:0_20:4 | 914.8177 | [M+NH4]+ | 23.12 | 0.025 ± 0.001 | 0.026 ± 0.007 | 0.031 ± 0.005 | 0.017 ± 0.001 | 0.010 ± 0.002 | 0.027 ± 0.003 |
| TG 17:0_18:1_18:1 | 890.8178 | [M+NH4]+\|[M+Na]+ | 23.34 | 2.47 ± 0.09 | 2.58 ± 0.40 | 4.00 ± 0.11 | 2.36 ± 0.16 | 2.05 ± 0.34 | 2.01 ± 0.16 |
| TG 17:0_18:1_18:2 | 888.8026 | [M+NH4]+\|[M+Na]+ | 22.42 | 2.70 ± 0.05 | 2.23 ± 0.39 | 4.47 ± 0.15 | 2.05 ± 0.30 | 2.27 ± 0.47 | 1.59 ± 0.20 |
| TG 17:0_18:1_20:1 | 918.8495 | [M+NH4]+\|[M+Na]+ | 24.35 | 0.20 ± 0.01 | 0.30 ± 0.04 | 0.27 ± 0.01 | 0.31 ± 0.02 | 0.16 ± 0.03 | 0.20 ± 0.02 |
| TG 17:0_18:1_20:2 | 916.8332 | [M+NH4]+ | 23.33 | 0.35 ± 0.01 | 0.41 ± 0.04 | 0.46 ± 0.02 | 0.42 ± 0.03 | 0.36 ± 0.07 | 0.25 ± 0.02 |
| TG 17:0_18:1_20:3 | 914.8178 | [M+NH4]+ | 22.40 | 0.20 ± 0.01 | 0.18 ± 0.01 | 0.26 ± 0.004 | 0.19 ± 0.01 | 0.23 ± 0.02 | 0.12 ± 0.01 |
| TG 17:0_18:1_20:4 | 912.8036 | [M+NH4]+ | 22.11 | 0.14 ± 0.01 | 0.12 ± 0.01 | 0.17 ± 0.01 | 0.14 ± 0.01 | 0.11 ± 0.01 | 0.27 ± 0.005 |
| TG 17:0_18:1_22:3 | 942.8498 | [M+NH4]+ | 23.46 | 0.024 ± 0.001 | 0.023 ± 0.004 | 0.031 ± 0.005 | 0.034 ± 0.004 | 0.026 ± 0.003 | 0.017 ± 0.004 |
| TG 17:0_18:1_22:5 | 938.8177 | [M+NH4]+ | 22.07 | 0.026 ± 0.003 | 0.034 ± 0.005 | 0.041 ± 0.006 | 0.070 ± 0.008 | 0.039 ± 0.004 | 0.19 ± 0.003 |
| TG 17:0_18:1_22:6 | 936.8024 | [M+NH4]+ | 21.50 | 0.035 ± 0.002 | 0.050 ± 0.007 | 0.059 ± 0.005 | 0.12 ± 0.003 | 0.063 ± 0.002 | 0.27 ± 0.02 |
| TG 17:0_18:2_20:4 | 910.7866 | [M+NH4]+ | 21.33 | 0.052 ± 0.012 | 0.063 ± 0.013 | 0.089 ± 0.004 | 0.14 ± 0.03 | 0.038 ± 0.012 | 0.28 ± 0.06 |
| TG 17:0_18:2_22:6 | 934.7856 | [M+NH4]+ | 20.75 | 0.015 ± 0.003 | 0.036 ± 0.010 | 0.073 ± 0.009 | 0.094 ± 0.006 | 0.044 ± 0.008 | 0.26 ± 0.02 |
| TG 17:0_20:4_22:6 | 958.7867 | [M+NH4]+ | 20.00 | <0.001 | <0.001 | <0.001 | 0.010 ± 0.002 | <0.001 | 0.086 ± 0.009 |
| TG 17:0_20:5_22:6 | 956.7712 | [M+NH4]+ | 19.62 | <0.001 | <0.001 | <0.001 | 0.011 ± 0.002 | <0.001 | 0.11 ± 0.01 |
| TG 17:1_17:1_17:1 | 860.7677 | [M+NH4]+ | 23.96 | <0.001 | <0.001 | <0.001 | <0.001 | <0.001 | <0.001 |
| TG 17:1_17:1_17:2 | 858.7523 | [M+NH4]+ | 18.43 | 0.001 ± <0.001 | 0.002 ± 0.002 | <0.001 | 0.056 ± 0.006 | <0.001 | <0.001 |
| TG 17:1_17:1_19:0 | 890.8193 | [M+NH4]+ | 25.37 | <0.001 | <0.001 | <0.001 | <0.001 | <0.001 | <0.001 |
| TG 17:1_17:1_21:0 | 918.8507 | [M+NH4]+ | 26.30 | 0.005 ± 0.002 | 0.006 ± 0.002 | 0.016 ± 0.009 | 0.007 ± 0.004 | 0.007 ± 0.003 | 0.003 ± 0.001 |
| TG 17:1_18:0_18:1 | 890.8184 | [M+NH4]+\|[M+Na]+ | 24.26 | 0.015 ± 0.004 | 0.037 ± 0.014 | 0.022 ± 0.006 | 0.027 ± 0.004 | 0.006 ± 0.002 | 0.012 ± 0.004 |
| TG 17:1_18:1_18:1 | 888.8025 | [M+NH4]+\|[M+Na]+ | 23.20 | 0.037 ± 0.004 | 0.067 ± 0.006 | 0.081 ± 0.005 | 0.070 ± 0.004 | 0.057 ± 0.011 | 0.017 ± 0.004 |
| TG 17:1_18:1_18:2 | 886.7867 | [M+NH4]+\|[M+Na]+ | 21.45 | 1.85 ± 0.06 | 1.54 ± 0.13 | 3.25 ± 0.03 | 1.67 ± 0.08 | 1.64 ± 0.17 | 1.21 ± 0.05 |
| TG 17:1_18:1_20:0 | 918.8495 | [M+NH4]+ | 25.40 | 0.010 ± 0.003 | 0.012 ± 0.001 | 0.020 ± 0.005 | 0.015 ± 0.003 | 0.008 ± 0.001 | 0.010 ± 0.001 |
| TG 17:1_18:1_20:1 | 916.8332 | [M+NH4]+ | 24.11 | 0.013 ± 0.002 | 0.016 ± 0.003 | 0.021 ± 0.008 | 0.021 ± 0.003 | 0.012 ± 0.003 | 0.012 ± 0.001 |
| TG 17:1_18:1_20:2 | 914.8183 | [M+NH4]+ | 23.29 | 0.004 ± 0.002 | 0.007 ± 0.004 | 0.007 ± 0.002 | 0.003 ± 0.002 | 0.001 ± 0.001 | 0.001 ± 0.001 |
| TG 17:1_18:1_20:3 | 912.8017 | [M+NH4]+ | 21.71 | 0.093 ± 0.002 | 0.089 ± 0.018 | 0.13 ± 0.002 | 0.083 ± 0.010 | 0.062 ± 0.011 | 0.12 ± 0.02 |
| TG 17:1_18:1_20:4 | 910.7871 | [M+NH4]+ | 21.16 | 0.15 ± 0.02 | 0.19 ± 0.02 | 0.23 ± 0.005 | 0.52 ± 0.01 | 0.12 ± 0.02 | 1.28 ± 0.01 |
| TG 17:1_18:1_22:1 | 944.8649 | [M+NH4]+ | 24.44 | 0.034 ± 0.006 | 0.067 ± 0.015 | 0.043 ± 0.010 | 0.094 ± 0.007 | 0.028 ± 0.009 | 0.049 ± 0.007 |
| TG 17:1_18:1_22:5 | 936.8024 | [M+NH4]+ | 21.21 | 0.020 ± 0.005 | 0.027 ± 0.003 | 0.037 ± 0.007 | 0.12 ± 0.01 | 0.035 ± 0.003 | 0.25 ± 0.01 |
| TG 17:1_18:2_18:2 | 884.7703 | [M+NH4]+\|[M+Na]+ | 20.65 | 0.51 ± 0.01 | 0.42 ± 0.01 | 0.92 ± 0.01 | 0.62 ± 0.02 | 0.45 ± 0.03 | 0.52 ± 0.04 |
| TG 17:1_18:2_20:4 | 908.7711 | [M+NH4]+ | 20.36 | 0.082 ± 0.006 | 0.11 ± 0.005 | 0.17 ± 0.01 | 0.41 ± 0.02 | 0.077 ± 0.003 | 0.87 ± 0.06 |
| TG 17:1_18:2_22:5 | 934.7863 | [M+NH4]+ | 20.35 | 0.001 ± <0.001 | 0.005 ± 0.002 | 0.008 ± 0.004 | 0.082 ± 0.007 | 0.007 ± 0.001 | 0.15 ± 0.01 |
| TG 17:1_18:2_22:6 | 932.7715 | [M+NH4]+ | 19.93 | 0.005 ± 0.001 | 0.013 ± 0.002 | 0.024 ± 0.005 | 0.064 ± 0.005 | 0.012 ± 0.005 | 0.17 ± 0.01 |
| TG 17:1_20:4_20:5 | 930.7554 | [M+NH4]+ | 19.34 | <0.001 | <0.001 | <0.001 | 0.017 ± 0.001 | <0.001 | 0.11 ± 0.006 |
| TG 17:2_17:2_17:2 | 854.7238 | [M+NH4]+ | 17.74 | <0.001 | <0.001 | <0.001 | 0.007 ± 0.002 | <0.001 | <0.001 |
| TG 17:2_18:2_18:3 | 880.7393 | [M+NH4]+ | 20.06 | <0.001 | <0.001 | <0.001 | 0.008 ± 0.003 | <0.001 | 0.005 ± 0.003 |
| TG 17:2_18:2_22:5 | 932.7723 | [M+NH4]+ | 19.59 | <0.001 | <0.001 | 0.002 ± 0.001 | 0.037 ± 0.003 | 0.001 ± <0.001 | 0.055 ± 0.001 |
| TG 18:0_18:0_18:1 | 906.8496 | [M+NH4]+\|[M+Na]+ | 24.92 | 2.29 ± 0.04 | 3.01 ± 0.42 | 2.38 ± 0.03 | 3.01 ± 0.31 | 0.60 ± 0.10 | 1.82 ± 0.23 |
| TG 18:0_18:0_20:4 | 928.8339 | [M+NH4]+ | 23.60 | 0.20 ± 0.01 | 0.15 ± 0.03 | 0.18 ± 0.01 | 0.092 ± 0.017 | 0.072 ± 0.022 | 0.12 ± 0.04 |
| TG 18:0_18:0_22:6 | 952.8333 | [M+NH4]+\|[M+Na]+ | 23.18 | 0.020 ± 0.002 | 0.029 ± 0.007 | 0.030 ± 0.003 | 0.080 ± 0.013 | 0.010 ± 0.004 | 0.11 ± 0.01 |
| TG 18:0_18:1_18:1 | 904.8336 | [M+NH4]+\|[M+Na]+ | 23.86 | 12.9 ± 0.4 | 12.9 ± 0.5 | 15.0 ± 0.1 | 11.8 ± 0.9 | 9.16 ± 0.94 | 7.71 ± 0.43 |
| TG 18:0_18:1_18:2 | 902.8179 | [M+NH4]+ | 22.89 | 22.9 ± 0.6 | 19.5 ± 3.7 | 28.5 ± 1.1 | 16.7 ± 2.5 | 17.8 ± 4.4 | 11.6 ± 2.3 |
| TG 18:0_18:1_20:3 | 928.8335 | [M+NH4]+ | 23.09 | 1.22 ± 0.03 | 0.98 ± 0.16 | 1.41 ± 0.03 | 0.84 ± 0.08 | 1.08 ± 0.21 | 0.87 ± 0.09 |
| TG 18:0_18:1_20:4 | 926.8187 | [M+NH4]+\|[M+Na]+ | 22.65 | 1.49 ± 0.03 | 1.00 ± 0.08 | 1.45 ± 0.08 | 0.75 ± 0.10 | 1.37 ± 0.09 | 1.37 ± 0.11 |
| TG 18:0_18:1_22:4 | 954.8491 | [M+NH4]+\|[M+Na]+ | 23.25 | 0.18 ± 0.005 | 0.18 ± 0.02 | 0.18 ± 0.003 | 0.18 ± 0.01 | 0.21 ± 0.02 | 0.29 ± 0.02 |
| TG 18:0_18:1_22:5 | 952.8338 | [M+NH4]+ | 22.61 | 0.070 ± 0.010 | 0.047 ± 0.012 | 0.042 ± 0.013 | 0.045 ± 0.016 | 0.052 ± 0.016 | 0.18 ± 0.04 |
| TG 18:0_18:1_22:6 | 950.8171 | [M+NH4]+\|[M+Na]+ | 22.15 | 0.30 ± 0.01 | 0.36 ± 0.02 | 0.47 ± 0.01 | 0.77 ± 0.004 | 0.36 ± 0.04 | 1.63 ± 0.04 |
| TG 18:0_18:1_24:0 | 990.9436 | [M+NH4]+ | 27.90 | 0.029 ± 0.001 | 0.13 ± 0.02 | 0.022 ± 0.002 | 0.087 ± 0.014 | 0.008 ± 0.004 | 0.066 ± 0.009 |
| TG 18:0_18:1_25:0 | 1004.9584 | [M+NH4]+ | 28.36 | 0.001 ± <0.001 | 0.004 ± 0.002 | 0.001 ± <0.001 | 0.004 ± 0.003 | <0.001 | 0.006 ± 0.004 |
| TG 18:0_18:2_18:4 | 896.7709 | [M+NH4]+ | 19.15 | <0.001 | <0.001 | <0.001 | 0.001 ± 0.001 | <0.001 | <0.001 |
| TG 18:0_19:0_20:4 | 942.8495 | [M+NH4]+ | 24.16 | 0.010 ± 0.002 | 0.009 ± 0.002 | 0.015 ± 0.001 | 0.011 ± 0.002 | 0.011 ± 0.002 | 0.010 ± 0.001 |
| TG 18:0_20:0_20:4 | 956.8648 | [M+NH4]+ | 24.68 | 0.013 ± 0.001 | 0.014 ± 0.005 | 0.016 ± 0.001 | 0.010 ± 0.003 | 0.006 ± 0.002 | 0.016 ± 0.003 |
| TG 18:0_20:1_20:4 | 954.8495 | [M+NH4]+ | 23.56 | 0.081 ± 0.007 | 0.073 ± 0.014 | 0.085 ± 0.003 | 0.065 ± 0.007 | 0.066 ± 0.011 | 0.10 ± 0.02 |
| TG 18:0_20:4_22:5 | 974.8178 | [M+NH4]+ | 21.34 | 0.051 ± 0.001 | 0.055 ± 0.008 | 0.082 ± 0.003 | 0.075 ± 0.003 | 0.051 ± 0.008 | 0.31 ± 0.04 |
| TG 18:0_20:4_22:6 | 972.8027 | [M+NH4]+ | 20.96 | 0.022 ± 0.002 | 0.037 ± 0.009 | 0.037 ± 0.013 | 0.053 ± 0.010 | 0.023 ± 0.012 | 0.34 ± 0.02 |
| TG 18:0_20:5_22:6 | 970.7876 | [M+NH4]+ | 20.29 | 0.006 ± 0.001 | 0.013 ± 0.008 | 0.020 ± 0.007 | 0.051 ± 0.009 | 0.006 ± 0.003 | 0.37 ± 0.04 |
| TG 18:0_22:4_22:6 | 1000.8331 | [M+NH4]+ | 21.51 | 0.001 ± 0.001 | 0.005 ± 0.003 | <0.001 | 0.014 ± 0.005 | 0.002 ± 0.002 | 0.045 ± 0.009 |
| TG 18:0_22:6_22:6 | 996.8028 | [M+NH4]+ | 20.56 | <0.001 | 0.001 ± <0.001 | 0.004 ± 0.001 | 0.013 ± 0.002 | <0.001 | 0.12 ± 0.006 |
| TG 18:1_18:1_18:1 | 902.8170 | [M+NH4]+ | 21.44 | 0.48 ± 0.01 | 0.43 ± 0.04 | 0.79 ± 0.04 | 0.43 ± 0.05 | 0.51 ± 0.03 | 0.33 ± 0.02 |
| TG 18:1_18:1_18:2 | 900.8024 | [M+NH4]+\|[M+Na]+ | 21.92 | 25.5 ± 0.2 | 21.5 ± 0.2 | 32.5 ± 0.3 | 21.4 ± 0.5 | 24.8 ± 1.3 | 16.9 ± 0.4 |
| TG 18:1_18:1_18:3 | 898.7860 | [M+NH4]+ | 19.92 | 0.018 ± 0.003 | 0.029 ± 0.005 | 0.082 ± 0.004 | 0.038 ± 0.006 | 0.042 ± 0.004 | 0.010 ± 0.003 |
| TG 18:1_18:1_19:0 | 918.8495 | [M+NH4]+ | 24.52 | 0.072 ± 0.011 | 0.098 ± 0.030 | 0.086 ± 0.009 | 0.087 ± 0.020 | 0.028 ± 0.016 | 0.051 ± 0.021 |
| TG 18:1_18:1_20:0 | 932.8650 | [M+NH4]+\|[M+Na]+ | 24.86 | 0.73 ± 0.02 | 1.43 ± 0.07 | 0.76 ± 0.01 | 1.59 ± 0.05 | 0.52 ± 0.04 | 1.19 ± 0.03 |
| TG 18:1_18:1_20:1 | 930.8491 | [M+NH4]+ | 23.88 | 1.07 ± 0.05 | 1.23 ± 0.25 | 1.12 ± 0.03 | 1.20 ± 0.16 | 0.90 ± 0.32 | 0.83 ± 0.15 |
| TG 18:1_18:1_20:4 | 924.8018 | [M+NH4]+ | 23.44 | <0.001 | <0.001 | 0.001 ± 0.001 | <0.001 | <0.001 | <0.001 |
| TG 18:1_18:1_21:0 | 946.8806 | [M+NH4]+ | 25.39 | 0.014 ± 0.004 | 0.072 ± 0.013 | 0.016 ± 0.004 | 0.089 ± 0.006 | 0.007 ± 0.005 | 0.057 ± 0.005 |
| TG 18:1_18:1_22:0 | 960.8961 | [M+NH4]+\|[M+Na]+ | 25.88 | 0.25 ± 0.01 | 0.72 ± 0.09 | 0.18 ± 0.005 | 0.58 ± 0.09 | 0.086 ± 0.022 | 0.51 ± 0.11 |
| TG 18:1_18:1_22:1 | 958.8807 | [M+NH4]+\|[M+Na]+ | 24.77 | 0.036 ± 0.004 | 0.089 ± 0.051 | 0.038 ± 0.003 | 0.18 ± 0.09 | 0.070 ± 0.017 | 0.12 ± 0.05 |
| TG 18:1_18:1_22:4 | 952.8321 | [M+NH4]+ | 22.30 | 0.74 ± 0.01 | 0.61 ± 0.07 | 0.86 ± 0.03 | 0.55 ± 0.07 | 0.75 ± 0.10 | 1.96 ± 0.11 |
| TG 18:1_18:1_22:5 | 950.8159 | [M+NH4]+ | 21.53 | 1.09 ± 0.01 | 0.97 ± 0.10 | 1.40 ± 0.03 | 1.13 ± 0.04 | 1.15 ± 0.10 | 4.01 ± 0.21 |
| TG 18:1_18:1_22:6 | 948.8022 | [M+NH4]+ | 21.16 | 1.26 ± 0.03 | 1.38 ± 0.04 | 1.90 ± 0.02 | 2.42 ± 0.04 | 1.71 ± 0.11 | 5.71 ± 0.23 |
| TG 18:1_18:1_23:0 | 974.9123 | [M+NH4]+ | 26.42 | 0.021 ± 0.003 | 0.051 ± 0.004 | 0.023 ± 0.002 | 0.062 ± 0.003 | 0.013 ± 0.003 | 0.044 ± 0.002 |
| TG 18:1_18:1_24:0 | 988.9268 | [M+NH4]+\|[M+Na]+ | 26.88 | 0.093 ± 0.007 | 0.33 ± 0.04 | 0.071 ± 0.005 | 0.24 ± 0.03 | 0.046 ± 0.009 | 0.18 ± 0.02 |
| TG 18:1_18:1_24:1 | 986.9117 | [M+NH4]+ | 25.74 | 0.018 ± 0.005 | 0.028 ± 0.012 | 0.015 ± 0.002 | 0.082 ± 0.019 | 0.032 ± 0.003 | 0.049 ± 0.013 |
| TG 18:1_18:1_25:0 | 1002.9434 | [M+NH4]+ | 27.39 | 0.006 ± <0.001 | 0.013 ± 0.004 | 0.008 ± 0.001 | 0.015 ± 0.003 | 0.004 ± 0.001 | 0.010 ± 0.002 |
| TG 18:1_18:1_26:0 | 1016.9582 | [M+NH4]+ | 27.87 | 0.011 ± 0.003 | 0.053 ± 0.008 | 0.008 ± <0.001 | 0.037 ± 0.005 | 0.005 ± 0.002 | 0.028 ± 0.003 |
| TG 18:1_18:2_18:2 | 898.7856 | [M+NH4]+\|[M+Na]+ | 21.10 | 20.0 ± 0.1 | 15.9 ± 0.6 | 28.3 ± 0.2 | 19.1 ± 0.7 | 19.7 ± 0.5 | 15.3 ± 0.9 |
| TG 18:1_18:2_18:3 | 896.7697 | [M+NH4]+\|[M+Na]+ | 20.34 | 8.18 ± 0.04 | 5.72 ± 0.16 | 14.6 ± 0.2 | 9.63 ± 0.54 | 7.62 ± 0.45 | 8.61 ± 0.24 |
| TG 18:1_18:2_18:4 | 894.7551 | [M+NH4]+ | 19.81 | 0.68 ± 0.02 | 0.44 ± 0.01 | 1.12 ± 0.08 | 1.27 ± 0.16 | 0.32 ± 0.03 | 1.37 ± 0.33 |
| TG 18:1_18:2_19:0 | 916.8340 | [M+NH4]+ | 24.36 | 0.017 ± 0.002 | 0.020 ± 0.003 | 0.025 ± 0.005 | 0.020 ± 0.002 | 0.012 ± 0.003 | 0.011 ± 0.002 |
| TG 18:1_18:2_20:2 | 926.8173 | [M+NH4]+ | 23.40 | 0.004 ± 0.003 | 0.003 ± 0.001 | 0.004 ± 0.001 | 0.002 ± 0.001 | 0.003 ± 0.001 | 0.003 ± 0.001 |
| TG 18:1_18:2_20:4 | 922.7865 | [M+NH4]+\|[M+Na]+ | 20.75 | 5.16 ± 0.05 | 4.29 ± 0.17 | 7.58 ± 0.08 | 7.38 ± 0.49 | 4.21 ± 0.05 | 13.3 ± 1.3 |
| TG 18:1_18:2_20:5 | 920.7708 | [M+NH4]+ | 20.12 | 1.35 ± 0.03 | 1.35 ± 0.12 | 2.29 ± 0.09 | 4.18 ± 0.50 | 1.25 ± 0.13 | 7.79 ± 1.40 |
| TG 18:1_18:2_22:0 | 958.8806 | [M+NH4]+\|[M+Na]+ | 24.95 | 0.34 ± 0.01 | 0.75 ± 0.03 | 0.29 ± 0.01 | 0.80 ± 0.02 | 0.20 ± 0.01 | 0.62 ± 0.01 |
| TG 18:1_18:2_22:1 | 956.8651 | [M+NH4]+ | 23.95 | 0.17 ± 0.01 | 0.27 ± 0.02 | 0.14 ± 0.003 | 0.32 ± 0.01 | 0.15 ± 0.02 | 0.21 ± 0.01 |
| TG 18:1_18:2_22:5 | 948.8008 | [M+NH4]+\|[M+Na]+ | 20.69 | 0.33 ± 0.01 | 0.31 ± 0.04 | 0.54 ± 0.01 | 0.53 ± 0.02 | 0.36 ± 0.01 | 1.35 ± 0.12 |
| TG 18:1_18:2_22:6 | 946.7865 | [M+NH4]+\|[M+Na]+ | 20.34 | 0.90 ± 0.03 | 0.99 ± 0.10 | 1.63 ± 0.04 | 2.13 ± 0.17 | 1.01 ± 0.17 | 4.59 ± 0.29 |
| TG 18:1_18:2_23:0 | 972.8962 | [M+NH4]+ | 24.98 | 0.024 ± 0.002 | 0.016 ± 0.004 | 0.025 ± 0.001 | 0.017 ± 0.002 | 0.021 ± 0.002 | 0.013 ± 0.003 |
| TG 18:1_18:2_24:0 | 986.9116 | [M+NH4]+\|[M+Na]+ | 25.97 | 0.085 ± 0.004 | 0.25 ± 0.02 | 0.067 ± 0.002 | 0.20 ± 0.02 | 0.039 ± 0.012 | 0.13 ± 0.02 |
| TG 18:1_18:2_24:1 | 984.8979 | [M+NH4]+ | 25.02 | 0.026 ± 0.004 | 0.074 ± 0.010 | 0.012 ± 0.011 | 0.081 ± 0.008 | 0.006 ± 0.004 | 0.044 ± 0.008 |
| TG 18:1_18:2_25:0 | 1000.9277 | [M+NH4]+ | 26.48 | 0.002 ± <0.001 | 0.002 ± 0.001 | 0.003 ± <0.001 | 0.003 ± <0.001 | 0.001 ± 0.001 | 0.005 ± 0.001 |
| TG 18:1_18:2_26:0 | 1014.9433 | [M+NH4]+ | 26.92 | 0.009 ± 0.002 | 0.038 ± 0.002 | 0.009 ± 0.001 | 0.030 ± 0.004 | 0.007 ± 0.001 | 0.017 ± 0.003 |
| TG 18:1_18:3_23:0 | 970.8808 | [M+NH4]+ | 24.32 | 0.012 ± 0.001 | 0.014 ± 0.002 | 0.016 ± 0.002 | 0.024 ± 0.004 | 0.013 ± 0.004 | 0.012 ± 0.001 |
| TG 18:1_19:0_20:4 | 940.8347 | [M+NH4]+ | 23.11 | 0.017 ± 0.002 | 0.016 ± 0.003 | 0.023 ± 0.002 | 0.018 ± 0.001 | 0.025 ± 0.003 | 0.032 ± 0.003 |
| TG 18:1_20:0_22:6 | 978.8494 | [M+NH4]+ | 22.31 | 0.034 ± 0.004 | 0.044 ± 0.009 | 0.043 ± 0.006 | 0.060 ± 0.011 | 0.071 ± 0.021 | 0.16 ± 0.02 |
| TG 18:1_20:1_22:4 | 980.8651 | [M+NH4]+ | 23.26 | 0.006 ± 0.002 | 0.005 ± 0.002 | 0.007 ± 0.002 | 0.014 ± 0.002 | 0.013 ± 0.013 | 0.078 ± 0.006 |
| TG 18:1_20:1_22:5 | 978.8499 | [M+NH4]+ | 22.72 | <0.001 | 0.001 ± 0.001 | <0.001 | 0.003 ± <0.001 | <0.001 | 0.026 ± 0.007 |
| TG 18:1_20:1_22:6 | 976.8341 | [M+NH4]+ | 22.11 | 0.025 ± 0.006 | 0.031 ± 0.001 | 0.030 ± 0.006 | 0.053 ± 0.008 | 0.032 ± 0.005 | 0.12 ± 0.004 |
| TG 18:1_20:1_24:1 | 1014.9431 | [M+NH4]+ | 26.76 | 0.003 ± 0.001 | 0.006 ± 0.005 | 0.003 ± <0.001 | 0.007 ± 0.004 | 0.005 ± 0.001 | 0.006 ± 0.002 |
| TG 18:1_20:2_24:1 | 1012.9269 | [M+NH4]+ | 25.80 | 0.004 ± <0.001 | 0.003 ± <0.001 | 0.004 ± 0.001 | 0.008 ± 0.001 | 0.004 ± 0.001 | 0.005 ± 0.002 |
| TG 18:1_20:3_22:4 | 976.8347 | [M+NH4]+ | 21.72 | 0.052 ± 0.008 | 0.067 ± 0.012 | 0.050 ± 0.008 | 0.049 ± 0.005 | 0.068 ± 0.023 | 0.27 ± 0.03 |
| TG 18:1_20:3_24:1 | 1010.9114 | [M+NH4]+ | 24.83 | 0.006 ± 0.001 | 0.006 ± 0.003 | 0.002 ± 0.001 | 0.018 ± 0.001 | 0.003 ± 0.001 | 0.019 ± 0.003 |
| TG 18:1_20:4_20:4 | 946.7857 | [M+NH4]+ | 19.41 | 0.034 ± 0.010 | 0.028 ± 0.005 | 0.049 ± 0.016 | 0.10 ± 0.01 | 0.030 ± 0.006 | 0.15 ± 0.03 |
| TG 18:1_20:4_21:0 | 968.8644 | [M+NH4]+ | 24.07 | 0.003 ± <0.001 | 0.003 ± 0.001 | 0.005 ± 0.002 | 0.007 ± 0.002 | 0.004 ± 0.001 | 0.010 ± 0.002 |
| TG 18:1_20:4_22:0 | 982.8805 | [M+NH4]+ | 24.58 | 0.008 ± 0.001 | 0.018 ± 0.002 | 0.013 ± 0.002 | 0.020 ± 0.002 | 0.009 ± 0.001 | 0.035 ± 0.006 |
| TG 18:1_20:4_22:4 | 974.8187 | [M+NH4]+ | 21.17 | 0.047 ± 0.010 | 0.056 ± 0.007 | 0.054 ± 0.008 | 0.068 ± 0.011 | 0.071 ± 0.006 | 0.26 ± 0.03 |
| TG 18:1_20:4_22:5 | 972.8014 | [M+NH4]+\|[M+Na]+ | 20.36 | 0.084 ± 0.005 | 0.11 ± 0.002 | 0.12 ± 0.003 | 0.19 ± 0.001 | 0.086 ± 0.004 | 0.84 ± 0.02 |
| TG 18:1_20:4_22:6 | 970.7873 | [M+NH4]+\|[M+Na]+ | 20.01 | 0.11 ± 0.002 | 0.17 ± 0.01 | 0.23 ± 0.01 | 0.43 ± 0.01 | 0.13 ± 0.02 | 2.19 ± 0.05 |
| TG 18:1_20:4_24:0 | 1010.9116 | [M+NH4]+ | 25.63 | 0.003 ± <0.001 | 0.003 ± 0.002 | 0.002 ± 0.001 | 0.003 ± 0.001 | 0.001 ± <0.001 | 0.007 ± 0.001 |
| TG 18:1_20:4_24:1 | 1008.8962 | [M+NH4]+ | 24.49 | 0.002 ± 0.001 | 0.004 ± <0.001 | 0.001 ± <0.001 | 0.009 ± 0.001 | 0.002 ± 0.001 | 0.019 ± <0.001 |
| TG 18:1_20:4_8:0 | 786.6603 | [M+NH4]+ | 18.02 | <0.001 | <0.001 | <0.001 | 0.25 ± 0.02 | <0.001 | 0.019 ± 0.003 |
| TG 18:1_20:5_22:6 | 968.7709 | [M+NH4]+\|[M+Na]+ | 19.28 | 0.038 ± 0.002 | 0.084 ± 0.005 | 0.11 ± 0.01 | 0.48 ± 0.01 | 0.045 ± 0.004 | 2.24 ± 0.05 |
| TG 18:1_22:0_22:0 | 1018.9739 | [M+NH4]+ | 28.59 | 0.008 ± 0.001 | 0.053 ± 0.008 | 0.005 ± 0.001 | 0.032 ± 0.003 | 0.002 ± <0.001 | 0.037 ± 0.002 |
| TG 18:1_22:0_22:6 | 1006.8811 | [M+NH4]+ | 24.08 | <0.001 | 0.001 ± 0.002 | <0.001 | 0.018 ± 0.003 | <0.001 | 0.050 ± 0.002 |
| TG 18:1_22:0_23:0 | 1032.9905 | [M+NH4]+ | 28.79 | <0.001 | 0.003 ± <0.001 | 0.006 ± 0.001 | 0.008 ± <0.001 | 0.002 ± 0.001 | 0.032 ± 0.002 |
| TG 18:1_22:0_24:0 | 1047.0058 | [M+NH4]+ | 28.93 | 0.004 ± <0.001 | 0.024 ± 0.003 | 0.002 ± <0.001 | 0.013 ± 0.002 | 0.001 ± <0.001 | 0.021 ± 0.002 |
| TG 18:1_22:4_22:6 | 998.8177 | [M+NH4]+ | 20.68 | 0.009 ± 0.001 | 0.022 ± 0.007 | 0.017 ± 0.002 | 0.029 ± 0.005 | 0.016 ± <0.001 | 0.35 ± 0.03 |
| TG 18:1_22:5_22:6 | 996.8022 | [M+NH4]+ | 19.98 | 0.006 ± 0.002 | 0.018 ± 0.001 | 0.018 ± 0.002 | 0.056 ± 0.002 | 0.009 ± 0.001 | 0.48 ± 0.01 |
| TG 18:1_22:6_22:6 | 994.7872 | [M+NH4]+\|[M+Na]+ | 19.63 | 0.005 ± 0.001 | 0.025 ± 0.001 | 0.034 ± 0.003 | 0.13 ± 0.005 | 0.026 ± <0.001 | 0.94 ± 0.04 |
| TG 18:1_24:0_24:1 | 1073.0204 | [M+NH4]+ | 28.94 | <0.001 | 0.003 ± 0.002 | 0.001 ± 0.001 | 0.004 ± <0.001 | <0.001 | 0.004 ± 0.002 |
| TG 18:2_18:2_18:2 | 896.7710 | [M+NH4]+ | 18.48 | <0.001 | <0.001 | <0.001 | 0.034 ± 0.002 | <0.001 | 0.003 ± 0.001 |
| TG 18:2_18:2_18:3 | 894.7536 | [M+NH4]+\|[M+Na]+ | 19.56 | 1.43 ± 0.01 | 0.95 ± 0.03 | 3.15 ± 0.06 | 2.48 ± 0.13 | 1.30 ± 0.04 | 1.73 ± 0.13 |
| TG 18:2_18:2_20:5 | 918.7551 | [M+NH4]+ | 19.31 | 0.20 ± 0.003 | 0.21 ± 0.02 | 0.43 ± 0.01 | 1.04 ± 0.04 | 0.16 ± 0.003 | 1.45 ± 0.08 |
| TG 18:2_18:2_23:0 | 970.8808 | [M+NH4]+ | 24.53 | 0.010 ± 0.001 | 0.014 ± 0.002 | 0.013 ± 0.002 | 0.017 ± 0.004 | 0.010 ± 0.003 | 0.007 ± 0.002 |
| TG 18:2_18:2_24:1 | 982.8797 | [M+NH4]+ | 23.98 | 0.017 ± 0.002 | 0.020 ± 0.004 | 0.015 ± 0.001 | 0.050 ± 0.003 | 0.026 ± 0.005 | 0.039 ± 0.005 |
| TG 18:2_18:2_25:0 | 998.9126 | [M+NH4]+ | 24.69 | 0.001 ± 0.001 | 0.002 ± 0.001 | 0.002 ± 0.001 | 0.002 ± 0.001 | <0.001 | 0.001 ± 0.001 |
| TG 18:2_18:2_8:0 | 760.6455 | [M+NH4]+ | 17.63 | <0.001 | <0.001 | <0.001 | 0.14 ± 0.01 | <0.001 | <0.001 |
| TG 18:2_18:3_18:3 | 892.7401 | [M+NH4]+\|[M+Na]+ | 18.96 | 0.13 ± 0.001 | 0.092 ± 0.004 | 0.33 ± 0.01 | 0.43 ± 0.03 | 0.13 ± 0.01 | 0.26 ± 0.07 |
| TG 18:2_18:3_8:0 | 758.6275 | [M+NH4]+ | 16.98 | <0.001 | <0.001 | <0.001 | 0.001 ± 0.001 | <0.001 | <0.001 |
| TG 18:2_20:4_20:5 | 942.7553 | [M+NH4]+\|[M+Na]+ | 18.99 | 0.069 ± 0.003 | 0.11 ± 0.01 | 0.17 ± 0.01 | 0.82 ± 0.01 | 0.046 ± 0.012 | 2.89 ± 0.06 |
| TG 18:2_20:5_20:5 | 940.7397 | [M+NH4]+\|[M+Na]+ | 18.36 | <0.001 | 0.015 ± 0.003 | 0.018 ± 0.001 | 0.33 ± 0.01 | <0.001 | 1.21 ± 0.03 |
| TG 18:2_20:5_22:6 | 966.7557 | [M+NH4]+\|[M+Na]+ | 18.62 | 0.007 ± 0.001 | 0.031 ± 0.002 | 0.044 ± 0.002 | 0.27 ± 0.01 | 0.011 ± 0.001 | 1.31 ± 0.02 |
| TG 18:2_22:0_24:0 | 1044.9894 | [M+NH4]+ | 28.61 | 0.002 ± 0.002 | 0.009 ± 0.003 | 0.001 ± 0.001 | 0.005 ± 0.002 | <0.001 | 0.007 ± 0.002 |
| TG 18:2_22:5_22:5 | 996.8027 | [M+NH4]+ | 20.25 | <0.001 | 0.015 ± 0.001 | 0.001 ± <0.001 | 0.022 ± 0.003 | 0.012 ± 0.002 | 0.28 ± 0.02 |
| TG 18:2_22:5_22:6 | 994.7867 | [M+NH4]+ | 19.23 | 0.001 ± <0.001 | 0.004 ± <0.001 | 0.006 ± 0.001 | 0.028 ± 0.002 | 0.002 ± 0.001 | 0.19 ± 0.005 |
| TG 18:3_18:3_18:3 | 890.7240 | [M+NH4]+ | 18.34 | <0.001 | <0.001 | 0.001 ± 0.001 | <0.001 | <0.001 | 0.014 ± 0.004 |
| TG 18:3_18:3_20:4 | 916.7399 | [M+NH4]+ | 19.34 | <0.001 | <0.001 | <0.001 | 0.001 ± 0.001 | <0.001 | 0.005 ± 0.002 |
| TG 18:3_20:5_20:5 | 938.7242 | [M+NH4]+ | 17.75 | <0.001 | <0.001 | <0.001 | 0.036 ± 0.002 | <0.001 | 0.078 ± 0.002 |
| TG 20:1_20:4_22:5 | 1000.8335 | [M+NH4]+ | 21.40 | 0.001 ± 0.001 | 0.008 ± 0.001 | 0.001 ± 0.001 | 0.023 ± 0.002 | 0.006 ± 0.002 | 0.080 ± 0.017 |
| TG 20:2_22:5_22:6 | 1022.8188 | [M+NH4]+ | 20.18 | <0.001 | <0.001 | <0.001 | 0.001 ± 0.001 | <0.001 | 0.12 ± 0.003 |
| TG 20:4_20:4_22:6 | 992.7710 | [M+NH4]+ | 18.90 | 0.003 ± 0.001 | 0.017 ± 0.002 | 0.026 ± 0.002 | 0.076 ± 0.002 | 0.013 ± 0.001 | 0.50 ± 0.02 |
| TG 20:4_20:5_20:5 | 964.7402 | [M+NH4]+ | 18.10 | <0.001 | 0.001 ± <0.001 | <0.001 | 0.023 ± 0.002 | <0.001 | 0.11 ± 0.01 |
| TG 20:4_20:5_22:5 | 992.7712 | [M+NH4]+ | 18.70 | <0.001 | 0.003 ± 0.001 | 0.001 ± <0.001 | 0.022 ± 0.006 | 0.001 ± 0.001 | 0.12 ± 0.03 |
| TG 20:4_20:5_22:6 | 990.7560 | [M+NH4]+ | 18.35 | <0.001 | 0.003 ± <0.001 | 0.002 ± 0.001 | 0.028 ± 0.001 | <0.001 | 0.17 ± 0.01 |
| TG 20:4_22:6_22:6 | 1016.7715 | [M+NH4]+ | 18.61 | <0.001 | 0.002 ± 0.001 | 0.001 ± <0.001 | 0.008 ± 0.003 | <0.001 | 0.090 ± 0.002 |
| TG 20:5_20:5_20:5 | 962.7248 | [M+NH4]+ | 17.54 | <0.001 | 0.001 ± <0.001 | <0.001 | 0.011 ± <0.001 | <0.001 | 0.13 ± 0.005 |
| TG 20:5_20:5_22:5 | 990.7561 | [M+NH4]+ | 18.08 | <0.001 | <0.001 | <0.001 | 0.004 ± 0.001 | <0.001 | 0.053 ± 0.003 |
| TG 20:5_20:5_22:6 | 988.7402 | [M+NH4]+ | 17.78 | <0.001 | 0.004 ± 0.001 | <0.001 | 0.024 ± <0.001 | <0.001 | 0.16 ± 0.004 |
| TG 20:5_22:6_22:6 | 1014.7546 | [M+NH4]+ | 18.03 | <0.001 | <0.001 | <0.001 | 0.008 ± <0.001 | <0.001 | 0.098 ± 0.002 |
|  |  |  |  |  |  |  |  |  |  |
| ***Glycerophospholipids*** |  |  |  |  |  |  |  |  |  |
| BMP 16:0_20:4 | 788.5435 | [M+NH4]+ | 11.33 | <0.001 | <0.001 | <0.001 | 0.003 ± <0.001 | 0.002 ± 0.001 | <0.001 |
| DMPE 14:0_16:0 | 690.5072 | [M-H]- | 12.26 | 0.002 ± 0.001 | 0.004 ± 0.002 | 0.002 ± 0.001 | 0.005 ± 0.003 | 0.001 ± <0.001 | 0.005 ± 0.001 |
| DMPE 14:0_18:2 | 714.5071 | [M-H]- | 11.34 | 0.005 ± 0.001 | 0.011 ± 0.001 | 0.011 ± 0.002 | 0.010 ± 0.003 | 0.006 ± 0.002 | 0.004 ± 0.001 |
| DMPE 15:0_20:4 | 752.5233 | [M-H]- | 12.83 | <0.001 | <0.001 | 0.001 ± 0.001 | 0.003 ± 0.002 | 0.001 ± 0.001 | 0.002 ± 0.003 |
| DMPE 16:0_16:1 | 716.5219 | [M-H]- | 12.44 | 0.009 ± 0.002 | 0.001 ± 0.001 | 0.002 ± 0.001 | 0.010 ± 0.003 | 0.002 ± 0.002 | 0.027 ± 0.003 |
| DMPE 16:0_18:0 | 746.5700 | [M-H]- | 14.78 | <0.001 | <0.001 | <0.001 | <0.001 | <0.001 | <0.001 |
| DMPE 16:0_18:1 | 744.5543 | [M-H]- | 13.98 | 0.34 ± 0.004 | 0.30 ± 0.03 | 0.33 ± 0.01 | 0.38 ± 0.04 | 0.35 ± 0.01 | 0.47 ± 0.05 |
| DMPE 16:0_18:2 | 742.5380 | [M-H]- | 12.71 | 0.68 ± 0.06 | 0.64 ± 0.08 | 0.74 ± 0.02 | 0.77 ± 0.08 | 0.79 ± 0.08 | 0.61 ± 0.04 |
| DMPE 16:0_20:3 | 768.5532 | [M-H]- | 13.09 | 0.13 ± 0.01 | 0.15 ± 0.05 | 0.17 ± 0.01 | 0.11 ± 0.01 | 0.19 ± 0.05 | 0.11 ± 0.01 |
| DMPE 16:0_20:4 | 766.5385 | [M-H]- | 12.42 | 0.24 ± 0.02 | 0.27 ± 0.05 | 0.25 ± 0.02 | 0.21 ± 0.01 | 0.64 ± 0.04 | 0.25 ± 0.01 |
| DMPE 16:0_20:5 | 764.5222 | [M-H]- | 11.52 | 0.018 ± 0.003 | 0.035 ± 0.002 | 0.040 ± <0.001 | 0.13 ± 0.01 | 0.030 ± 0.002 | 0.24 ± 0.01 |
| DMPE 16:0_22:5 | 792.5542 | [M-H]- | 16.01 | <0.001 | <0.001 | <0.001 | <0.001 | <0.001 | <0.001 |
| DMPE 16:0_22:6 | 790.5371 | [M-H]- | 11.15 | 0.004 ± 0.002 | 0.001 ± <0.001 | 0.008 ± 0.002 | 0.007 ± 0.004 | 0.004 ± 0.002 | <0.001 |
| DMPE 16:1_20:5 | 762.5084 | [M-H]- | 11.17 | <0.001 | 0.001 ± 0.002 | <0.001 | 0.003 ± 0.001 | 0.011 ± 0.009 | 0.024 ± 0.014 |
| DMPE 17:0_22:5 | 806.5704 | [M-H]- | 12.83 | 3.37 ± 0.66 | 4.51 ± 1.08 | 3.98 ± 0.47 | 3.74 ± 0.44 | 4.68 ± 0.83 | 3.41 ± 0.11 |
| DMPE 18:0_18:1 | 772.5853 | [M-H]- | 15.28 | 0.076 ± 0.006 | 0.067 ± 0.006 | 0.088 ± 0.011 | 0.078 ± 0.004 | 0.063 ± 0.002 | 0.073 ± 0.008 |
| DMPE 18:0_18:2 | 770.5686 | [M-H]- | 14.41 | 0.47 ± 0.05 | 0.58 ± 0.07 | 0.59 ± 0.02 | 0.53 ± 0.07 | 0.78 ± 0.02 | 0.47 ± 0.05 |
| DMPE 18:0_20:3 | 796.5848 | [M-H]- | 14.60 | 0.069 ± 0.005 | 0.062 ± 0.006 | 0.10 ± 0.004 | 0.051 ± 0.009 | 0.069 ± 0.002 | 0.058 ± 0.006 |
| DMPE 18:0_20:4 | 794.5710 | [M-H]- | 14.02 | 0.026 ± 0.006 | 0.037 ± 0.025 | 0.057 ± 0.014 | 0.017 ± 0.018 | 0.036 ± 0.016 | 0.013 ± 0.019 |
| DMPE 18:0_20:5 | 792.5536 | [M-H]- | 12.86 | 0.046 ± 0.004 | 0.068 ± 0.019 | 0.097 ± 0.011 | 0.13 ± 0.02 | 0.090 ± 0.006 | 0.17 ± 0.01 |
| DMPE 18:0_22:5 | 820.5855 | [M-H]- | 15.59 | 0.001 ± 0.002 | <0.001 | 0.001 ± 0.002 | <0.001 | 0.012 ± 0.008 | <0.001 |
| DMPE 18:1_18:2 | 768.5531 | [M-H]- | 12.88 | 0.060 ± 0.013 | 0.13 ± 0.04 | 0.084 ± 0.017 | 0.089 ± 0.017 | 0.28 ± 0.09 | 0.21 ± 0.04 |
| DMPE 18:1_20:3 | 794.5704 | [M-H]- | 15.05 | 0.006 ± 0.001 | 0.010 ± 0.007 | 0.010 ± 0.002 | 0.020 ± <0.001 | 0.022 ± 0.005 | 0.019 ± 0.002 |
| DMPE 18:1_20:4 | 792.5530 | [M-H]- | 12.50 | 0.019 ± 0.005 | 0.019 ± 0.007 | 0.022 ± 0.003 | 0.028 ± 0.003 | 0.027 ± 0.006 | 0.042 ± 0.008 |
| DMPE 18:2_18:2 | 766.5378 | [M-H]- | 11.73 | 0.038 ± 0.019 | 0.055 ± 0.019 | 0.053 ± 0.011 | 0.068 ± 0.007 | 0.078 ± 0.023 | 0.031 ± 0.012 |
| DMPE 18:2_20:4 | 790.5387 | [M-H]- | 11.47 | 0.025 ± 0.010 | 0.029 ± 0.010 | 0.046 ± 0.013 | 0.016 ± 0.001 | 0.036 ± 0.017 | 0.007 ± 0.003 |
| DMPE 20:3_20:3 | 818.5715 | [M-H]- | 12.96 | 0.002 ± 0.002 | <0.001 | 0.002 ± 0.004 | 0.004 ± 0.003 | <0.001 | 0.007 ± 0.003 |
| DMPE 20:3_20:4 | 816.5549 | [M-H]- | 12.27 | 0.24 ± 0.01 | 0.25 ± 0.03 | 0.33 ± 0.02 | 0.28 ± 0.05 | 0.28 ± 0.01 | 0.36 ± 0.12 |
| DMPE 22:6_22:6 | 862.5410 | [M-H]- | 11.60 | 0.023 ± 0.001 | 0.004 ± 0.002 | 0.006 ± 0.004 | 0.001 ± 0.001 | 0.003 ± 0.002 | <0.001 |
| LPC O-16:0 | 482.3611 | [M+H]+ | 5.50 | 0.51 ± 0.04 | 0.58 ± 0.01 | 0.61 ± 0.01 | 0.60 ± 0.02 | 0.24 ± 0.01 | 0.26 ± 0.004 |
| LPC O-16:1 | 480.3461 | [M+H]+ | 5.49 | 0.40 ± 0.06 | 0.55 ± 0.02 | 0.51 ± 0.02 | 0.53 ± 0.08 | 0.24 ± 0.02 | 0.23 ± 0.01 |
| LPC O-18:0 | 552.3666 | [M+HCO2]- | 5.70 | 0.24 ± 0.003 | 0.34 ± 0.01 | 0.39 ± 0.01 | 0.39 ± 0.01 | 0.13 ± 0.004 | 0.11 ± 0.01 |
| LPC O-18:1 | 550.3504 | [M+HCO2]- | 4.94 | 0.004 ± 0.001 | 0.006 ± <0.001 | 0.007 ± 0.001 | 0.006 ± 0.001 | <0.001 | <0.001 |
| LPC O-20:0 | 538.4242 | [M+H]+ | 8.76 | 0.028 ± 0.002 | 0.029 ± 0.002 | 0.033 ± <0.001 | 0.034 ± 0.001 | 0.018 ± 0.001 | 0.015 ± <0.001 |
| LPC O-20:1 | 536.4087 | [M+H]+ | 7.36 | 0.021 ± <0.001 | 0.020 ± 0.001 | 0.024 ± <0.001 | 0.027 ± 0.001 | 0.011 ± 0.001 | 0.007 ± 0.001 |
| LPC O-22:0 | 608.4294 | [M+HCO2]- | 8.77 | 0.011 ± 0.003 | 0.013 ± 0.003 | 0.011 ± 0.005 | 0.030 ± 0.001 | 0.010 ± 0.005 | 0.011 ± 0.002 |
| LPC O-22:1 | 606.4135 | [M+HCO2]- | 7.64 | 0.008 ± <0.001 | 0.008 ± 0.001 | 0.008 ± <0.001 | 0.010 ± 0.001 | 0.004 ± 0.001 | 0.003 ± 0.001 |
| LPC O-22:2 | 562.4243 | [M+H]+ | 7.63 | 0.011 ± 0.001 | 0.011 ± <0.001 | 0.011 ± 0.001 | 0.014 ± <0.001 | 0.006 ± <0.001 | 0.005 ± 0.001 |
| LPC O-24:0 | 636.4605 | [M+HCO2]- | 10.15 | 0.063 ± 0.004 | 0.052 ± 0.003 | 0.063 ± <0.001 | 0.091 ± 0.002 | 0.060 ± 0.001 | 0.063 ± <0.001 |
| LPC O-24:1 | 634.4452 | [M+HCO2]- | 9.01 | 0.018 ± 0.001 | 0.019 ± 0.002 | 0.019 ± 0.001 | 0.026 ± 0.003 | 0.013 ± 0.001 | 0.012 ± <0.001 |
| LPC O-24:2 | 590.4559 | [M+H]+ | 9.01 | 0.025 ± 0.002 | 0.025 ± 0.001 | 0.025 ± 0.001 | 0.034 ± 0.002 | 0.016 ± 0.001 | 0.016 ± 0.001 |
| LPC P-16:0 | 524.3351 | [M+HCO2]- | 5.50 | 0.58 ± 0.02 | 0.72 ± 0.01 | 0.68 ± 0.02 | 0.81 ± 0.02 | 0.37 ± 0.005 | 0.35 ± 0.01 |
| LPC P-16:1 | 478.3297 | [M+H]+ | 5.12 | 0.028 ± 0.006 | 0.027 ± 0.002 | 0.037 ± 0.006 | 0.037 ± 0.002 | 0.015 ± 0.006 | 0.017 ± 0.002 |
| LPC P-18:0 | 552.3666 | [M+HCO2]- | 7.11 | 0.067 ± 0.008 | 0.064 ± 0.006 | 0.068 ± <0.001 | 0.072 ± 0.006 | 0.025 ± 0.006 | 0.018 ± 0.004 |
| LPC P-18:1 | 550.3511 | [M+HCO2]- | 5.51 | 0.045 ± 0.004 | 0.059 ± 0.004 | 0.055 ± 0.005 | 0.062 ± 0.009 | 0.022 ± 0.003 | 0.016 ± 0.002 |
| PC O-16:0/14:0 | 736.5503 | [M+HCO2]-\|[M+H]+ | 13.30 | 0.057 ± 0.002 | 0.055 ± 0.010 | 0.066 ± 0.002 | 0.038 ± 0.013 | 0.085 ± 0.017 | 0.049 ± 0.011 |
| PC O-16:0/15:0 | 706.5760 | [M+H]+ | 14.10 | 0.013 ± <0.001 | 0.011 ± 0.002 | 0.014 ± 0.004 | 0.011 ± 0.003 | 0.030 ± 0.001 | 0.018 ± 0.004 |
| PC O-16:0/16:0 | 764.5807 | [M+HCO2]-\|[M+H]+ | 14.79 | 1.61 ± 0.02 | 1.32 ± 0.02 | 1.59 ± 0.01 | 1.30 ± 0.04 | 2.05 ± 0.08 | 1.70 ± 0.02 |
| PC O-16:0/16:1 | 762.5653 | [M+HCO2]-\|[M+H]+ | 13.63 | 0.24 ± 0.01 | 0.15 ± 0.03 | 0.22 ± 0.01 | 0.15 ± 0.02 | 0.30 ± 0.02 | 0.20 ± 0.04 |
| PC O-16:0/17:0 | 778.5964 | [M+HCO2]- | 15.41 | <0.001 | 0.001 ± 0.002 | <0.001 | <0.001 | <0.001 | <0.001 |
| PC O-16:0/18:1 | 746.6063 | [M+H]+ | 15.78 | 0.052 ± 0.001 | 0.046 ± 0.001 | 0.047 ± 0.003 | 0.045 ± 0.002 | 0.057 ± 0.003 | 0.047 ± 0.001 |
| PC O-16:0/18:2 | 788.5815 | [M+HCO2]-\|[M+H]+ | 13.75 | 2.35 ± 0.04 | 2.14 ± 0.44 | 2.65 ± 0.06 | 1.82 ± 0.52 | 2.78 ± 0.46 | 1.36 ± 0.32 |
| PC O-16:0/18:3 | 742.5764 | [M+H]+ | 12.59 | 0.009 ± 0.004 | 0.009 ± 0.002 | 0.015 ± 0.010 | 0.016 ± 0.001 | 0.042 ± 0.008 | 0.010 ± 0.004 |
| PC O-16:0/20:3 | 814.5961 | [M+HCO2]-\|[M+H]+ | 14.11 | 0.55 ± 0.04 | 0.39 ± 0.09 | 0.61 ± 0.02 | 0.29 ± 0.07 | 0.68 ± 0.12 | 0.26 ± 0.06 |
| PC O-16:0/20:4 | 812.5812 | [M+HCO2]-\|[M+H]+ | 13.42 | 6.52 ± 0.14 | 5.84 ± 0.90 | 6.54 ± 0.05 | 4.72 ± 0.84 | 7.70 ± 0.70 | 4.75 ± 0.68 |
| PC O-16:0/20:5 | 810.5660 | [M+HCO2]-\|[M+H]+ | 12.31 | 0.13 ± 0.02 | 0.25 ± 0.02 | 0.20 ± 0.01 | 0.85 ± 0.14 | 0.26 ± 0.01 | 1.48 ± 0.15 |
| PC O-16:0/22:4 | 840.6114 | [M+HCO2]-\|[M+H]+ | 14.48 | 0.80 ± 0.002 | 0.62 ± 0.06 | 0.77 ± 0.02 | 0.44 ± 0.03 | 0.92 ± 0.08 | 0.39 ± 0.02 |
| PC O-16:0/22:6 | 836.5798 | [M+HCO2]-\|[M+H]+ | 12.90 | 0.42 ± 0.01 | 0.64 ± 0.05 | 0.66 ± 0.02 | 0.85 ± 0.04 | 0.75 ± 0.03 | 0.93 ± 0.09 |
| PC O-16:1/14:0 | 734.5335 | [M+HCO2]- | 12.97 | 0.044 ± 0.014 | 0.047 ± 0.005 | 0.032 ± 0.006 | <0.001 | 0.036 ± 0.005 | 0.005 ± 0.002 |
| PC O-16:1/16:0 | 762.5643 | [M+HCO2]- | 14.60 | 1.04 ± 0.01 | 0.94 ± 0.03 | 0.94 ± 0.02 | 1.03 ± 0.05 | 1.27 ± 0.04 | 1.42 ± 0.02 |
| PC O-16:1/16:1 | 760.5491 | [M+HCO2]- | 13.30 | 0.14 ± 0.01 | 0.10 ± 0.02 | 0.11 ± 0.01 | 0.12 ± 0.02 | 0.15 ± 0.02 | 0.16 ± 0.04 |
| PC O-16:1/17:0 | 776.5792 | [M+HCO2]-\|[M+H]+ | 14.99 | <0.001 | <0.001 | <0.001 | <0.001 | <0.001 | <0.001 |
| PC O-16:1/17:1 | 774.5636 | [M+HCO2]- | 13.80 | <0.001 | <0.001 | <0.001 | 0.002 ± 0.003 | <0.001 | 0.003 ± 0.006 |
| PC O-16:1/18:0 | 746.6065 | [M+H]+ | 14.84 | 2.84 ± 0.06 | 2.10 ± 0.22 | 2.90 ± 0.10 | 2.55 ± 0.22 | 3.76 ± 0.28 | 2.63 ± 0.15 |
| PC O-16:1/18:1 | 788.5808 | [M+HCO2]- | 14.67 | 1.59 ± 0.04 | 1.53 ± 0.14 | 1.51 ± 0.03 | 1.75 ± 0.29 | 2.07 ± 0.14 | 1.75 ± 0.21 |
| PC O-16:1/18:2 | 786.5653 | [M+HCO2]- | 13.49 | 3.62 ± 0.06 | 3.63 ± 0.90 | 3.71 ± 0.03 | 3.35 ± 0.96 | 4.86 ± 0.97 | 2.28 ± 0.56 |
| PC O-16:1/20:3 | 812.5800 | [M+HCO2]- | 13.84 | 0.78 ± 0.04 | 0.62 ± 0.12 | 0.81 ± 0.02 | 0.53 ± 0.10 | 1.09 ± 0.12 | 0.44 ± 0.09 |
| PC O-16:1/20:4 | 810.5653 | [M+HCO2]- | 13.14 | 3.81 ± 0.19 | 3.56 ± 0.82 | 3.79 ± 0.06 | 2.96 ± 0.91 | 4.15 ± 1.01 | 2.69 ± 0.75 |
| PC O-16:1/20:5 | 808.5494 | [M+HCO2]- | 12.09 | 0.14 ± 0.004 | 0.24 ± 0.01 | 0.19 ± 0.01 | 0.92 ± 0.02 | 0.30 ± 0.01 | 1.46 ± 0.02 |
| PC O-16:1/22:4 | 838.5956 | [M+HCO2]- | 14.33 | 0.36 ± 0.04 | 0.31 ± 0.11 | 0.26 ± 0.01 | 0.30 ± 0.15 | 0.59 ± 0.15 | 0.33 ± 0.10 |
| PC O-16:1/22:5 | 836.5807 | [M+HCO2]- | 13.70 | 0.29 ± 0.01 | 0.22 ± 0.01 | 0.25 ± 0.01 | 0.39 ± 0.03 | 0.32 ± 0.003 | 0.48 ± 0.01 |
| PC O-16:1/22:6 | 834.5640 | [M+HCO2]- | 12.61 | 0.33 ± 0.01 | 0.46 ± 0.05 | 0.47 ± 0.005 | 0.79 ± 0.12 | 0.59 ± 0.05 | 0.87 ± 0.08 |
| PC O-18:0/16:0 | 792.6128 | [M+HCO2]-\|[M+H]+ | 15.93 | 0.27 ± 0.01 | 0.21 ± 0.02 | 0.32 ± 0.004 | 0.18 ± 0.02 | 0.28 ± 0.003 | 0.17 ± 0.003 |
| PC O-18:0/18:2 | 816.6134 | [M+HCO2]-\|[M+H]+ | 15.12 | 1.16 ± 0.003 | 0.80 ± 0.12 | 1.24 ± 0.01 | 0.78 ± 0.11 | 1.32 ± 0.13 | 0.53 ± 0.08 |
| PC O-18:0/20:3 | 842.6282 | [M+HCO2]-\|[M+H]+ | 15.38 | 0.39 ± 0.004 | 0.27 ± 0.01 | 0.45 ± 0.01 | 0.22 ± 0.01 | 0.61 ± 0.03 | 0.20 ± 0.006 |
| PC O-18:0/20:4 | 840.6118 | [M+HCO2]-\|[M+H]+ | 14.87 | 3.77 ± 0.04 | 2.88 ± 0.29 | 3.87 ± 0.05 | 2.19 ± 0.27 | 4.28 ± 0.16 | 1.98 ± 0.16 |
| PC O-18:0/20:5 | 838.5962 | [M+HCO2]- | 13.97 | 0.30 ± 0.02 | 0.25 ± 0.03 | 0.29 ± 0.004 | 0.40 ± 0.10 | 0.42 ± 0.02 | 0.43 ± 0.14 |
| PC O-18:0/22:4 | 868.6427 | [M+HCO2]-\|[M+H]+ | 15.67 | 0.32 ± 0.03 | 0.12 ± 0.04 | 0.19 ± 0.02 | 0.040 ± 0.016 | 0.24 ± 0.02 | 0.038 ± 0.019 |
| PC O-18:0/22:5 | 822.6379 | [M+H]+ | 15.26 | 0.13 ± 0.004 | 0.092 ± 0.002 | 0.11 ± 0.005 | 0.14 ± 0.01 | 0.16 ± 0.002 | 0.15 ± 0.01 |
| PC O-18:0/22:6 | 864.6113 | [M+HCO2]-\|[M+H]+ | 14.44 | 0.35 ± 0.01 | 0.42 ± 0.03 | 0.50 ± 0.01 | 0.59 ± 0.02 | 0.62 ± 0.04 | 0.59 ± 0.02 |
| PC O-18:1/16:0 | 790.5953 | [M+HCO2]- | 14.84 | 3.11 ± 0.005 | 2.46 ± 0.27 | 3.24 ± 0.07 | 2.97 ± 0.46 | 4.14 ± 0.25 | 2.92 ± 0.27 |
| PC O-18:1/18:1 | 816.6118 | [M+HCO2]- | 15.83 | 0.32 ± 0.001 | 0.31 ± 0.01 | 0.30 ± 0.01 | 0.36 ± 0.01 | 0.41 ± 0.02 | 0.30 ± 0.002 |
| PC O-18:1/18:2 | 814.5970 | [M+HCO2]- | 14.92 | 1.31 ± 0.01 | 1.43 ± 0.05 | 1.31 ± 0.02 | 1.41 ± 0.09 | 1.75 ± 0.07 | 0.87 ± 0.06 |
| PC O-18:1/20:2 | 842.6279 | [M+HCO2]- | 15.96 | 0.043 ± 0.007 | 0.036 ± 0.006 | 0.019 ± 0.004 | 0.027 ± 0.005 | 0.038 ± 0.012 | 0.008 ± 0.003 |
| PC O-18:1/20:3 | 840.6107 | [M+HCO2]- | 15.12 | 0.35 ± 0.01 | 0.24 ± 0.05 | 0.33 ± 0.02 | 0.18 ± 0.02 | 0.40 ± 0.04 | 0.14 ± 0.03 |
| PC O-18:1/20:4 | 838.5966 | [M+HCO2]-\|[M+H]+ | 13.47 | 5.30 ± 0.28 | 4.47 ± 0.62 | 5.89 ± 0.03 | 4.37 ± 0.74 | 6.04 ± 0.55 | 3.69 ± 0.50 |
| PC O-18:1/20:5 | 836.5793 | [M+HCO2]- | 12.43 | <0.001 | 0.004 ± 0.007 | <0.001 | 0.20 ± 0.03 | 0.006 ± 0.010 | 0.10 ± 0.10 |
| PC O-18:1/22:4 | 866.6257 | [M+HCO2]-\|[M+H]+ | 14.52 | 0.36 ± 0.02 | 0.26 ± 0.04 | 0.38 ± 0.01 | 0.28 ± 0.05 | 0.36 ± 0.07 | 0.22 ± 0.05 |
| PC O-18:1/22:5 | 864.6120 | [M+HCO2]-\|[M+H]+ | 13.56 | 0.34 ± 0.02 | 0.36 ± 0.02 | 0.48 ± 0.02 | 0.56 ± 0.04 | 0.49 ± 0.04 | 0.50 ± 0.02 |
| PC O-18:1/22:6 | 862.5961 | [M+HCO2]-\|[M+H]+ | 12.96 | 0.22 ± 0.03 | 0.39 ± 0.07 | 0.46 ± 0.002 | 0.72 ± 0.15 | 0.49 ± 0.08 | 0.58 ± 0.11 |
| PC O-20:0/16:0 | 820.6444 | [M+HCO2]-\|[M+H]+ | 16.93 | 0.032 ± 0.002 | 0.073 ± 0.010 | 0.070 ± 0.008 | 0.079 ± 0.004 | 0.083 ± 0.007 | 0.080 ± 0.011 |
| PC O-20:0/20:4 | 868.6432 | [M+HCO2]-\|[M+H]+ | 15.97 | 0.67 ± 0.02 | 0.56 ± 0.06 | 0.70 ± 0.01 | 0.49 ± 0.06 | 0.87 ± 0.04 | 0.38 ± 0.03 |
| PC O-20:0/20:5 | 866.6268 | [M+HCO2]- | 15.25 | 0.17 ± 0.01 | 0.11 ± 0.003 | 0.13 ± 0.005 | 0.17 ± 0.01 | 0.18 ± 0.004 | 0.18 ± 0.01 |
| PC O-20:0/22:4 | 896.6734 | [M+HCO2]- | 16.59 | 0.020 ± 0.003 | 0.007 ± 0.003 | 0.013 ± <0.001 | 0.005 ± 0.002 | 0.011 ± 0.008 | 0.003 ± 0.001 |
| PC O-20:1/20:3 | 824.6538 | [M+H]+ | 15.38 | 0.16 ± 0.01 | 0.11 ± 0.01 | 0.15 ± 0.004 | 0.11 ± 0.01 | 0.24 ± 0.01 | 0.092 ± 0.014 |
| PC O-20:1/20:4 | 866.6271 | [M+HCO2]-\|[M+H]+ | 14.84 | 1.08 ± 0.05 | 0.86 ± 0.03 | 1.03 ± 0.01 | 1.00 ± 0.03 | 1.35 ± 0.03 | 0.93 ± 0.05 |
| PC O-20:1/22:6 | 890.6292 | [M+HCO2]- | 14.42 | 0.031 ± 0.006 | 0.043 ± 0.011 | 0.049 ± 0.005 | 0.11 ± 0.02 | 0.098 ± 0.021 | 0.099 ± 0.011 |
| PC O-22:0/16:0 | 848.6750 | [M+HCO2]-\|[M+H]+ | 17.66 | 0.027 ± 0.002 | 0.035 ± 0.002 | 0.042 ± 0.003 | 0.037 ± 0.002 | 0.040 ± 0.009 | 0.029 ± 0.001 |
| PC O-22:0/18:2 | 828.6859 | [M+H]+ | 17.05 | 0.046 ± 0.007 | 0.048 ± 0.003 | 0.062 ± 0.006 | 0.061 ± 0.011 | 0.082 ± 0.004 | 0.037 ± 0.005 |
| PC O-22:0/18:3 | 826.6705 | [M+H]+ | 16.87 | 0.019 ± 0.003 | 0.019 ± 0.004 | 0.026 ± 0.001 | 0.020 ± 0.002 | 0.022 ± 0.004 | 0.018 ± 0.003 |
| PC O-22:0/20:3 | 898.6897 | [M+HCO2]- | 17.21 | 0.036 ± 0.004 | 0.029 ± 0.004 | 0.039 ± 0.009 | 0.028 ± 0.003 | 0.056 ± 0.012 | 0.024 ± 0.002 |
| PC O-22:0/20:4 | 896.6744 | [M+HCO2]-\|[M+H]+ | 16.88 | 0.39 ± 0.004 | 0.42 ± 0.005 | 0.46 ± 0.02 | 0.38 ± 0.03 | 0.60 ± 0.02 | 0.32 ± 0.004 |
| PC O-22:0/22:4 | 924.7051 | [M+HCO2]- | 17.37 | 0.019 ± 0.002 | 0.010 ± 0.005 | 0.014 ± 0.002 | 0.004 ± <0.001 | 0.025 ± 0.010 | 0.008 ± 0.006 |
| PC O-22:1/18:2 | 870.6585 | [M+HCO2]-\|[M+H]+ | 16.12 | 0.27 ± 0.01 | 0.21 ± 0.02 | 0.28 ± 0.01 | 0.27 ± 0.02 | 0.43 ± 0.01 | 0.22 ± 0.03 |
| PC O-22:1/20:4 | 894.6589 | [M+HCO2]-\|[M+H]+ | 15.90 | 0.96 ± 0.05 | 0.81 ± 0.05 | 0.88 ± 0.002 | 0.93 ± 0.05 | 1.39 ± 0.03 | 0.83 ± 0.02 |
| PC O-22:1/22:4 | 922.6901 | [M+HCO2]- | 16.48 | 0.060 ± 0.005 | 0.050 ± 0.007 | 0.070 ± 0.004 | 0.050 ± 0.009 | 0.10 ± 0.01 | 0.046 ± 0.006 |
| PC O-22:1/22:6 | 918.6590 | [M+HCO2]-\|[M+H]+ | 15.58 | 0.090 ± 0.002 | 0.12 ± 0.02 | 0.12 ± 0.003 | 0.28 ± 0.02 | 0.23 ± 0.02 | 0.26 ± 0.02 |
| PC O-24:0/16:0 | 876.7061 | [M+HCO2]- | 18.50 | 0.004 ± 0.002 | 0.005 ± 0.003 | 0.004 ± 0.001 | 0.007 ± 0.003 | 0.005 ± 0.001 | 0.012 ± 0.007 |
| PC O-24:0/18:1 | 902.7215 | [M+HCO2]-\|[M+H]+ | 18.54 | 0.005 ± 0.002 | 0.019 ± 0.003 | 0.023 ± 0.002 | 0.064 ± 0.014 | 0.027 ± 0.002 | 0.20 ± 0.04 |
| PC O-24:0/18:2 | 900.7059 | [M+HCO2]- | 17.85 | 0.053 ± 0.003 | 0.059 ± 0.009 | 0.079 ± 0.003 | 0.076 ± 0.004 | 0.071 ± 0.013 | 0.054 ± 0.003 |
| PC O-24:0/20:4 | 924.7054 | [M+HCO2]-\|[M+H]+ | 17.66 | 0.10 ± 0.002 | 0.12 ± 0.01 | 0.11 ± 0.001 | 0.11 ± 0.01 | 0.15 ± 0.01 | 0.10 ± 0.01 |
| PC O-24:0/22:5 | 906.7325 | [M+H]+ | 17.56 | 0.045 ± 0.002 | 0.038 ± 0.005 | 0.050 ± 0.002 | 0.055 ± 0.001 | 0.057 ± 0.007 | 0.059 ± 0.002 |
| PC O-24:0/22:6 | 948.7067 | [M+HCO2]-\|[M+H]+ | 17.38 | 0.005 ± 0.002 | 0.016 ± 0.002 | 0.015 ± 0.007 | 0.043 ± 0.004 | 0.041 ± 0.004 | 0.046 ± 0.006 |
| PC O-24:1/18:1 | 900.7061 | [M+HCO2]- | 17.60 | 0.073 ± 0.003 | 0.072 ± 0.009 | 0.081 ± 0.009 | 0.12 ± 0.004 | 0.13 ± 0.01 | 0.13 ± 0.006 |
| PC O-24:1/18:2 | 898.6883 | [M+HCO2]-\|[M+H]+ | 16.96 | 0.30 ± 0.003 | 0.27 ± 0.02 | 0.35 ± 0.01 | 0.37 ± 0.05 | 0.54 ± 0.04 | 0.30 ± 0.02 |
| PC O-24:1/20:3 | 924.7055 | [M+HCO2]-\|[M+H]+ | 17.13 | 0.11 ± 0.01 | 0.085 ± 0.004 | 0.14 ± 0.004 | 0.12 ± 0.01 | 0.28 ± 0.01 | 0.12 ± 0.01 |
| PC O-24:1/20:4 | 922.6884 | [M+HCO2]-\|[M+H]+ | 16.78 | 1.10 ± 0.06 | 1.16 ± 0.02 | 1.26 ± 0.02 | 1.33 ± 0.07 | 2.04 ± 0.08 | 1.36 ± 0.02 |
| PC O-24:1/20:5 | 876.6843 | [M+H]+ | 17.03 | 0.003 ± 0.001 | 0.004 ± 0.001 | 0.009 ± 0.005 | 0.005 ± 0.002 | 0.011 ± 0.007 | 0.010 ± 0.007 |
| PC O-24:1/22:4 | 950.7218 | [M+HCO2]- | 17.56 | 0.063 ± 0.002 | 0.060 ± 0.004 | 0.063 ± 0.008 | 0.078 ± 0.003 | 0.087 ± 0.006 | 0.085 ± 0.002 |
| PC O-24:1/22:5 | 948.7058 | [M+HCO2]-\|[M+H]+ | 16.81 | 0.12 ± 0.003 | 0.13 ± 0.003 | 0.13 ± 0.01 | 0.23 ± 0.01 | 0.19 ± 0.01 | 0.28 ± 0.003 |
| PC O-24:1/22:6 | 946.6916 | [M+HCO2]-\|[M+H]+ | 16.50 | 0.11 ± 0.01 | 0.17 ± 0.006 | 0.18 ± 0.01 | 0.42 ± 0.004 | 0.29 ± 0.01 | 0.42 ± 0.01 |
| PC O-24:2/18:2 | 896.6738 | [M+HCO2]-\|[M+H]+ | 16.21 | 0.20 ± 0.01 | 0.17 ± 0.01 | 0.21 ± 0.01 | 0.21 ± 0.01 | 0.36 ± 0.02 | 0.18 ± 0.006 |
| PC O-24:2/20:3 | 878.6989 | [M+H]+ | 16.57 | 0.066 ± 0.003 | 0.051 ± 0.002 | 0.071 ± 0.002 | 0.066 ± 0.005 | 0.11 ± 0.005 | 0.064 ± 0.002 |
| PC O-24:2/20:4 | 920.6731 | [M+HCO2]-\|[M+H]+ | 16.00 | 0.72 ± 0.03 | 0.76 ± 0.02 | 0.76 ± 0.02 | 1.05 ± 0.03 | 1.07 ± 0.03 | 1.19 ± 0.01 |
| PC O-24:2/20:5 | 918.6585 | [M+HCO2]- | 15.19 | 0.037 ± 0.005 | 0.038 ± 0.008 | 0.039 ± 0.002 | 0.12 ± 0.02 | 0.063 ± 0.011 | 0.18 ± 0.006 |
| PC O-24:2/22:5 | 946.6903 | [M+HCO2]-\|[M+H]+ | 15.98 | 0.035 ± 0.001 | 0.037 ± 0.001 | 0.037 ± 0.002 | 0.090 ± 0.003 | 0.060 ± 0.003 | 0.11 ± 0.01 |
| PC O-24:2/22:6 | 944.6755 | [M+HCO2]-\|[M+H]+ | 15.66 | 0.081 ± 0.002 | 0.099 ± 0.006 | 0.11 ± 0.01 | 0.24 ± 0.01 | 0.14 ± 0.01 | 0.16 ± 0.01 |
| PC P-16:0/14:0 | 690.5453 | [M+H]+ | 13.04 | 0.023 ± 0.003 | 0.036 ± 0.007 | 0.030 ± 0.002 | 0.032 ± 0.002 | 0.042 ± 0.009 | 0.044 ± 0.004 |
| PC P-16:0/15:0 | 704.5605 | [M+H]+ | 13.87 | 0.010 ± 0.004 | 0.011 ± <0.001 | 0.014 ± 0.002 | 0.013 ± 0.001 | 0.024 ± 0.001 | 0.019 ± 0.002 |
| PC P-16:0/16:0 | 718.5744 | [M+H]+ | 14.60 | 0.85 ± 0.01 | 0.75 ± 0.02 | 0.71 ± 0.01 | 0.83 ± 0.01 | 1.05 ± 0.01 | 1.19 ± 0.01 |
| PC P-16:0/16:1 | 716.5596 | [M+H]+ | 13.33 | 0.047 ± 0.007 | 0.044 ± 0.002 | 0.046 ± 0.005 | 0.054 ± 0.004 | 0.066 ± 0.004 | 0.082 ± 0.007 |
| PC P-16:0/17:0 | 732.5921 | [M+H]+ | 15.13 | 0.017 ± 0.001 | 0.011 ± 0.004 | 0.018 ± 0.002 | 0.011 ± 0.004 | 0.021 ± 0.005 | 0.013 ± 0.001 |
| PC P-16:0/18:1 | 744.5908 | [M+H]+ | 14.65 | 0.28 ± 0.005 | 0.20 ± 0.03 | 0.068 ± 0.012 | 0.24 ± 0.11 | 0.21 ± 0.04 | 1.76 ± 0.07 |
| PC P-16:0/18:2 | 742.5755 | [M+H]+ | 13.49 | 3.65 ± 0.01 | 3.98 ± 0.30 | 3.71 ± 0.08 | 3.66 ± 0.27 | 5.77 ± 0.21 | 2.78 ± 0.14 |
| PC P-16:0/20:3 | 768.5915 | [M+H]+ | 13.84 | 0.90 ± 0.05 | 0.73 ± 0.14 | 0.90 ± 0.04 | 0.58 ± 0.10 | 1.26 ± 0.14 | 0.50 ± 0.08 |
| PC P-16:0/20:4 | 766.5750 | [M+H]+ | 13.14 | 3.98 ± 0.22 | 4.33 ± 0.03 | 3.97 ± 0.02 | 3.80 ± 0.004 | 5.57 ± 0.04 | 3.71 ± 0.005 |
| PC P-16:0/20:5 | 764.5588 | [M+H]+ | 12.09 | 0.17 ± 0.002 | 0.24 ± 0.01 | 0.22 ± 0.01 | 0.92 ± <0.001 | 0.30 ± 0.01 | 1.54 ± 0.02 |
| PC P-16:0/22:1 | 800.6539 | [M+H]+ | 16.72 | 0.012 ± 0.001 | 0.012 ± 0.002 | 0.011 ± 0.001 | 0.016 ± 0.002 | 0.016 ± 0.002 | 0.009 ± 0.002 |
| PC P-16:0/22:4 | 794.6054 | [M+H]+ | 14.21 | 0.32 ± 0.03 | 0.23 ± 0.01 | 0.27 ± 0.01 | 0.17 ± 0.03 | 0.32 ± 0.03 | 0.17 ± 0.02 |
| PC P-16:0/22:5 | 792.5907 | [M+H]+ | 13.70 | 0.35 ± 0.01 | 0.26 ± 0.01 | 0.29 ± 0.002 | 0.44 ± 0.01 | 0.38 ± 0.01 | 0.56 ± 0.01 |
| PC P-16:0/22:6 | 790.5732 | [M+H]+ | 12.60 | 0.32 ± 0.01 | 0.49 ± 0.004 | 0.45 ± 0.005 | 0.80 ± 0.01 | 0.65 ± 0.02 | 0.90 ± 0.02 |
| PC P-16:1/18:1 | 786.5653 | [M+HCO2]- | 14.97 | 0.049 ± 0.001 | 0.046 ± 0.008 | 0.059 ± 0.006 | 0.039 ± 0.005 | 0.064 ± 0.006 | 0.025 ± 0.004 |
| PC P-16:1/18:2 | 784.5492 | [M+HCO2]-\|[M+H]+ | 12.32 | 0.021 ± 0.003 | 0.024 ± 0.005 | 0.029 ± 0.004 | 0.021 ± 0.004 | 0.048 ± 0.008 | 0.010 ± 0.003 |
| PC P-16:1/20:4 | 808.5489 | [M+HCO2]- | 11.77 | 0.004 ± 0.002 | 0.003 ± 0.001 | 0.009 ± 0.001 | 0.004 ± 0.001 | 0.004 ± 0.002 | 0.002 ± 0.002 |
| PC P-16:1/20:5 | 762.5444 | [M+H]+ | 11.09 | 0.001 ± <0.001 | 0.001 ± <0.001 | 0.001 ± <0.001 | 0.001 ± <0.001 | 0.001 ± <0.001 | 0.003 ± 0.001 |
| PC P-16:1/22:6 | 832.5496 | [M+HCO2]- | 12.29 | 0.030 ± 0.004 | 0.031 ± 0.010 | 0.047 ± 0.016 | 0.039 ± 0.015 | 0.041 ± 0.014 | 0.047 ± 0.014 |
| PC P-18:0/18:1 | 772.6224 | [M+H]+ | 15.83 | 0.22 ± 0.01 | 0.20 ± 0.01 | 0.21 ± 0.005 | 0.25 ± 0.005 | 0.29 ± 0.01 | 0.20 ± 0.01 |
| PC P-18:0/18:2 | 770.6062 | [M+H]+ | 14.92 | 1.08 ± 0.02 | 1.07 ± 0.08 | 1.06 ± 0.03 | 1.03 ± 0.04 | 1.40 ± 0.11 | 0.65 ± 0.07 |
| PC P-18:0/20:4 | 794.6062 | [M+H]+ | 14.64 | 1.32 ± 0.02 | 1.19 ± 0.02 | 1.25 ± 0.01 | 1.10 ± 0.01 | 1.62 ± 0.02 | 0.92 ± 0.01 |
| PC P-18:0/22:5 | 820.6219 | [M+H]+ | 15.08 | 0.052 ± 0.002 | 0.036 ± 0.005 | 0.043 ± 0.003 | 0.047 ± 0.004 | 0.062 ± 0.005 | 0.044 ± 0.009 |
| PC P-18:0/22:6 | 818.6073 | [M+H]+ | 14.18 | 0.14 ± 0.005 | 0.19 ± 0.002 | 0.18 ± 0.001 | 0.29 ± 0.01 | 0.28 ± 0.005 | 0.29 ± 0.001 |
| PC P-18:1/20:4 | 836.5795 | [M+HCO2]-\|[M+H]+ | 13.22 | 1.47 ± 0.10 | 1.43 ± 0.01 | 1.59 ± 0.03 | 1.64 ± 0.003 | 1.80 ± 0.03 | 1.64 ± 0.06 |
| PC P-18:1/20:5 | 834.5653 | [M+HCO2]-\|[M+H]+ | 12.17 | 0.057 ± 0.005 | 0.060 ± 0.012 | 0.068 ± 0.006 | 0.21 ± 0.02 | 0.070 ± 0.019 | 0.27 ± 0.03 |
| PC P-18:1/22:5 | 862.5959 | [M+HCO2]- | 13.25 | 0.099 ± 0.016 | 0.081 ± 0.016 | 0.083 ± 0.008 | 0.14 ± 0.02 | 0.084 ± 0.005 | 0.13 ± 0.02 |
| PC P-18:1/22:6 | 860.5805 | [M+HCO2]-\|[M+H]+ | 12.70 | 0.051 ± 0.010 | 0.081 ± 0.012 | 0.086 ± 0.005 | 0.15 ± 0.03 | 0.12 ± 0.02 | 0.15 ± 0.01 |
| PC P-20:0/18:2 | 798.6379 | [M+H]+ | 15.94 | 0.078 ± 0.001 | 0.060 ± 0.005 | 0.073 ± 0.002 | 0.067 ± 0.004 | 0.089 ± 0.010 | 0.047 ± 0.002 |
| PC P-20:0/22:6 | 846.6386 | [M+H]+ | 14.44 | 0.048 ± 0.007 | 0.058 ± 0.009 | 0.069 ± 0.004 | 0.14 ± 0.01 | 0.12 ± 0.01 | 0.12 ± 0.01 |
| PC P-20:1/18:2 | 840.6125 | [M+HCO2]- | 16.18 | <0.001 | <0.001 | <0.001 | <0.001 | <0.001 | <0.001 |
| PC P-20:1/20:4 | 864.6110 | [M+HCO2]-\|[M+H]+ | 13.74 | 0.025 ± 0.002 | 0.012 ± 0.011 | 0.030 ± 0.008 | 0.006 ± 0.010 | 0.008 ± 0.014 | 0.006 ± 0.010 |
| PC P-20:1/20:5 | 818.6045 | [M+H]+ | 12.53 | 0.018 ± 0.005 | 0.022 ± 0.002 | 0.024 ± <0.001 | 0.056 ± 0.004 | 0.031 ± 0.004 | 0.069 ± 0.004 |
| PC P-20:1/22:5 | 890.6286 | [M+HCO2]- | 14.77 | 0.089 ± 0.009 | 0.087 ± 0.007 | 0.086 ± 0.008 | 0.13 ± 0.01 | 0.11 ± 0.004 | 0.13 ± 0.01 |
| PC P-20:1/22:6 | 844.6212 | [M+H]+ | 13.14 | 0.022 ± 0.002 | 0.033 ± 0.003 | 0.037 ± 0.003 | 0.055 ± 0.002 | 0.042 ± 0.001 | 0.036 ± 0.004 |
| PC P-22:0/18:1 | 828.6863 | [M+H]+ | 17.41 | 0.008 ± 0.002 | 0.008 ± 0.004 | 0.010 ± 0.002 | 0.009 ± 0.002 | 0.015 ± 0.005 | 0.016 ± 0.001 |
| PC P-22:1/18:1 | 870.6605 | [M+HCO2]- | 16.68 | 0.032 ± 0.002 | 0.024 ± 0.003 | 0.048 ± 0.002 | 0.033 ± 0.006 | 0.039 ± 0.008 | 0.030 ± 0.007 |
| PC P-22:1/18:2 | 868.6434 | [M+HCO2]-\|[M+H]+ | 15.20 | 0.075 ± 0.002 | 0.069 ± 0.007 | 0.080 ± 0.002 | 0.094 ± 0.007 | 0.14 ± 0.02 | 0.072 ± 0.010 |
| PC P-22:1/20:3 | 894.6575 | [M+HCO2]-\|[M+H]+ | 15.55 | 0.029 ± 0.005 | 0.026 ± 0.008 | 0.023 ± 0.002 | 0.034 ± 0.004 | 0.074 ± 0.009 | 0.020 ± 0.005 |
| PC P-22:1/20:4 | 892.6415 | [M+HCO2]-\|[M+H]+ | 14.97 | 0.42 ± 0.03 | 0.40 ± 0.006 | 0.41 ± 0.001 | 0.61 ± 0.01 | 0.59 ± 0.01 | 0.65 ± 0.01 |
| PC P-22:1/20:5 | 890.6288 | [M+HCO2]- | 13.92 | 0.001 ± 0.001 | 0.001 ± 0.001 | <0.001 | <0.001 | 0.003 ± 0.005 | <0.001 |
| PC P-22:1/22:5 | 918.6594 | [M+HCO2]- | 14.96 | 0.009 ± 0.002 | 0.013 ± 0.004 | 0.011 ± 0.001 | 0.030 ± 0.004 | 0.026 ± 0.005 | 0.039 ± 0.008 |
| PC P-22:1/22:6 | 916.6422 | [M+HCO2]-\|[M+H]+ | 14.54 | 0.017 ± 0.002 | 0.021 ± 0.006 | 0.024 ± 0.010 | 0.071 ± 0.010 | 0.048 ± 0.010 | 0.052 ± 0.004 |
| PC P-24:0/18:1 | 856.7161 | [M+H]+ | 17.60 | 0.048 ± 0.002 | 0.044 ± 0.003 | 0.056 ± 0.003 | 0.067 ± 0.006 | 0.086 ± 0.002 | 0.087 ± 0.003 |
| PC P-24:0/22:4 | 906.7322 | [M+H]+ | 17.32 | 0.052 ± 0.002 | 0.041 ± 0.001 | 0.055 ± 0.002 | 0.043 ± 0.007 | 0.085 ± 0.002 | 0.046 ± 0.005 |
| PC P-24:0/22:6 | 902.6999 | [M+H]+ | 16.32 | 0.009 ± 0.002 | 0.008 ± 0.001 | 0.008 ± <0.001 | 0.017 ± 0.003 | 0.015 ± 0.002 | 0.029 ± 0.003 |
| PC P-24:1/18:1 | 854.7017 | [M+H]+ | 17.48 | 0.009 ± 0.001 | 0.007 ± 0.002 | 0.010 ± 0.001 | 0.013 ± 0.003 | 0.011 ± 0.003 | 0.011 ± 0.003 |
| PC P-24:1/20:4 | 876.6854 | [M+H]+ | 16.63 | 0.14 ± 0.003 | 0.15 ± 0.02 | 0.20 ± 0.01 | 0.25 ± 0.01 | 0.25 ± 0.02 | 0.23 ± 0.01 |
| PC P-24:1/20:5 | 874.6681 | [M+H]+ | 16.23 | 0.001 ± <0.001 | 0.002 ± 0.002 | 0.001 ± 0.001 | 0.002 ± 0.001 | 0.002 ± 0.001 | 0.004 ± 0.001 |
| PC P-24:1/22:6 | 944.6772 | [M+HCO2]-\|[M+H]+ | 16.34 | 0.002 ± 0.001 | 0.008 ± 0.004 | 0.006 ± <0.001 | 0.036 ± 0.005 | 0.017 ± 0.005 | 0.025 ± 0.007 |
| PC P-24:2/20:4 | 918.6596 | [M+HCO2]-\|[M+H]+ | 15.83 | 0.13 ± 0.01 | 0.13 ± 0.01 | 0.15 ± 0.004 | 0.21 ± 0.01 | 0.16 ± 0.02 | 0.21 ± 0.004 |
| PC P-24:2/20:5 | 872.6547 | [M+H]+ | 14.99 | 0.009 ± 0.001 | 0.009 ± 0.001 | 0.012 ± 0.003 | 0.027 ± 0.003 | 0.021 ± 0.002 | 0.038 ± 0.004 |
| PC P-24:2/22:6 | 942.6604 | [M+HCO2]-\|[M+H]+ | 15.49 | 0.004 ± 0.001 | 0.010 ± 0.002 | 0.007 ± 0.003 | 0.014 ± 0.003 | 0.008 ± 0.001 | 0.010 ± 0.003 |
| PE O-16:0/18:1 | 702.5449 | [M-H]- | 15.21 | 0.15 ± 0.02 | 0.12 ± 0.06 | 0.19 ± 0.03 | 0.11 ± 0.05 | 0.11 ± 0.03 | 0.075 ± 0.051 |
| PE O-16:0/18:2 | 700.5280 | [M-H]- | 14.18 | 0.096 ± 0.003 | 0.079 ± 0.080 | 0.090 ± 0.024 | 0.032 ± 0.009 | 0.056 ± 0.063 | 0.023 ± 0.020 |
| PE O-16:0/22:4 | 754.5755 | [M+H]+ | 14.90 | 0.061 ± 0.002 | 0.038 ± 0.006 | 0.031 ± 0.006 | 0.015 ± 0.002 | 0.036 ± 0.006 | 0.010 ± 0.001 |
| PE O-18:0/18:1 | 730.5753 | [M-H]- | 14.87 | 0.004 ± 0.002 | 0.001 ± 0.001 | 0.002 ± 0.001 | 0.001 ± 0.001 | 0.009 ± 0.009 | 0.005 ± 0.004 |
| PE O-18:0/18:2 | 728.5590 | [M-H]- | 13.78 | 0.011 ± 0.002 | 0.012 ± 0.002 | 0.011 ± 0.005 | 0.004 ± 0.003 | 0.007 ± 0.003 | 0.006 ± 0.006 |
| PE O-18:0/20:4 | 752.5592 | [M-H]- | 15.19 | 1.55 ± 0.04 | 1.05 ± 0.57 | 1.39 ± 0.21 | 0.66 ± 0.18 | 1.34 ± 0.54 | 0.48 ± 0.24 |
| PE O-18:0/22:4 | 780.5916 | [M-H]- | 16.08 | 0.044 ± 0.012 | 0.22 ± 0.17 | 0.12 ± 0.04 | 0.22 ± 0.16 | 0.25 ± 0.13 | 0.16 ± 0.12 |
| PE O-18:0/22:5 | 778.5764 | [M-H]-\|[M+H]+ | 15.25 | 0.33 ± 0.02 | 0.25 ± 0.04 | 0.38 ± 0.03 | 0.33 ± 0.08 | 0.30 ± 0.03 | 0.40 ± 0.07 |
| PE O-20:0/20:4 | 780.5902 | [M-H]- | 14.90 | 0.026 ± 0.015 | 0.009 ± 0.006 | 0.015 ± 0.010 | <0.001 | 0.051 ± 0.013 | <0.001 |
| PE P-16:0/16:0 | 674.5127 | [M-H]- | 15.04 | 0.015 ± 0.001 | 0.021 ± 0.002 | 0.017 ± 0.001 | 0.016 ± 0.001 | 0.021 ± 0.001 | 0.015 ± 0.001 |
| PE P-16:0/18:1 | 700.5281 | [M-H]-\|[M+H]+ | 15.10 | 0.38 ± 0.01 | 0.47 ± 0.05 | 0.50 ± 0.01 | 0.41 ± 0.04 | 0.58 ± 0.04 | 0.37 ± 0.04 |
| PE P-16:0/18:2 | 698.5121 | [M-H]-\|[M+H]+ | 14.02 | 0.83 ± 0.03 | 0.98 ± 0.14 | 1.09 ± 0.04 | 0.79 ± 0.12 | 1.13 ± 0.07 | 0.45 ± 0.04 |
| PE P-16:0/18:3 | 696.4962 | [M-H]- | 12.84 | 0.059 ± 0.008 | 0.065 ± 0.011 | 0.12 ± 0.01 | 0.062 ± 0.014 | 0.10 ± 0.01 | 0.022 ± 0.008 |
| PE P-16:0/20:3 | 724.5286 | [M-H]- | 14.75 | 0.025 ± <0.001 | 0.020 ± 0.004 | 0.021 ± 0.003 | 0.010 ± 0.002 | 0.023 ± 0.003 | 0.007 ± 0.001 |
| PE P-16:0/20:4 | 722.5129 | [M-H]-\|[M+H]+ | 13.68 | 2.46 ± 0.07 | 3.18 ± 0.42 | 2.77 ± 0.01 | 2.13 ± 0.40 | 3.22 ± 0.32 | 1.80 ± 0.26 |
| PE P-16:0/20:5 | 720.4961 | [M-H]-\|[M+H]+ | 12.55 | 0.15 ± 0.003 | 0.29 ± 0.04 | 0.27 ± 0.01 | 1.09 ± 0.12 | 0.21 ± 0.01 | 1.44 ± 0.07 |
| PE P-16:0/22:4 | 750.5441 | [M-H]-\|[M+H]+ | 14.69 | 0.42 ± 0.01 | 0.37 ± 0.01 | 0.42 ± 0.01 | 0.25 ± 0.003 | 0.42 ± 0.01 | 0.20 ± 0.01 |
| PE P-16:0/22:6 | 746.5122 | [M-H]-\|[M+H]+ | 13.15 | 1.21 ± 0.06 | 1.77 ± 0.27 | 2.01 ± 0.04 | 2.20 ± 0.38 | 1.76 ± 0.29 | 2.05 ± 0.32 |
| PE P-18:0/16:0 | 702.5436 | [M-H]-\|[M+H]+ | 16.13 | 0.031 ± 0.001 | 0.038 ± 0.004 | 0.036 ± 0.002 | 0.031 ± 0.001 | 0.034 ± 0.003 | 0.022 ± 0.002 |
| PE P-18:0/18:1 | 728.5608 | [M-H]-\|[M+H]+ | 16.17 | 0.41 ± 0.02 | 0.47 ± 0.005 | 0.54 ± 0.02 | 0.43 ± 0.01 | 0.65 ± 0.03 | 0.31 ± 0.01 |
| PE P-18:0/18:2 | 726.5443 | [M-H]-\|[M+H]+ | 15.33 | 2.06 ± 0.004 | 2.18 ± 0.08 | 2.91 ± 0.06 | 2.04 ± 0.04 | 2.71 ± 0.04 | 0.94 ± 0.03 |
| PE P-18:0/20:3 | 752.5593 | [M-H]-\|[M+H]+ | 15.56 | 0.49 ± 0.01 | 0.41 ± 0.03 | 0.57 ± 0.02 | 0.28 ± 0.01 | 0.71 ± 0.02 | 0.19 ± 0.01 |
| PE P-18:0/20:4 | 750.5446 | [M-H]-\|[M+H]+ | 15.06 | 5.65 ± 0.03 | 5.70 ± 0.75 | 6.02 ± 0.08 | 4.39 ± 0.42 | 6.90 ± 0.73 | 3.40 ± 0.25 |
| PE P-18:0/20:5 | 748.5276 | [M-H]-\|[M+H]+ | 14.15 | 0.78 ± 0.06 | 0.70 ± 0.07 | 0.80 ± 0.01 | 2.15 ± 0.08 | 0.78 ± 0.07 | 2.56 ± 0.09 |
| PE P-18:0/22:4 | 778.5753 | [M-H]-\|[M+H]+ | 15.83 | 0.22 ± 0.002 | 0.20 ± 0.005 | 0.24 ± 0.004 | 0.11 ± 0.01 | 0.19 ± 0.006 | 0.076 ± 0.003 |
| PE P-18:0/22:5 | 776.5601 | [M-H]-\|[M+H]+ | 15.05 | 0.80 ± 0.02 | 0.81 ± 0.05 | 1.13 ± 0.02 | 0.97 ± 0.02 | 0.91 ± 0.04 | 0.74 ± 0.02 |
| PE P-18:0/22:6 | 774.5443 | [M-H]-\|[M+H]+ | 14.65 | 1.36 ± 0.04 | 1.84 ± 0.29 | 2.16 ± 0.03 | 2.58 ± 0.36 | 2.08 ± 0.09 | 2.02 ± 0.20 |
| PE P-18:1/17:1 | 714.5435 | [M+H]+ | 14.71 | 0.039 ± 0.003 | 0.037 ± 0.005 | 0.055 ± 0.002 | 0.029 ± 0.003 | 0.056 ± 0.002 | 0.009 ± 0.002 |
| PE P-18:1/18:1 | 728.5593 | [M+H]+ | 15.20 | 0.11 ± 0.002 | 0.29 ± 0.16 | 0.19 ± 0.003 | 0.28 ± 0.13 | 0.38 ± 0.13 | 0.17 ± 0.03 |
| PE P-18:1/18:2 | 724.5279 | [M-H]-\|[M+H]+ | 14.08 | 1.05 ± 0.07 | 1.26 ± 0.14 | 1.21 ± 0.01 | 0.98 ± 0.07 | 1.40 ± 0.13 | 0.68 ± 0.05 |
| PE P-18:1/18:3 | 724.5263 | [M+H]+ | 12.88 | 0.005 ± 0.003 | 0.016 ± 0.010 | 0.004 ± 0.004 | 0.052 ± 0.005 | 0.016 ± <0.001 | 0.073 ± 0.031 |
| PE P-18:1/20:3 | 752.5596 | [M+H]+ | 14.50 | 0.099 ± 0.010 | 0.13 ± 0.03 | 0.15 ± 0.03 | 0.12 ± 0.02 | 0.21 ± 0.01 | 0.12 ± 0.02 |
| PE P-18:1/20:4 | 748.5279 | [M-H]-\|[M+H]+ | 13.79 | 2.79 ± 0.18 | 3.54 ± 0.12 | 3.45 ± 0.01 | 3.33 ± 0.56 | 3.90 ± 0.17 | 3.10 ± 0.55 |
| PE P-18:1/20:5 | 746.5127 | [M-H]-\|[M+H]+ | 12.82 | 0.005 ± 0.002 | 0.017 ± 0.015 | 0.013 ± 0.014 | 0.54 ± 0.28 | 0.004 ± 0.007 | 0.60 ± 0.46 |
| PE P-18:1/22:2 | 782.6060 | [M+H]+ | 16.29 | 0.016 ± 0.005 | 0.009 ± 0.006 | 0.006 ± 0.007 | 0.011 ± 0.007 | 0.010 ± 0.005 | 0.002 ± 0.002 |
| PE P-18:1/22:4 | 778.5748 | [M+H]+ | 14.91 | 0.15 ± 0.01 | 0.21 ± 0.06 | 0.25 ± 0.01 | 0.27 ± 0.10 | 0.26 ± 0.06 | 0.22 ± 0.05 |
| PE P-18:1/22:5 | 774.5439 | [M-H]-\|[M+H]+ | 13.80 | 0.15 ± 0.01 | 0.15 ± 0.003 | 0.21 ± 0.01 | 0.21 ± 0.01 | 0.19 ± 0.01 | 0.17 ± 0.01 |
| PE P-18:1/22:6 | 772.5286 | [M-H]-\|[M+H]+ | 13.25 | 0.57 ± 0.05 | 0.86 ± 0.23 | 1.16 ± 0.02 | 1.37 ± 0.32 | 0.84 ± 0.18 | 1.02 ± 0.23 |
| PE P-18:1/24:1 | 812.6543 | [M+H]+ | 17.74 | 0.006 ± 0.001 | 0.008 ± 0.003 | 0.006 ± 0.001 | <0.001 | 0.016 ± 0.002 | 0.005 ± 0.004 |
| PE P-20:0/18:1 | 756.5902 | [M-H]-\|[M+H]+ | 17.04 | 0.057 ± 0.001 | 0.077 ± 0.004 | 0.084 ± 0.001 | 0.075 ± 0.005 | 0.076 ± 0.008 | 0.058 ± 0.003 |
| PE P-20:0/18:2 | 754.5753 | [M-H]-\|[M+H]+ | 16.35 | 0.22 ± 0.01 | 0.27 ± 0.006 | 0.30 ± 0.01 | 0.27 ± 0.004 | 0.26 ± 0.01 | 0.15 ± 0.01 |
| PE P-20:0/20:1 | 786.6382 | [M+H]+ | 17.85 | 0.001 ± <0.001 | <0.001 | 0.001 ± <0.001 | 0.001 ± <0.001 | 0.001 ± 0.001 | 0.002 ± 0.003 |
| PE P-20:0/20:2 | 784.6212 | [M+H]+ | 17.19 | 0.017 ± 0.007 | 0.043 ± 0.004 | 0.044 ± 0.008 | 0.012 ± 0.003 | 0.022 ± 0.008 | 0.018 ± 0.001 |
| PE P-20:0/20:3 | 780.5894 | [M-H]- | 16.54 | 0.052 ± 0.001 | 0.044 ± 0.001 | 0.062 ± 0.002 | 0.035 ± 0.001 | 0.061 ± 0.001 | 0.020 ± 0.001 |
| PE P-20:0/20:4 | 778.5746 | [M-H]-\|[M+H]+ | 16.13 | 0.36 ± 0.004 | 0.43 ± 0.03 | 0.44 ± 0.01 | 0.38 ± 0.01 | 0.36 ± 0.02 | 0.27 ± 0.01 |
| PE P-20:0/22:3 | 810.6390 | [M+H]+ | 17.31 | <0.001 | <0.001 | 0.001 ± 0.001 | 0.003 ± 0.001 | 0.001 ± 0.001 | <0.001 |
| PE P-20:0/22:5 | 806.6066 | [M+H]+ | 16.15 | 0.006 ± 0.001 | 0.007 ± 0.002 | 0.015 ± 0.004 | 0.006 ± 0.001 | 0.011 ± 0.003 | 0.007 ± 0.001 |
| PE P-20:0/22:6 | 802.5752 | [M-H]- | 15.80 | 0.087 ± 0.007 | 0.17 ± 0.01 | 0.15 ± 0.01 | 0.25 ± 0.01 | 0.150 ± 0.010 | 0.19 ± 0.01 |
| PE P-20:1/18:2 | 754.5755 | [M+H]+ | 15.26 | 0.30 ± 0.01 | 0.24 ± 0.01 | 0.35 ± 0.02 | 0.23 ± 0.02 | 0.51 ± 0.01 | 0.14 ± 0.01 |
| PE P-20:1/22:6 | 800.5607 | [M-H]- | 14.63 | 0.048 ± 0.005 | 0.083 ± 0.003 | 0.11 ± 0.004 | 0.18 ± 0.01 | 0.089 ± 0.004 | 0.084 ± 0.002 |
| PS P-16:0/18:2 | 742.5038 | [M-H]- | 14.68 | <0.001 | <0.001 | <0.001 | <0.001 | <0.001 | <0.001 |
| PS P-16:0/20:3 | 768.5168 | [M-H]- | 15.11 | <0.001 | <0.001 | <0.001 | <0.001 | <0.001 | <0.001 |
| PS P-16:0/20:4 | 766.5019 | [M-H]- | 14.43 | <0.001 | <0.001 | <0.001 | <0.001 | <0.001 | <0.001 |
| PS P-16:0/22:4 | 794.5316 | [M-H]- | 15.17 | 0.012 ± 0.003 | 0.043 ± 0.003 | 0.047 ± 0.007 | 0.051 ± 0.010 | 0.034 ± 0.002 | 0.021 ± 0.010 |
| PS P-16:0/22:5 | 792.5161 | [M-H]- | 14.50 | <0.001 | <0.001 | <0.001 | <0.001 | <0.001 | <0.001 |
| PS P-16:0/22:6 | 790.5033 | [M-H]- | 11.03 | 0.013 ± 0.002 | 0.036 ± 0.016 | 0.007 ± 0.003 | 0.054 ± 0.025 | 0.059 ± 0.025 | 0.12 ± 0.07 |
| PS P-16:0/24:0 | 830.6293 | [M-H]- | 17.82 | <0.001 | 0.001 ± 0.002 | 0.001 ± 0.002 | <0.001 | <0.001 | <0.001 |
| PS P-16:1/16:0 | 716.4867 | [M-H]- | 11.59 | <0.001 | <0.001 | <0.001 | <0.001 | <0.001 | <0.001 |
| PS P-16:1/18:3 | 738.4730 | [M-H]- | 12.85 | <0.001 | <0.001 | <0.001 | <0.001 | <0.001 | <0.001 |
| PS P-16:1/22:5 | 790.5011 | [M-H]- | 12.83 | <0.001 | <0.001 | <0.001 | <0.001 | <0.001 | <0.001 |
| PS P-18:0/18:2 | 770.5347 | [M-H]- | 12.88 | 0.088 ± 0.010 | 0.16 ± 0.04 | 0.18 ± 0.04 | 0.24 ± 0.04 | 0.25 ± 0.05 | 0.24 ± 0.02 |
| PS P-18:0/20:3 | 796.5477 | [M-H]- | 16.16 | 0.043 ± 0.003 | 0.13 ± 0.01 | 0.11 ± 0.005 | 0.11 ± 0.004 | 0.14 ± 0.03 | 0.058 ± 0.012 |
| PS P-18:0/20:4 | 794.5338 | [M-H]- | 12.85 | 0.19 ± 0.03 | 0.29 ± 0.13 | 0.43 ± 0.01 | 0.26 ± 0.10 | 0.34 ± 0.08 | 0.20 ± 0.02 |
| PS P-18:0/22:5 | 820.5517 | [M-H]- | 12.85 | 0.068 ± 0.010 | 0.11 ± 0.03 | 0.16 ± 0.01 | 0.14 ± 0.03 | 0.16 ± 0.03 | 0.14 ± 0.07 |
| PS P-18:0/22:6 | 818.5353 | [M-H]- | 12.36 | 0.009 ± 0.003 | 0.039 ± 0.005 | 0.031 ± 0.012 | 0.077 ± 0.009 | 0.062 ± 0.020 | 0.072 ± 0.032 |
| PS P-18:1/20:4 | 792.5195 | [M-H]- | 11.39 | 0.002 ± <0.001 | 0.010 ± 0.005 | 0.004 ± 0.003 | 0.018 ± 0.006 | 0.018 ± 0.006 | 0.025 ± 0.003 |
| PS P-18:1/22:5 | 818.5323 | [M-H]- | 14.46 | <0.001 | <0.001 | <0.001 | <0.001 | <0.001 | <0.001 |
| PS P-18:1/22:6 | 816.5164 | [M-H]- | 13.40 | <0.001 | <0.001 | <0.001 | <0.001 | <0.001 | <0.001 |
| PS P-18:1/23:0 | 842.6297 | [M-H]- | 17.08 | <0.001 | <0.001 | <0.001 | <0.001 | <0.001 | <0.001 |
| PS P-18:1/24:1 | 854.6292 | [M-H]- | 16.59 | <0.001 | <0.001 | <0.001 | <0.001 | <0.001 | <0.001 |
| PS P-20:0/16:0 | 774.5660 | [M-H]- | 11.28 | 2.35 ± 0.22 | 2.69 ± 1.68 | 4.40 ± 0.75 | 2.47 ± 1.16 | 2.33 ± 1.63 | 2.28 ± 1.71 |
| PS P-20:0/18:0 | 802.5975 | [M-H]- | 14.60 | 0.037 ± 0.021 | 0.17 ± 0.08 | 0.212 ± 0.054 | 0.032 ± 0.049 | 0.065 ± 0.026 | 0.10 ± 0.06 |
| PS P-20:0/18:1 | 800.5836 | [M-H]- | 13.99 | 0.067 ± 0.012 | 0.20 ± 0.03 | 0.14 ± 0.03 | 0.20 ± 0.02 | 0.33 ± 0.05 | 0.063 ± 0.005 |
| PS P-20:0/18:2 | 798.5646 | [M-H]- | 12.59 | 0.021 ± 0.003 | 0.049 ± 0.014 | 0.053 ± 0.009 | 0.027 ± 0.003 | 0.11 ± 0.03 | 0.059 ± 0.013 |
| PS P-20:0/20:3 | 824.5798 | [M-H]- | 12.85 | <0.001 | 0.004 ± 0.008 | 0.006 ± 0.010 | <0.001 | 0.025 ± 0.022 | <0.001 |
| PS P-20:0/20:4 | 822.5640 | [M-H]- | 12.58 | 0.001 ± <0.001 | 0.006 ± 0.005 | 0.003 ± 0.002 | 0.066 ± 0.013 | 0.010 ± 0.007 | 0.11 ± 0.02 |
| PS P-20:1/18:0 | 800.5817 | [M-H]- | 13.33 | 0.021 ± 0.015 | 0.066 ± 0.048 | 0.081 ± 0.032 | 0.025 ± 0.022 | 0.047 ± 0.051 | 0.044 ± 0.040 |
| PS P-20:1/20:4 | 820.5473 | [M-H]- | 15.62 | 0.001 ± 0.001 | <0.001 | <0.001 | <0.001 | 0.002 ± 0.003 | <0.001 |
| PS P-20:1/22:6 | 844.5474 | [M-H]- | 15.07 | <0.001 | <0.001 | <0.001 | <0.001 | <0.001 | <0.001 |
| LPA 16:0 | 409.2370 | [M-H]- | 5.08 | 0.016 ± 0.002 | 0.021 ± 0.003 | 0.018 ± 0.002 | 0.018 ± 0.002 | 0.017 ± <0.001 | 0.020 ± 0.001 |
| LPC 14:0 | 512.2986 | [M+HCO2]- | 3.98 | 0.73 ± 0.03 | 1.20 ± 0.06 | 1.11 ± 0.02 | 1.23 ± 0.04 | 0.27 ± 0.02 | 0.42 ± 0.03 |
| LPC 15:0 | 526.3143 | [M+HCO2]-\|[M+H]+ | 4.52 | 0.33 ± 0.02 | 0.51 ± 0.01 | 0.67 ± 0.02 | 0.50 ± 0.01 | 0.22 ± 0.01 | 0.26 ± 0.01 |
| LPC 15:1 | 524.2983 | [M+HCO2]- | 3.96 | 0.001 ± <0.001 | 0.003 ± 0.001 | 0.003 ± 0.001 | 0.003 ± <0.001 | <0.001 | <0.001 |
| LPC 16:0 | 540.3302 | [M+HCO2]-\|[M+H]+\|[M+Na]+ | 5.13 | 48.6 ± 2.6 | 58.5 ± 1.1 | 59.6 ± 1.4 | 65.9 ± 1.0 | 24.8 ± 0.1 | 28.8 ± 0.7 |
| LPC 16:1 | 538.3153 | [M+HCO2]-\|[M+H]+\|[M+Na]+ | 4.19 | 1.74 ± 0.05 | 1.54 ± 0.07 | 2.01 ± 0.01 | 1.75 ± 0.05 | 0.64 ± 0.03 | 0.80 ± 0.05 |
| LPC 17:0 | 554.3451 | [M+HCO2]- | 5.50 | 1.08 ± 0.06 | 1.38 ± 0.04 | 1.65 ± 0.01 | 1.52 ± 0.09 | 0.64 ± 0.01 | 0.67 ± 0.03 |
| LPC 17:1 | 552.3301 | [M+HCO2]- | 4.73 | 0.12 ± 0.01 | 0.15 ± 0.00 | 0.16 ± 0.004 | 0.19 ± 0.003 | 0.066 ± 0.002 | 0.073 ± 0.003 |
| LPC 17:2 | 506.3259 | [M+H]+ | 5.41 | 0.022 ± 0.001 | 0.016 ± 0.002 | 0.021 ± 0.003 | 0.013 ± 0.001 | 0.005 ± 0.002 | 0.004 ± 0.001 |
| LPC 18:0 | 568.3613 | [M+HCO2]-\|[M+H]+\|[M+Na]+ | 6.59 | 22.2 ± 1.4 | 26.4 ± 0.1 | 31.6 ± 0.7 | 29.0 ± 0.4 | 8.51 ± 0.20 | 9.63 ± 0.14 |
| LPC 18:1 | 566.3459 | [M+HCO2]-\|[M+H]+\|[M+Na]+ | 5.32 | 12.8 ± 0.05 | 14.6 ± 0.1 | 14.5 ± 0.1 | 17.6 ± 0.1 | 7.33 ± 0.02 | 6.08 ± 0.04 |
| LPC 18:2 | 564.3310 | [M+HCO2]-\|[M+H]+\|[M+Na]+ | 4.46 | 14.6 ± 0.5 | 20.0 ± 0.7 | 16.5 ± 0.5 | 23.6 ± 0.6 | 11.4 ± 0.7 | 8.09 ± 0.59 |
| LPC 18:3 | 562.3144 | [M+HCO2]- | 3.85 | 0.31 ± 0.01 | 0.38 ± 0.01 | 0.34 ± 0.002 | 0.67 ± 0.02 | 0.18 ± 0.007 | 0.12 ± 0.006 |
| LPC 18:4 | 516.3095 | [M+H]+ | 3.41 | 0.002 ± <0.001 | 0.001 ± <0.001 | <0.001 | 0.012 ± <0.001 | <0.001 | 0.001 ± <0.001 |
| LPC 19:0 | 582.3772 | [M+HCO2]- | 7.33 | 0.066 ± 0.005 | 0.11 ± 0.003 | 0.11 ± 0.004 | 0.12 ± 0.003 | 0.047 ± 0.001 | 0.047 ± <0.001 |
| LPC 20:0 | 596.3929 | [M+HCO2]- | 8.05 | 0.082 ± 0.009 | 0.14 ± 0.01 | 0.12 ± 0.004 | 0.16 ± 0.01 | 0.060 ± 0.004 | 0.062 ± 0.004 |
| LPC 20:1 | 594.3774 | [M+HCO2]- | 6.71 | 0.15 ± 0.01 | 0.23 ± 0.01 | 0.21 ± 0.01 | 0.25 ± 0.02 | 0.11 ± 0.01 | 0.079 ± 0.009 |
| LPC 20:2 | 592.3616 | [M+HCO2]-\|[M+Na]+ | 5.50 | 0.26 ± 0.003 | 0.35 ± 0.004 | 0.34 ± 0.01 | 0.36 ± 0.01 | 0.19 ± 0.003 | 0.10 ± 0.004 |
| LPC 20:3 | 590.3454 | [M+HCO2]-\|[M+Na]+ | 4.82 | 1.16 ± 0.03 | 1.36 ± 0.01 | 1.51 ± 0.03 | 1.18 ± 0.01 | 0.71 ± 0.01 | 0.45 ± 0.01 |
| LPC 20:4 | 588.3301 | [M+HCO2]-\|[M+H]+\|[M+Na]+ | 4.33 | 3.48 ± 0.06 | 4.42 ± 0.20 | 3.95 ± 0.12 | 3.69 ± 0.11 | 1.83 ± 0.15 | 1.78 ± 0.13 |
| LPC 20:5 | 586.3147 | [M+HCO2]- | 3.74 | 0.21 ± 0.003 | 0.35 ± 0.02 | 0.32 ± 0.01 | 1.61 ± 0.04 | 0.13 ± 0.006 | 1.25 ± 0.08 |
| LPC 21:0 | 566.4198 | [M+H]+ | 8.76 | <0.001 | 0.001 ± 0.001 | 0.001 ± <0.001 | 0.002 ± 0.001 | <0.001 | <0.001 |
| LPC 22:0 | 624.4241 | [M+HCO2]- | 9.46 | 0.023 ± 0.002 | 0.040 ± 0.001 | 0.028 ± <0.001 | 0.042 ± 0.001 | 0.021 ± 0.001 | 0.025 ± 0.001 |
| LPC 22:1 | 622.4087 | [M+HCO2]- | 8.09 | 0.012 ± 0.001 | 0.017 ± 0.001 | 0.012 ± 0.001 | 0.023 ± 0.001 | 0.009 ± 0.001 | 0.013 ± <0.001 |
| LPC 22:3 | 574.3870 | [M+H]+ | 6.20 | <0.001 | <0.001 | <0.001 | <0.001 | <0.001 | <0.001 |
| LPC 22:4 | 616.3623 | [M+HCO2]-\|[M+H]+ | 5.21 | 0.079 ± 0.003 | 0.069 ± 0.006 | 0.062 ± 0.003 | 0.034 ± 0.005 | 0.029 ± 0.003 | 0.019 ± 0.003 |
| LPC 22:5 | 614.3458 | [M+HCO2]-\|[M+H]+\|[M+Na]+ | 4.48 | 0.17 ± 0.01 | 0.16 ± 0.006 | 0.19 ± 0.01 | 0.34 ± 0.01 | 0.082 ± 0.006 | 0.17 ± 0.02 |
| LPC 22:6 | 612.3302 | [M+HCO2]-\|[M+H]+\|[M+Na]+ | 4.15 | 0.53 ± 0.01 | 1.03 ± 0.06 | 0.79 ± 0.02 | 1.96 ± 0.06 | 0.41 ± 0.03 | 0.76 ± 0.05 |
| LPC 24:0 | 652.4559 | [M+HCO2]- | 10.83 | 0.038 ± 0.003 | 0.054 ± 0.005 | 0.048 ± 0.001 | 0.063 ± 0.007 | 0.036 ± 0.002 | 0.047 ± 0.003 |
| LPC 24:1 | 650.4405 | [M+HCO2]-\|[M+H]+ | 9.45 | 0.011 ± 0.001 | 0.014 ± <0.001 | 0.011 ± <0.001 | 0.024 ± 0.001 | 0.012 ± <0.001 | 0.020 ± 0.001 |
| LPC 26:0 | 680.4872 | [M+HCO2]- | 12.22 | 0.007 ± 0.001 | 0.009 ± 0.003 | 0.008 ± 0.001 | 0.011 ± 0.003 | 0.008 ± 0.002 | 0.011 ± 0.003 |
| LPE 15:0 | 438.2620 | [M-H]- | 4.68 | 0.007 ± 0.001 | 0.006 ± 0.001 | 0.006 ± <0.001 | 0.007 ± <0.001 | 0.005 ± <0.001 | 0.005 ± <0.001 |
| LPE 16:0 | 452.2778 | [M-H]-\|[M+H]+ | 5.30 | 0.55 ± 0.05 | 0.57 ± 0.02 | 0.49 ± 0.01 | 0.84 ± 0.02 | 0.31 ± 0.01 | 0.59 ± 0.004 |
| LPE 16:1 | 450.2624 | [M-H]-\|[M+H]+ | 4.33 | 0.033 ± 0.001 | 0.020 ± 0.002 | 0.018 ± <0.001 | 0.027 ± 0.001 | 0.008 ± 0.001 | 0.035 ± 0.002 |
| LPE 17:0 | 466.2932 | [M-H]- | 6.08 | 0.007 ± 0.001 | 0.007 ± 0.001 | 0.008 ± <0.001 | 0.007 ± 0.002 | 0.004 ± <0.001 | 0.007 ± 0.001 |
| LPE 18:0 | 480.3091 | [M-H]-\|[M+H]+\|[M+Na]+ | 6.82 | 0.66 ± 0.07 | 0.73 ± 0.02 | 0.82 ± 0.02 | 0.83 ± 0.02 | 0.30 ± <0.001 | 0.41 ± 0.006 |
| LPE 18:1 | 478.2935 | [M-H]-\|[M+Na]+ | 5.49 | 0.72 ± 0.01 | 0.84 ± 0.01 | 0.70 ± 0.01 | 1.23 ± 0.01 | 0.55 ± 0.005 | 0.52 ± 0.006 |
| LPE 18:2 | 476.2778 | [M-H]-\|[M+H]+ | 4.62 | 0.88 ± 0.04 | 0.89 ± 0.05 | 0.76 ± 0.02 | 1.22 ± 0.02 | 0.52 ± 0.03 | 0.48 ± 0.01 |
| LPE 18:3 | 474.2621 | [M-H]-\|[M+H]+\|[M+Na]+ | 3.96 | 0.026 ± 0.001 | 0.025 ± 0.002 | 0.023 ± 0.001 | 0.060 ± 0.002 | 0.019 ± 0.001 | 0.016 ± 0.001 |
| LPE 20:1 | 506.3245 | [M-H]- | 6.95 | 0.008 ± 0.001 | 0.013 ± 0.001 | 0.010 ± <0.001 | 0.015 ± 0.001 | 0.007 ± 0.001 | 0.006 ± 0.001 |
| LPE 20:3 | 502.2930 | [M-H]-\|[M+H]+ | 4.98 | 0.066 ± 0.003 | 0.062 ± 0.004 | 0.063 ± 0.003 | 0.056 ± 0.001 | 0.043 ± 0.001 | 0.037 ± 0.001 |
| LPE 20:4 | 500.2780 | [M-H]-\|[M+H]+ | 4.48 | 0.86 ± 0.04 | 0.85 ± 0.01 | 0.70 ± 0.03 | 0.73 ± 0.02 | 0.47 ± 0.01 | 0.48 ± 0.02 |
| LPE 20:5 | 498.2624 | [M-H]-\|[M+H]+ | 3.86 | 0.023 ± 0.001 | 0.026 ± 0.001 | 0.021 ± <0.001 | 0.12 ± 0.01 | 0.011 ± <0.001 | 0.19 ± 0.01 |
| LPE 22:5 | 526.2944 | [M-H]-\|[M+H]+ | 4.99 | 0.048 ± 0.003 | 0.036 ± 0.004 | 0.028 ± 0.003 | 0.019 ± 0.001 | 0.027 ± 0.004 | 0.013 ± 0.001 |
| LPE 22:6 | 524.2777 | [M-H]-\|[M+H]+\|[M+Na]+ | 4.28 | 0.34 ± 0.02 | 0.48 ± 0.03 | 0.35 ± 0.02 | 0.69 ± 0.02 | 0.29 ± 0.004 | 0.57 ± 0.02 |
| LPI 18:0 | 599.3195 | [M-H]- | 5.48 | 0.064 ± 0.006 | 0.024 ± 0.004 | 0.032 ± 0.002 | 0.031 ± 0.002 | 0.023 ± 0.001 | 0.026 ± 0.001 |
| LPI 18:1 | 597.3039 | [M-H]- | 4.55 | 0.020 ± 0.003 | 0.008 ± 0.002 | 0.006 ± <0.001 | 0.020 ± <0.001 | 0.004 ± <0.001 | 0.007 ± <0.001 |
| LPI 18:2 | 595.2885 | [M-H]- | 3.83 | 0.023 ± 0.004 | 0.010 ± 0.001 | 0.009 ± <0.001 | 0.018 ± 0.001 | 0.004 ± <0.001 | 0.005 ± <0.001 |
| LPI 20:4 | 619.2885 | [M-H]- | 3.74 | 0.043 ± 0.005 | 0.021 ± 0.001 | 0.022 ± 0.001 | 0.033 ± 0.001 | 0.011 ± <0.001 | 0.015 ± <0.001 |
| LPI 22:6 | 643.2881 | [M-H]- | 3.62 | 0.004 ± 0.001 | 0.004 ± 0.001 | 0.003 ± 0.001 | 0.003 ± 0.002 | 0.001 ± <0.001 | 0.002 ± 0.002 |
| MMPE 15:0_20:3 | 740.5224 | [M-H]- | 11.72 | 0.011 ± 0.003 | 0.007 ± 0.002 | 0.015 ± 0.001 | 0.017 ± 0.003 | 0.010 ± 0.006 | 0.011 ± 0.004 |
| MMPE 16:0_18:1 | 730.5397 | [M-H]- | 14.90 | <0.001 | <0.001 | <0.001 | <0.001 | 0.002 ± 0.002 | 0.001 ± 0.001 |
| MMPE 16:0_22:5 | 778.5387 | [M-H]- | 13.34 | 0.011 ± 0.003 | 0.011 ± 0.007 | 0.014 ± 0.003 | 0.013 ± 0.008 | 0.015 ± 0.015 | 0.020 ± 0.015 |
| MMPE 16:1_20:5 | 748.4923 | [M-H]- | 11.09 | <0.001 | <0.001 | <0.001 | <0.001 | <0.001 | <0.001 |
| MMPE 16:1_22:5 | 776.5241 | [M-H]- | 13.21 | 0.002 ± 0.002 | 0.007 ± 0.001 | 0.001 ± 0.001 | 0.016 ± 0.002 | 0.005 ± 0.004 | 0.056 ± 0.009 |
| MMPE 16:1_22:6 | 774.5086 | [M-H]- | 11.51 | <0.001 | <0.001 | <0.001 | <0.001 | <0.001 | 0.006 ± 0.001 |
| MMPE 17:1_22:5 | 790.5416 | [M-H]- | 13.45 | 0.002 ± 0.003 | <0.001 | 0.001 ± 0.002 | <0.001 | 0.001 ± 0.003 | <0.001 |
| MMPE 18:1_20:5 | 776.5253 | [M-H]- | 12.23 | 0.001 ± <0.001 | <0.001 | <0.001 | <0.001 | <0.001 | <0.001 |
| MMPE 18:2_20:5 | 774.5087 | [M-H]- | 11.15 | <0.001 | <0.001 | <0.001 | 0.002 ± 0.002 | <0.001 | 0.003 ± 0.001 |
| OxLPC 15:0(CHO) | 496.3046 | [M+H]+ | 4.86 | <0.001 | <0.001 | <0.001 | <0.001 | <0.001 | <0.001 |
| OxLPC 16:1(CHO) | 508.3040 | [M+H]+ | 5.52 | <0.001 | <0.001 | <0.001 | <0.001 | <0.001 | <0.001 |
| OxLPC 16:1(OO) | 526.3146 | [M+H]+ | 5.75 | <0.001 | <0.001 | <0.001 | <0.001 | <0.001 | <0.001 |
| OxLPC 17:2(Ke) | 520.3030 | [M+H]+ | 4.46 | <0.001 | <0.001 | <0.001 | <0.001 | <0.001 | <0.001 |
| OxLPC 17:2(KeOH) | 536.2991 | [M+H]+ | 4.25 | 0.004 ± 0.001 | 0.005 ± 0.001 | 0.005 ± <0.001 | 0.005 ± <0.001 | 0.002 ± <0.001 | 0.002 ± <0.001 |
| OxLPC 18:1(OH) | 582.3409 | [M+HCO2]- | 2.91 | 0.001 ± 0.001 | 0.002 ± <0.001 | 0.001 ± <0.001 | 0.002 ± <0.001 | 0.001 ± <0.001 | 0.001 ± <0.001 |
| OxLPC 18:1(OOO) | 570.3404 | [M+H]+ | 3.90 | 0.010 ± 0.001 | 0.004 ± 0.001 | 0.003 ± <0.001 | 0.009 ± 0.001 | 0.002 ± <0.001 | 0.003 ± 0.001 |
| OxLPC 18:2(OOO) | 568.3250 | [M+H]+ | 3.39 | 0.023 ± <0.001 | 0.006 ± 0.001 | 0.004 ± <0.001 | 0.014 ± 0.001 | 0.005 ± <0.001 | 0.010 ± <0.001 |
| OxLPC 18:3(OH) | 534.3179 | [M+H]+ | 4.26 | 0.003 ± 0.002 | 0.003 ± 0.001 | 0.003 ± 0.001 | 0.003 ± 0.001 | 0.003 ± <0.001 | 0.006 ± <0.001 |
| OxLPC 18:4(CHO) | 530.2880 | [M+H]+ | 4.51 | 0.018 ± 0.002 | 0.006 ± 0.002 | 0.004 ± 0.002 | 0.004 ± 0.001 | 0.008 ± 0.001 | 0.004 ± <0.001 |
| OxLPC 18:4(OO) | 548.2999 | [M+H]+ | 4.46 | <0.001 | <0.001 | <0.001 | <0.001 | <0.001 | <0.001 |
| OxLPC 18:5(CHO) | 528.2713 | [M+H]+ | 4.60 | 0.001 ± <0.001 | <0.001 | <0.001 | <0.001 | <0.001 | <0.001 |
| OxLPC 19:4(COOH) | 560.2993 | [M+H]+ | 4.12 | 0.004 ± <0.001 | 0.004 ± <0.001 | 0.002 ± <0.001 | 0.002 ± 0.001 | 0.001 ± <0.001 | 0.001 ± <0.001 |
| OxLPC 19:5(CHO) | 542.2892 | [M+H]+ | 4.12 | <0.001 | <0.001 | <0.001 | <0.001 | <0.001 | <0.001 |
| OxLPC 19:5(COOH) | 558.2811 | [M+H]+ | 4.00 | 0.006 ± 0.003 | 0.011 ± 0.004 | 0.010 ± 0.001 | 0.010 ± 0.005 | 0.003 ± 0.001 | 0.005 ± 0.001 |
| OxLPC 20:2(OO) | 580.3613 | [M+H]+ | 5.22 | 0.019 ± 0.003 | 0.011 ± 0.003 | 0.008 ± <0.001 | 0.054 ± 0.002 | 0.015 ± 0.004 | 0.065 ± 0.006 |
| OxLPC 20:2(OOO) | 596.3578 | [M+H]+ | 5.02 | 0.004 ± <0.001 | <0.001 | <0.001 | 0.002 ± 0.001 | <0.001 | 0.004 ± <0.001 |
| OxLPC 20:3(OOO) | 594.3408 | [M+H]+ | 3.65 | 0.001 ± <0.001 | <0.001 | <0.001 | <0.001 | <0.001 | <0.001 |
| OxLPC 22:2(OO) | 608.3927 | [M+H]+ | 6.58 | <0.001 | <0.001 | <0.001 | <0.001 | <0.001 | 0.006 ± 0.001 |
| OxLPC 22:3(OH) | 634.3719 | [M+HCO2]- | 5.25 | <0.001 | <0.001 | <0.001 | <0.001 | <0.001 | <0.001 |
| OxLPC 22:3(OO) | 606.3775 | [M+H]+ | 5.23 | 0.001 ± <0.001 | <0.001 | <0.001 | 0.001 ± <0.001 | <0.001 | 0.004 ± 0.001 |
| OxLPC 24:1(KeOH) | 636.4245 | [M+H]+\|[M+Na]+ | 5.89 | 0.097 ± 0.002 | 0.049 ± 0.008 | 0.062 ± 0.002 | 0.060 ± 0.001 | 0.057 ± 0.006 | 0.063 ± 0.007 |
| OxLPC 24:1(OH) | 622.4456 | [M+H]+ | 8.08 | 0.005 ± <0.001 | 0.003 ± <0.001 | 0.002 ± <0.001 | 0.003 ± 0.001 | 0.004 ± <0.001 | 0.003 ± <0.001 |
| OxLPC 24:1(OO) | 638.4401 | [M+H]+ | 5.48 | <0.001 | <0.001 | <0.001 | <0.001 | <0.001 | <0.001 |
| OxLPC 24:1(OOO) | 654.4348 | [M+H]+ | 5.50 | 0.002 ± 0.001 | 0.001 ± 0.001 | <0.001 | 0.002 ± 0.001 | 0.001 ± 0.001 | 0.003 ± 0.001 |
| OxLPE 22:3(OH) | 548.3353 | [M+H]+ | 5.76 | <0.001 | <0.001 | <0.001 | <0.001 | <0.001 | <0.001 |
| OxPC 16:0_10:0(COOH) | 724.4411 | [M+HCO2]- | 6.69 | 0.030 ± 0.001 | 0.002 ± 0.001 | 0.001 ± <0.001 | 0.014 ± 0.001 | 0.019 ± 0.004 | 0.021 ± 0.002 |
| OxPC 16:0_11:0(CHO) | 722.4615 | [M+HCO2]- | 11.30 | <0.001 | <0.001 | <0.001 | <0.001 | <0.001 | 0.001 ± 0.001 |
| OxPC 16:0_11:1(CHO) | 720.4456 | [M+HCO2]- | 6.48 | 0.070 ± 0.005 | 0.040 ± 0.002 | 0.046 ± 0.004 | 0.052 ± 0.008 | 0.052 ± 0.001 | 0.040 ± 0.002 |
| OxPC 16:0_12:1(CHO) | 734.4619 | [M+HCO2]- | 10.65 | 0.035 ± 0.003 | 0.007 ± <0.001 | 0.007 ± 0.002 | 0.020 ± 0.002 | 0.013 ± 0.002 | 0.022 ± 0.001 |
| OxPC 16:0_12:2(COOH) | 748.4403 | [M+HCO2]- | 5.74 | 0.015 ± 0.002 | 0.007 ± 0.001 | 0.005 ± 0.001 | 0.017 ± 0.002 | 0.024 ± 0.002 | 0.016 ± 0.002 |
| OxPC 16:0_18:1(1O) | 820.5697 | [M+HCO2]- | 10.88 | 0.008 ± 0.003 | 0.002 ± 0.002 | <0.001 | 0.004 ± 0.004 | 0.003 ± 0.003 | 0.002 ± 0.001 |
| OxPC 16:0_18:1(OH) | 820.5714 | [M+HCO2]- | 12.25 | 0.34 ± 0.005 | 0.24 ± 0.05 | 0.33 ± 0.02 | 0.26 ± 0.04 | 0.28 ± 0.04 | 0.31 ± 0.05 |
| OxPC 16:0_18:2(1O) | 818.5549 | [M+HCO2]- | 9.43 | 0.014 ± 0.001 | <0.001 | 0.001 ± <0.001 | 0.001 ± 0.001 | 0.001 ± <0.001 | <0.001 |
| OxPC 16:0_18:2(OH) | 818.5548 | [M+HCO2]- | 11.29 | 0.33 ± 0.02 | 0.32 ± 0.04 | 0.41 ± 0.02 | 0.39 ± 0.03 | 0.32 ± 0.03 | 0.39 ± 0.05 |
| OxPC 16:0_20:4(1O) | 842.5550 | [M+HCO2]- | 9.36 | 0.006 ± 0.001 | <0.001 | <0.001 | <0.001 | <0.001 | <0.001 |
| OxPC 16:0_20:4(OH) | 842.5551 | [M+HCO2]- | 10.05 | 0.010 ± 0.002 | <0.001 | <0.001 | <0.001 | <0.001 | 0.005 ± 0.003 |
| OxPC 16:0_22:6(OH) | 866.5551 | [M+HCO2]- | 9.40 | <0.001 | <0.001 | <0.001 | <0.001 | <0.001 | <0.001 |
| OxPC 18:0_12:1(CHO) | 762.4927 | [M+HCO2]- | 11.82 | 0.030 ± 0.006 | 0.021 ± 0.002 | 0.025 ± 0.004 | 0.020 ± 0.012 | 0.027 ± 0.009 | 0.002 ± 0.003 |
| OxPC 18:0_18:2(1O) | 846.5856 | [M+HCO2]- | 10.41 | 0.003 ± 0.001 | <0.001 | <0.001 | <0.001 | <0.001 | <0.001 |
| OxPC 18:0_20:4(1O) | 870.5856 | [M+HCO2]- | 10.52 | 0.001 ± 0.001 | <0.001 | <0.001 | <0.001 | <0.001 | <0.001 |
| OxPC 18:0_20:4(OH) | 870.5867 | [M+HCO2]- | 10.85 | 0.006 ± 0.004 | 0.004 ± 0.003 | 0.003 ± 0.003 | 0.012 ± 0.010 | 0.005 ± 0.004 | 0.002 ± 0.002 |
| OxPC 18:1_18:0(1O) | 848.6010 | [M+HCO2]- | 13.81 | 0.009 ± 0.007 | 0.002 ± 0.003 | 0.006 ± 0.002 | 0.003 ± 0.003 | 0.008 ± 0.008 | 0.001 ± 0.001 |
| OxPC 18:1_18:1(1O) | 846.5841 | [M+HCO2]- | 12.58 | 0.035 ± 0.006 | 0.037 ± 0.006 | 0.051 ± 0.005 | 0.035 ± 0.005 | 0.037 ± 0.002 | 0.033 ± 0.006 |
| OxPC 18:1_18:3(1O) | 842.5555 | [M+HCO2]- | 11.04 | 0.085 ± 0.002 | 0.069 ± 0.003 | 0.091 ± 0.004 | 0.048 ± 0.003 | 0.054 ± 0.011 | 0.059 ± 0.007 |
| OxPC 18:1_20:3(1O) | 870.5858 | [M+HCO2]- | 12.54 | <0.001 | <0.001 | <0.001 | <0.001 | <0.001 | <0.001 |
| OxPC 18:2_18:1(OH) | 844.5699 | [M+HCO2]- | 11.56 | 0.019 ± 0.003 | 0.008 ± 0.007 | 0.023 ± 0.001 | 0.003 ± 0.001 | 0.007 ± 0.003 | 0.001 ± 0.001 |
| OxPC 18:2_20:3(OH) | 868.5699 | [M+HCO2]- | 11.51 | <0.001 | <0.001 | <0.001 | <0.001 | <0.001 | <0.001 |
| OxPE 18:0_22:3(OH) | 812.5815 | [M-H]- | 15.49 | 0.005 ± 0.001 | 0.004 ± <0.001 | 0.005 ± 0.002 | 0.005 ± 0.003 | 0.008 ± 0.002 | 0.024 ± 0.004 |
| OxPE 18:1_18:0(1O) | 760.5494 | [M-H]- | 12.15 | 0.010 ± 0.003 | 0.014 ± 0.002 | 0.014 ± 0.002 | 0.018 ± 0.002 | 0.022 ± 0.003 | 0.009 ± 0.002 |
| OxPE 18:1_18:1(1O) | 758.5337 | [M-H]- | 13.67 | 0.021 ± 0.003 | 0.025 ± 0.003 | 0.022 ± 0.001 | 0.020 ± 0.007 | 0.028 ± 0.001 | 0.013 ± 0.004 |
| OxPE 18:1_20:3(1O) | 782.5324 | [M-H]- | 12.81 | 0.098 ± 0.013 | 0.060 ± 0.026 | 0.14 ± 0.03 | 0.014 ± 0.005 | 0.11 ± 0.02 | 0.014 ± 0.003 |
| OxPE 18:1_20:3(2O) | 798.5285 | [M-H]- | 12.11 | 0.003 ± 0.002 | 0.005 ± 0.003 | 0.004 ± 0.003 | 0.005 ± 0.003 | 0.008 ± 0.005 | 0.006 ± 0.004 |
| OxPE 18:1_20:4(1O) | 780.5163 | [M-H]- | 14.71 | <0.001 | <0.001 | <0.001 | <0.001 | <0.001 | <0.001 |
| OxPG 16:0_18:0(1O) | 765.5269 | [M-H]- | 15.05 | <0.001 | <0.001 | <0.001 | <0.001 | <0.001 | <0.001 |
| OxPG 18:0_18:0(1O) | 793.5584 | [M-H]- | 14.90 | 0.040 ± 0.004 | 0.024 ± 0.002 | 0.023 ± 0.001 | <0.001 | 0.063 ± 0.002 | 0.002 ± 0.002 |
| OxPG 18:0_18:1(1O) | 791.5437 | [M-H]- | 13.61 | <0.001 | <0.001 | <0.001 | <0.001 | 0.003 ± 0.001 | <0.001 |
| OxPG 18:1_18:0(1O) | 791.5431 | [M-H]- | 12.97 | <0.001 | <0.001 | 0.001 ± 0.001 | 0.001 ± 0.001 | 0.001 ± 0.001 | 0.007 ± 0.007 |
| PC 12:0_14:0 | 650.4758 | [M+H]+ | 9.77 | 0.010 ± <0.001 | 0.037 ± 0.003 | 0.013 ± 0.002 | 0.042 ± 0.002 | 0.002 ± 0.001 | 0.004 ± 0.001 |
| PC 12:0_18:2 | 702.5076 | [M+H]+ | 10.14 | 0.014 ± 0.001 | 0.036 ± 0.001 | 0.020 ± 0.001 | 0.044 ± 0.001 | 0.006 ± <0.001 | 0.012 ± <0.001 |
| PC 14:0_14:0 | 722.4983 | [M+HCO2]-\|[M+H]+ | 10.96 | 0.024 ± <0.001 | 0.30 ± 0.03 | 0.004 ± 0.005 | 0.26 ± 0.03 | <0.001 | 0.022 ± 0.021 |
| PC 14:0_15:0 | 736.5130 | [M+HCO2]-\|[M+H]+ | 11.56 | <0.001 | 0.002 ± 0.003 | <0.001 | 0.005 ± 0.001 | <0.001 | 0.003 ± 0.003 |
| PC 14:0_16:0 | 750.5294 | [M+HCO2]-\|[M+H]+\|[M+Na]+ | 12.27 | 0.80 ± 0.07 | 1.62 ± 0.05 | 0.77 ± 0.07 | 1.56 ± 0.14 | 0.68 ± 0.06 | 1.65 ± 0.05 |
| PC 14:0_16:1 | 748.5126 | [M+HCO2]-\|[M+H]+ | 11.09 | 0.062 ± 0.009 | 0.083 ± 0.021 | 0.033 ± 0.006 | 0.10 ± 0.03 | 0.009 ± 0.006 | 0.19 ± 0.03 |
| PC 14:0_18:2 | 774.5288 | [M+HCO2]-\|[M+H]+\|[M+Na]+ | 11.34 | 1.85 ± 0.06 | 2.72 ± 0.16 | 3.16 ± 0.03 | 2.49 ± 0.22 | 2.09 ± 0.07 | 1.35 ± 0.07 |
| PC 14:0_18:3 | 772.5132 | [M+HCO2]-\|[M+H]+ | 10.52 | 0.033 ± 0.001 | 0.070 ± 0.006 | 0.069 ± 0.002 | 0.12 ± 0.01 | 0.047 ± 0.003 | 0.035 ± 0.001 |
| PC 14:0_20:4 | 798.5290 | [M+HCO2]-\|[M+H]+\|[M+Na]+ | 11.11 | 0.64 ± 0.02 | 1.16 ± 0.01 | 1.07 ± 0.02 | 0.82 ± 0.02 | 0.68 ± 0.02 | 0.55 ± 0.01 |
| PC 14:0_20:5 | 796.5126 | [M+HCO2]-\|[M+H]+ | 10.33 | 0.050 ± 0.003 | 0.11 ± 0.002 | 0.11 ± 0.004 | 0.25 ± 0.01 | 0.060 ± 0.005 | 0.31 ± 0.01 |
| PC 14:0_22:6 | 822.5291 | [M+HCO2]-\|[M+H]+\|[M+Na]+ | 10.79 | 0.18 ± 0.004 | 0.44 ± 0.02 | 0.40 ± 0.01 | 0.64 ± 0.01 | 0.27 ± 0.01 | 0.61 ± 0.005 |
| PC 15:0_16:1 | 762.5270 | [M+HCO2]-\|[M+H]+ | 11.70 | 0.001 ± 0.001 | 0.001 ± 0.001 | 0.001 ± 0.001 | 0.001 ± 0.001 | 0.011 ± 0.004 | 0.083 ± 0.023 |
| PC 15:0_18:1 | 790.5604 | [M+HCO2]- | 13.21 | 1.26 ± 0.02 | 1.09 ± 0.02 | 1.50 ± 0.02 | 1.30 ± 0.09 | 1.54 ± 0.10 | 2.62 ± 0.10 |
| PC 15:0_18:2 | 788.5442 | [M+HCO2]-\|[M+H]+ | 11.97 | 1.08 ± 0.02 | 1.35 ± 0.05 | 2.02 ± 0.01 | 1.14 ± 0.03 | 2.12 ± 0.03 | 1.03 ± 0.02 |
| PC 15:0_18:3 | 786.5279 | [M+HCO2]-\|[M+H]+ | 11.16 | 0.017 ± 0.004 | 0.032 ± 0.006 | 0.044 ± 0.008 | 0.047 ± 0.003 | 0.051 ± 0.002 | 0.027 ± 0.002 |
| PC 15:0_20:3 | 770.5713 | [M+H]+ | 12.54 | 0.007 ± 0.004 | 0.008 ± 0.009 | 0.018 ± 0.005 | 0.020 ± 0.007 | 0.028 ± 0.005 | 0.009 ± 0.001 |
| PC 15:0_20:4 | 812.5445 | [M+HCO2]-\|[M+H]+\|[M+Na]+ | 11.73 | 0.44 ± 0.01 | 0.55 ± 0.03 | 0.74 ± 0.02 | 0.40 ± 0.03 | 0.69 ± 0.03 | 0.67 ± 0.03 |
| PC 15:0_20:5 | 810.5289 | [M+HCO2]-\|[M+H]+\|[M+Na]+ | 10.93 | 0.034 ± 0.003 | 0.058 ± 0.002 | 0.081 ± 0.002 | 0.18 ± 0.004 | 0.052 ± 0.001 | 0.52 ± 0.01 |
| PC 15:0_22:5 | 838.5600 | [M+HCO2]- | 11.90 | 0.095 ± 0.004 | 0.11 ± 0.02 | 0.16 ± 0.02 | 0.29 ± 0.01 | 0.14 ± 0.03 | 0.58 ± 0.04 |
| PC 15:0_22:6 | 792.5533 | [M+H]+\|[M+Na]+ | 11.36 | 0.16 ± 0.003 | 0.30 ± 0.01 | 0.36 ± 0.01 | 0.41 ± 0.01 | 0.39 ± 0.03 | 0.59 ± 0.02 |
| PC 15:1_16:0 | 762.5278 | [M+HCO2]- | 12.21 | <0.001 | <0.001 | 0.001 ± 0.001 | <0.001 | 0.002 ± 0.001 | <0.001 |
| PC 16:0_16:0 | 778.5600 | [M+HCO2]-\|[M+H]+\|[M+Na]+ | 13.87 | 6.51 ± 0.15 | 6.87 ± 0.09 | 6.29 ± 0.08 | 7.90 ± 0.13 | 6.94 ± 0.24 | 9.63 ± 0.18 |
| PC 16:0_16:1 | 776.5446 | [M+HCO2]-\|[M+H]+ | 12.41 | 5.51 ± 0.03 | 3.36 ± 0.36 | 4.16 ± 0.08 | 4.34 ± 0.65 | 3.43 ± 0.20 | 8.72 ± 0.97 |
| PC 16:0_17:0 | 792.5755 | [M+HCO2]-\|[M+H]+ | 14.59 | 0.12 ± 0.004 | 0.16 ± 0.01 | 0.14 ± 0.01 | 0.17 ± 0.01 | 0.16 ± 0.02 | 0.20 ± 0.01 |
| PC 16:0_17:1 | 790.5596 | [M+HCO2]- | 13.62 | 0.013 ± 0.013 | 0.003 ± 0.003 | 0.006 ± <0.001 | 0.003 ± 0.002 | 0.013 ± 0.013 | 0.008 ± 0.003 |
| PC 16:0_18:0 | 806.5923 | [M+HCO2]-\|[M+H]+ | 15.21 | 1.91 ± 0.02 | 2.31 ± 0.39 | 2.34 ± 0.07 | 2.24 ± 0.45 | 1.49 ± 0.16 | 1.80 ± 0.21 |
| PC 16:0_18:1 | 804.5759 | [M+HCO2]-\|[M+Na]+ | 13.98 | 73.3 ± 0.7 | 57 ± 13 | 75.2 ± 1.5 | 64 ± 20 | 63 ± 14 | 75 ± 19 |
| PC 16:0_18:2 | 802.5600 | [M+HCO2]-\|[M+Na]+ | 12.71 | 113 ± 1 | 114 ± 13 | 136 ± 2 | 112 ± 22 | 131 ± 13 | 89 ± 15 |
| PC 16:0_18:3 | 800.5445 | [M+HCO2]-\|[M+H]+\|[M+Na]+ | 11.71 | 6.13 ± 0.19 | 5.58 ± 0.32 | 7.92 ± 0.20 | 7.56 ± 0.30 | 7.39 ± 0.35 | 4.18 ± 0.21 |
| PC 16:0_19:0 | 776.6171 | [M+H]+ | 15.33 | 0.002 ± <0.001 | 0.002 ± 0.001 | 0.001 ± <0.001 | 0.001 ± 0.001 | 0.013 ± 0.004 | 0.006 ± 0.001 |
| PC 16:0_20:3 | 828.5756 | [M+HCO2]- | 13.10 | 43.5 ± 1.7 | 36.3 ± 3.1 | 52.0 ± 0.6 | 31.3 ± 2.0 | 50.0 ± 3.8 | 29.3 ± 1.3 |
| PC 16:0_20:4 | 826.5603 | [M+HCO2]-\|[M+H]+ | 12.06 | 1.66 ± 0.03 | 1.39 ± 0.09 | 2.40 ± 0.06 | 1.96 ± 0.04 | 3.03 ± 0.16 | 2.28 ± 0.01 |
| PC 16:0_20:5 | 824.5455 | [M+HCO2]-\|[M+H]+\|[M+Na]+ | 11.51 | 6.06 ± 0.12 | 8.90 ± 0.20 | 9.66 ± 0.15 | 28.1 ± 0.4 | 7.61 ± 0.17 | 49.6 ± 0.5 |
| PC 16:0_22:4 | 854.5910 | [M+HCO2]-\|[M+H]+\|[M+Na]+ | 13.57 | 5.00 ± 0.04 | 3.08 ± 0.50 | 4.77 ± 0.12 | 2.35 ± 0.42 | 3.55 ± 0.47 | 2.73 ± 0.36 |
| PC 16:0_22:5 | 808.5856 | [M+H]+ | 12.53 | 11.4 ± 0.7 | 11.2 ± 1.0 | 13.5 ± 0.0 | 14.4 ± 2.2 | 13.6 ± 0.5 | 19.5 ± 3.6 |
| PC 16:0_22:6 | 850.5587 | [M+HCO2]-\|[M+Na]+ | 11.99 | 20.0 ± 0.2 | 29.0 ± 1.4 | 28.8 ± 0.2 | 45.0 ± 4.4 | 31.2 ± 1.7 | 46.8 ± 3.6 |
| PC 16:0_24:0 | 890.6850 | [M+HCO2]- | 17.90 | 0.007 ± 0.003 | 0.014 ± 0.002 | 0.011 ± 0.001 | 0.015 ± 0.002 | 0.012 ± 0.001 | 0.014 ± 0.002 |
| PC 16:1_16:1 | 774.5281 | [M+HCO2]- | 12.86 | 0.35 ± 0.04 | 0.24 ± 0.11 | 0.44 ± 0.09 | 0.35 ± 0.14 | 0.44 ± 0.11 | 0.53 ± 0.16 |
| PC 16:1_18:2 | 800.5443 | [M+HCO2]-\|[M+H]+\|[M+Na]+ | 11.45 | 2.46 ± 0.10 | 2.99 ± 0.40 | 3.79 ± 0.07 | 2.69 ± 0.22 | 4.42 ± 0.42 | 1.48 ± 0.14 |
| PC 16:1_20:4 | 824.5444 | [M+HCO2]-\|[M+H]+\|[M+Na]+ | 11.29 | 1.01 ± 0.04 | 1.31 ± 0.17 | 1.40 ± 0.06 | 1.48 ± 0.63 | 1.51 ± 0.15 | 1.78 ± 0.77 |
| PC 16:1_20:5 | 822.5289 | [M+HCO2]-\|[M+H]+ | 10.50 | 0.060 ± 0.003 | 0.096 ± 0.018 | 0.11 ± 0.01 | 0.30 ± 0.03 | 0.089 ± 0.010 | 0.45 ± 0.02 |
| PC 16:1_22:6 | 848.5442 | [M+HCO2]-\|[M+H]+\|[M+Na]+ | 10.90 | 0.29 ± 0.02 | 0.49 ± 0.01 | 0.52 ± 0.02 | 0.71 ± 0.03 | 0.57 ± 0.01 | 0.66 ± 0.03 |
| PC 17:0_18:0 | 820.6078 | [M+HCO2]- | 15.76 | 0.023 ± 0.005 | 0.029 ± 0.007 | 0.026 ± 0.005 | 0.027 ± 0.006 | 0.017 ± 0.007 | 0.032 ± 0.005 |
| PC 17:0_18:1 | 818.5913 | [M+HCO2]-\|[M+H]+ | 14.68 | 1.60 ± 0.04 | 1.29 ± 0.23 | 1.87 ± 0.01 | 1.49 ± 0.28 | 1.94 ± 0.19 | 2.50 ± 0.36 |
| PC 17:0_18:2 | 816.5755 | [M+HCO2]-\|[M+H]+ | 13.54 | 2.93 ± 0.07 | 2.66 ± 0.48 | 3.99 ± 0.07 | 2.68 ± 0.56 | 4.36 ± 0.65 | 2.41 ± 0.40 |
| PC 17:0_20:3 | 842.5924 | [M+HCO2]-\|[M+H]+ | 13.89 | 0.49 ± 0.004 | 0.38 ± 0.05 | 0.71 ± 0.02 | 0.30 ± 0.02 | 0.82 ± 0.07 | 0.40 ± 0.05 |
| PC 17:0_20:4 | 840.5748 | [M+HCO2]-\|[M+H]+ | 13.23 | 1.07 ± 0.01 | 0.91 ± 0.18 | 1.26 ± 0.04 | 0.70 ± 0.19 | 1.19 ± 0.20 | 1.11 ± 0.27 |
| PC 17:0_20:5 | 838.5602 | [M+HCO2]-\|[M+H]+\|[M+Na]+ | 12.18 | 0.15 ± 0.01 | 0.20 ± 0.02 | 0.24 ± 0.02 | 0.53 ± 0.05 | 0.25 ± 0.01 | 1.35 ± 0.03 |
| PC 17:0_22:5 | 866.5913 | [M+HCO2]-\|[M+H]+ | 13.24 | 0.18 ± 0.005 | 0.16 ± 0.02 | 0.21 ± 0.01 | 0.21 ± 0.03 | 0.19 ± 0.02 | 0.37 ± 0.03 |
| PC 17:0_22:6 | 820.5840 | [M+H]+ | 12.73 | 0.069 ± 0.008 | 0.11 ± 0.01 | 0.14 ± 0.01 | 0.17 ± 0.02 | 0.12 ± 0.01 | 0.35 ± 0.04 |
| PC 17:1_18:0 | 774.6017 | [M+H]+ | 15.45 | 0.011 ± 0.002 | 0.011 ± 0.006 | 0.018 ± 0.002 | 0.005 ± 0.003 | 0.017 ± 0.002 | 0.014 ± 0.004 |
| PC 17:1_18:1 | 816.5769 | [M+HCO2]- | 14.21 | 0.002 ± 0.003 | <0.001 | <0.001 | <0.001 | <0.001 | 0.001 ± 0.001 |
| PC 17:1_18:2 | 814.5608 | [M+HCO2]-\|[M+H]+ | 12.16 | 0.49 ± 0.03 | 0.49 ± 0.02 | 0.76 ± 0.01 | 0.54 ± 0.03 | 0.92 ± 0.01 | 0.41 ± 0.03 |
| PC 17:1_20:5 | 836.5445 | [M+HCO2]-\|[M+H]+ | 11.10 | 0.003 ± 0.002 | 0.011 ± 0.001 | 0.007 ± 0.002 | 0.038 ± 0.005 | 0.010 ± 0.002 | 0.064 ± 0.003 |
| PC 17:1_22:6 | 862.5609 | [M+HCO2]- | 11.47 | 0.042 ± 0.011 | 0.022 ± 0.002 | 0.024 ± 0.005 | 0.054 ± 0.012 | 0.028 ± 0.010 | 0.044 ± 0.003 |
| PC 17:2_18:2 | 768.5542 | [M+H]+ | 11.16 | 0.007 ± <0.001 | 0.011 ± 0.001 | 0.013 ± 0.001 | 0.018 ± 0.001 | 0.017 ± 0.003 | 0.008 ± <0.001 |
| PC 18:0_18:0 | 834.6245 | [M+HCO2]-\|[M+H]+ | 16.24 | 0.14 ± 0.005 | 0.19 ± 0.01 | 0.21 ± 0.005 | 0.18 ± 0.02 | 0.12 ± 0.01 | 0.14 ± 0.01 |
| PC 18:0_18:1 | 832.6078 | [M+HCO2]-\|[M+H]+\|[M+Na]+ | 15.28 | 24.0 ± 0.2 | 20.4 ± 2.7 | 26.7 ± 0.6 | 22.8 ± 3.4 | 19.9 ± 2.4 | 26.2 ± 3.9 |
| PC 18:0_18:2 | 830.5922 | [M+HCO2]- | 14.28 | 94.2 ± 0.6 | 94 ± 11 | 124 ± 2 | 90 ± 18 | 98 ± 10 | 66 ± 9 |
| PC 18:0_20:1 | 860.6385 | [M+HCO2]-\|[M+H]+ | 16.22 | 0.090 ± 0.010 | 0.10 ± 0.01 | 0.14 ± 0.01 | 0.13 ± 0.01 | 0.091 ± 0.013 | 0.15 ± 0.03 |
| PC 18:0_20:2 | 858.6222 | [M+HCO2]-\|[M+H]+ | 15.40 | 2.82 ± 0.03 | 2.76 ± 0.17 | 3.48 ± 0.06 | 2.75 ± 0.31 | 3.68 ± 0.23 | 2.32 ± 0.09 |
| PC 18:0_20:3 | 856.6076 | [M+HCO2]-\|[M+H]+ | 15.01 | 1.39 ± 0.02 | 0.97 ± 0.17 | 1.32 ± 0.05 | 0.70 ± 0.12 | 0.91 ± 0.10 | 0.87 ± 0.10 |
| PC 18:0_20:4 | 854.5918 | [M+HCO2]-\|[M+H]+ | 30.37 | 0.006 ± 0.004 | 0.006 ± 0.004 | 0.009 ± 0.003 | 0.009 ± 0.003 | 0.005 ± 0.001 | 0.010 ± 0.005 |
| PC 18:0_20:5 | 852.5762 | [M+HCO2]-\|[M+H]+\|[M+Na]+ | 12.96 | 6.18 ± 0.04 | 5.92 ± 0.73 | 7.19 ± 0.04 | 13.4 ± 1.9 | 6.04 ± 0.23 | 23.2 ± 3.5 |
| PC 18:0_22:4 | 882.6226 | [M+HCO2]-\|[M+H]+\|[M+Na]+ | 14.94 | 3.00 ± 0.05 | 1.98 ± 0.05 | 2.81 ± 0.05 | 1.41 ± 0.04 | 1.85 ± 0.04 | 1.66 ± 0.05 |
| PC 18:0_22:5 | 880.6067 | [M+HCO2]-\|[M+H]+ | 14.03 | 2.45 ± 0.07 | 1.92 ± 0.05 | 3.21 ± 0.06 | 3.66 ± 0.10 | 2.01 ± 0.07 | 5.29 ± 0.06 |
| PC 18:0_22:6 | 878.5904 | [M+HCO2]-\|[M+H]+\|[M+Na]+ | 13.54 | 8.31 ± 0.24 | 10.4 ± 1.4 | 13.9 ± 0.4 | 18.2 ± 3.1 | 9.48 ± 1.13 | 19.0 ± 2.2 |
| PC 18:1_18:2 | 828.5753 | [M+HCO2]-\|[M+H]+\|[M+Na]+ | 12.87 | 13.5 ± 1.1 | 15.5 ± 0.8 | 18.3 ± 0.5 | 15.7 ± 0.9 | 21.7 ± 0.6 | 10.2 ± 0.8 |
| PC 18:1_19:0 | 846.6226 | [M+HCO2]- | 15.94 | 0.091 ± 0.013 | 0.069 ± 0.015 | 0.093 ± 0.021 | 0.074 ± 0.032 | 0.072 ± 0.021 | 0.067 ± 0.025 |
| PC 18:1_20:3 | 854.5906 | [M+HCO2]-\|[M+H]+\|[M+Na]+ | 13.29 | 3.53 ± 0.20 | 2.98 ± 0.23 | 4.51 ± 0.01 | 2.23 ± 0.03 | 5.24 ± 0.12 | 2.03 ± 0.04 |
| PC 18:1_20:4 | 852.5750 | [M+HCO2]-\|[M+Na]+ | 12.53 | 11.5 ± 0.4 | 11.0 ± 0.4 | 13.7 ± 0.2 | 14.0 ± 0.3 | 12.4 ± 0.2 | 19.0 ± 1.7 |
| PC 18:1_22:0 | 888.6707 | [M+HCO2]-\|[M+H]+ | 17.13 | 0.046 ± 0.003 | 0.065 ± 0.010 | 0.047 ± 0.003 | 0.066 ± 0.010 | 0.056 ± 0.007 | 0.075 ± 0.013 |
| PC 18:1_22:1 | 886.6557 | [M+HCO2]-\|[M+H]+ | 16.30 | 0.027 ± 0.004 | 0.047 ± 0.014 | 0.026 ± 0.001 | 0.056 ± 0.014 | 0.055 ± 0.007 | 0.059 ± 0.002 |
| PC 18:1_22:5 | 878.5916 | [M+HCO2]-\|[M+H]+ | 12.56 | 0.36 ± 0.06 | 0.28 ± 0.03 | 0.44 ± 0.03 | 0.38 ± 0.04 | 0.41 ± 0.01 | 0.47 ± 0.06 |
| PC 18:1_22:6 | 876.5746 | [M+HCO2]-\|[M+H]+ | 12.09 | 1.72 ± 0.09 | 2.21 ± 0.07 | 2.48 ± 0.03 | 3.02 ± 0.03 | 3.13 ± 0.06 | 2.41 ± 0.04 |
| PC 18:1_24:0 | 916.7009 | [M+HCO2]-\|[M+H]+ | 17.93 | 0.027 ± 0.003 | 0.035 ± 0.005 | 0.024 ± 0.004 | 0.043 ± 0.001 | 0.034 ± <0.001 | 0.055 ± 0.001 |
| PC 18:1_26:0 | 900.7440 | [M+H]+ | 18.75 | 0.004 ± 0.002 | 0.004 ± 0.002 | 0.005 ± 0.002 | <0.001 | 0.007 ± <0.001 | 0.010 ± 0.003 |
| PC 18:2_18:2 | 826.5590 | [M+HCO2]-\|[M+H]+\|[M+Na]+ | 11.68 | 9.25 ± 0.65 | 14.9 ± 0.5 | 14.1 ± 0.3 | 14.2 ± 0.9 | 17.5 ± 0.4 | 6.55 ± 0.33 |
| PC 18:2_18:3 | 824.5444 | [M+HCO2]-\|[M+H]+\|[M+Na]+ | 10.85 | 0.32 ± 0.01 | 0.53 ± 0.05 | 0.57 ± 0.01 | 0.80 ± 0.10 | 0.74 ± 0.07 | 0.23 ± 0.02 |
| PC 18:2_19:0 | 844.6075 | [M+HCO2]-\|[M+H]+ | 14.94 | 0.62 ± 0.01 | 0.80 ± 0.02 | 0.85 ± 0.02 | 0.86 ± 0.06 | 1.12 ± 0.07 | 0.83 ± 0.04 |
| PC 18:2_20:1 | 856.6050 | [M+HCO2]-\|[M+H]+ | 14.25 | 1.14 ± 0.11 | 1.11 ± 0.16 | 1.32 ± 0.06 | 1.15 ± 0.19 | 2.03 ± 0.32 | 1.01 ± 0.18 |
| PC 18:2_20:4 | 850.5593 | [M+HCO2]-\|[M+H]+\|[M+Na]+ | 11.45 | 3.67 ± 0.27 | 5.68 ± 0.12 | 5.86 ± 0.08 | 5.69 ± 0.15 | 5.60 ± 0.12 | 6.30 ± 0.08 |
| PC 18:2_20:5 | 848.5442 | [M+HCO2]-\|[M+H]+\|[M+Na]+ | 10.67 | 0.17 ± 0.01 | 0.42 ± 0.03 | 0.40 ± 0.01 | 1.27 ± 0.03 | 0.38 ± 0.03 | 1.47 ± 0.02 |
| PC 18:2_21:0 | 872.6393 | [M+HCO2]- | 16.04 | 0.012 ± 0.014 | 0.034 ± 0.020 | 0.046 ± 0.017 | 0.007 ± 0.002 | 0.012 ± 0.012 | 0.019 ± 0.017 |
| PC 18:2_22:0 | 842.6632 | [M+H]+ | 16.48 | 0.073 ± 0.001 | 0.11 ± 0.01 | 0.077 ± 0.006 | 0.096 ± 0.003 | 0.091 ± 0.007 | 0.092 ± 0.009 |
| PC 18:2_22:1 | 884.6383 | [M+HCO2]-\|[M+H]+ | 15.46 | 0.081 ± 0.005 | 0.099 ± 0.008 | 0.082 ± 0.013 | 0.12 ± 0.01 | 0.14 ± 0.01 | 0.12 ± 0.003 |
| PC 18:2_22:5 | 876.5758 | [M+HCO2]- | 11.47 | 0.065 ± 0.007 | 0.071 ± 0.011 | 0.093 ± 0.008 | 0.091 ± 0.014 | 0.076 ± 0.011 | 0.049 ± 0.008 |
| PC 18:2_22:6 | 874.5595 | [M+HCO2]- | 11.14 | 0.52 ± 0.03 | 0.75 ± 0.10 | 0.71 ± 0.03 | 0.77 ± 0.13 | 0.67 ± 0.08 | 0.56 ± 0.10 |
| PC 18:2_24:0 | 914.6855 | [M+HCO2]-\|[M+H]+ | 17.30 | 0.059 ± 0.002 | 0.11 ± 0.003 | 0.069 ± 0.007 | 0.11 ± 0.003 | 0.090 ± 0.005 | 0.091 ± 0.002 |
| PC 18:2_24:1 | 912.6714 | [M+HCO2]-\|[M+H]+ | 16.41 | 0.017 ± 0.004 | 0.028 ± 0.004 | 0.015 ± 0.007 | 0.050 ± 0.008 | 0.048 ± 0.001 | 0.048 ± 0.010 |
| PC 18:2_26:0 | 942.7162 | [M+HCO2]-\|[M+H]+ | 18.09 | 0.006 ± 0.003 | 0.012 ± 0.004 | 0.008 ± 0.002 | 0.014 ± 0.003 | 0.008 ± 0.001 | 0.014 ± 0.002 |
| PC 18:3_18:3 | 778.5391 | [M+H]+ | 10.07 | 0.001 ± <0.001 | 0.001 ± <0.001 | 0.005 ± 0.001 | 0.023 ± 0.002 | 0.006 ± 0.002 | 0.001 ± <0.001 |
| PC 18:3_20:5 | 846.5283 | [M+HCO2]- | 10.25 | <0.001 | <0.001 | <0.001 | 0.004 ± 0.001 | <0.001 | <0.001 |
| PC 19:0_20:3 | 870.6206 | [M+HCO2]- | 15.22 | 0.032 ± 0.003 | 0.033 ± 0.006 | 0.063 ± 0.007 | 0.029 ± 0.002 | 0.067 ± 0.020 | 0.045 ± 0.011 |
| PC 19:0_20:4 | 868.6080 | [M+HCO2]-\|[M+H]+ | 14.69 | 0.15 ± 0.01 | 0.19 ± 0.02 | 0.19 ± 0.002 | 0.17 ± 0.01 | 0.24 ± 0.02 | 0.25 ± 0.01 |
| PC 19:0_22:6 | 848.6155 | [M+HCO2]-\|[M+H]+ | 14.26 | <0.001 | <0.001 | <0.001 | 0.002 ± 0.001 | <0.001 | 0.008 ± 0.003 |
| PC 20:0_20:3 | 884.6379 | [M+HCO2]-\|[M+H]+ | 15.77 | 0.14 ± 0.01 | 0.11 ± 0.006 | 0.16 ± 0.01 | 0.086 ± 0.005 | 0.17 ± 0.01 | 0.12 ± 0.01 |
| PC 20:0_20:4 | 882.6233 | [M+HCO2]- | 15.28 | 0.23 ± 0.01 | 0.27 ± 0.03 | 0.28 ± 0.004 | 0.22 ± 0.04 | 0.27 ± 0.03 | 0.20 ± 0.04 |
| PC 20:0_22:4 | 866.6651 | [M+H]+ | 15.93 | 0.013 ± 0.001 | 0.012 ± 0.004 | 0.014 ± 0.003 | 0.009 ± <0.001 | 0.014 ± 0.005 | 0.010 ± 0.003 |
| PC 20:0_22:6 | 906.6214 | [M+HCO2]-\|[M+H]+ | 14.92 | 0.020 ± 0.004 | 0.044 ± 0.005 | 0.037 ± 0.004 | 0.092 ± 0.011 | 0.044 ± 0.002 | 0.096 ± 0.009 |
| PC 20:1_22:5 | 862.6332 | [M+H]+ | 14.13 | 0.007 ± 0.001 | 0.006 ± 0.001 | 0.006 ± 0.002 | 0.008 ± 0.003 | 0.012 ± 0.005 | 0.013 ± 0.002 |
| PC 20:1_22:6 | 904.6068 | [M+HCO2]- | 13.54 | 0.029 ± 0.005 | 0.052 ± 0.015 | 0.053 ± 0.001 | 0.11 ± 0.02 | 0.17 ± 0.02 | 0.11 ± 0.03 |
| PC 20:2_20:3 | 880.6062 | [M+HCO2]-\|[M+H]+ | 13.38 | 0.003 ± 0.003 | 0.001 ± 0.002 | 0.004 ± 0.003 | <0.001 | 0.012 ± 0.020 | <0.001 |
| PC 20:2_22:5 | 904.6068 | [M+HCO2]- | 13.32 | <0.001 | <0.001 | <0.001 | 0.001 ± 0.002 | 0.004 ± 0.003 | 0.001 ± 0.001 |
| PC 20:2_22:6 | 858.6014 | [M+H]+ | 12.16 | 0.063 ± 0.002 | 0.084 ± 0.015 | 0.096 ± 0.004 | 0.083 ± 0.004 | 0.11 ± 0.01 | 0.074 ± 0.008 |
| PC 20:3_20:4 | 876.5754 | [M+HCO2]- | 11.80 | 0.20 ± 0.01 | 0.27 ± 0.04 | 0.31 ± 0.01 | 0.27 ± 0.07 | 0.35 ± 0.07 | 0.26 ± 0.03 |
| PC 20:3_22:0 | 912.6683 | [M+HCO2]- | 16.71 | 0.001 ± 0.001 | 0.001 ± 0.001 | <0.001 | 0.001 ± 0.001 | 0.003 ± 0.003 | 0.002 ± 0.002 |
| PC 20:4_20:4 | 830.5703 | [M+H]+ | 11.14 | 0.52 ± 0.04 | 0.81 ± 0.09 | 0.77 ± 0.03 | 0.91 ± 0.09 | 0.73 ± 0.11 | 0.72 ± 0.08 |
| PC 20:4_20:5 | 872.5453 | [M+HCO2]-\|[M+H]+ | 10.40 | 0.014 ± 0.002 | 0.034 ± 0.003 | 0.030 ± 0.002 | 0.096 ± 0.007 | 0.021 ± 0.003 | 0.12 ± 0.004 |
| PC 20:4_22:0 | 910.6522 | [M+HCO2]-\|[M+H]+ | 16.29 | 0.026 ± 0.005 | 0.053 ± 0.010 | 0.026 ± 0.004 | 0.032 ± 0.009 | 0.025 ± 0.004 | 0.035 ± 0.012 |
| PC 20:4_22:1 | 908.6376 | [M+HCO2]-\|[M+H]+ | 15.18 | 0.071 ± 0.010 | 0.076 ± 0.013 | 0.084 ± 0.017 | 0.094 ± 0.011 | 0.079 ± 0.018 | 0.13 ± 0.01 |
| PC 20:4_22:3 | 904.6057 | [M+HCO2]-\|[M+H]+ | 13.00 | 0.002 ± 0.004 | <0.001 | <0.001 | <0.001 | 0.002 ± 0.004 | <0.001 |
| PC 20:4_22:4 | 902.5922 | [M+HCO2]- | 12.17 | 0.086 ± 0.009 | 0.072 ± 0.018 | 0.083 ± 0.006 | 0.059 ± 0.006 | 0.098 ± 0.011 | 0.043 ± 0.003 |
| PC 20:4_22:5 | 900.5764 | [M+HCO2]-\|[M+H]+ | 11.23 | 0.043 ± 0.004 | 0.055 ± 0.008 | 0.061 ± 0.011 | 0.10 ± 0.003 | 0.053 ± 0.010 | 0.10 ± 0.01 |
| PC 20:4_22:6 | 898.5610 | [M+HCO2]-\|[M+H]+\|[M+Na]+ | 10.84 | 0.077 ± 0.004 | 0.19 ± 0.005 | 0.15 ± 0.001 | 0.24 ± 0.01 | 0.15 ± 0.002 | 0.18 ± 0.002 |
| PC 20:4_24:0 | 894.6965 | [M+H]+ | 17.13 | 0.026 ± <0.001 | 0.040 ± 0.001 | 0.027 ± <0.001 | 0.035 ± 0.002 | 0.034 ± 0.002 | 0.044 ± 0.001 |
| PC 20:4_24:1 | 936.6719 | [M+HCO2]-\|[M+H]+ | 16.21 | 0.024 ± 0.003 | 0.026 ± 0.002 | 0.020 ± 0.001 | 0.040 ± 0.002 | 0.040 ± 0.005 | 0.055 ± 0.004 |
| PC 20:5_22:0 | 864.6490 | [M+H]+ | 15.67 | 0.002 ± 0.001 | 0.004 ± 0.002 | 0.003 ± 0.001 | 0.014 ± 0.001 | 0.006 ± 0.001 | 0.032 ± 0.001 |
| PC 20:5_22:5 | 854.5688 | [M+H]+ | 10.48 | <0.001 | <0.001 | <0.001 | 0.011 ± 0.001 | <0.001 | 0.014 ± 0.001 |
| PC 20:5_22:6 | 896.5445 | [M+HCO2]-\|[M+H]+ | 10.08 | <0.001 | 0.004 ± 0.002 | 0.003 ± 0.001 | 0.052 ± 0.005 | 0.002 ± <0.001 | 0.064 ± 0.001 |
| PC 22:0_22:6 | 934.6539 | [M+HCO2]- | 15.99 | 0.001 ± <0.001 | 0.003 ± 0.001 | <0.001 | 0.006 ± 0.002 | 0.001 ± 0.001 | 0.005 ± 0.002 |
| PC 22:5_22:6 | 880.5866 | [M+H]+ | 10.90 | 0.002 ± <0.001 | 0.005 ± 0.001 | 0.004 ± <0.001 | 0.016 ± 0.001 | 0.007 ± 0.001 | 0.019 ± 0.001 |
| PC 22:5_24:0 | 920.7114 | [M+H]+ | 17.08 | 0.005 ± 0.002 | 0.006 ± 0.001 | 0.005 ± 0.001 | 0.010 ± <0.001 | 0.007 ± <0.001 | 0.016 ± 0.001 |
| PC 22:6_22:6 | 922.5603 | [M+HCO2]- | 10.52 | <0.001 | 0.008 ± 0.001 | 0.002 ± 0.001 | 0.023 ± 0.001 | 0.005 ± 0.001 | 0.021 ± 0.001 |
| PC 22:6_24:0 | 962.6842 | [M+HCO2]- | 16.84 | <0.001 | 0.001 ± 0.001 | <0.001 | 0.001 ± 0.001 | <0.001 | 0.002 ± 0.003 |
| PE 16:0_16:1 | 688.4925 | [M-H]- | 12.86 | 0.15 ± 0.02 | 0.073 ± 0.022 | 0.11 ± 0.01 | 0.11 ± 0.03 | 0.10 ± 0.02 | 0.47 ± 0.11 |
| PE 16:0_18:0 | 718.5386 | [M-H]- | 13.88 | 0.029 ± 0.001 | 0.028 ± 0.002 | 0.025 ± 0.001 | 0.036 ± 0.003 | 0.026 ± 0.005 | 0.050 ± 0.008 |
| PE 16:0_18:1 | 716.5220 | [M-H]- | 11.44 | 0.012 ± 0.005 | 0.013 ± 0.006 | 0.025 ± 0.009 | 0.032 ± 0.002 | 0.036 ± 0.004 | 0.019 ± 0.005 |
| PE 16:0_18:2 | 714.5075 | [M-H]-\|[M+H]+ | 13.20 | 0.89 ± 0.02 | 0.83 ± 0.02 | 1.01 ± 0.01 | 1.17 ± 0.04 | 1.56 ± 0.03 | 1.09 ± 0.005 |
| PE 16:0_18:3 | 712.4928 | [M-H]-\|[M+H]+ | 12.09 | 0.022 ± 0.002 | 0.021 ± 0.001 | 0.028 ± 0.001 | 0.048 ± 0.002 | 0.069 ± 0.004 | 0.026 ± 0.001 |
| PE 16:0_20:4 | 738.5088 | [M-H]- | 15.01 | 0.023 ± 0.002 | 0.005 ± 0.001 | <0.001 | 0.009 ± 0.001 | 0.029 ± 0.007 | 0.034 ± 0.006 |
| PE 16:0_20:5 | 736.4921 | [M-H]-\|[M+H]+ | 11.86 | 0.024 ± 0.001 | 0.035 ± 0.003 | 0.027 ± 0.001 | 0.21 ± 0.01 | 0.045 ± 0.004 | 0.64 ± 0.03 |
| PE 16:0_22:4 | 766.5389 | [M-H]- | 14.01 | 0.12 ± 0.01 | 0.057 ± 0.006 | 0.070 ± 0.003 | 0.051 ± 0.001 | 0.095 ± 0.009 | 0.065 ± 0.006 |
| PE 16:0_22:6 | 762.5072 | [M-H]-\|[M+H]+ | 12.42 | 0.87 ± 0.07 | 1.61 ± 0.12 | 1.54 ± 0.12 | 2.17 ± 0.17 | 3.18 ± 0.12 | 3.74 ± 0.23 |
| PE 16:1_20:4 | 736.4924 | [M-H]- | 11.55 | 0.004 ± 0.001 | 0.008 ± 0.004 | 0.003 ± 0.003 | 0.001 ± 0.001 | 0.008 ± 0.001 | 0.002 ± 0.002 |
| PE 16:1_20:5 | 734.4758 | [M-H]- | 10.77 | <0.001 | <0.001 | <0.001 | <0.001 | <0.001 | 0.003 ± 0.001 |
| PE 16:1_22:6 | 762.5083 | [M+H]+ | 11.29 | <0.001 | 0.001 ± 0.001 | <0.001 | <0.001 | 0.001 ± 0.001 | 0.002 ± 0.003 |
| PE 17:0_18:1 | 730.5393 | [M-H]- | 15.06 | 0.007 ± 0.002 | 0.007 ± 0.003 | 0.006 ± 0.002 | 0.005 ± 0.003 | 0.005 ± 0.002 | 0.002 ± 0.001 |
| PE 17:0_18:2 | 728.5240 | [M-H]- | 14.00 | 0.026 ± 0.001 | 0.017 ± 0.009 | 0.035 ± 0.006 | 0.029 ± 0.012 | 0.040 ± 0.007 | 0.026 ± 0.008 |
| PE 17:0_20:4 | 752.5233 | [M-H]-\|[M+H]+ | 13.70 | 0.040 ± 0.002 | 0.036 ± 0.008 | 0.049 ± 0.002 | 0.026 ± 0.006 | 0.064 ± 0.012 | 0.048 ± 0.010 |
| PE 17:0_20:5 | 752.5219 | [M+H]+ | 12.20 | <0.001 | <0.001 | <0.001 | <0.001 | <0.001 | <0.001 |
| PE 17:0_22:6 | 778.5390 | [M+H]+ | 13.23 | 0.002 ± 0.001 | 0.007 ± 0.003 | 0.009 ± 0.004 | 0.021 ± 0.004 | 0.036 ± 0.006 | 0.064 ± 0.008 |
| PE 18:0_18:1 | 744.5554 | [M-H]-\|[M+H]+ | 15.61 | 0.58 ± 0.01 | 0.66 ± 0.08 | 0.80 ± 0.02 | 0.91 ± 0.06 | 0.96 ± 0.08 | 0.82 ± 0.03 |
| PE 18:0_18:2 | 742.5399 | [M-H]-\|[M+Na]+ | 14.68 | 2.63 ± 0.02 | 2.46 ± 0.29 | 3.36 ± 0.03 | 3.03 ± 0.28 | 3.81 ± 0.16 | 2.05 ± 0.14 |
| PE 18:0_18:3 | 740.5240 | [M-H]- | 13.89 | 0.030 ± 0.003 | 0.016 ± 0.008 | 0.024 ± 0.001 | 0.023 ± 0.007 | 0.019 ± 0.009 | 0.021 ± 0.003 |
| PE 18:0_20:1 | 772.5851 | [M-H]- | 16.55 | 0.001 ± <0.001 | 0.006 ± <0.001 | 0.003 ± 0.002 | 0.005 ± 0.002 | 0.019 ± 0.007 | 0.007 ± 0.004 |
| PE 18:0_20:3 | 768.5544 | [M-H]-\|[M+H]+ | 14.97 | 0.32 ± 0.01 | 0.23 ± 0.03 | 0.45 ± 0.01 | 0.21 ± 0.04 | 0.61 ± 0.06 | 0.27 ± 0.04 |
| PE 18:0_20:4 | 766.5395 | [M-H]-\|[M+H]+ | 14.42 | 3.35 ± 0.05 | 3.21 ± 0.41 | 4.26 ± 0.09 | 2.51 ± 0.40 | 4.69 ± 0.43 | 2.76 ± 0.34 |
| PE 18:0_20:5 | 764.5235 | [M-H]-\|[M+H]+ | 13.43 | 0.40 ± 0.03 | 0.36 ± 0.04 | 0.35 ± 0.01 | 0.77 ± 0.01 | 0.61 ± 0.02 | 1.50 ± 0.05 |
| PE 18:0_22:3 | 796.5865 | [M-H]- | 16.03 | 0.015 ± <0.001 | 0.020 ± 0.001 | 0.020 ± 0.001 | 0.023 ± <0.001 | 0.029 ± 0.001 | 0.027 ± 0.001 |
| PE 18:0_22:4 | 794.5714 | [M-H]-\|[M+H]+ | 15.28 | 0.090 ± 0.008 | 0.027 ± 0.008 | 0.069 ± 0.015 | 0.022 ± 0.002 | 0.043 ± 0.007 | 0.085 ± 0.001 |
| PE 18:0_22:5 | 792.5559 | [M-H]-\|[M+H]+ | 14.42 | 0.16 ± 0.004 | 0.10 ± 0.02 | 0.20 ± 0.01 | 0.23 ± 0.03 | 0.22 ± 0.02 | 0.55 ± 0.10 |
| PE 18:0_22:6 | 790.5395 | [M-H]-\|[M+H]+ | 13.99 | 0.70 ± 0.02 | 0.71 ± 0.22 | 1.09 ± 0.04 | 1.44 ± 0.45 | 1.17 ± 0.35 | 1.85 ± 0.58 |
| PE 18:1_18:1 | 742.5387 | [M-H]- | 14.54 | 0.24 ± 0.01 | 0.48 ± 0.24 | 0.36 ± 0.02 | 0.79 ± 0.23 | 0.93 ± 0.17 | 0.48 ± 0.10 |
| PE 18:1_18:2 | 740.5236 | [M-H]-\|[M+H]+ | 13.29 | 0.79 ± 0.05 | 0.80 ± 0.07 | 0.93 ± 0.01 | 1.14 ± 0.09 | 1.50 ± 0.10 | 0.59 ± 0.03 |
| PE 18:1_20:3 | 768.5548 | [M+H]+ | 14.02 | <0.001 | <0.001 | <0.001 | <0.001 | <0.001 | <0.001 |
| PE 18:1_20:4 | 764.5240 | [M-H]-\|[M+H]+ | 12.87 | 1.46 ± 0.14 | 1.37 ± 0.19 | 1.82 ± 0.08 | 1.54 ± 0.15 | 2.46 ± 0.13 | 3.15 ± 0.58 |
| PE 18:1_20:5 | 762.5072 | [M-H]- | 12.06 | 0.002 ± 0.002 | 0.002 ± 0.001 | 0.003 ± 0.001 | 0.006 ± 0.005 | 0.002 ± <0.001 | 0.010 ± 0.003 |
| PE 18:1_22:5 | 790.5399 | [M-H]- | 12.89 | 0.024 ± 0.001 | 0.013 ± 0.006 | 0.027 ± 0.007 | 0.025 ± 0.012 | 0.007 ± 0.003 | 0.079 ± 0.014 |
| PE 18:1_22:6 | 788.5237 | [M-H]- | 12.50 | 0.13 ± 0.01 | 0.24 ± 0.03 | 0.23 ± 0.01 | 0.34 ± 0.01 | 0.59 ± 0.01 | 0.41 ± 0.02 |
| PE 18:2_18:2 | 738.5076 | [M-H]- | 12.20 | 0.096 ± 0.013 | 0.13 ± 0.01 | 0.12 ± 0.01 | 0.21 ± 0.02 | 0.15 ± 0.02 | 0.079 ± 0.034 |
| PE 18:2_20:1 | 768.5561 | [M-H]- | 14.61 | 0.30 ± 0.03 | 0.18 ± 0.17 | 0.25 ± 0.14 | 0.10 ± 0.06 | 0.25 ± 0.17 | 0.089 ± 0.094 |
| PE 18:2_20:4 | 762.5073 | [M-H]-\|[M+H]+ | 11.80 | 0.060 ± 0.007 | 0.073 ± 0.014 | 0.063 ± 0.004 | 0.13 ± 0.01 | 0.075 ± 0.007 | 0.16 ± 0.02 |
| PE 18:2_20:5 | 762.5071 | [M+H]+ | 10.98 | 0.001 ± <0.001 | <0.001 | 0.001 ± 0.001 | 0.005 ± 0.005 | 0.001 ± <0.001 | <0.001 |
| PE 18:2_22:5 | 788.5237 | [M-H]- | 11.81 | <0.001 | <0.001 | <0.001 | <0.001 | <0.001 | <0.001 |
| PE 18:2_22:6 | 786.5091 | [M-H]- | 11.44 | 0.012 ± 0.001 | 0.022 ± 0.006 | 0.019 ± 0.001 | 0.035 ± 0.005 | 0.020 ± 0.001 | 0.009 ± 0.003 |
| PE 19:0_20:4 | 780.5548 | [M-H]- | 14.72 | 0.009 ± 0.005 | 0.007 ± 0.004 | 0.002 ± 0.002 | 0.044 ± 0.002 | 0.012 ± 0.002 | 0.053 ± 0.015 |
| PE 20:0_20:4 | 796.5855 | [M+H]+ | 15.65 | <0.001 | <0.001 | <0.001 | <0.001 | <0.001 | <0.001 |
| PE 20:4_20:4 | 788.5236 | [M+H]+ | 11.46 | <0.001 | 0.002 ± 0.003 | <0.001 | 0.007 ± 0.005 | 0.002 ± 0.002 | 0.001 ± 0.001 |
| PG 16:0_16:1 | 719.4868 | [M-H]- | 11.36 | <0.001 | <0.001 | <0.001 | <0.001 | <0.001 | <0.001 |
| PG 16:0_18:0 | 749.5324 | [M-H]- | 12.90 | 0.003 ± 0.001 | 0.028 ± 0.033 | 0.011 ± 0.002 | 0.075 ± 0.027 | 0.016 ± 0.006 | 0.11 ± 0.02 |
| PG 16:0_18:1 | 747.5170 | [M-H]- | 12.82 | 0.025 ± 0.019 | 0.020 ± 0.027 | 0.042 ± 0.028 | 0.23 ± 0.03 | 0.011 ± 0.010 | 0.30 ± 0.07 |
| PG 16:0_18:2 | 745.5016 | [M-H]- | 11.58 | <0.001 | <0.001 | <0.001 | 0.004 ± 0.002 | <0.001 | 0.002 ± 0.001 |
| PG 18:0_18:1 | 775.5478 | [M-H]- | 14.10 | <0.001 | <0.001 | <0.001 | <0.001 | <0.001 | <0.001 |
| PG 18:1_18:1 | 773.5348 | [M-H]- | 12.86 | <0.001 | 0.026 ± 0.020 | 0.017 ± 0.017 | 0.087 ± 0.008 | 0.039 ± 0.016 | 0.099 ± 0.008 |
| PG 18:1_18:2 | 771.5170 | [M-H]- | 11.10 | 0.010 ± 0.001 | 0.008 ± 0.001 | 0.012 ± 0.001 | 0.011 ± 0.002 | 0.009 ± 0.003 | 0.007 ± 0.001 |
| PG 18:1_20:4 | 814.5581 | [M+NH4]+ | 11.30 | 0.001 ± 0.001 | <0.001 | <0.001 | <0.001 | <0.001 | <0.001 |
| PG 22:4_22:4 | 873.5655 | [M-H]- | 14.89 | <0.001 | <0.001 | <0.001 | <0.001 | <0.001 | <0.001 |
| PI 14:0_16:0 | 781.4864 | [M-H]- | 10.96 | 0.008 ± 0.001 | 0.013 ± 0.001 | 0.006 ± 0.001 | 0.020 ± 0.001 | 0.004 ± 0.001 | 0.12 ± 0.006 |
| PI 16:0_16:0 | 809.5188 | [M-H]- | 12.20 | 0.071 ± 0.002 | 0.045 ± 0.008 | 0.034 ± 0.003 | 0.016 ± 0.007 | 0.028 ± 0.016 | 0.59 ± 0.17 |
| PI 16:0_16:1 | 807.5025 | [M-H]- | 11.09 | 0.23 ± 0.01 | 0.11 ± 0.01 | 0.17 ± 0.01 | 0.20 ± 0.03 | 0.094 ± 0.009 | 1.32 ± 0.06 |
| PI 16:0_17:1 | 821.5198 | [M-H]- | 11.69 | 0.020 ± 0.002 | 0.052 ± 0.032 | 0.037 ± 0.008 | 0.040 ± 0.010 | 0.050 ± 0.009 | 0.12 ± 0.02 |
| PI 16:0_18:0 | 837.5505 | [M-H]- | 13.71 | <0.001 | <0.001 | <0.001 | <0.001 | <0.001 | 0.047 ± 0.082 |
| PI 16:0_18:1 | 835.5351 | [M-H]- | 12.31 | 1.09 ± 0.02 | 0.78 ± 0.14 | 0.83 ± 0.08 | 1.12 ± 0.34 | 0.73 ± 0.22 | 2.90 ± 0.59 |
| PI 16:0_18:2 | 833.5169 | [M-H]- | 11.30 | 1.16 ± 0.02 | 1.00 ± 0.03 | 1.14 ± 0.06 | 1.60 ± 0.04 | 0.81 ± 0.01 | 2.30 ± 0.14 |
| PI 16:0_18:3 | 831.5029 | [M-H]- | 10.54 | 0.006 ± 0.001 | 0.003 ± 0.001 | 0.007 ± <0.001 | 0.020 ± 0.002 | 0.002 ± 0.001 | 0.021 ± 0.005 |
| PI 16:0_20:3 | 859.5338 | [M-H]- | 11.58 | 0.55 ± 0.004 | 0.36 ± 0.05 | 0.45 ± 0.03 | 0.38 ± 0.03 | 0.37 ± 0.06 | 0.47 ± 0.03 |
| PI 16:0_20:4 | 857.5180 | [M-H]- | 11.12 | 1.20 ± 0.05 | 1.19 ± 0.12 | 1.26 ± 0.04 | 1.70 ± 0.12 | 0.93 ± 0.10 | 2.86 ± 0.26 |
| PI 16:0_20:5 | 855.5023 | [M-H]- | 10.37 | 0.008 ± 0.001 | 0.008 ± 0.001 | 0.010 ± 0.001 | 0.059 ± 0.003 | 0.003 ± <0.001 | 0.28 ± 0.02 |
| PI 16:0_22:6 | 881.5183 | [M-H]- | 10.80 | 0.11 ± 0.005 | 0.19 ± 0.01 | 0.13 ± 0.01 | 0.39 ± 0.01 | 0.15 ± 0.004 | 1.04 ± 0.06 |
| PI 17:0_18:2 | 847.5333 | [M-H]- | 11.91 | 0.043 ± <0.001 | 0.025 ± 0.007 | 0.037 ± 0.004 | 0.038 ± 0.004 | 0.034 ± 0.005 | 0.071 ± 0.012 |
| PI 17:0_20:3 | 873.5489 | [M-H]- | 12.23 | 0.007 ± 0.002 | 0.005 ± 0.003 | 0.008 ± 0.003 | 0.002 ± 0.001 | 0.007 ± 0.008 | 0.008 ± 0.007 |
| PI 17:0_20:4 | 871.5328 | [M-H]- | 11.69 | 0.086 ± 0.003 | 0.064 ± 0.006 | 0.091 ± 0.002 | 0.091 ± 0.016 | 0.076 ± 0.010 | 0.17 ± 0.02 |
| PI 18:0_18:1 | 863.5660 | [M-H]- | 13.83 | 0.87 ± 0.03 | 0.73 ± 0.20 | 0.67 ± 0.05 | 1.01 ± 0.31 | 0.79 ± 0.20 | 1.28 ± 0.26 |
| PI 18:0_18:2 | 861.5487 | [M-H]- | 12.61 | 2.66 ± 0.03 | 1.65 ± 0.21 | 2.19 ± 0.16 | 2.47 ± 0.62 | 1.55 ± 0.26 | 1.96 ± 0.29 |
| PI 18:0_18:3 | 859.5347 | [M-H]- | 11.83 | 0.084 ± 0.003 | 0.053 ± 0.004 | 0.062 ± 0.004 | 0.045 ± 0.007 | 0.033 ± 0.009 | 0.091 ± 0.012 |
| PI 18:0_20:1 | 891.5962 | [M-H]- | 15.03 | <0.001 | 0.001 ± <0.001 | 0.001 ± 0.001 | 0.003 ± 0.001 | 0.001 ± 0.001 | <0.001 |
| PI 18:0_20:2 | 889.5805 | [M-H]- | 13.98 | 0.044 ± 0.004 | 0.026 ± 0.008 | 0.037 ± 0.008 | 0.043 ± 0.011 | 0.080 ± 0.020 | 0.034 ± 0.006 |
| PI 18:0_20:3 | 887.5651 | [M-H]- | 12.98 | 0.90 ± 0.03 | 0.65 ± 0.16 | 0.99 ± 0.10 | 0.85 ± 0.19 | 1.22 ± 0.23 | 0.73 ± 0.11 |
| PI 18:0_20:4 | 885.5504 | [M-H]- | 12.36 | 8.93 ± 0.15 | 6.91 ± 0.96 | 9.37 ± 1.00 | 10.6 ± 1.4 | 7.62 ± 0.58 | 8.78 ± 0.59 |
| PI 18:0_22:4 | 913.5804 | [M-H]- | 13.42 | 0.089 ± 0.001 | 0.029 ± 0.009 | 0.040 ± 0.007 | 0.026 ± 0.006 | 0.033 ± 0.009 | 0.025 ± 0.010 |
| PI 18:0_22:5 | 911.5644 | [M-H]- | 12.37 | 0.20 ± 0.004 | 0.12 ± 0.01 | 0.17 ± 0.03 | 0.30 ± 0.01 | 0.13 ± 0.01 | 0.65 ± 0.04 |
| PI 18:0_22:6 | 909.5491 | [M-H]- | 11.95 | 0.24 ± 0.01 | 0.29 ± 0.01 | 0.23 ± 0.02 | 0.49 ± 0.04 | 0.30 ± 0.003 | 0.98 ± 0.03 |
| PI 18:1_18:2 | 859.5332 | [M-H]- | 11.40 | 0.35 ± 0.02 | 0.37 ± 0.07 | 0.32 ± 0.02 | 0.68 ± 0.04 | 0.47 ± 0.04 | 0.45 ± 0.08 |
| PI 18:1_20:4 | 883.5330 | [M-H]- | 11.19 | 0.98 ± 0.03 | 0.66 ± 0.03 | 0.78 ± 0.01 | 1.04 ± 0.06 | 0.67 ± 0.03 | 1.78 ± 0.08 |
| PI 18:1_20:5 | 881.5182 | [M-H]- | 10.48 | 0.003 ± 0.001 | 0.002 ± <0.001 | 0.003 ± 0.001 | 0.018 ± 0.002 | 0.003 ± 0.001 | 0.034 ± 0.002 |
| PI 18:1_22:5 | 909.5497 | [M-H]- | 11.29 | 0.022 ± 0.001 | 0.005 ± 0.002 | 0.008 ± 0.002 | 0.021 ± 0.001 | 0.007 ± 0.003 | 0.041 ± 0.002 |
| PI 18:1_22:6 | 907.5338 | [M-H]- | 11.00 | 0.020 ± 0.003 | 0.021 ± 0.002 | 0.009 ± 0.003 | 0.048 ± 0.006 | 0.022 ± 0.005 | 0.078 ± 0.007 |
| PS 16:0_20:5 | 780.4806 | [M-H]- | 12.16 | <0.001 | <0.001 | <0.001 | <0.001 | <0.001 | <0.001 |
| PS 18:0_18:0 | 790.5602 | [M-H]- | 14.18 | 0.051 ± 0.016 | 0.12 ± 0.04 | 0.17 ± 0.02 | 0.037 ± 0.021 | 0.24 ± 0.08 | 0.070 ± 0.032 |
| PS 18:0_18:1 | 788.5437 | [M-H]- | 14.18 | <0.001 | <0.001 | <0.001 | <0.001 | <0.001 | <0.001 |
| PS 18:0_20:4 | 810.5275 | [M-H]- | 14.70 | 0.16 ± 0.01 | 0.42 ± 0.06 | 0.38 ± 0.02 | 0.52 ± 0.04 | 0.59 ± 0.03 | 0.33 ± 0.03 |
| PS 18:1_18:1 | 786.5270 | [M-H]- | 13.49 | <0.001 | <0.001 | <0.001 | <0.001 | <0.001 | <0.001 |
| PS 18:1_20:3 | 810.5277 | [M-H]- | 14.54 | 0.005 ± 0.001 | 0.042 ± 0.019 | 0.028 ± 0.013 | 0.078 ± 0.021 | 0.095 ± 0.032 | 0.057 ± 0.014 |
| PS 18:2_18:2 | 782.4958 | [M-H]- | 13.23 | 0.022 ± 0.007 | 0.047 ± 0.011 | 0.043 ± 0.015 | 0.066 ± 0.015 | 0.079 ± 0.012 | 0.049 ± 0.007 |
| PS 18:2_20:3 | 808.5114 | [M-H]- | 13.30 | 0.011 ± 0.005 | 0.057 ± 0.006 | 0.019 ± 0.010 | 0.095 ± 0.005 | 0.10 ± 0.02 | 0.039 ± 0.005 |
| PS 18:2_22:6 | 830.4953 | [M-H]- | 11.80 | <0.001 | <0.001 | <0.001 | <0.001 | <0.001 | <0.001 |
|  |  |  |  |  |  |  |  |  |  |
| ***Sphingolipids*** |  |  |  |  |  |  |  |  |  |
| Cer-AP t18:0/23:0 | 714.6248 | [M+HCO2]- | 17.13 | 0.007 ± <0.001 | 0.007 ± <0.001 | 0.010 ± 0.001 | 0.008 ± 0.001 | 0.008 ± <0.001 | 0.007 ± <0.001 |
| Cer-AP t18:0/24:0 | 728.6406 | [M+HCO2]-\|[M-H]- | 17.52 | 0.024 ± <0.001 | 0.027 ± 0.001 | 0.030 ± 0.002 | 0.030 ± 0.001 | 0.025 ± 0.001 | 0.026 ± 0.001 |
| Cer-AP t18:0/24:1 | 726.6262 | [M+HCO2]- | 16.55 | 0.003 ± 0.001 | 0.003 ± <0.001 | 0.010 ± 0.002 | 0.008 ± 0.001 | 0.001 ± 0.001 | 0.003 ± 0.002 |
| Cer-AP t18:0/26:0 | 756.6715 | [M+HCO2]-\|[M-H]- | 18.36 | 0.002 ± <0.001 | 0.002 ± <0.001 | 0.002 ± 0.001 | 0.005 ± <0.001 | 0.003 ± <0.001 | 0.003 ± <0.001 |
| Cer-AP t20:0/22:0 | 682.6337 | [M-H]- | 17.21 | 0.037 ± 0.009 | 0.032 ± 0.012 | 0.028 ± 0.004 | 0.024 ± 0.012 | 0.017 ± 0.003 | 0.021 ± 0.004 |
| Cer-AS d18:1/22:0 | 682.5995 | [M+HCO2]- | 17.11 | 0.014 ± 0.001 | 0.025 ± 0.002 | 0.052 ± 0.002 | 0.022 ± 0.013 | 0.015 ± 0.001 | 0.012 ± 0.001 |
| Cer-AS d18:1/23:0 | 650.6097 | [M-H]-\|[M+HCO2]- | 17.41 | 0.034 ± 0.002 | 0.030 ± 0.003 | 0.074 ± 0.001 | 0.051 ± 0.003 | 0.024 ± 0.001 | 0.023 ± 0.001 |
| Cer-AS d18:1/24:0 | 710.6307 | [M+HCO2]- | 18.02 | 0.072 ± 0.005 | 0.19 ± 0.02 | 0.14 ± 0.01 | 0.68 ± 0.16 | 0.051 ± 0.002 | 0.11 ± 0.03 |
| Cer-AS d18:1/24:1 | 708.6140 | [M+HCO2]-\|[M-H]- | 17.04 | 0.016 ± 0.001 | 0.024 ± 0.005 | 0.043 ± 0.001 | 0.023 ± 0.002 | 0.027 ± 0.003 | 0.025 ± 0.001 |
| Cer-AS d26:2/16:1 | 706.5979 | [M+HCO2]- | 16.24 | 0.003 ± <0.001 | 0.004 ± 0.001 | 0.011 ± 0.001 | 0.004 ± 0.002 | 0.005 ± 0.001 | 0.003 ± <0.001 |
| Cer-AS d27:1/16:0 | 724.6466 | [M+HCO2]- | 18.26 | 0.016 ± 0.001 | 0.014 ± 0.001 | 0.019 ± 0.004 | 0.019 ± 0.003 | 0.006 ± 0.002 | 0.008 ± 0.002 |
| Cer-BDS d24:0/17:0 | 698.6300 | [M+HCO2]- | 16.86 | 0.006 ± <0.001 | 0.007 ± <0.001 | 0.007 ± 0.001 | 0.009 ± 0.001 | 0.006 ± <0.001 | 0.010 ± 0.005 |
| Cer-BS d18:1/24:1 | 708.6137 | [M+HCO2]- | 16.67 | <0.001 | <0.001 | <0.001 | 0.003 ± 0.002 | <0.001 | <0.001 |
| Cer-NDS d16:0/24:1 | 666.6044 | [M+HCO2]- | 16.95 | 0.015 ± 0.001 | 0.018 ± 0.002 | 0.029 ± 0.001 | 0.022 ± 0.005 | 0.021 ± 0.008 | 0.013 ± 0.005 |
| Cer-NDS d18:0/16:0 | 584.5255 | [M+HCO2]-\|[M+H]+ | 14.79 | 0.028 ± <0.001 | 0.030 ± 0.006 | 0.035 ± <0.001 | 0.028 ± 0.005 | 0.019 ± 0.002 | 0.015 ± 0.002 |
| Cer-NDS d18:0/18:0 | 612.5564 | [M+HCO2]- | 16.30 | 0.37 ± 0.07 | 0.68 ± 0.29 | 0.64 ± 0.05 | 0.91 ± 0.40 | 0.32 ± 0.16 | 0.47 ± 0.24 |
| Cer-NDS d18:0/20:0 | 640.5887 | [M+HCO2]- | 17.20 | 0.12 ± 0.02 | 0.20 ± 0.07 | 0.17 ± 0.01 | 0.27 ± 0.07 | 0.084 ± 0.029 | 0.14 ± 0.05 |
| Cer-NDS d18:0/22:0 | 668.6197 | [M+HCO2]-\|[M+H]+ | 17.78 | 0.092 ± 0.007 | 0.13 ± 0.002 | 0.18 ± 0.01 | 0.11 ± 0.01 | 0.067 ± 0.004 | 0.074 ± 0.002 |
| Cer-NDS d18:0/23:0 | 682.6355 | [M+HCO2]-\|[M+H]+ | 18.21 | 0.058 ± 0.002 | 0.084 ± 0.002 | 0.11 ± 0.002 | 0.064 ± 0.004 | 0.056 ± 0.002 | 0.045 ± 0.003 |
| Cer-NDS d18:0/24:0 | 696.6502 | [M+HCO2]-\|[M-H]-\|[M+H]+ | 18.65 | 0.14 ± 0.004 | 0.18 ± 0.01 | 0.25 ± 0.01 | 0.16 ± 0.005 | 0.10 ± 0.004 | 0.095 ± <0.001 |
| Cer-NDS d18:0/24:1 | 694.6350 | [M+HCO2]-\|[M+H]+ | 17.71 | 0.087 ± 0.004 | 0.067 ± 0.018 | 0.12 ± 0.01 | 0.082 ± 0.020 | 0.060 ± 0.018 | 0.049 ± 0.014 |
| Cer-NDS d18:0/24:2 | 692.6195 | [M+HCO2]-\|[M+H]+ | 16.99 | 0.017 ± 0.001 | 0.011 ± 0.002 | 0.025 ± 0.001 | 0.016 ± <0.001 | 0.014 ± <0.001 | 0.006 ± 0.001 |
| Cer-NDS d19:0/24:0 | 710.6664 | [M+HCO2]-\|[M+H]+ | 18.91 | 0.008 ± 0.001 | 0.012 ± 0.001 | 0.021 ± 0.002 | 0.011 ± 0.001 | 0.009 ± 0.002 | 0.004 ± 0.001 |
| Cer-NDS d19:0/24:1 | 708.6509 | [M+HCO2]- | 18.10 | 0.003 ± <0.001 | 0.003 ± 0.001 | 0.007 ± <0.001 | 0.004 ± 0.001 | 0.005 ± 0.001 | 0.003 ± 0.001 |
| Cer-NDS d20:0/23:0 | 710.6655 | [M+HCO2]- | 19.25 | <0.001 | <0.001 | 0.001 ± <0.001 | <0.001 | <0.001 | <0.001 |
| Cer-NDS d20:0/24:0 | 724.6821 | [M+HCO2]- | 19.56 | 0.004 ± <0.001 | 0.006 ± <0.001 | 0.008 ± <0.001 | 0.004 ± 0.001 | 0.003 ± <0.001 | 0.003 ± <0.001 |
| Cer-NP t18:0/24:2 | 708.6145 | [M+HCO2]- | 15.98 | 0.005 ± <0.001 | 0.005 ± <0.001 | 0.008 ± 0.001 | 0.008 ± <0.001 | 0.006 ± <0.001 | 0.006 ± <0.001 |
| Cer-NP t18:1/23:0 | 696.6151 | [M+HCO2]- | 16.89 | 0.007 ± 0.001 | 0.003 ± <0.001 | 0.005 ± <0.001 | 0.028 ± 0.006 | 0.002 ± <0.001 | 0.041 ± 0.024 |
| Cer-NP t18:1/23:1 | 694.5992 | [M+HCO2]- | 16.75 | 0.002 ± <0.001 | 0.005 ± 0.001 | 0.011 ± <0.001 | 0.002 ± <0.001 | 0.003 ± <0.001 | 0.002 ± <0.001 |
| Cer-NP t20:0/24:1 | 738.6623 | [M+HCO2]- | 17.80 | 0.001 ± <0.001 | <0.001 | <0.001 | 0.037 ± 0.003 | 0.001 ± <0.001 | 0.003 ± 0.002 |
| Cer-NS d15:1/15:0 | 526.4464 | [M+HCO2]-\|[M-H]-\|[M+H]+ | 11.18 | 0.12 ± 0.01 | 0.13 ± 0.01 | 0.15 ± 0.01 | 0.15 ± 0.06 | 0.13 ± 0.01 | 0.13 ± 0.001 |
| Cer-NS d16:1/16:0 | 554.4784 | [M+HCO2]- | 12.61 | 0.015 ± <0.001 | 0.020 ± 0.006 | 0.033 ± 0.001 | 0.024 ± 0.010 | 0.021 ± 0.002 | 0.013 ± 0.002 |
| Cer-NS d16:1/22:0 | 592.5661 | [M-H]- | 16.63 | 0.003 ± <0.001 | 0.003 ± <0.001 | 0.006 ± <0.001 | 0.003 ± <0.001 | 0.003 ± <0.001 | 0.002 ± <0.001 |
| Cer-NS d16:1/24:0 | 666.6039 | [M+HCO2]- | 17.76 | 0.001 ± <0.001 | 0.001 ± <0.001 | 0.001 ± 0.001 | 0.001 ± <0.001 | <0.001 | <0.001 |
| Cer-NS d17:1/18:0 | 596.5258 | [M+HCO2]- | 14.95 | 0.008 ± 0.001 | 0.010 ± 0.003 | 0.019 ± 0.002 | 0.011 ± 0.002 | 0.007 ± 0.001 | 0.006 ± 0.002 |
| Cer-NS d17:1/20:0 | 624.5561 | [M+HCO2]- | 16.13 | 0.010 ± 0.001 | 0.010 ± 0.004 | 0.022 ± 0.001 | 0.009 ± 0.003 | 0.008 ± 0.002 | 0.006 ± 0.002 |
| Cer-NS d17:1/22:0 | 652.5888 | [M+HCO2]-\|[M+H]+ | 17.08 | 0.063 ± 0.003 | 0.075 ± 0.004 | 0.15 ± 0.003 | 0.079 ± 0.005 | 0.067 ± 0.002 | 0.059 ± 0.003 |
| Cer-NS d17:1/24:2 | 676.5881 | [M+HCO2]- | 16.22 | 0.004 ± 0.001 | 0.002 ± <0.001 | 0.006 ± 0.001 | 0.003 ± 0.001 | 0.004 ± <0.001 | 0.002 ± <0.001 |
| Cer-NS d17:2/22:0 | 650.5726 | [M+HCO2]- | 16.26 | 0.003 ± <0.001 | 0.003 ± 0.001 | 0.008 ± <0.001 | 0.002 ± <0.001 | 0.002 ± 0.001 | 0.002 ± 0.001 |
| Cer-NS d18:1/16:0 | 582.5097 | [M+HCO2]-\|[M-H]-\|[M+H]+ | 14.29 | 0.16 ± 0.004 | 0.12 ± 0.04 | 0.21 ± 0.004 | 0.13 ± 0.04 | 0.12 ± 0.02 | 0.10 ± 0.03 |
| Cer-NS d18:1/18:0 | 610.5406 | [M+HCO2]-\|[M+H]+ | 15.68 | 1.20 ± 0.09 | 0.86 ± 0.61 | 1.42 ± 0.06 | 1.14 ± 0.09 | 0.37 ± 0.45 | 0.94 ± 0.05 |
| Cer-NS d18:1/20:0 | 594.5824 | [M+H]+ | 16.64 | 0.096 ± 0.002 | 0.099 ± 0.007 | 0.17 ± 0.01 | 0.097 ± 0.002 | 0.087 ± 0.004 | 0.071 ± 0.003 |
| Cer-NS d18:1/22:0 | 666.6043 | [M+HCO2]-\|[M-H]-\|[M+H]+ | 17.48 | 0.66 ± 0.03 | 0.77 ± 0.06 | 1.14 ± 0.02 | 0.72 ± 0.02 | 0.45 ± 0.01 | 0.52 ± 0.03 |
| Cer-NS d18:1/22:2 | 662.5714 | [M+HCO2]- | 15.74 | 0.031 ± 0.028 | 0.028 ± 0.012 | 0.085 ± 0.040 | 0.026 ± 0.010 | 0.038 ± 0.011 | 0.026 ± 0.014 |
| Cer-NS d18:1/23:0 | 680.6198 | [M+HCO2]-\|[M-H]-\|[M+H]+ | 17.89 | 0.55 ± 0.03 | 0.62 ± 0.03 | 0.98 ± 0.04 | 0.63 ± 0.003 | 0.45 ± 0.01 | 0.49 ± 0.02 |
| Cer-NS d18:1/24:0 | 694.6350 | [M+HCO2]-\|[M-H]-\|[M+H]+ | 18.33 | 1.74 ± 0.08 | 2.14 ± 0.01 | 2.82 ± 0.11 | 2.04 ± 0.07 | 1.27 ± 0.05 | 1.49 ± 0.03 |
| Cer-NS d18:1/24:1 | 692.6193 | [M+HCO2]-\|[M-H]- | 17.40 | 0.57 ± 0.03 | 0.58 ± 0.09 | 0.73 ± 0.02 | 0.82 ± 0.10 | 0.60 ± 0.07 | 0.64 ± 0.09 |
| Cer-NS d18:1/24:2 | 646.6123 | [M+H]+ | 17.78 | 0.021 ± 0.001 | 0.032 ± 0.001 | 0.041 ± 0.003 | 0.016 ± 0.001 | 0.016 ± <0.001 | 0.013 ± 0.001 |
| Cer-NS d18:1/25:0 | 662.6434 | [M-H]-\|[M+H]+ | 18.79 | 0.001 ± <0.001 | 0.001 ± 0.001 | 0.002 ± <0.001 | 0.001 ± <0.001 | <0.001 | <0.001 |
| Cer-NS d18:1/26:0 | 722.6666 | [M+HCO2]- | 19.23 | 0.046 ± 0.002 | 0.056 ± 0.004 | 0.084 ± 0.005 | 0.046 ± 0.001 | 0.035 ± 0.001 | 0.033 ± 0.001 |
| Cer-NS d18:1/26:1 | 720.6508 | [M+HCO2]- | 18.22 | 0.027 ± 0.002 | 0.027 ± 0.002 | 0.035 ± 0.001 | 0.032 ± 0.001 | 0.020 ± 0.001 | 0.023 ± 0.001 |
| Cer-NS d18:1/26:2 | 718.6331 | [M+HCO2]- | 17.47 | 0.004 ± <0.001 | 0.003 ± 0.001 | 0.003 ± 0.001 | 0.004 ± 0.001 | 0.001 ± 0.001 | 0.003 ± <0.001 |
| Cer-NS d18:2/16:0 | 580.4940 | [M+HCO2]- | 12.81 | 0.069 ± 0.008 | 0.065 ± 0.006 | 0.12 ± 0.01 | 0.076 ± 0.011 | 0.070 ± 0.003 | 0.064 ± 0.009 |
| Cer-NS d18:2/18:0 | 608.5254 | [M+HCO2]- | 14.49 | 0.15 ± 0.01 | 0.12 ± 0.00 | 0.25 ± 0.02 | 0.22 ± 0.01 | 0.060 ± 0.004 | 0.076 ± 0.008 |
| Cer-NS d18:2/20:0 | 636.5565 | [M+HCO2]- | 15.64 | 0.53 ± 0.08 | 0.36 ± 0.15 | 0.75 ± 0.09 | 0.27 ± 0.06 | 0.21 ± 0.13 | 0.15 ± 0.06 |
| Cer-NS d18:2/22:0 | 664.5880 | [M+HCO2]-\|[M-H]-\|[M+H]+ | 16.71 | 0.19 ± 0.002 | 0.21 ± 0.02 | 0.34 ± 0.01 | 0.18 ± 0.01 | 0.14 ± 0.02 | 0.15 ± 0.02 |
| Cer-NS d18:2/23:0 | 678.6042 | [M+HCO2]-\|[M+H]+ | 17.15 | 0.14 ± 0.004 | 0.15 ± 0.005 | 0.27 ± 0.01 | 0.17 ± 0.01 | 0.13 ± 0.006 | 0.14 ± 0.01 |
| Cer-NS d18:2/24:0 | 648.6294 | [M+H]+ | 17.51 | 0.76 ± 0.05 | 0.68 ± 0.13 | 1.01 ± 0.06 | 0.70 ± 0.09 | 0.52 ± 0.14 | 0.49 ± 0.10 |
| Cer-NS d18:2/24:1 | 690.6041 | [M-H]-\|[M+HCO2]-\|[M+H]+ | 16.64 | 0.21 ± 0.004 | 0.16 ± 0.01 | 0.27 ± 0.01 | 0.21 ± 0.01 | 0.17 ± 0.01 | 0.16 ± 0.01 |
| Cer-NS d18:2/24:2 | 644.5964 | [M+H]+ | 17.49 | 0.12 ± 0.002 | 0.15 ± 0.004 | 0.18 ± 0.004 | 0.10 ± 0.01 | 0.10 ± 0.003 | 0.083 ± 0.001 |
| Cer-NS d18:2/25:0 | 706.6347 | [M+HCO2]-\|[M+H]+ | 17.94 | 0.029 ± 0.001 | 0.027 ± 0.005 | 0.043 ± 0.002 | 0.030 ± 0.003 | 0.020 ± 0.004 | 0.022 ± 0.004 |
| Cer-NS d19:1/20:0 | 652.5889 | [M+HCO2]- | 16.91 | 0.004 ± 0.001 | 0.006 ± 0.002 | 0.008 ± <0.001 | 0.007 ± 0.005 | 0.008 ± 0.002 | 0.006 ± 0.004 |
| Cer-NS d19:1/22:2 | 632.5976 | [M+H]+ | 17.36 | <0.001 | <0.001 | <0.001 | <0.001 | <0.001 | <0.001 |
| Cer-NS d19:1/24:0 | 708.6505 | [M+HCO2]-\|[M-H]-\|[M+H]+ | 18.59 | 0.13 ± 0.004 | 0.20 ± 0.03 | 0.30 ± 0.003 | 0.18 ± 0.03 | 0.18 ± 0.03 | 0.12 ± 0.02 |
| Cer-NS d19:1/24:1 | 706.6347 | [M+HCO2]-\|[M+H]+ | 17.64 | 0.042 ± 0.002 | 0.053 ± 0.006 | 0.073 ± 0.004 | 0.061 ± 0.006 | 0.074 ± 0.009 | 0.042 ± 0.007 |
| Cer-NS d19:1/24:2 | 704.6198 | [M+HCO2]- | 17.06 | 0.009 ± <0.001 | 0.007 ± 0.001 | 0.014 ± 0.001 | 0.009 ± 0.001 | 0.008 ± 0.001 | 0.006 ± <0.001 |
| Cer-NS d20:1/24:0 | 722.6667 | [M+HCO2]-\|[M+H]+ | 19.09 | 0.007 ± 0.001 | 0.010 ± 0.003 | 0.014 ± <0.001 | 0.009 ± 0.004 | 0.010 ± 0.002 | 0.007 ± 0.002 |
| Cer-NS d20:1/24:1 | 676.6611 | [M+H]+ | 18.23 | 0.039 ± 0.001 | 0.033 ± 0.003 | 0.046 ± 0.001 | 0.046 ± 0.002 | 0.035 ± 0.002 | 0.030 ± 0.002 |
| Cer-NS d20:1/25:0 | 736.6823 | [M+HCO2]- | 19.60 | 0.001 ± <0.001 | 0.001 ± <0.001 | <0.001 | 0.001 ± <0.001 | 0.001 ± <0.001 | <0.001 |
| NeuAcα2-3Galβ1-4Glcβ-Cer d34:1 | 1151.7054 | [M-H]- | 10.77 | 0.047 ± 0.004 | 0.042 ± 0.007 | 0.045 ± 0.002 | 0.052 ± 0.004 | 0.036 ± 0.002 | 0.046 ± 0.002 |
| NeuAcα2-3Galβ1-4Glcβ-Cer d36:1 | 1179.7365 | [M-H]- | 11.92 | 0.016 ± 0.001 | 0.013 ± 0.001 | 0.016 ± 0.002 | 0.019 ± 0.003 | 0.010 ± 0.001 | 0.011 ± <0.001 |
| NeuAcα2-3Galβ1-4Glcβ-Cer d38:1 | 1207.7673 | [M-H]- | 13.38 | 0.002 ± 0.001 | 0.004 ± 0.003 | 0.004 ± 0.003 | 0.008 ± 0.002 | 0.004 ± 0.002 | 0.005 ± 0.001 |
| NeuAcα2-3Galβ1-4Glcβ-Cer d40:1 | 1235.7979 | [M-H]- | 14.71 | 0.034 ± 0.001 | 0.033 ± 0.011 | 0.040 ± 0.003 | 0.034 ± 0.009 | 0.019 ± 0.003 | 0.023 ± 0.006 |
| NeuAcα2-3Galβ1-4Glcβ-Cer d42:1 | 1263.8294 | [M-H]- | 15.72 | 0.037 ± 0.002 | 0.041 ± 0.005 | 0.036 ± 0.002 | 0.043 ± 0.006 | 0.022 ± 0.003 | 0.029 ± 0.002 |
| NeuAcα2-3Galβ1-4Glcβ-Cer d42:2 | 1261.8142 | [M-H]- | 14.62 | 0.031 ± 0.001 | 0.025 ± 0.002 | 0.022 ± 0.001 | 0.040 ± 0.002 | 0.023 ± <0.001 | 0.035 ± 0.002 |
| HexCer-AP t18:0/16:0 | 778.5680 | [M+HCO2]- | 11.85 | 0.005 ± <0.001 | 0.007 ± 0.001 | 0.008 ± <0.001 | 0.008 ± 0.001 | 0.005 ± <0.001 | 0.006 ± 0.001 |
| HexCer-AP t18:0/22:0 | 862.6603 | [M+HCO2]- | 16.07 | 0.006 ± 0.001 | 0.011 ± 0.001 | 0.012 ± 0.001 | 0.012 ± <0.001 | 0.012 ± 0.001 | 0.014 ± 0.003 |
| HexCer-AP t18:0/24:0 | 890.6924 | [M+HCO2]- | 16.91 | 0.009 ± <0.001 | 0.014 ± 0.001 | 0.017 ± 0.001 | 0.017 ± 0.001 | 0.014 ± 0.001 | 0.015 ± 0.002 |
| HexCer-AP t18:0/24:1 | 888.6765 | [M+HCO2]-\|[M-H]- | 15.95 | 0.009 ± 0.001 | 0.013 ± 0.001 | 0.015 ± 0.002 | 0.017 ± 0.001 | 0.015 ± 0.002 | 0.021 ± 0.001 |
| HexCer-AP t18:0/24:2 | 886.6622 | [M+HCO2]- | 15.01 | <0.001 | <0.001 | <0.001 | <0.001 | <0.001 | <0.001 |
| HexCer-NDS d18:0/22:0 | 786.6828 | [M+H]+ | 16.95 | <0.001 | <0.001 | <0.001 | <0.001 | <0.001 | <0.001 |
| HexCer-NDS d18:0/24:1 | 812.6961 | [M+H]+ | 16.87 | 0.001 ± 0.001 | <0.001 | <0.001 | 0.001 ± 0.001 | 0.001 ± 0.001 | 0.002 ± 0.002 |
| HexCer-NDS d24:0/16:1 | 784.6659 | [M+H]+ | 17.08 | <0.001 | <0.001 | <0.001 | 0.004 ± 0.002 | <0.001 | <0.001 |
| HexCer-NS d16:1/16:0 | 716.5321 | [M+HCO2]- | 11.43 | 0.005 ± <0.001 | 0.006 ± 0.001 | 0.009 ± 0.001 | 0.009 ± 0.001 | 0.009 ± <0.001 | 0.007 ± 0.001 |
| HexCer-NS d17:1/22:0 | 814.6432 | [M+HCO2]-\|[M+H]+ | 16.22 | 0.016 ± <0.001 | 0.017 ± <0.001 | 0.030 ± 0.002 | 0.023 ± 0.002 | 0.027 ± 0.001 | 0.026 ± 0.002 |
| HexCer-NS d17:1/31:0 | 896.7915 | [M+H]+ | 22.22 | <0.001 | <0.001 | <0.001 | <0.001 | <0.001 | <0.001 |
| HexCer-NS d18:1/16:0 | 744.5635 | [M-H]-\|[M+HCO2]-\|[M+H]+ | 12.88 | 0.33 ± 0.02 | 0.25 ± 0.02 | 0.46 ± 0.04 | 0.26 ± 0.05 | 0.27 ± 0.02 | 0.33 ± 0.05 |
| HexCer-NS d18:1/17:0 | 714.5879 | [M+H]+ | 13.76 | 0.002 ± 0.002 | 0.001 ± <0.001 | 0.002 ± 0.002 | 0.001 ± 0.001 | 0.001 ± <0.001 | 0.003 ± 0.001 |
| HexCer-NS d18:1/18:0 | 772.5938 | [M+HCO2]-\|[M+H]+ | 14.52 | 0.041 ± 0.005 | 0.027 ± 0.007 | 0.045 ± 0.003 | 0.036 ± 0.004 | 0.043 ± 0.004 | 0.037 ± 0.006 |
| HexCer-NS d18:1/20:0 | 800.6260 | [M+HCO2]-\|[M+H]+ | 15.71 | 0.048 ± 0.002 | 0.046 ± 0.006 | 0.078 ± 0.003 | 0.068 ± 0.005 | 0.059 ± 0.003 | 0.065 ± 0.001 |
| HexCer-NS d18:1/22:0 | 828.6572 | [M-H]-\|[M+HCO2]-\|[M+H]+ | 16.66 | 0.30 ± 0.01 | 0.29 ± 0.02 | 0.42 ± 0.02 | 0.36 ± 0.002 | 0.37 ± 0.01 | 0.39 ± 0.007 |
| HexCer-NS d18:1/23:0 | 842.6722 | [M+HCO2]-\|[M+H]+ | 17.08 | 0.14 ± 0.01 | 0.12 ± 0.01 | 0.19 ± 0.01 | 0.17 ± 0.005 | 0.17 ± 0.004 | 0.21 ± 0.002 |
| HexCer-NS d18:1/24:0 | 856.6874 | [M+HCO2]-\|[M-H]-\|[M+H]+ | 17.46 | 0.36 ± 0.02 | 0.33 ± 0.02 | 0.44 ± 0.01 | 0.45 ± 0.01 | 0.40 ± 0.02 | 0.48 ± 0.01 |
| HexCer-NS d18:1/24:1 | 854.6713 | [M+HCO2]-\|[M+H]+ | 16.59 | 0.32 ± 0.01 | 0.23 ± 0.01 | 0.32 ± 0.01 | 0.37 ± 0.01 | 0.38 ± 0.01 | 0.53 ± 0.02 |
| HexCer-NS d18:1/25:0 | 870.7034 | [M+HCO2]-\|[M+H]+ | 17.86 | 0.011 ± <0.001 | 0.009 ± 0.002 | 0.013 ± 0.002 | 0.013 ± 0.001 | 0.011 ± 0.001 | 0.013 ± 0.001 |
| HexCer-NS d18:1/25:1 | 868.6883 | [M+HCO2]- | 16.97 | 0.005 ± 0.001 | 0.006 ± 0.003 | 0.006 ± 0.001 | 0.008 ± 0.001 | 0.006 ± 0.001 | 0.009 ± 0.002 |
| HexCer-NS d18:1/26:0 | 884.7189 | [M+HCO2]-\|[M+H]+ | 18.27 | 0.004 ± 0.001 | 0.003 ± 0.001 | 0.003 ± <0.001 | 0.003 ± <0.001 | 0.003 ± 0.001 | 0.005 ± 0.001 |
| HexCer-NS d18:2/22:0 | 826.6413 | [M+HCO2]-\|[M+H]+ | 15.80 | 0.042 ± 0.004 | 0.038 ± 0.001 | 0.063 ± 0.002 | 0.055 ± 0.003 | 0.043 ± 0.002 | 0.056 ± 0.003 |
| HexCer-NS d18:2/23:0 | 840.6585 | [M+HCO2]-\|[M+H]+ | 16.29 | 0.020 ± 0.003 | 0.014 ± 0.001 | 0.028 ± 0.002 | 0.024 ± 0.004 | 0.026 ± 0.004 | 0.029 ± 0.007 |
| HexCer-NS d18:2/24:0 | 808.6666 | [M-H]- | 16.66 | 0.012 ± 0.001 | 0.012 ± 0.001 | 0.015 ± 0.002 | 0.016 ± 0.005 | 0.012 ± 0.002 | 0.023 ± 0.002 |
| HexCer-NS d18:2/24:1 | 852.6564 | [M+HCO2]-\|[M+H]+ | 15.74 | 0.058 ± 0.001 | 0.040 ± 0.004 | 0.059 ± 0.002 | 0.059 ± 0.006 | 0.065 ± 0.003 | 0.081 ± 0.003 |
| HexCer-NS d18:2/32:1 | 920.7919 | [M+H]+ | 21.32 | <0.001 | <0.001 | <0.001 | <0.001 | <0.001 | <0.001 |
| HexCer-NS d18:2/34:1 | 948.8239 | [M+H]+ | 22.31 | <0.001 | <0.001 | <0.001 | <0.001 | <0.001 | <0.001 |
| SM d16:1/14:0 | 647.5119 | [M+H]+ | 9.69 | 0.18 ± 0.01 | 0.32 ± 0.01 | 0.38 ± 0.01 | 0.28 ± 0.01 | 0.26 ± 0.01 | 0.23 ± 0.001 |
| SM d16:1/16:0 | 675.5442 | [M+H]+ | 10.99 | 6.95 ± 0.68 | 7.47 ± 0.23 | 11.9 ± 0.3 | 8.52 ± 0.27 | 8.59 ± 0.43 | 9.34 ± 0.14 |
| SM d16:1/22:0 | 759.6378 | [M+H]+ | 15.19 | 0.015 ± 0.006 | 0.39 ± 0.65 | 0.051 ± 0.027 | 0.48 ± 0.79 | 0.35 ± 0.33 | 0.58 ± 0.46 |
| SM d16:1/23:0 | 773.6547 | [M+H]+ | 15.90 | 4.31 ± 0.29 | 4.60 ± 0.15 | 9.40 ± 0.17 | 5.55 ± 0.33 | 6.28 ± 0.21 | 6.62 ± 0.21 |
| SM d17:1/14:0 | 661.5280 | [M+H]+ | 10.35 | 0.093 ± 0.009 | 0.14 ± 0.004 | 0.211 ± 0.003 | 0.15 ± 0.001 | 0.15 ± 0.01 | 0.16 ± 0.001 |
| SM d17:1/16:0 | 689.5591 | [M+H]+ | 11.62 | 4.71 ± 0.41 | 4.29 ± 0.11 | 6.69 ± 0.04 | 4.66 ± 0.15 | 5.51 ± 0.04 | 5.38 ± 0.04 |
| SM d17:1/18:0 | 717.5910 | [M+H]+ | 13.20 | 1.73 ± 0.02 | 1.58 ± 0.10 | 2.76 ± 0.01 | 0.61 ± 0.09 | 0.29 ± 0.03 | 2.27 ± 0.05 |
| SM d17:1/24:1 | 799.6685 | [M+H]+ | 15.85 | 3.03 ± 0.29 | 3.39 ± 0.89 | 4.52 ± 0.13 | 5.53 ± 1.74 | 6.61 ± 0.83 | 7.86 ± 1.65 |
| SM d18:0/18:1 | 731.6067 | [M+H]+ | 13.23 | 0.29 ± 0.03 | 0.12 ± 0.01 | 0.28 ± 0.01 | 0.14 ± 0.02 | 0.11 ± 0.02 | 0.076 ± 0.007 |
| SM d18:1/16:0 | 703.5765 | [M+H]+ | 12.33 | 55.5 ± 1.2 | 50.4 ± 0.4 | 63.5 ± 0.8 | 54.3 ± 0.6 | 54.4 ± 1.6 | 55.7 ± 1.4 |
| SM d18:1/18:0 | 731.6065 | [M+H]+ | 14.03 | 8.64 ± 0.23 | 7.59 ± 0.14 | 11.7 ± 0.1 | 8.07 ± 0.09 | 8.00 ± 0.23 | 8.01 ± 0.08 |
| SM d18:1/18:1 | 729.5915 | [M+H]+ | 12.55 | 7.11 ± 0.25 | 5.04 ± 0.17 | 9.55 ± 0.07 | 5.71 ± 0.27 | 5.31 ± 0.20 | 6.29 ± 0.15 |
| SM d18:1/18:2 | 727.5758 | [M+H]+ | 11.78 | 0.12 ± 0.01 | 0.034 ± 0.002 | 0.09 ± 0.005 | 0.042 ± 0.002 | 0.030 ± 0.005 | 0.029 ± 0.007 |
| SM d18:1/20:0 | 759.6373 | [M+H]+ | 15.35 | 11.0 ± 0.5 | 11.9 ± 0.3 | 19.2 ± 0.04 | 13.3 ± 0.4 | 12.7 ± 0.2 | 13.2 ± 0.2 |
| SM d18:1/22:0 | 787.6699 | [M+H]+ | 16.38 | 28.8 ± 1.3 | 30.0 ± 1.2 | 44.9 ± 0.5 | 29.9 ± 0.5 | 26.8 ± 0.3 | 30.5 ± 0.4 |
| SM d18:1/23:0 | 801.6853 | [M+H]+ | 16.83 | 12.5 ± 0.8 | 12.0 ± 0.8 | 21.0 ± 0.5 | 13.5 ± 0.6 | 12.6 ± 0.5 | 15.2 ± 0.4 |
| SM d18:1/24:0 | 815.7001 | [M+H]+ | 17.23 | 21.4 ± 1.3 | 21.4 ± 0.9 | 32.4 ± 0.3 | 23.3 ± 0.7 | 18.0 ± 0.5 | 22.0 ± 0.3 |
| SM d18:1/24:1 | 813.6850 | [M+H]+ | 16.29 | 27.6 ± 1.8 | 25.6 ± 3.8 | 30.3 ± 1.0 | 37.3 ± 3.7 | 37.1 ± 4.7 | 44.0 ± 6.0 |
| SM d18:1/25:0 | 829.7158 | [M+H]+ | 17.47 | 0.49 ± 0.04 | 0.56 ± 0.03 | 1.13 ± 0.04 | 0.71 ± 0.11 | 0.82 ± 0.06 | 0.60 ± 0.10 |
| SM d18:2/12:0 | 645.4982 | [M+H]+ | 8.53 | 0.021 ± 0.002 | 0.066 ± 0.003 | 0.059 ± 0.003 | 0.035 ± 0.002 | 0.033 ± 0.001 | 0.027 ± 0.001 |
| SM d18:2/14:0 | 673.5278 | [M+H]+ | 9.87 | 0.54 ± 0.02 | 0.78 ± 0.02 | 1.18 ± 0.03 | 0.61 ± 0.04 | 0.62 ± 0.04 | 0.65 ± 0.02 |
| SM d18:2/16:0 | 701.5600 | [M+H]+ | 11.14 | 14.0 ± 0.9 | 12.5 ± 0.3 | 20.0 ± 0.3 | 14.7 ± 0.4 | 12.9 ± 0.8 | 16.5 ± 0.2 |
| SM d18:2/16:1 | 699.5442 | [M+H]+ | 10.18 | 0.076 ± 0.001 | 0.055 ± 0.004 | 0.12 ± 0.004 | 0.058 ± 0.005 | 0.054 ± 0.006 | 0.053 ± 0.002 |
| SM d18:2/17:0 | 715.5754 | [M+H]+ | 11.82 | 0.47 ± 0.03 | 0.33 ± 0.01 | 0.69 ± 0.01 | 0.38 ± 0.03 | 0.46 ± 0.006 | 0.48 ± 0.03 |
| SM d18:2/18:1 | 727.5752 | [M+H]+ | 11.42 | 0.99 ± 0.01 | 0.46 ± 0.04 | 1.07 ± 0.04 | 0.42 ± 0.01 | 0.47 ± 0.08 | 0.34 ± 0.05 |
| SM d18:2/20:0 | 757.6225 | [M+H]+ | 14.17 | 3.70 ± 0.09 | 3.40 ± 0.10 | 5.48 ± 0.09 | 3.97 ± 0.02 | 3.60 ± 0.03 | 4.73 ± 0.08 |
| SM d18:2/20:1 | 755.6038 | [M+H]+ | 14.53 | 0.026 ± 0.002 | 0.025 ± 0.006 | 0.031 ± 0.003 | 0.029 ± 0.001 | 0.018 ± 0.007 | 0.024 ± 0.005 |
| SM d18:2/20:3 | 751.5734 | [M+H]+ | 12.58 | 0.061 ± 0.005 | 0.042 ± 0.012 | 0.10 ± 0.01 | 0.047 ± 0.009 | 0.051 ± 0.007 | 0.048 ± 0.006 |
| SM d18:2/20:4 | 749.5568 | [M+H]+ | 11.33 | 0.016 ± 0.001 | 0.019 ± 0.005 | 0.026 ± 0.001 | 0.022 ± 0.002 | 0.030 ± 0.005 | 0.022 ± 0.004 |
| SM d18:2/22:0 | 785.6546 | [M+H]+ | 15.46 | 17.8 ± 0.9 | 17.5 ± 1.2 | 27.4 ± 0.3 | 17.6 ± 0.3 | 15.9 ± 1.5 | 19.4 ± 0.8 |
| SM d18:2/22:1 | 783.6359 | [M+H]+ | 15.81 | 0.007 ± 0.001 | 0.008 ± 0.001 | 0.018 ± 0.006 | 0.013 ± 0.003 | 0.021 ± 0.006 | 0.018 ± 0.005 |
| SM d18:2/22:3 | 779.6069 | [M+H]+ | 14.18 | 0.059 ± 0.004 | 0.076 ± 0.010 | 0.081 ± 0.010 | 0.071 ± 0.007 | 0.069 ± 0.012 | 0.071 ± 0.009 |
| SM d18:2/23:0 | 799.6694 | [M+H]+ | 15.99 | 7.00 ± 0.40 | 6.30 ± 0.72 | 11.5 ± 0.4 | 7.25 ± 0.34 | 7.49 ± 1.13 | 9.65 ± 0.87 |
| SM d18:2/24:1 | 811.6693 | [M+H]+ | 15.38 | 23.2 ± 1.0 | 18.4 ± 0.1 | 23.8 ± 0.2 | 23.7 ± 0.7 | 24.5 ± 0.5 | 28.5 ± 0.3 |
| SM d18:2/25:0 | 827.6994 | [M+H]+ | 16.57 | 1.96 ± 0.14 | 1.77 ± 0.05 | 2.87 ± 0.05 | 2.33 ± 0.10 | 3.32 ± 0.10 | 2.48 ± 0.07 |
| SM d20:0/18:4 | 753.5892 | [M+H]+ | 14.12 | 0.021 ± 0.009 | 0.023 ± 0.003 | 0.023 ± 0.001 | 0.003 ± 0.003 | 0.017 ± 0.007 | 0.003 ± 0.002 |
| SM d20:1/18:3 | 753.5890 | [M+H]+ | 13.78 | 0.002 ± 0.002 | 0.010 ± 0.004 | 0.014 ± 0.006 | 0.004 ± 0.002 | 0.008 ± 0.005 | 0.002 ± 0.002 |
| Sulfatide d18:1_16:0(2OH) | 796.5255 | [M+H]+ | 10.63 | 0.20 ± 0.005 | 0.25 ± 0.02 | 0.26 ± 0.01 | 0.21 ± 0.02 | 0.17 ± 0.01 | 0.26 ± 0.05 |
| Sulfatide d18:1_22:0 | 864.6247 | [M+H]+ | 14.73 | <0.001 | <0.001 | <0.001 | <0.001 | <0.001 | <0.001 |
|  |  |  |  |  |  |  |  |  |  |
| ***Sterols*** |  |  |  |  |  |  |  |  |  |
| CE 16:0 | 642.6190 | [M+NH4]+ | 23.80 | 12.2 ± 0.9 | 26.8 ± 0.9 | 33.2 ± 1.1 | 29.4 ± 1.0 | 24.0 ± 0.04 | 30.0 ± 1.5 |
| CE 16:1 | 640.6041 | [M+NH4]+ | 22.44 | 26.8 ± 1.5 | 18.6 ± 1.5 | 24.8 ± 1.5 | 22.5 ± 1.7 | 12.3 ± 0.5 | 48.2 ± 3.9 |
| CE 17:0 | 656.6341 | [M+NH4]+ | 24.15 | 0.20 ± 0.04 | 0.33 ± 0.04 | 0.59 ± 0.02 | 0.35 ± 0.05 | 0.36 ± 0.02 | 0.54 ± 0.06 |
| CE 17:1 | 654.6185 | [M+NH4]+ | 23.09 | 1.81 ± 0.26 | 1.51 ± 0.45 | 2.75 ± 0.09 | 1.58 ± 0.32 | 1.56 ± 0.18 | 4.09 ± 0.29 |
| CE 17:2 | 652.6036 | [M+NH4]+ | 22.05 | 0.16 ± 0.005 | 0.27 ± 0.11 | 0.22 ± 0.03 | 0.29 ± 0.08 | 0.10 ± 0.03 | 0.29 ± 0.08 |
| CE 18:0 | 670.6505 | [M+NH4]+ | 25.00 | 2.88 ± 0.08 | 3.23 ± 0.22 | 6.34 ± 0.21 | 2.66 ± 0.32 | 1.84 ± 0.20 | 3.56 ± 0.12 |
| CE 18:1 | 668.6346 | [M+NH4]+ | 23.69 | 282 ± 3 | 260 ± 27 | 385 ± 4 | 295 ± 42 | 230 ± 32 | 326 ± 31 |
| CE 18:2 | 666.6190 | [M+NH4]+ | 22.58 | 1323 ± 40 | 1429 ± 68 | 1856 ± 177 | 1460 ± 152 | 1256 ± 121 | 1305 ± 23 |
| CE 18:3 | 664.6034 | [M+NH4]+ | 21.62 | 151 ± 2 | 179 ± 7 | 286 ± 19 | 195 ± 17 | 127 ± 11 | 137 ± 2 |
| CE 18:4 | 662.5874 | [M+NH4]+ | 20.73 | 3.35 ± 0.06 | 4.26 ± 0.14 | 6.25 ± 0.43 | 8.61 ± 0.30 | 1.96 ± 0.21 | 6.44 ± 0.19 |
| CE 20:1 | 696.6669 | [M+NH4]+ | 23.66 | 2.41 ± 0.54 | 1.44 ± 0.34 | 2.29 ± 0.45 | 1.49 ± 0.46 | 1.61 ± 0.37 | 1.30 ± 0.45 |
| CE 20:2 | 694.6503 | [M+NH4]+ | 23.38 | 4.69 ± 0.08 | 9.05 ± 0.56 | 6.93 ± 0.27 | 10.3 ± 0.5 | 5.96 ± 0.49 | 6.48 ± 0.18 |
| CE 20:3 | 692.6356 | [M+NH4]+ | 22.71 | 52.2 ± 2.3 | 48.4 ± 5.2 | 71.8 ± 8.5 | 43.3 ± 4.2 | 55.0 ± 3.4 | 52.9 ± 1.5 |
| CE 20:4 | 690.6188 | [M+NH4]+ | 21.96 | 267 ± 14 | 343 ± 22 | 350 ± 30 | 271 ± 25 | 249 ± 12 | 366 ± 19 |
| CE 20:5 | 688.6037 | [M+NH4]+ | 21.02 | 42.0 ± 1.8 | 78.5 ± 6.3 | 79.4 ± 6.7 | 296 ± 15 | 42.7 ± 2.9 | 571 ± 27 |
| CE 22:4 | 718.6503 | [M+NH4]+ | 22.74 | 0.85 ± 0.11 | 1.03 ± 0.50 | 0.79 ± 0.22 | 0.88 ± 0.42 | 0.56 ± 0.20 | 0.59 ± 0.25 |
| CE 22:5 | 716.6348 | [M+NH4]+ | 22.25 | 7.41 ± 0.16 | 6.61 ± 0.81 | 8.69 ± 0.91 | 4.12 ± 0.16 | 6.79 ± 0.48 | 4.25 ± 0.25 |
| CE 22:6 | 714.6191 | [M+NH4]+ | 21.35 | 69.3 ± 1.3 | 135 ± 17 | 157 ± 17 | 263 ± 7 | 102 ± 6 | 270 ± 20 |
| CE 24:1 | 752.7282 | [M+NH4]+ | 27.14 | 0.079 ± 0.008 | 0.078 ± 0.015 | 0.081 ± 0.011 | 0.11 ± 0.01 | 0.081 ± 0.010 | 0.15 ± 0.03 |
|  |  |  |  |  |  |  |  |  |  |
| ***Prenols*** |  |  |  |  |  |  |  |  |  |
| Co Q9 | 812.6561 | [M+NH4]+ | 18.44 | 0.016 ± 0.001 | <0.001 | 0.033 ± 0.003 | <0.001 | 0.024 ± <0.001 | <0.001 |
| Co Q10 | 880.7196 | [M+NH4]+ | 19.77 | 0.49 ± 0.02 | 0.44 ± 0.01 | 0.70 ± 0.02 | 0.60 ± 0.01 | 0.46 ± 0.03 | 0.57 ± 0.02 |

Concentration values are shown as means ± standard deviation from technical replicates (n = 4 per sample type). RT, retention time; SRM, National Institutes of Standards and Technology Standard Reference Material; ILQC, Intra-Laboratory Quality Control; LO3, Low Omega-3; HO3, High Omega-3; DG, diacylglycerol; GlcADG, glucuronosyl diacylglycerol; MGDG, monogalactosyldiacylglycerol; SQDG, sulfoquinovosyl diacylglycerol; TG O, ether-linked triacylglycerol; OxTG, oxidized triacylglycerol; TG, triacylglycerol; BMP, bismonoacylglycerophosphate; DMPE, dimethyl-phosphatidylethanolamine; LPC O, plasmanyl lysophosphatidylcholine; LPC P, plasmenyl lysophosphatidylcholine; PC O, plasmanyl phosphatidylcholine; PC P, plasmenyl phosphatidylcholine; PE O, plasmanyl phosphatidylethanolamine; PE P, plasmenyl phosphatidylethanolamine; PS P, plasmenyl phosphatidylserine; LPA, lysophosphatidic acid; LPC, lysophosphatidylcholine; LPE, lysophosphatidylethanolamine; LPI, lysophosphatidylinositol; MMPE, monomethyl-phosphatidylethanolamine; OxLPC, oxidized lysophosphatidylcholine; OxLPE, oxidized lysophosphatidylethanolamine; OxPC, oxidized phosphatidylcholine; OxPE, oxidized phosphatidylethanolamine; PC, phosphatidylcholine; PE, phosphatidylethanolamine; PG, phosphatidylglycerol; PI, phosphatidylinositol; PS, phosphatidylserine; Cer-AP, ceramide alpha-hydroxy fatty acid-phytospingosine;; Cer-AS, ceramide alpha-hydroxy fatty acid-sphingosine; Cer-BDS, ceramide beta-hydroxy fatty acid-dihydrosphingosine; Cer-BS, ceramide beta-hydroxy fatty acid-sphingosine; Cer-NDS, ceramide non-hydroxyfatty acid-dihydrosphingosine; Cer-NP, ceramide non-hydroxyfatty acid-phytosphingosine; Cer-NS, ceramide non-hydroxyfatty acid-sphingosine; NeuAcα2-3Galβ1-4Glcβ-Cer, ganglioside; HexCer-AP, hexosylceramide alpha-hydroxy fatty acid-phytospingosine; HexCer-NDS, hexosylceramide non-hydroxyfatty acid-dihydrosphingosine; HexCer-NS, hexosylceramide non-hydroxyfatty acid-sphingosine; SM, sphingomyelin; CE, cholesteryl ester; Co Q; coenzyme quinone.
