# Supplementary material for: The n-3 PUFA content of the global lipidomes of NIST SRM 2378, SRM 1950, and intralaboratory quality control materials
Source: J Lipid Res. 2025 Dec 26;67(2):100970. doi: 10.1016/j.jlr.2025.100970 (PMC12861023; doi:10.1016/j.jlr.2025.100970)
Supplement: Supplementary Figures — s [file mmc3.docx]

**
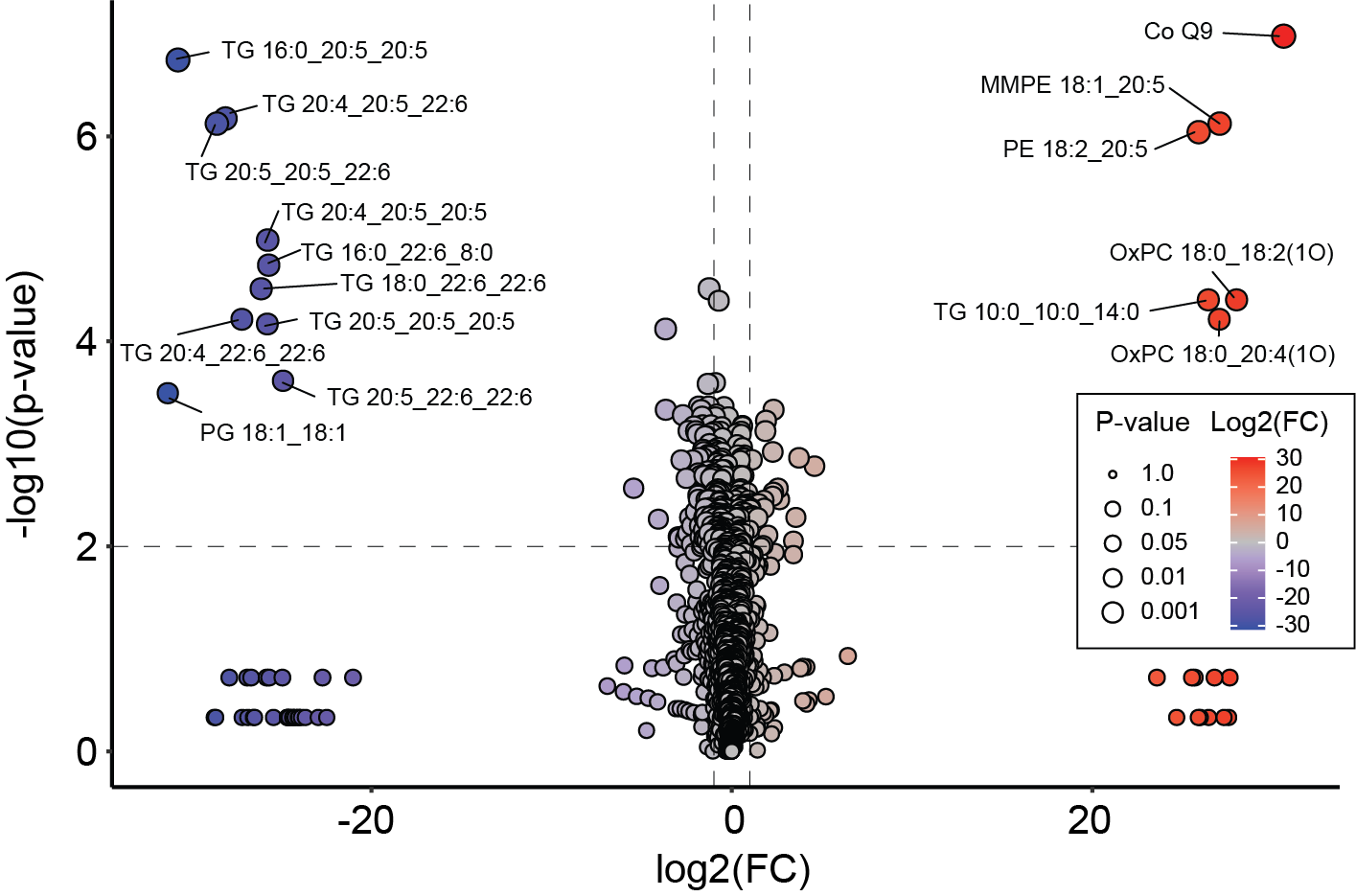
**

**Figure S1**. Volcano plot of differences between SRM 2378-3 (serum) vs. SRM 1950 (plasma by heparin) reference materials. Data was log-transformed and pareto scaled as significance was determined using a fold change threshold of 2.0 and a false discovery rate (FDR) p-value threshold of 0.01. n = 83 lipids were lower in SRM 1950, n = 31 lipids higher in SRM 1950. SRM, National Institutes of Standards and Technology Standard Reference Material. TG, triacylglycerol; Ox, oxidized; PG, phosphatidylglycerol; OxPC, oxidized phosphatidylcholine; CoQ, coenzyme quinone.

**
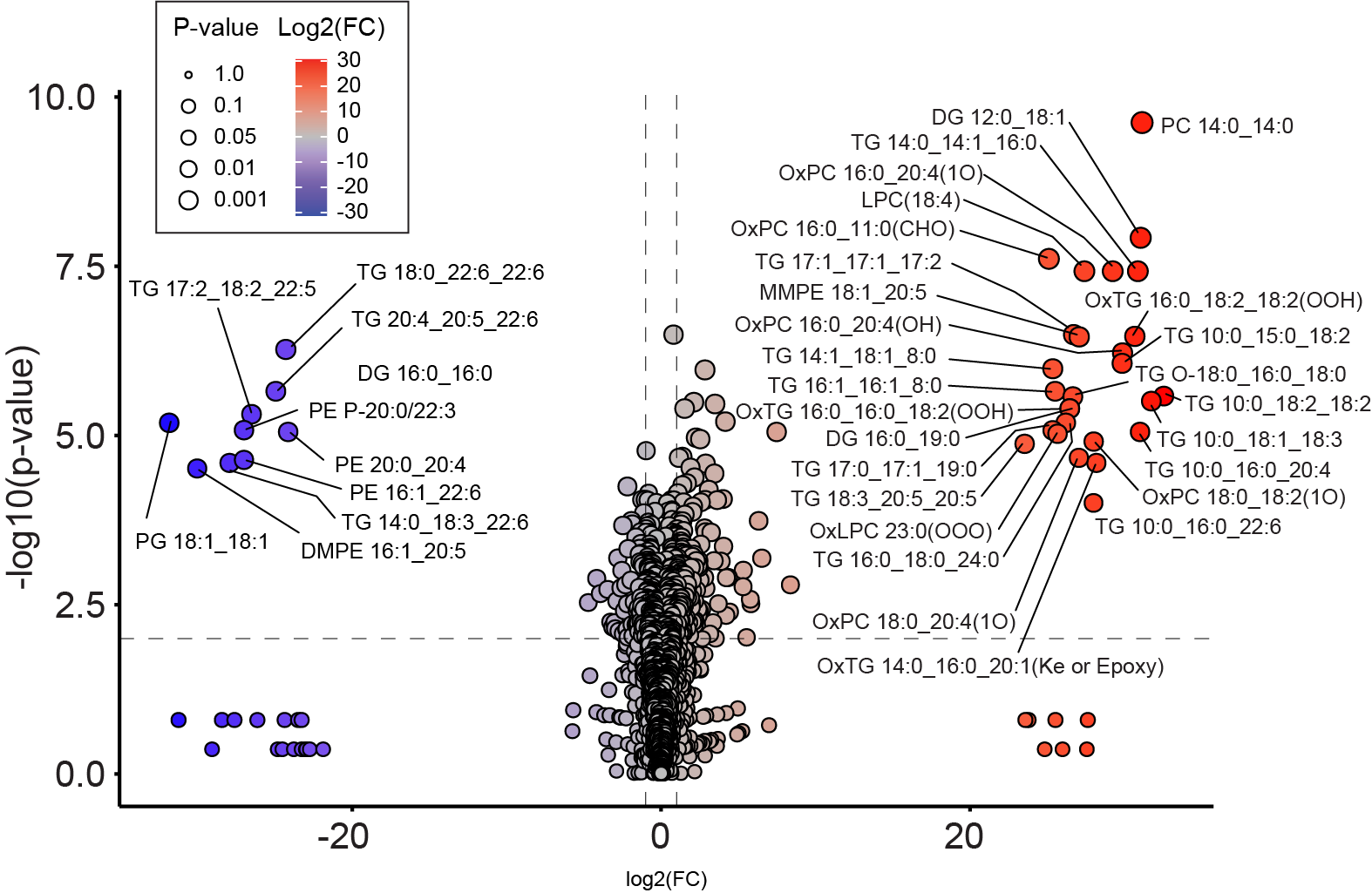
**

**Figure S2**. Volcano plot of differences between ILQC LO3 (plasma by EDTA) vs. SRM 1950 (plasma by heparin) reference materials. Data was log-transformed and pareto scaled as significance was determined using a fold change threshold of 2.0 and a false discovery rate (FDR) p-value threshold of 0.01. n = 74 lipids were lower in SRM 1950, n = 174 lipids higher in SRM 1950. ILQC LO3, intra-laboratory quality control low omega-3; SRM, National Institutes of Standards and Technology Standard Reference Material. TG, triacylglycerol; DG, diacylglycerol; PE, phosphatidylethanolamine; PE P, plasmenyl phosphatidylethanolamine; DM, dimethyl; MM, monomethyl; Ox, oxidized; PG, phosphatidylglycerol; PC, phosphatidylcholine; LPC, lysophosphatidylcholine.

**
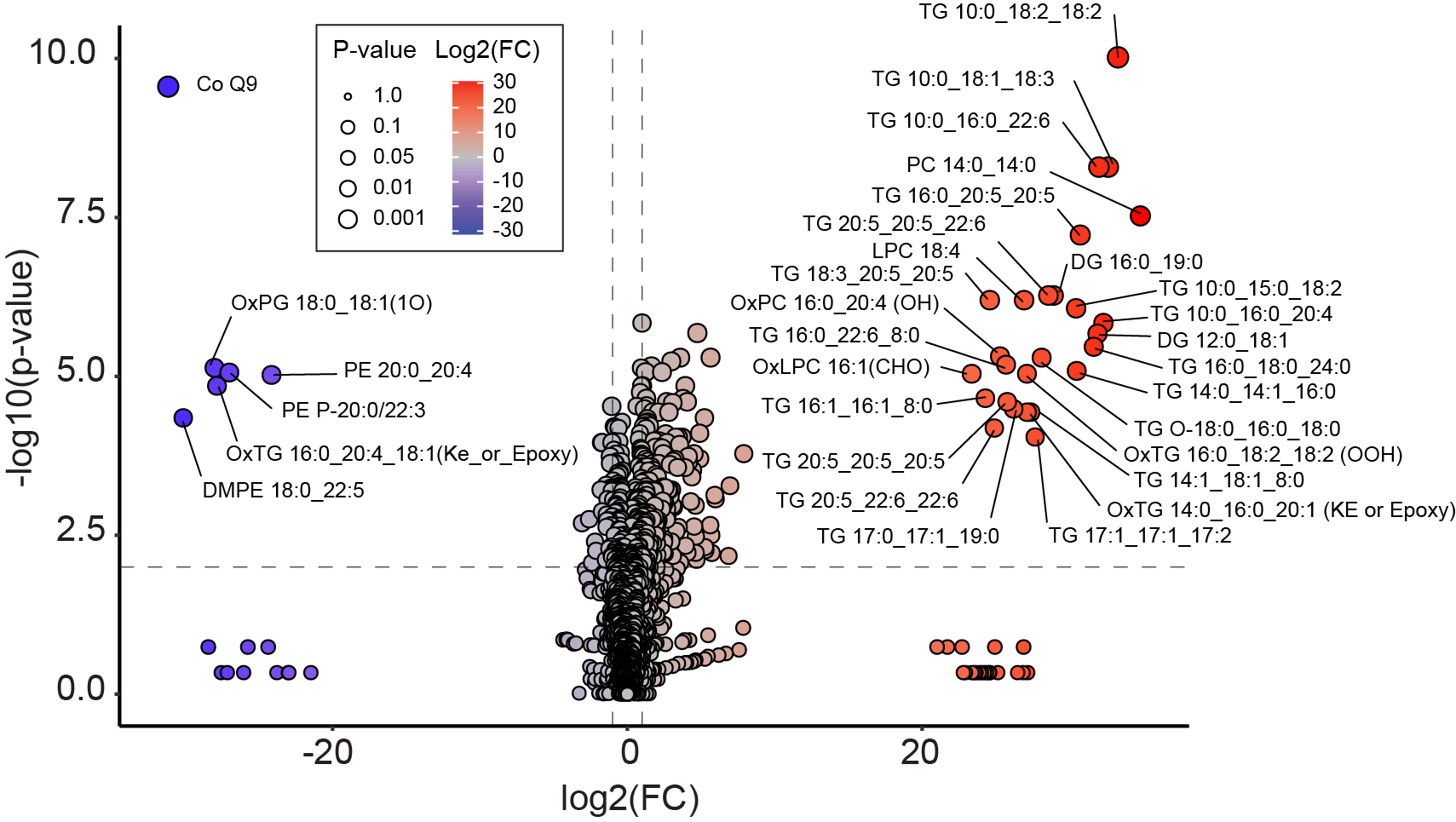
**

**Figure S3**. Volcano plot of differences between SRM 2378-3 (serum) vs. ILQC LO3 (plasma by EDTA) reference materials. Data was log-transformed and pareto scaled as significance was determined using a fold change threshold of 2.0 and a false discovery rate (FDR) p-value threshold of 0.01. n = 36 lipids were lower in SRM 2378-3, n = 183 lipids higher in SRM 2378-3. ILQC LO3, intra-laboratory quality control low omega-3; SRM, National Institutes of Standards and Technology Standard Reference Material. CoQ, coenzyme quinone; TG, triacylglycerol; DG, diacylglycerol; PE, phosphatidylethanolamine; PE P, plasmenyl phosphatidylethanolamine; DM, dimethyl; MM, monomethyl; Ox, oxidized; PG, phosphatidylglycerol; PC, phosphatidylcholine; LPC, lysophosphatidylcholine; TG O, plasmanyl triacylglycerol.
